# Supplementary material for: Understanding 6th-century barbarian social organization and migration through paleogenomics
Source: Nat Commun. 2018 Sep 11;9:3547. doi: 10.1038/s41467-018-06024-4 (PMC6134036; doi:10.1038/s41467-018-06024-4)
Supplement: Supplementary file 3 — Description of Additional Supplementary Files [file 41467_2018_6024_MOESM3_ESM.pdf]

Description of Additional Supplementary Files

File Name: Supplementary Data 1
Description: Summary statistics for samples sequenced in this study.

File Name: Supplementary Data 2
Description: List of previously published ancient samples used in this study.

File Name: Supplementary Data 3
Description: Alignment of the variable SNPs from 37 medieval and 1 Bronze age individuals.

File Name: Supplementary Data 4
Description: Most likely modern population assigned to each ancient sample test using PAA.

File Name: Supplementary Data 5
Description: . Kinship coefficients and ks for putatively related individuals.
