# Supplementary material for: Understanding 6th-century barbarian social organization and migration through paleogenomics
Source: Nat Commun. 2018 Sep 11;9:3547. doi: 10.1038/s41467-018-06024-4 (PMC6134036; doi:10.1038/s41467-018-06024-4)
Supplement: Supplementary file 1 — Supplementary Information [file 41467_2018_6024_MOESM1_ESM.pdf]

Supplementary Information

Understanding 6th-Century Barbarian Social Organization and Migration through Paleogenomics

Amorim et al.

SUPPLEMENTARY NOTE 1. BRIEF HISTORY OF THE LOMBARDS AND THE MIGRATION PERIOD

Walter Pohl, Patrick J. Geary, Cristina La Rocca

The Longobardi first appear in Roman texts early in the first century of the Common Era (CE), when the Roman historian Velleius Paterculus, writing about the military expedition of Tiberius around 10 CE in which he himself participated, listed among the peoples defeated by the Romans the "Longobardi, a people surpassing even the Germans in savagery." The geographer Strabo, writing around the same time, considered the Longobards a part of the Suebi and stated that they were at present living on the eastern banks of the Elbe. He described their lifestyle as essentially nomadic, saying that they lived for the most part from their flocks dwelling in temporary huts and packing all of their goods into wagons when moving. At the end of the first century, the Roman historian Cornelius Tacitus, in his *Annales*, briefly describes the Longobardi along with the Semnones as among those who revolted against the Romanized Suebian king Maroboduus around 17 CE. In his *Germania*, written around 98, his only mention of the Longobards is the terse comment that they are distinguished by their small numbers and find safety only in battle. After this the Longobards disappear from Roman texts; only Cassius Dio, writing in the early third century, states that during the reign of Marcus Aurelius (161 to 180), six thousand Longobardi and Obii crossed the Danube but were soundly defeated, sued for peace, and then returned home[1].

The name of the Longobardi reappears ca. 490 again beyond the Danube, but these Longobardi are, according to the Byzantine historian Procopius, a Christian people under the domination of the Heruli, a barbarian people that in the course of the later fifth century occupied

territory in what is now northern Austria and the southern Czech Republic. Procopius further wrote that ca. 508 the Longobards defeated the Heruli, killing their king Rodulf and driving out the Heruli. These territories south of the Danube were in the former Roman province of Pannonia, from which Roman administrative and military infrastructure had been removed in the course of the fifth century. Pannonia had fallen first under Hunnic, then under Ostrogothic control, when the Ostrogoths moved into the Balkans in 473. Soon after, in 476, Odoacer, a Roman commander of barbarian origin in Italy who led a mixed army of Heruli, Rugii and others, overthrew the last West Roman emperor and ruled Italy as king and patricius by arrangement with the East Roman emperor in Constantinople. He exerted loose control over the former Roman provinces to the north and east including Pannonia. The population of the region thus presumably comprised descendants of Roman provincials as well as members of the various barbarian populations that had entered the region over the previous two centuries[2–4].

In 493 the Ostrogothic king Theoderic the Great, acting on the orders of the Roman Emperor Zeno, who had made him patrician and master of the military in the West, killed Odoacer and took over the Kingdom of Italy which he ruled nominally as a representative of the Emperor until his death in 526. Taking advantage of a troubled succession within the Ostrogothic kingdom, in 535 the Byzantine (East Roman Empire) invaded Italy and began a reconquest under Emperor Justinian I. From around this time, Byzantine diplomacy began to develop treaties with the Longobards in order to isolate the Ostrogoths and to counter the Gepids, another barbarian people along the Danube and Tisa Rivers, and granted them the "city of Noricum and the fortifications in Pannonia as well as other towns and a great amount of money". This probably took place around 547/48, and legitimized the entry of the Longobards into the former Roman

provinces south of the Danube. Thereafter the fortunes of the Longobards were progressively intertwined with those of the Romans: Romans sided with the Longobards in their increasingly serious conflicts with the Gepids, and the Longobards provided troops to aid the Romans against the Ostrogoths. After the death of Justinian in 565 and the succession of Justin II, however, Roman policy shifted to support for the Gepids and in 566 the latter defeated the Longobards with Roman support. This drove the Longobards into an alliance with the central Asian Avars who had newly arrived in the vicinity of the Carpathian basis east of the Gepids, an alliance that made possible the Longobard's final defeat of the Gepid kingdom in 567[2].

King Alboin did not use his victory to build a hegemonial kingdom along the Middle Danube (as the Avars would soon do), but to assemble a large army to invade Italy. Later Longobard texts date the beginning of this march to April 1, 568. Even though some historians have argued that it may also have happened in 569, we can be rather precise about the date, and also about the route taken along the ancient Roman Via Postumia to Aquileia and Verona, even though no archaeological evidence survives for these events. Seventh-century sources attribute the Longobard invasion of Italy in 568 to an invitation issued to the Longobard King Alboin by the Roman commander Narses, although this is not mentioned in sixth-century sources and is greeted with skepticism by modern scholars[2],p. 98-100). It was clear that the battered infrastructure in Pannonia could not meet the ambitions of a growing Longobard army, which now had also incorporated part of the defeated Gepids. Later sources claimed that Alboin had left his former kingdom to his Avar allies. The invasion met with surprisingly little organized Roman resistance, but still the Longobard conquest of parts of Italy was a poorly organized and long-drawn out affair. The main army moved westward and took Pavia, which would later

become the Longobard capital, after a siege, but did not move on to attack Ravenna or Rome. Instead, Alboin's army began to fall apart into separate bands led by individual dukes who went their own ways, some into southern Italy and others into Burgundy, and some straight into Roman service. Alboin and his immediate successor were both assassinated, and unity, at least in the north, was only reestablished after a decade by Authari and his Bavarian wife Theodelinda, a descendant of an earlier Longobard king. Upon Authari's death in 590, Theodelinda then married his successor, Agilulf (591-616), duke of Turin but described by sources as a Thuringian. In spite of its consolidation, the Longobard kingdom only controlled the North of Italy with the exception of the modern Romagna around Ravenna, and Tuscany; the Longobard duchies of Spoleto and Benevento ruled much of the inland areas of the peninsula, while the coastal strips and the land around Ravenna and Rome remained under Roman control[3,5].

Under the patronage of Theodelinda, Secundus of Non/Trent, wrote a short history of the Longobards that has subsequently disappeared. During dynastic disputes in the second half of the seventh century, the Origo Gentis Langobardorum, a brief account of the mythic origins of the Lombards written within the context of rival dynastic interests, presented their origins in "Scandanan," where they carried the name of the Winnili. Attacked by the Vandals, they obtained favor from Wodan through the intervention of his wife Frea, and acquired the name Longobardi or long-beards. The text then tells of their migration through various regions before establishing themselves in the former land of the Rugii, where they fought with and defeated the Heruls and then came into conflict with the Gepids. The text details the various royal marriage alliances with Gepids, Thuringians, Franks, and Herules prior to the movement of the Longobards into Pannonia. It tells of the final defeat of the Gepids in which King Alboin killed

the Gepid king Cunimund, the king's marriage with the daughter of Cunimund, Rosemund, and the invasion of Italy. This text makes no mention of alliances with the Byzantines nor does it credit the invasion of Italy to an invitation from Narses[5,6].

At the end of the eighth century, after the Longobard kingdom had been conquered by the Frankish king Charles the Great (Charlemagne) in 774, the Longobard cleric Paul the Deacon wrote a much fuller history of the Longobards[7], drawing on the *Origo Gentis Langobardorum*, the now-lost history of Secundus, and other seventh-century sources. His account, although written over two centuries or more after the events it recounts, has nevertheless been taken, often uncritically, as a reliable account of Longobard history, a position increasingly disputed[8,9]. Concerning the invasion of Italy, he states that Alboin's invading army included not only Longobards but Gepids, Bulgars, Sarmatians, Pannonians, Suevi, Noricans, and others[6,10].

The social structure of pre-Migration Longobard society is extremely difficult to reconstruct. Much depends on whether one sees the seventh-century legal compilations, and in particular, the Edict of King Rothari promulgated in 643 as containing some indication of pre-migration social and cultural organization. Judging from the compensation tariffs attached individuals, the society was divided into three strata: free-born (termed *exercitales* or soldiers in later laws); *aldii* or clients, and *servi*, slaves [11]. An important but poorly understood social institution was the *fara*. Marius of Avenches describes the invasion of Italy as having taken place *in fara*, and the Edict of Rothari includes a provision for a free man to move about "with his *fara*." A number of place names, both in Italy and in other regions such as Burgundy occupied by barbarian armies include the term *fara*, as do some personal names from Burgundy, although the interpretation of this evidence is uncertain. By the late eighth century, Paul the Deacon

defined *fara* as a kin group. However recent scholarship has argued on etymological grounds that the term originally designated not a kindred but a small, mobile military unit, which only acquired the meaning of a kindred following the establishment of the Longobard Kingdom in Italy[4,12–16].

SUPPLEMENTARY NOTE 2. ARCHAEOLOGICAL CONTEXT OF SZÓLÁD

Tivadar Vida, Uta von Freeden, Daniel Winger

This Longobard-associated/-period cemetery is located above the modern village of Szólád in the Somogy County in present day Hungary, about 5 km south of Lake Balaton (latitude 46º17 ', Longitude 17º51') in a 30 km long and at this point about 400-600 m wide valley of the Transdanubian hills. It is situated on a Löss-slope inclined to the south, which is covered with about 40-50 cm thick calcareous Chernozem (black earth). The valley today consists of a marshy lowland that reaches to Lake Balaton. It is a now silted-up branch of the lake[17] which means that in Lombard time the actual shore of Lake Balaton was near the cemetery and the associated settlement which was situated most probably on a terrace beside the bay. Already before and during the highway excavations in 2003, some further Longobard sites were discovered in the region around Lake Balaton (Supplementary Figure 1). Most of the Longobard-period burial grounds in Pannonia are located near former Roman villas, forts and camps, and the Szólád cemetery seems not to be an exception, since there are hints of a Roman villa nearby.

The first grave was discovered in 2003 during the development of an access road from Szólád to the M7 motorway (Grave 1[18]). Preceding the excavation of the burial ground, extensive geomagnetic prospecting took place (in 2005 and 2007 conducted by the Thuringian State Office for the Preservation of Monuments and Archeology, Weimar, and in 2009 and 2011 undertaken by the Römisch-Germanische Kommission, D. Winger), which also included the surrounding area north and south of the cemetery and in the probable settlement area.

In 2005, an international research team began the archeological investigation of the sixth century cemetery. It consisted of the Institute of Archaeology of the Hungarian Academy of

Sciences (lead by Tivadar Vida) in cooperation with the Römisch-Germanische Komission des Deutschen Archäologischen Instituts in Frankfurt (lead by Uta von Freeden and from 2010 by Daniel Winger). The project was also joined by the Anthropological Institute of the Johannes Gutenberg-Universität Mainz (from 2008 Kurt W. Alt) to conduct the physical anthropological, mitochondrial and isotopic analyses.

The burial ground can be considered as completely excavated: borders are recorded on three sides, only in the southwest there are small inaccessible plots, on which perhaps very few burials could still lie. Even around grave 44, which is located separately in the south, there was no indication of further burials in a review of the environment in the last year of excavation. The excavation also revealed numerous Neolithic, Iron Age and Avar period structures.

The bone preservation in the calcareous soil was excellent and allowed scientific analyses, which to some extent were performed for the first time on early medieval populations. The cemetery contained forty-five graves from the sixth century. Sixteen were the burials of adult men. Grave goods suggested that boys and male juveniles had been interred in seven graves, and the anthropological analyses revealed that four graves without any grave goods had been male burials too. Males were buried in a well-defined cluster in the middle/western part of the cemetery, while the female burials (ten adult women and two young girls) are situated around the male graves in a semi-circle in the south-eastern part (Supplementary Figure 2). The number of child and infant burials was surprisingly high – possibly also due to the careful excavation technique.

The burials were up to 4.5 meters deep in the loess, and partly designed elaborately with wood. Graves of women and children are generally less deep, but correspond in their dimensions

and structures to those of men[19]. Different findings and grave forms – partly observed for the first time in Hungary – are identical for both sexes: graves with ledges and straight walls, wood interiors, grave borders in the form of trenches, as well as plank and tree coffins are documented. The equipment with grave goods can be regarded as very high quality and extensive – despite the also documented grave disorders and the reopening of interments, that took place already in ancient times in approximately 40% of the graves.

Our knowledge regarding the period's archaeological material and especially regarding the funerary practices and grave constructions was substantially enriched by the Szólád excavations. After the removal of the humus, the soil stains outlined 45 graves, including six graves ringed by a circular ditch (e. g. grave 30, Supplementary Figure 3) and two graves enclosed by a rectangular ditch (e. g. grave 5, Supplementary Figure 4). Grave ditches of this kind were previously unknown in Pannonia. Comparable round grave ditches are known from Merovingian cemeteries in central Europe[20] and may be interpreted as an indication of a burial mound raised over the grave of high-status individuals. Interestingly enough, comparable rectangular grave ditches have only been reported from Gaul in Saint-Vit[21], St. Pölten-Unterradlberg in Austria[22] and Britain in Burton Farm[23], both former provinces of the Roman Empire and newly from Hiddestorf in Northern Germany[24]. Such rectangular grave structures possibly imitate Late Antique burial customs.

A not entirely new phenomenon in the Longobard period grave constructions in Pannonia are graves with ledges on the long sides of the grave pits. 32 of the 45 burials in Szólád had such ledges, to be seen for example in grave 31 (Supplementary Figure 5). Their average depth turns out to be gender-dependent: around 3 m for men; just under 2.5 m for women; round about 2 m

in the cases of child burials. The width of the pit narrows to the bottom: the average value of the 32 has an upper width of 128 cm and a lower width of 60 cm.

Excavation pictures illustrate what these ledges on the long sides could have served for. In the case of grave 4 (Supplementary Figure 6) with a square enclosure, a completely intact beamed ceiling was uncovered. The beams rested on the heels on the long sides. On this wooden structure grave goods were deposited, for example, food gifts.

As shown by the intact timbers of this undisturbed grave and the broken planks of a the robbed burial 24 (Supplementary Figure 7), the chamber was covered and furthermore protected by such wooden constructions. Comparable constructions are known from Merovingian Europe as well as contemporary Pannonia (e.g. Szentendre). This burial type also survived in Italy, where in some cases the wooden steps were replaced by stone and the 'chamber' was covered with stone slabs (as at Castel Trosino[25]). Most graves contained tree coffins e g. grave 13 (Supplementary Figure 8), a few burials yielded the remains of rectangular plank coffins e. g. grave 38 (Supplementary Figure 9), reflecting only slightly different funerary customs.

The smaller number of twelve graves with straight walls are only deepened from 1.60 to 0.65 m. They are found clustered in the southeast of the cemetery – except for burial 19. With such a shallow depth, it probably does not make sense to subdivide the long sides with ledges. The distribution of tombs with ledges and those with straight walls marked a clear subdivision of the burial ground. This is reflected in the archaeological material as well: graves with straight walls contained hardly any grave goods. Exceptions are the atypical adult female grave 19 with a bracelet (Supplementary Figure 10) and the girl grave 38 – which contained a bracelet as well (Supplementary Figure 9).

Female burials generally contained up to four brooches (Supplementary Figure 11), which is typical for this period's female costume in Merovingian Europe. Bigger bow brooches hung from the belt. Parallels to the small S-shaped brooches worn on the torso are known from both Pannonia and from today South Germany, as well as from Italy and Slovenia. The technological and decorative traits of the S-shaped brooches and the bow brooches with rectangular head plate suggest that these jewelry items represent the emerging phase of the so-called Pannonian-Italian style, an independent phase of local Pannonian metalwork[26]. Beads and pins are also part of the female dress, whereas pottery and combs were deposited in both male and female burials. Weapons on the other hand are parts of purely male equipment. The number of weapons deposited in the Szólád graves is unusually high compared to other burial grounds in Pannonia. Nearly every male burial contained at least one weapon, and the joint occurrence of spatha, spear/lance and shield was quite common. Spathas were especially frequent, deposited in about 60 percent of the male burials, reflecting a strongly military organization (and, indirectly, the military nature of the sixth century society).

In addition to vessels of northern origin, the grave pottery from Szólád includes local Pannonian wares such as stamped vessels, and spouted vessels with smoothed-in decoration[27]. The community's contacts with faraway regions are reflected by finds of an ivory ring from Grave 38, the fragments of a Mediterranean glass chalice from Grave 30, or the weights and scales of the male buried with his horse in grave 13.

Grave 19, a grave with straight walls, contained the burial of a woman in a wooden plank coffin, whose single grave good was a bronze bracelet on her left arm (Supplementary Figure 10). This custom has been observed among the Romanized populations of Pannonia, the Eastern

Alpine region and the Mediterranean. The woman came perhaps from one of the surviving Late Roman communities, and the strontium isotope analyses of this individual indeed showed a completely different background compared with the other individuals. Traditionally, most tombs would be considered as Longobard due to the furniture with weapons and brooches, the food offerings, the pottery, the grave robbery, and the dating in to the mid-sixth century (i.e. fitting to the historical knowledge of this region). But the case of grave 19 (or grave 38) (Supplementary Figures 9-10) as well as different grave constructions (straight walls, rectangular grave enclosures) illustrates the difficulties of the interpretation of early medieval societies solely by archaeological means. It is obvious that there is something new, foreign or at least different in general, but the different aspects within the cemetery show also the variety of the interpretation of any individual identity. For this reason in previous publications on Szólád, the term Longobard period instead of the pure ethnic Longobard was used.

As noted by Alt et al.[28] the burial ground was used only for a single generation in the middle of the sixth century – based on archaeology as well as selected radiocarbon dating (see below)– without any antecedent or successor.

The findings in Szólád show a relatively rich, well-nourished population of the mid-sixth century with long distance contacts to modern South-, West- and Central-Germany, to Moravia and Austria, to Slovenia and northern Italy. There are no older traditions known in Pannonia regarding the grave constructions or grave goods: something new or foreign occurs in this cemetery. But at the same time different further, perhaps late Roman, perhaps local, influences are integrated in this burial society.

Since it was a research excavation, anthropological and scientific methods were integrated into the archaeological project as early as possible in order to obtain all imaginable information in an exemplary way – only few can be mentioned here. Physical anthropological, ancient DNA (aDNA) and isotope analyses offered many new possibilities for a better understanding of this community[29]. This includes classical analysis like age, sex, and state of health as well as isotopic analyses for the reconstruction of dietary habits and possible origin of individuals[28].

In summary, the small Longobard period cemetery from Szólád is the necropolis of a small, wealthy, highly mobile and population from the middle of the sixth century. In grave construction and grave goods at least two groups can be distinguished, which suggest the integration of different traditions. The small population settled for only one generation in Pannonia at the shore of Lake Balaton and therefore appears to have been very mobile - especially the women indicate isotopically and genetically heterogeneous backgrounds[28].

For new radiocarbon (^{14}C) dating performed in this study, collagen was extracted from bone material for 8 samples (including 2 lacking grave goods, SZ37 and SZ43) at the Curt-Engelhorn-Center for Archaeometry, purified by ultrafiltration (fraction >30kD) and freeze-dried. Collagen was then combusted to CO_2 in an Elemental Analyzer (EA). CO_2 was then converted catalytically to graphite. Dating was performed using the MIni CArbon DAting System (MICADAS-AMS) of the Klaus-Tschira-Archäometrie-Zentrum.

^{14}C ages were normalized to δ13C=-25%[30]. The ^{14}C ages were given in BP (before present) meaning years before 1050. In order to provide absolute calendar ages the ^{14}C ages were calibrated using the dataset INTCAL13 dataset[31] via the software SwissCal 1.0. Results of the

calibration are shown in columns “Cal 1-sigma” and “Cal 2-sigma” using the 1-sigma and 2-sigma uncertainty of the ^{14}C ages, respectively in Supplementary Table 1. C:N ratios and C concentrations were within the range of normal values, except for samples SZ43, SZ21, SZ27B and AV2, which have elevated C:N ratios that could indicate degraded collagen and negatively affected ^{14}C ages. Collagen content of the sample material was considered good. All samples dated to the early medieval (range of 412-604 CE across all samples and both sigma estimates for our Lombard-era samples and 541-641 for the two Avar period samples).

SUPPLEMENTARY NOTE 3. ARCHAEOLOGICAL CONTEXT OF COLLEGNO

Caterina Giostra, Luisella Pejrani Baricco, Elena Bedini

Collegno is located 7 km west of the city of Turin, in Piedmont, near a crossing of the river Dora. It is located along the road leading to Val di Susa and the Alpine passes to Gaul which in the later sixth and early seventh centuries were controlled by the Frankish kingdom.

Between 2002 and 2006, the then Superintendence for Archaeological Heritage of Piedmont investigated an extensive area during the works for the construction of the Torino subway. A long-standing village and two funerary areas were found at the archaeological site (Supplementary Figure 12) containing a group of eight tombs that chronologically and culturally suggested Gothic presence, including two cases of artificial modification of the skull (Supplementary Figure 13) and a large cemetery from the Longobard period. The village related to the funerary contexts was also in use in both the Gothic and the Longobard periods as evidenced by the presence of sunken-feature buildings (Supplementary Figure 14) and stamped pottery of northern European tradition[32–34].

The necropolis was completely excavated; it contained 157 graves (Supplementary Figure 15), although the loss of some superficial burials cannot be excluded because of modern agricultural activities. Some small sectors and some burials were damaged before the start of the archaeological excavation. The graves have a broad chronology, which stretches from the late sixth to eighth centuries. We can recognize a progressive expansion from the center to the outside, toward both the east and the west; only in the last phase (8th century) burials are again located among the central graves.

The use of the cemetery can be divided into three phases based on grave typologies,

objects found in the tombs and location plausibility:

- Phase 1: 570/590 – 630/640 (orange on the plan, Supplementary Figure 15). It is not easy to establish precisely when burials began. It is possible that they started a few years after the arrival of the Longobards in Italy. Regardless, the first phase has a broad range and we can identify two sub-phases within the first phase: 1A: end of the sixth century - first years of the seventh century, 1B: early decades of the seventh century.

- Phase 2: 640 - 700 ca. (green on the plan). During this time, the eastern and western most sectors of the cemetery were used.

- Phase 3: eighth century (grey on the plan). During this period, new graves without grave goods are placed once again in the central areas of the cemetery, among the existing oldest graves. The chronology has been confirmed by radiocarbon analysis of individuals from this phase , which was performed at Beta Analytic Radiocarbon Dating Laboratory in Miami, Florida (see Pejrani Baricco[32]). Estimates from calibration curves ranged from 675 to 880 CE with 95% probability, and from 700 to 795 CE with 68% probability.

In the early stages of cemetery use (1A and 1B), the determination of sex and age on an anthropological and archaeological basis according to Ferembach et al.[35] shows that the number of male individuals is clearly higher than the number of female individuals; children and adolescents consist of only 20% of the graves[36]. The graves of men, women, and children are integrated, and this likely reflects the organization and use of space based on parental ties.

The graves are oriented along an west-east axis, with the head of the deceased to the west. They are often aligned along short lines with a north-south development. The graves of the first phase (Supplementary Figure 16) are pits in the earth not deeper than 1m from the present

ground elevation. Among the oldest graves (Phase 1A; up to 600-610 ca.) several contained wooden chambers, but these disappear after the first decades, in line with what generally occurred in Italy. Thus graves dating from 600 to 630/640 (Phase 1B) lack these wooden chambers. Various male graves have weapons sets (Supplementary Figure). Progressively belts for the suspension of weapons acquire more numerous seals which are decorated with the animal style (Supplementary Figure 18)[37,38]. The female tombs often appear to have few grave goods, perhaps because of the age of little girls or older women, and also in line with the evolution of Italian funeral rituals in the 7th century. Among the offerings one finds pottery of the stamped type already produced in Pannonia. Even a horse was also found, which was buried headless.

The necropolis at least in part has been associated with the Longobards because of these practices and because of the material culture encountered (typologies, production features and decorative style), along with the typology of the sunken-feature buildings in the settlement. These elements present the following traits: a clear discontinuity with respect to the previous Italian context in which these distinctive features are absent; continuity with respect to areas inhabited by barbarian groups, especially the Longobards in Pannonia; internal consistency with respect to these factors from the same site and absence in other coeval sites in the same area; coexistence with the arrival of migrant groups described by written sources (end of sixth century)[39].

There appear to be two older nuclei, one in the center (1A), which expands to the west (1B; 2), and a more eastern one (1, A) that expands to the east (1, B; 2) (Supplementary Figure). This second nucleus is centered on a row of tombs with gold foil crosses (Supplementary Figure 15). The horse was found within this group. Some female tombs (CL48, CL47, CL147 and

perhaps others) contained non-Italian but transalpine objects (Supplementary Figure 20) which suggested the presence of some individuals of a different origin that differs from both Italy and Pannonia, especially women who may have moved from neighboring regions (possibly from the Frankish-Burgundian area in south-eastern Gaul, starting from the nearby Val di Susa) due to exogamy, perhaps accompanied by some subordinate individuals[37]. Strontium isotope data suggest a non-local origin different from the other individuals for women CL48 and CL147. Unfortunately it was not possible to obtain DNA from CL48, but they may be linked with neighboring grave CL47. Anthropological analysis has shown that the skulls of women CL48 and CL47 both had probably hereditary scaphocephaly, making kinship more credible (Supplementary Figure 21).

The two men from graves 49 (also a non-local individual, based on strontium isotope data) and 70 (with grave goods very similar to the tomb 53) both had an inherited pathology, DISH (Diffuse Idiopathic Skeletal Hyperostosis), allowing us to potentially enlarge the network of parental relationships in this group of graves. In the necropolis, even the possible symbolic transmission of some objects, especially parts of belts, has allowed us to speculate on possible relations of kinship between some individuals[36].

Some graves, however, are distinct as they lack wooden chambers, weapons, or objects that are barbarian by type and decoration; these are found in both marginal sectors and between the two different kin groups with these elements (Supplementary Figure 19).

Skeletal remains have different degrees of preservation, with individuals from the first phase tending to be worse. Metric and morphometric analysis on six individuals has found the presence of elongated skulls, in a marked or intermediate measure[40]. Male individuals are

especially tall, with almost all individuals reaching or exceeding 170-175cm. Skeletal analysis published in 2004 suggested aspects of lifestyle which appear to have changed significantly in the second period and even more in the third[36]. In the first phase, men with weapons show a marked muscular development and skeletal alterations that indicate an intense physical activity, including evidence of horseback riding. At least 4 individuals of the first phase have cranial lesions produced from cutting of the weapons (Supplementary Figure 22). The data suggest an intense military training and even fighting.

In the second phase (640-700 ca.) the physical effort extended was still stressful, but less risky than in the previous phase: none of the few traumas found appear to have been produced by aggression. In the third period of use of the necropolis (eighth century) both the degree of muscular development and the traumatic pathologies, while still indicative of a discrete degree of physical stress, are different from those of previous phases and due to a complete change of lifestyle. The men seem to have engaged in agricultural and craft work[36].

Finally, in 2004 all the adult individuals from the first 73 graves already excavated at that time were subjected to paleonutritional analysis by atomic absorption spectroscopy (AAS) at the Laboratory of Paleonutrition of the Department of Archaeological Sciences of the University of Pisa[41]. Also the organic remains adherent to the metallic finds were analyzed by light microscopy[42].

Skeletal remains are currently stored in the warehouse of the Dipartimento di Biologia dell'Università di Firenze and owned by Soprintendenza Archeologia, Belle Arti e Paesaggio per la città metropolitana di Torino.

SUPPLEMENTARY NOTE 4. DNA ISOLATION, LIBRARY PREPARATION, SCREENING AND GENOME-WIDE CAPTURE

Stefania Vai, Alessandra Modi, Krishna R. Veeramah, Cosimo Posth

Experimental steps of DNA isolation, library preparation and enrichment were carried out at the Laboratory of Anthropology and Paleogenetics at the University of Florence (Italy), the University of Tübingen (Germany) and at the Department of Archaeogenetics at the Max Planck Institute for the Science of Human History in Jena (Germany) in facilities exclusively dedicated to ancient DNA analysis. Appropriate criteria to prevent contamination with present-day DNA were followed. DNA extraction and library preparation reactions included negative controls at all stages.

Bone samples, namely the petrous portion of the temporal bone (henceforth petrous bone), teeth, and long bone fragments, were collected for 47 individuals from Szólád and for 36 individuals from Collegno. To remove potential contamination, the outer layer of the samples was mechanically removed using a dentistry microdrill with disposable tools and irradiated by ultraviolet light (254 nm) for 45min in a Biolink DNA Crosslinker (Biometra™). Petrous bones were sectioned using a disk saw, and the densest part of inner ear part was selected as proposed in Pinhasi et al.[43]. The dentine portion from teeth and the inner part of dense compact tissue for bones were selected to obtain bone powder using a microdrill with disposable tips. 50-100 mg of bone powder was used for DNA extraction using a silica-based protocol that allows ancient DNA molecules to be efficiently recovered even if highly fragmented[44]. DNA was eluted twice in 50 µl of TET buffer (10 nM Tris, 1 mM EDTA, 0.05% Tween-20).

As a first step, a screening was performed in order evaluate the quantity and quality of the endogenous DNA content. For this purpose, aDNA libraries were prepared from 20µl of extract following a custom double-indexing protocol[45,46] optimized for ancient samples, in order to make the DNA immortalized, barcoded and available for Next Generation Sequencing (NGS) on Illumina platforms. No enzymatic damage repair was performed at this stage in order to preserve and analyze the damage patterns of DNA fragments.

Libraries were shotgun sequenced on Illumina MiSeq and NextSeq runs (either single-end run 75 cycles in Jena or paired-end run 75 cycles in New York). Raw sequence data obtained from the sequencing runs were analyzed using pipelines specific for ancient DNA samples. In Jena the general approach of Peltzer was followed[47]. Adapters were clipped-off and the resulting reads of 30bp or longer were then mapped on to the human reference genome (hg19) using BWA[47,48] with the following parameters (-l 16500, -n 0.01). Duplicates were removed using DeDup, a tool that considers both ends of a DNA fragment to recognize them as clonal. In New York we largely followed the ancient DNA pipeline of Kircher[49], though slight modifications were made to scripts to accommodate our data. The first 50bp of each read was trimmed using the TrimFastQ.py script. TagDust[50] was then used to identify potential library artifact sequences based on known Illumina adaptor sequences. The thirty most frequent artifacts for each sample were identified and a pairwise alignment was constructed for each against the adaptor sequences. Pairwise alignments were then manually inspected for evidence of sequence motifs that likely represent artifacts such as forward and reverse adaptors containing no or very small inserts. This list of motifs was used as input for the MergeReadsFastQ_cc.py script in order to merge paired-end reads with substantial sequence overlap into single reads and remove

adaptor sequence. Any remaining paired-end reads were discarded. Reads with at least 5 base calls with a Phred-scaled quality score of less than 15 were removed using the QualityFilterFastQ_gz.py script. Reads were mapped to the the human reference genome GR37 with BWA aln[47,48] using the following parameters: maximum edit distance=1%, number of gap opens=2, l=16500. Read groups were added using the PICARD tool AddOrReplaceReadGroups and duplicate reads were marked using MarkDuplicates.

After mapping in both Jena and New York, reads with mapping quality below 30 were discarded. Length and deamination patterns were estimated using MapDamage 2.0[51]. Endogenous content of at least of 0.075% (though, using petrous bone this value was usually much higher, with a mean 36% and a max of 86%), deamination patterns compatible with the age of the individuals (above 20% C to T substitutions at 5' end) and the absence of contamination as evaluated from mitochondrial genomes were assessed when choosing samples for further processing. Relative coverage of reads mapping to the autosomes, X-chromosome and Y-chromosome were used to provide an initial assessment of sex.

A total of 39 samples from Szólád and 24 from Collegno showed endogenous DNA quantity and quality compatible with further genome-wide analyses. A second library with partial UDG treatment (UDGhalf)[52] was performed for 60 out of 63 selected samples starting from 30 µl of DNA extract (Supplementary Data 1). Ten libraries from Szólád, obtained from petrous bones genetically assigned to male individuals[53], showed enough endogenous DNA content (between 44% and 86%) to undergo whole-genome sequencing in New York on an Illumina HiSeq (100bp single-end run, 4 lanes per sample). In-solution capture[54] was performed on the remaining 53 individuals targeting ~1,237,207 single nucleotide polymorphisms (SNPs)

("1240K capture")[55] scattered across the nuclear genome. The enriched DNA was then sequenced on a NextSeq (75bp single-end run or 150 cycles paired-end run).

SUPPLEMENTARY NOTE 5. BIOINFORMATIC PROCESSING

Cosimo Posth, Carlos Eduardo G. Amorim, Krishna R. Veeramah

5.1. Mapping and Quality Control

Genome-wide capture read data was processed as described above in Supplementary Note 4 for screening libraries in Jena with the additional step for any paired-end data of merging reads into single sequence using Clip&Merge when there was a minimum overlap of 10bp between reads. WGS data was processed as described above in Supplementary Note 4 for screening libraries in New York with the additional step of realigning InDels using the GATK RealignerTargetCreator and IndelRealigner tools[56] and using KeyAdapterTrimFastQ_cc_gz.py to only remove adaptor sequence but not merge reads. All 63 individuals presented damage patterns as expected from the applied library protocol (Supplementary Data 1). Relative fold coverage on the targeted autosomal SNPs was compared to the coverage on the X- and Y-chromosome to confirm genetic sex assignment[57]. Nuclear contamination in males was performed with the tool ANGSD[58] by estimating heterozygozity levels on known X-chromosome polymorphic sites. Mitochondrial DNA (mtDNA) off-target reads were used, when possible, to reconstruct complete or partial mitochondrial genomes and estimate mtDNA contamination using schmutzi[59]. The online program Haplofind[60] was used for mtDNA haplogroup assignment.

5.2. SNP and Genotype Calling

Genotypes were called at SNPs targeted as part of the 1240K capture for all 63 ancient individuals from Szólád and Collegno. In addition, we called genotypes at the same sites for whole genome shotgun re-sequencing data generated from 7 third to seventh century individuals

(UDG treated) from the UK associated with the Anglo-Saxon material culture[61], 2 fifth century individuals (not UDG treated) from Bavaria, Germany[62], as well as 9 medieval samples from West Eurasia and 5 Scythians from eastern Hungary (not UDG treated) [63]. Thus we generated new calls for a total of 86 ancient individuals. We applied two different models for genotype calling (implemented in Python and available from https://github.com/kveeramah/) depending on if genomic libraries were subject to UDG treatment or not.

For the 67 samples that underwent partial UDG treatment (60 from the present work plus seven from Schiffels et al.[61]), we implemented a standard genotype likelihood model described by De Pristo et al[56], but trimming the last and first *B* bases of each read. *B* was set as 3 for the 60 individuals generated in this study and 5 for the 7 Anglo-Saxon-associated samples. By doing so, we sought to eliminate the excess of transitions due to PMD that remain at the end of reads due to partial UDG treatment. For the remaining 19 samples that did not undergo any kind of UDG treatment (3 from the present study, 2 from Veeramah et al.[62] and 14 from Damgaard et al. [63]), we called genotypes using a model taking into account PMG described in Hofmanova et al.[64], but considering a Weibull distribution[65].

Both models produced genotype likelihoods for each of the ten possible genotypes at the targeted 1240K capture SNPS. Because coverage for most samples was only ~1x, we obtained haploid genotypes for each individual based on the highest homozygote genotype likelihood. When likelihoods for two or more different homozygote genotypes were equal, we randomly picked one allele. We limited haploid genotype calling to those sites with a Phred-scaled quality score of at least 45 (except for the samples from Damgaard et al.[63], where we used a threshold of 20 as coverage was much lower compared to the sequences we generated). This value represents

the highest possible quality score when only one read covers a position (i.e. we discard any SNP calls where there is only one read and does not have the highest possible base quality score . Mean coverage at the captured regions (in samples undergoing 1240K capture only) ranged from <0.01x to 2.9x, with a mean of 1.5x and an average of ~520K SNPs successfully sequenced across these 53 samples from Szólád and Collegno.

We also determined full diploid genotypes at all sites in the genome (not just the targeted SNPs) for the 10 high-coverage genomes from Szólád. Mean coverage for these samples ranged from 6.8x to 14.5x, with a mean of 11.3x.

Finally, for analysis comparing ancient samples to each other directly, in order to not bias against samples with lower coverage (which will have a greater error rate), we obtained haploid calls at the 1240K capture SNPs for each individual by randomly sampling one read per site. Because of the potential effect of PMD, we did not include non-UDG treated samples in this dataset.

SUPPLEMENTARY NOTE 6. MODERN AND ANCIENT REFERENCE DATASET CONSTRUCTION

Krishna R. Veeramah

We assembled SNP data for three modern reference datasets and one ancient reference dataset for comparison to the early medieval samples generated in this study. Datasets were merged using PLINK[66] and custom Python scripts. Except when we focused the analyses on the 10 WGS sequences, diploid genotypes for modern samples were transformed into pseudo-haploid calls by picking one allele at random.

POPRES: The original European Population Reference Sample (POPRES) project[67] dataset consists of 3,192 individuals genotyped at 500,586 loci. Novembre et al.[68] identified a subset of 197,108 SNPs in 1,385 European individuals[68] for demographic analysis. We imputed additional SNPs from the original Novembre et al. dataset using the Michigan Imputation Server[69]. After accounting for potential strand flips there were 165,443 usable SNPs that could be orientated with human reference genome build 37. Phasing was performed using Eagle2[70] with the worldwide sample set from 1000 Genomes Phase 3[71] acting as the reference panel. Following imputation, biallelic imputed SNPs were filtered to be at >=5% minor allele frequency with a r^2 imputation of value of >= 0.9, resulting in a final imputed SNP set of 2,113,389 SNPs. 329,386 SNPs overlapped with the autosomal SNPs included in the 1240K capture and were thus used for downstream statistical analyses in this study.

HellBus: The same imputation procedure was carried out on 512,368 SNPs in 1,582 European and Asian individuals from Hellenthal et al.[72] (henceforth the HellBus dataset). Following

imputation, biallelic SNPs were filtered to be >=5% minor allele frequency with a r^2 imputation of value of >=0.9, resulting in a final imputed SNP set of 4,883,514 SNPs. 779,577 SNPs overlapped with the autosomal SNPs included in the 1240K capture and were thus used for downstream statistical analyses.

1000 Genomes and Simons Genome Diversity Project: All SNPs from the 1240K capture were extracted from whole genome sequences generated from European (CEU, FIN, GBR, IBS and TSI, together named EUR), SouthEast Asian (GIH, PJL, BEB, STU, and ITU, together named SAS), East Asian (CHB, JPT, CHS, CDX and KHV, together named EAS) and Yoruban (YRI) populations that are part of the 1000 Genomes Project[71] as well the 290 publically available whole genomes from the Simon Genome Diversity Project (SGDP)[73], giving us 1240 capture SNP data for a total of 1,883 genomes. 1000 Genomes SNPs were obtained using VCFtools[74] and SGDP SNPs were obtained using cpullout from the cTools package (https://github.com/mengyao/cTools).

Ancient Reference Panel: Genotype data from 435 ancient, prehistorical West Eurasians genotyped at the 1240K capture using haploid calls was obtained from Mathieson et al. (2015)[75] (n=210, 6,500 - 600 BC) (though we note that this includes samples from multiple prior studies), Mathieson et al. (2018)[76] (n=13, 3,400 - 400 BC) and Olade et al.[77] (n=212, 2,500 - 800 BC). For the latter two studies we only utilize individuals dating from the Bronze Age (within which we included the Beaker Culture) or more recent.. In addition, if a number of individuals from a geographic location were available from multiple time points, we included only the samples from the most recent period (for example choosing British Late Bronze Age samples over Early Bronze Age samples in Damgaard et al. [63]). We ignored individuals determined to be related or

outliers in that study. Depending on the analysis, this dataset was merged with one of the three modern reference datasets. A full list of utilized ancient samples is given in Supplementary Data 2.

Each of the three modern datasets provides a certain advantage over the others for downstream population genetic analysis. The POPRES dataset has the greatest sampling density for Europe while having the smallest SNP overlap with the 1240K capture. The HellBus dataset has the greatest sampling density for Eurasia while having only medium SNP overlap with the 1240K capture. The 1000 Genomes+SGDP dataset has essentially maximum 1240K SNP overlap but has the smallest geographic sampling density.

SUPPLEMENTARY NOTE 7. PRINCIPAL COMPONENT ANALYSIS

Carlos Eduardo G. Amorim, Krishna R. Veeramah

All principal components analysis (PCA) was conducted using smartpca[78] unless stated otherwise, with our primary approach involving performing separate PCA analyses via pseudo-haploid calls using some set of reference samples against an ancient sample of interest such that there was no missing data at any SNP. When the number of starting non-missing SNPs for any particular comparison was greater than 100,000 SNPs, PLINK 1.9 was used to filter for possible linkage disequilibrium via the --indep-pairwise argument, using a window size of 50, a step size of 5 and an r^2 value of 0.2. Individual PCAs were then combined using a Procrustes transformation in R using the vegan package as described previously in Veeramah and Hammer[79].

We first performed PCAs using each of the three modern reference datasets against our medieval samples (Supplementary Figures 23-25). Location of the ancient samples was generally very consistent across reference datasets, the only noticeable difference being the HellBus analysis shifting CL31 towards modern populations from the Caucasus (which is also where the 6 Alan samples were found, consisting with their sampling location), but this sample showed high levels of contamination (which we hypothesize is the result of plastic wares produced in China that were utilized in DNA extraction) and thus the results are unreliable. Otherwise, all other samples appear to be overlap with modern European populations.

Previous work has shown that modern European genetic variation is primarily the result of admixture three major ancestry groups, Paleolithic Hunter-Gatherers, Neolithic farmers from Anatolia and Bronze age Steppe Herders[55,80,81]. In order to explore this effect in the context of our

medieval samples. we next merged the Ancient Reference Panel with both the POPRES and HellBus European samples. We then performed Procrustes PCAs using the modern samples as reference as well one genome from Mathieson from each of these three groups with the highest coverage (Loschbour representing Hunter-gatherers, I0707 representing Anatolian farmers and I0231 representing Steppe people), with both the medieval and the remaining ancient samples used as the targets for iterative analysis (Supplementary Figures 65-68). The medieval samples showed no strong evidence of being significantly closer to any particular ancestral population, with modern, medieval and Bronze Age samples overlapping each other.

As genomic sampling from the Migration Period is limited to only a handful of studies beyond this paper[61–63], modern Europeans represent the closest population temporally (~1,500 years, reflecting a difference of about 50-60 generations before our Migration Period samples) with which we can contextualize the ancestry of Szólád and Collegno against a large reference dataset. However, it remains an open question to what extent the distribution of modern European genetic variation today, with its strong signal of isolation-by-distance [68], reflect the patterns of the Migration Period. The next closest period for which there exist a broad continent-wide sampling would be the Bronze Age samples from our ancient reference panel (n=303, range of 3,500 - 500 BC, median = ~2,200 BC, reflecting a difference of about 90-110 generations before our Migration Period samples).

Examining the distribution of Bronze Age samples alongside the modern POPRES samples in the PCA (Supplementary Figures 73-74) reveals a broad concordance between these two time points for the genetic ancestry found in individuals from specific sampling locations in Europe, thought there is a skewed shift for the Bronze Age samples compared to their modern

counterparts (i.e.southeastern Europeans and Hungarians are skewed westerwards and southwards relative to modern samples from the same location and appear closest to modern Italians, Iberian Bronze Age individuals are shifted further to the west, while Bronze Age northern, central and Poles are more clumped together more towards the north). These patterns suggests that the strong signal of IBD observed in Europe today were emerging during the Bronze Age after the initial influx of EEF and SA ancestry. In theory we might therefore also expect this to apply even more so to the intermediate Migration Period as relates to the modern data. Certainly, it seems reasonable to assume that their is general concordance with regard to northern and southern Europe (i.e. a Migration Period sample with ancestry that looks like a northern European today is more likely to look like a northern European than a southern European during the Bronze Age).

Focusing on the Bronze Age populations that best match the sampling location of Szólád and Collegno, we observe that Hungarian Bronze age individuals, as previously noted,[77], are very diverse (Fig 2b, Supplementary Figure 75), with the majority of the samples containing ancestry that is similar to modern southern Europeans, but some that also appear closest to modern northern Europeans, though there is lack of the extreme northern and southern ancestry that we see in Szólád and Collegno (Bronze Age Hungarians are more intermediate).

Though sampling is limited (only four were of sufficient coverage for PCA analysis), Bronze Age Italians possess ancestry that is exclusively found in modern day south and southwest Europe (Fig 2b, Supplementary Figure 76). The three samples from northern Italy do not overlap modern Italy at all, with one looking like a modern day Iberian and two that are orientated towards modern day Sardinian (similar to the Iceman sampled in the Alps) and

another. Only the Bronze Age sample from Sicily is found close to our southern looking individuals from Szólád and Collegno. Bronze Age southeastern Europeans and Hungarians show better overlap with these samples (Supplementary Figure 77). However, what is clear is there is no evidence for ancestry in any Bronze Age southern European (Italy or the southeast) that is associated with modern day northern Europe. Interestingly this is not the case for Bronze Age samples from western Europe, where there are a six samples that show some similarity to Szólád and Collegno samples with northern European ancestry, though five of these were sampled in eastern France rather than Iberia (Supplementary Figure 78).

Bronze Age central, eastern (essentially individuals sampled from modern day Poland) and northern Europeans all overlap the Szólád and Collegno samples with modern northern European-like ancestry (Supplementary Figure 79-81), while northwestern Europeans (of which we focused specifically on the middle and late Bronze Age samples from the UK) are shifted to the west of these samples and correspond strongly with modern UK individuals (Supplementary Figure 82). It is noteworthy that the three Bronze Age individuals from Switzerland are differentiated from those in Northern Italy (being of more modern northern-like ancestry), consistent with the Alps being a major barrier to gene flow through time[82].

Finally, we also performed a PCA analysis on a set of 39 unrelated medieval samples that maximized the amount of SNP overlap amongst samples and did not include any other ancient or modern reference samples. To avoid biases due to SNP calling quality amongst samples, we applied this to haploid calls generated by randomly sampling a single read from each site for each individual. As such the five non-UDG samples were excluded from this analysis. When only analyzing SNPs with no missing data amongst samples, we ended up with a set of ~50-60K

SNPs. CL31 (Supplementary Figure 26), followed by SZ45 (Supplementary Figure 27) were clear outliers. This is not surprising given that CL31 was shown to have high levels of contamination, while ADMIXTURE analysis in the next chapter showed SZ45 to possess a unique ancestry profile. However, analysis of the remaining samples Supplementary Figure 28) essentially recapitulated the main north-south pattern of modern genetic variation along PC1, despite no actual modern reference individuals being used.

SUPPLEMENTARY NOTE 8. MODEL-BASED CLUSTERING ANALYSIS

Carlos Eduardo G. Amorim, Krishna R. Veeramah

All model-based clustering analysis was implemented using ADMIXTURE[83]. We performed two primary analyses, the first to understand how our medieval samples were related to modern samples, and second to explore to what extent their ancestry was shaped by the three major prehistoric groups.

Given that the PCA showed that all our medieval samples contained primarily European genetic variation, we performed a supervised ADMIXTURE analysis, treating samples from the 1000 Genomes FIN, CEU, GBR, IBS and TSI populations as 5 distinct parental groups (i.e. K was set to 5). We also performed the analysis using the same groupings as well all South Asian (SAS), East Asian (EAS) and Yoruba (YRI) being treated as 3 additional parental groups (i.e. K=8). Following the procedure for PCAs above, a separate supervised ADMIXTURE analysis was performed for each medieval sample alongside modern reference samples, with no SNPs analyzed with any missing data and LD filtering for cases with more than 100,000 SNPs. For K=5, each sample was analyzed 10 times with different random seeds and the run with the highest likelihood taken as the final result. However, for K=8 we restricted ourselves to a single run per sample due to the extra computational burden (though we note that the best K=5 results are highly concordant with our K=8 runs).

SGDP European (for K=5) and SGDP Eurasian (for K=8) whole genomes were also included in each of these analyses. These acted as control samples, allowing us to examine to what extent ancestry estimates were varying as result of the different SNP positions potentially used for each medieval sample. We found that while estimates of FIN, IBS and TSI ancestry

were fairly consistent for the SGDP samples (especially when limiting to medieval samples with at least 50,000 callable SNPs, estimates for CEU and GBR showed much more variation (Supplementary Figures 29-33). However, summing these two ancestry estimates together gave much narrower ranges (Supplementary Figure 34), suggesting that ADMIXTURE was having difficulty distinguished CEU and GBR ancestry because of their low genetic divergence. Therefore we report all results combining estimates for CEU and GBR.

Overall, results were concordant with the PCA analysis, with samples primarily of either CEU+GBR ancestry or TSI ancestry corresponding with samples placed near northern and southern European populations in the PCA (Fig 2A). We devised a crude color-based grouping system based on relative amounts of ancestry to demonstrate this concordance: >70% CEU+GBR+FIN = blue, majority CEU+GBR+FIN = cyan, >70% TSI = red, majority TSI = pink, majority TSI+IBS = purple. We note these are not intended to provide population genetic robust groupings, simply to aid visualizing the connection between the model-free PCA and model-based ADMIXTURE plots. It appears that northern samples in Szólád appear to have greater FIN ancestry than those from Collegno in general, though it is a minor component regardless. Estimates of the four European ancestry coefficients were highly robust when increasing K=5 (Supplementary Figures 35-37) to K=8 (Fig 2B,C, Supplementary Figure 38) using additional non-European parental populations, the major noticeable difference being the 20% EAS component for CL31, though results for this sample are unreliable because of high estimated contamination (Supplementary Data 1). In addition, likely due to the absence of an appropriate modern reference population, the six medieval Caucasus samples demonstrated a

significant SAS component (and one sample an EAS component) that was not present when using K=5.

Given that modern European genetic variation demonstrates a strong isolation-by-distance pattern as seen in PCA rather than distinct clusters, we also estimated ancestry coefficients for K=5 for all POPRES European individuals, and overlaid the relative individual values on the corresponding PCA. This would provide better context for interpreting our medieval ADMIXTURE ancestry results (for example, what does having high TSI ancestry actually mean given such a strong continuous isolation-by-distance pattern in Europe?). All five 1000 Genomes populations show the strongest signals closest to POPRES populations from their region of interest (Supplementary Figures 39-43). However their specific distributions that are useful indicators of their meaning. Firstly, FIN ancestry is fairly modest, even in North East Europe, with only the Finnish POPRES individual demonstrating 100% ancestry, reflecting this populations high levels of genetic drift/low N_e and historic inbreeding. Northern Western and Northern/Central Europe is dominated by CEU and GBR ancestry, though there is less smoothness to this relationship compared to IBS and TSI. However, while IBS ancestry is highly localized to the Iberian peninsula, TSI ancestry is highest in both southern Italy and parts of South East Europe.

In order to examine whether any biases were introduced by fixing reference samples, we also performed an unsupervised ADMIXTURE analysis using the same 1000 Genomes populations as utilized above for K=8. In this case, rather than iterative analyses, we performed a single analysis using only the set of unrelated Szólád and Collegno samples.

A five-fold cross-validation analysis identified K=6 as the most appropriate number of ancestral components (Supplementary Figure 44). At K=4, there is clear clustering amongst the European, South Asian, East Asian and Yoruba (Supplementary Figure 45), with all Szólád and Collegno demonstrating almost 100% European ancestry (blue cluster) (Supplementary Figure 46). At K=6 two components emerge amongst the European populations, one that is predominant in FIN (blue), and another that is predominant in TSI and to a lesser extent IBS (red). CEU and GBR are intermediate for both these components. At K=8 a component emerges that is predominantly in FIN (light blue), another that is the majority type in CEU and GBS (blue) and one that is the major type in TSI and to a less extent IBS. At this point the Szólád and Collegno samples show variation in this component which corresponds strongly with whether they have predominant CEU+GBR versus TSI ancestry in the supervised analysis. Therefore, this suggests that our supervised analysis is not introducing any major biases into our results. Further increasing K did not give meaningful clusters for our purposes, instead tending to add components to the South Asian populations.

Our use of modern samples as surrogates for ancestral populations for the medieval samples was guided by the fact that the latter are temporally quite close (1,500 years, 50-60 generations), and thus we hypothesized that fifth to sixth century European population structure would likely be fairly well approximated by modern European population structure. However, it also might be of interest to examine these patterns within the context of the three major European prehistorical ancestry groups that have previously been identified [80]. Therefore we performed a supervised ADMIXTURE analysis with K=3, with a hunter-gatherer (WHG) ancestral population consisting of northern and western hunter-gatherers (n=9), an EEF ancestral

population consisting of all Anatolian Neolithic farmers (n=24) and an SA ancestral population consisting of samples from the Yamnaya culture (n=15). Because of the limited SNP data in the ancient reference samples because of uneven coverage, we did not perform individual analysis for each medieval individual. We instead again analyzed the set of unrelated individuals simultaneously with the remaining ancient reference samples that had a genotyping rate >0.7 (though only 19 Bronze Age samples met this criteria, we found using samples with lower call rates than this threshold of 0.7 introduced biases in ADMIXTURE that were coverage-dependent) as well as either POPRES or HellBus European samples. LD filtering was again performed prior to analysis. Ten runs with random seeds were performed and the run with the highest likelihood used to in the final analysis. There are two sets of results for the medieval samples, one for the POPRES samples and one for the HellBus samples. We report the latter due to the increased number of SNPs overlapping with the 1240K but the former was very similar.

Almost every individual contains ancestry from each of the three ancestral groups (the exception being CL30 which lacks any WHG ancestry) (Supplementary Figure 69, bottom row). However, the relative amounts were highly variable amongst individuals. In general, individuals that we previously identified as having high CEU+GBR ancestry had higher WHG and SA ancestry, while those who were classified as having high TSI ancestry had higher EEF ancestry. To better visualize these ancestry components we performed PCA using the prcomp function in R based on the three ADMIXTURE coefficients (not the actual SNP data) for all unrelated medieval samples, all POPRES individuals and all European HellBus individuals. The resulting plot (Supplementary Figure 70) large recapitulates the PCA analysis based on SNP data, with regard to both modern European population structure and the relative placement of the medieval

samples. However, this analysis also reveals to what extent this pattern is driven by the three prehistorical ancestry groups. Modern southern European ancestry is primarily driven by Neolithic farming ancestry, while northwestern and northeastern Europe have increased hunter-gatherer and steppe ancestry respectively.

An unsupervised analysis of the same data suggested K=2 had the least cross-validation error (Supplementary Figure 71, Supplementary Figure 72 top row). However, when K=3 was set, clear clusters were formed that corresponded strongly with the original WHG, SA and EEF groups under the supervised framework (Supplementary Figure 72 middle and bottom row). Once again, the ancestry coefficients for the medieval samples were highly similar between the supervised and unsupervised analysis (Supplementary Figure 69), suggesting no major biases when utilizing the former approach.

SUPPLEMENTARY NOTE 9. CHROMOSOME-Y HAPLOGROUP INFERENCE

Paolo Francalacci

9.1 METHODS

Sequencing data were provided for the Y chromosome of 39 individuals and the respective VCF files reported information for 32,126 biallelic Single Nucleotide Polymorphisms (SNPs). The VCF data were imported in an Excel spreadsheet, where, for each sample and for each physical position (in build37), the number 0 refers to a nucleotide identical to the reference, the number 1 the alternative and a period (.) indicates a missing nucleotide call. Two individuals (SZ6 and SZ20) showed poor quality calls (both 0 and 1) and were excluded from further analysis. The remaining 37 individuals showed an average of 15,913.5 calls out of the 32,126 positions (52.2%). However, the ratio between positive vs. no calls was not homogeneous in the database, since the 9 individuals for which we had whole genome sequences yielded a positive call for most of the 32,126 SNPs, with only 1.4% of no calls, while 28 individuals for which we had SNP capture data showed an average of 32.2% of positive calls.

Among the 32,126 SNPs analysed, 27,904 were not polymorphic across the samples and were identical to the reference in all the samples showing a positive call, 2,049 were singleton (presenting the alternative nucleotide in only one individual) and 2,173 were polymorphic sites that occurred in at least two individuals.

Polymorphic nucleotide positions and respective genotypes were listed and the variants were assigned according to their association to known haplogroups (groups of haplotypes sharing one or more common ancestral SNPs). The phylogenetic position of each SNPs was established according to its occurrence in public database or in published literature[84–88].

The reference genome is a chimera of at least two individuals and contains a major portion belonging to haplogroup R, with about 1Mb (from 14.3Mb to 15.3Mb) belonging to haplogroup G. To overcome this confounding factor, we referred to the ancestral allelic status, inferred by parsimony, to describe each SNP, rather than to the allele reported in the reference.

A total of 1,087 variants univocally associated with a known haplogroup, sub-haplogroup or phylogenetically related haplogroups were considered informative. The lack of base calls due to the absence of reads at a position in a particular sample was resolved either as an ancestral or derived allele by a hierarchical inferential method according to the phylogenetic context based on a cladistic approach. In fact, the absence of recombination and the low recurrence and reversion rates of the Male Specific portion of the Y chromosome (MSY) implies the sequential accumulation of mutations over time, so that the presence of a derivate recent (apomorphic) allele allows the attribution to the derivate status to all the ancestral (plesiomorphic) alleles present upstream in the haplogroup, regardless of being directly observed or not. Hence, the allelic status of a specific SNPs is not always experimentally determined for each sequenced individual, but we report an average of 53.7% directly detected and 46.3% inferred SNPs for each sample (closely reflecting the analogous positive/missing call ratio for the total 32,126 SNPs).

A total of 1,066 polymorphic sites that were discovered in multiple individuals but could not be unequivocally assigned to any of the haplogroups were discarded.

In addition to these 1,087 informative SNPs detected in at least two individuals, there were 2,049 detected in only one individual (singletons). Since in this case it is not possible to apply a phylogenetic criterion, only 226 SNPs already observed in other databases and coherent

with the previous haplogroup assignment made with the informative SNPs were considered. Considering both informative SNPs and known singletons, the database yielded a total of 1,313 variants (Supplementary Data 3) which allowed the classification of the 38 individuals according to the nomenclature defined by the Y-Chromosome Consortium[89] and further detailed in the site of the ISOGG[88] (Supplementary Table 2).

The 1,313 phylogenetically informative SNPs were used to build a parsimony-based phylogenetic tree using the Phylip v3.69 package[90], using the Pars application (Discrete character parsimony algorithm). FigTree v1.4.2 software was used to display the generated tree[91] (Supplementary Figure 47).

The Collegno (COL) and Szólád (SZO) samples were compared with sequences coming from 5 European (Tuscans=TSI; GBR=Britons; Iberians=IBS; Finnish=FIN; Sardinians=SAR) and one American of European origin (Central European from Utah = CEU) populations from published databases[84,85,87]. For sake of clarity, only the 428 individuals belonging to the haplotypes identified in the ancient samples were included in the analysis. The phylogenetic analysis and the resulting tree were carried out as above (Supplementary Figure 48).

A bar plot showing the relative frequencies for all the haplogroup and relevant sub-haplogroup observed in the above-mentioned populations was drawn using Excel (Supplementary Figure 49).

9.2 RESULTS

The analysis of the SNPs detected by sequencing the ancient DNA extracted from 37 samples from Szólád (N=21) and Collegno (N=16) (Tab. 1) allowed the attribution to their respective

haplogroup and sub-haplogroup (Supplementary Figure 48). In spite of the presence of missing data and false positive results for some polymorphic sites, as expected for sequences derived from aDNA, about a half of the polymorphic sites (1,087 out of 2,173) can be used for constructing a robust phylogenetic tree encompassing the 37 samples (Supplementary Figure 47). In fact, because of the features of the non-recombining portion of the Y chromosome, the lack of base calls due to the absence of reads at a position in a particular sample can be resolved either as an ancestral or derived allele by a hierarchical inferential approach (see Methods in this supplementary section above), which allows the correct attribution of the allelic status of a polymorphic site to all the individuals belonging to a clade (even in the case of a missing call for most of the samples) if they show subsequent derivate SNPs. It should be noted that such a phylogenetically based inference strategy is inherently less accurate for the rarest lineages, where the chance is higher of missing a polymorphic site in all the individuals. As a consequence, all the haplogroups encompassing one or two individuals show a shorter branch length. Moreover, the individuals with a poor yield in terms of positive calls tends to root in the basal position of their respective haplogroup or sub-haplogroup (Supplementary Figure 48). This is due to the higher probability of having a missing call for all the downstream position and, consequently, they cannot be further assigned to any subclade of the main haplogroups.

We excluded sample SZ1 from further analyses because this sample was found to belong to a different era of occupation of Szólád.

The samples do not appear to be paternally related in most of the cases, as the shared haplotypes are generally rooted in the basal position of each haplogroup, suggesting it could be due to the incomplete detection of derived alleles for individuals with poor sequencing coverage,

as mentioned above, and not necessarily to biological kinship (though we note haplogroup assignment makes sense in light of kinship inferred from autosomal loci.

While most of the haplogroups observed in Szólád and Collegno are also present in Africa, the Middle East, Central Asia and the Indian subcontinent, the sub-haplogroups shows a typical European distribution (Supplementary Figure 49), although with different frequencies at a subregional level (see the review Francalacci and Sanna[92] and references therein). The haplogroups detected in the samples show a prevalence of R1b (55.3%), which is the most common sub-haplogroup in western Europe, with a peak in the Iberian Peninsula and in the British islands and a west-east gradient in central Europe[93]. A consistent percentage of haplotypes belongs to the I haplogroup (26.4%), both in the I1a and, more abundantly, in I2a2 sub-haplogroups. They are particularly frequent in the northern Balkans with a westward gradient in central and western Europe, with some lineages belonging to I2a2a1b particularly common in the Germanic region[94]. Rarer lineages included R1a, which is possibly related to the Indo-European diffusion and shows its higher occurrence in eastern Europe [93], and G2a for CL31, which probably has its origin is in the Caucasus and followed the Danubian route of the spread of agriculture in central Europe and in Italy until the islands of Corsica and Sardinia[95,96]. Two sub-haplogroups, E1b1 and T1a (both at 5.3%) show a Mediterranean distribution with a prevalence in southern Europe, in particular in the Iberian and Italian peninsulas[93,97].

The comparison of Szólád and Collegno, considered as a whole, with other European populations (Supplementary Figure 49) shows a marked resemblance with the CEU (Central Europeans) because a similar ratio between R1a/I haplogroups, with a certain influence of lineages present in the TSI (Tuscans), indicating a major Central/Northern European component,

with a noticeable contribution from Southern European populations (in agreement with the results for the autosomes). However, even with the limitation due to the low sample size, the Central European/Balkan influence is more apparent in the Szólád sample, which shows a relevant presence of the lineages belonging to the haplogroup I, than in the Collegno sample, with a prevalence of Southern/Western European haplotypes.

SUPPLEMENTARY NOTE 10. POPULATION ASSIGNMENT ANALYSIS

Krishna R. Veeramah

In order to obtain a more precise estimate of the modern population most closely resembling our ancient samples, we applied the following likelihood framework to our medieval ancient sample data using either the POPRES or HellBus datasets as reference populations in what we call a Population Assignment Analysis (PAA)[62].

For every reference population, *k*, with at least *n*/2 individuals we estimated for every SNP, *i*, the allele frequency of an arbitrary allele (q_{ik}) for a randomly drawn set of *n* chromosomes (so sample sizes were equal across populations). Then, for each ancient sample we determined the most likely population of origin by estimating the log-likelihood of observing the pseudo-haploid call, D_i, given a particular reference population for each SNP position, which is simply the log of q_{ik} for the observed allele, and summing across loci. In order to account for a reference population being fixed for the allele not observed in a particular ancient sample (which may happen because of either the low sample size of the reference population or a sequencing error for the ancient sample), we allowed an 0.1% error rate, *e*, such that the log likelihood for each SNP used was:

$$lnLL(k \mid D_i) = ln[q_{ik}\,(1\text{-e})) + ((1\text{-}q_{ik})\,e)] \quad ,$$

where q_{ik} is the frequency of the observed allele, D_i. However, the results were robust to different choices of *e* (both smaller and larger).

In order to obtain an estimate of uncertainty in our most likely reference population and take into account correlation amongst neighbouring SNPs, we performed 100 bootstrap iterations, where for each iteration we resampled with replacement 5 Mb non-overlapping

windows of SNPs from across the genome. For each bootstrap iteration we noted the reference population with the highest likelihood using the above expression and scored the total number of times each population obtained the highest log likelihood across the 100 iterations.

We performed this assignment both at the level of countries which could be assigned a latitude and longitude and region (see Supplementary Figure 50 for European region definitions based on grouping modern countries). When performing the analysis at the level of regions, we applied two different cut offs for *n.* The reason for this was that increasing *n* to a larger number should in theory improve resolution, but because of the limitations of our sampling database, this also leads to not having enough samples to represent Northern Europe (NE).

The most likely reference population for POPRES and HellBus samples is shown in Supplementary Data 4. In general there is high concordance between population assignments, PCA position and ancestry estimation. Notably, southern ancestry individuals from Collegno tended to be assigned to Southern Europe (SE, essentially Italy) with high probability, while those from Szólád were more diverse, with greater weight towards South East Europe (SEE) assignments. The average SE/SEE probability for the four individuals with >70% TSI ancestry in Collegno (excluding CL31) using the POPRES and HellBus datasets (highest *n* cutoff) was 1.0/0.0 and 0.80/0.20 respectively. For the five individuals with >70% TSI ancestry in Szólád the pattern was reversed, with values of 0.31/0.43 and 0.36/0.49 (i.e. a higher SEE probability overall).

For the 17 individuals assigned to the NE region using the lower cutoff for *n* for the POPRES dataset, 9 and 8 were assigned to CE and NWE respectively at the higher cutoff. For the 13 individuals assigned to the NE region using the lower cutoff for *n* for the HellBus dataset,

6, 6 and 1 were assigned to CE, NWE and WE respectively at the higher cutoff. When comparing the two major kindred (see Supplementary Note 13), the POPRES data assigned 5 of the 10 individuals to NWE for Kindred CL2, but only 1 individual for Kindred SZ1 (SZ15, who is only peripherally connected to the family via SZ6). This appears to correspond with the slight shift of Kindred CL1 to NWE in the PCA and SPA analysis, and the increased FIN ancestry in Kindred SZ1. This effect is not as apparent using the HellBus dataset, but this reference population is not as well represented for NWE individuals (Supplementary Data 4).

SUPPLEMENTARY NOTE 11. D-STATISTIC ANALYSIS

Krishna R. Veeramah

11.1. Testing Szólád and Collegno against modern reference samples

In order to formally test whether any of our ancient medieval samples statistically share the same underlying genomic ancestry, we performed a series of pairwise D-statistic analyses [98] on our set of unrelated samples from Szólád and Collegno, as well as on the seven Anglo-Saxon samples. To ensure no downstream biases were introduced due to differences in genotype calling quality in our ancient samples, we used haploid calls based on sampling a random read, and only for those samples that undergone some form of UDG treatment.

Our framework involved comparing each possible pair of ancient samples (anc1 and anc2) to a set of modern reference populations (ref) to determine whether they formed a clade with significant statistical support. For each pair we performed three tests of the form D_A(anc1, anc2, ref, outgroup), D_B(anc1, ref, anc2, outgroup) and D_C(anc2, ref, anc1, outgroup). Using a significant Z-score cutoff of 3, if D_A was not significant and either (or both) D_B and D_C were significant, we considered this pair to form a clade compared to the reference population. If none of the three tests was significant we considered that this pair may form a clade given the resolutions of our data (i.e. we cannot reject this hypothesis). If D_A was significant we did not consider the pair to form a clade regardless of D_B and D_C. Given this analysis involves almost 500,000 individual D tests, it is likely a Z-score cutoff of |3| will result in a number of false positives, but we find that general patterns, such as clusters in agreement with other analyses in this paper, can still be observed via this criteria.

We applied this framework to three reference datasets. In the first (henceforth the SGDP-test), we compared each pair to every Eurasian genome (n=166) in the SGDP, with the calls being pseudo-haploid. B_Yoruba-3 were both used as outgroups (again, pseudo-haploid). These reference comparisons maximized the number of SNPs available for any pairwise comparison while keeping all samples sizes equal (i.e. comparison involved four haploid genomes). We also repeated this analysis using S_Khomani_San-1 as the outgroup, but results were highly similar (Z-score r^2 of 0.993). Of the instances of the D-statistic test where at least using one outgroup was significant (n=199,006), 6% (n=12,269) of cases were not significant for the other outgroup, and the directionality of the Z-score was always in the same direction

In the second (henceforth HellBus-region), we used the HellBus samples grouped by European region, as well as Caucasus, Middle East, Central Asia and South Asia, as the reference populations. We also added the five East Asian populations from the 1000 Genomes project pooled together to this comparisons, for a total of n=13 comparisons. Each reference population was downsampled such the allele frequencies at each SNP were based on 36 chromosomes (as in the PAA analysis). The 1000 Genomes Yoruba population was used as an outgroup. These reference comparisons maximized the reference sample size for each comparison, which should increase resolution of the test, even if the usable SNP number was reduced.

In the third (henceforth HellBus-country, we used the HellBus samples grouped by country, as well as the five East Asian populations from the 1000 Genomes project (this time not pooled), as the reference populations, for a total of n=55 comparisons. Each reference population

was downsampled such the allele frequencies at each SNP were based on 20 chromosomes (as in the PAA analysis). The 1000 Genomes Yoruba population was again used as an outgroup.

11.2. Results

The analysis described above involved almost 500,000 individual test. In order to summarize our results, we tally for each pair of ancient samples the proportion of comparisons to reference populations where they (a) form a significant clade or (b) where a clade cannot be rejected. When the former proportion is 100%, it suggests that this pair of ancient samples is closer to each other than any available modern reference population. When the latter proportion is 100%, we cannot reject this scenario. Plotting these results in a pairwise matrix format reveals general consistency across the different reference populations, though the resolution varies with regard to evidence for statistical clades (Supplementary Figures 51-56). When samples are ordered based on their ancestry assignment in Figure 1a, there is clearly greater similarity for pairs of samples placed in the same genetic ancestry category. In almost all cases we cannot reject that ancient pairs compared within the central/northern ancestry component and separately within the southern ancestry group form a clade. Similarly we cannot reject Anglo-Saxon period samples forming clades with the predominantly central/northern ancestry samples. Central/northern ancestry pairs appear to more often form significant clades than southern ancestry pairs, suggesting the similarity is stronger amongst former group, and that the southern group could potentially be formed from multiple subgroups.

There are no instances where a pair of samples shows 100% of significant clades compared to reference populations when using the SGDP data (Supplementary Figure 52), but

there 14 and 8 when using the HellBus region and HellBus country reference sets (Supplementary Figure 54, Supplementary Figure 56, Supplementary Table 3), the latter of which are all a subset of the former. They predominantly involve pairs of populations with significant central/northern ancestry, and notably four of these include pairwise comparison between a sample from Szólád and Collegno, particularly between members of the large kindred CL1 (individuals CL87 and CL151) and SZ10, SZ13 (for the former) and SZ30 and SZ38 (for the latter) (Supplementary Table 3).

Focusing on the HellBus datasets, when examining just the pairs of ancient samples from the central/northern group, for the cases where clades did not have a significant statistical support but could not be rejected, present-day northern and central European reference populations and regions dominate (Supplementary Figure 57-58). For the pairs of ancient samples within the southern ancestry group, southern European populations are more prominent as modern reference groups that cannot be distinguished from the ancient pairs, but the pattern is not as clear cut (Supplementary Figure 59-60), as it is for the central/northern group. Non-European reference populations and regions play almost no role in clades involving pairs of northern ancestry (i.e. the ancient samples are almost all significantly a clade when comparing to these reference populations), though the Caucasus sometimes cannot be distinguished from some pairs of samples of southern ancestry.

11.3. Testing Szólád and Collegno against Bronze Age reference samples.

In order to formally examine to what extent our medieval samples were more similar to Bronze Age samples sampled from the same region (Hungary, HUb) and Italy, ITb) versus other European regions, we performed a series of D-statistic analyses of the form

D(Other_Bronze_Age, HUb/ITn, medieval sample, outgroup). We applied this test to each of our unrelated sample as above, this time using the best set of pseudo-haploid calls for the 1240K capture with the other regions tested being Bronze Age northern Europe (n=12, Nb), Bronze Age northwestern Europe (n=31, NWb), Bronze Age eastern Europe, (n=15, Eb), Bronze Age central Europe (n=141, Cb), Bronze Age western Europe (n=40, Wb) and Bronze Age southeastern Europe (n=13, SEb). We applied this test using both the YRI and San as outgroups but the results were very similar; therefore we only report the former here.

Fairly consistently, individuals with greater CEU+GBR ancestry from both Szólád and Collegno (as well as the 4-7th century UK individuals) were more related to NWb, Cb, Eb than Bronze Age Hungarians or Italians, with the signal flipping direction for samples with more TSI ancestry (Supplementary Figure 83), consistent with what might be expected given the PCA results. Similarly, individuals with high TSI ancestry generally (but not always) demonstrated greater similarity with Bronze Age Italians (Supplementary Figure 84) and SEb than Hungarians.

Perhaps unexpectedly for Nb, while the overall relative D-statistics were of a similar distribution to those for NWb, Cb and Eb (they appear somewhat left-shifted), they were rarely significant with a $|Z| > 3$ in the expected direction for individuals with high CEU+GBR ancestry, and in one case, highly significant in the other direction (for example CL87). Given the clear overlap of the Nb samples and those with greatest CEU+GBR ancestry from both Szólád and Collegno in the PCA, whether this effect is real or is some other artifact is unclear. However, Nb does appear closer to the 4-7th UK genomes, perhaps pointing to a subtle but real effect. Indeed, D-statistics of the form D(Cb/NWb/Eb, HUb, Nb, YRI) are all significantly positive, suggesting HUb share some ancestry with Cb/NWb/Eb that is lacking in Nb, despite tests of the form

D(Cb/NWb/Eb, Nb, HUb, YRI) also being significantly positive. However, we note that the Nb samples are the only set involving completely non-UDG treated ancient DNA, and thus the shifts may simply reflect slight differences in error rates due to post-mortem damage.

In general our Migration Period individuals largely cannot be distinguished when comparing Wb to HUb or SEb to ITb, though individuals with high TSI ancestry do tend to be more similar to Wb than HUb (7 individual tests with $|Z| > 3$).

SUPPLEMENTARY NOTE 12. RARE VARIANT ANALYSIS

Krishna R. Veeramah

While the majority of our analysis involves the use of methods that examine specific SNPs in the 1240K capture, our medium-high coverage WGS of 10 samples from Szólád (even though one would later be found to have origins from an earlier period) allowed us to make inferences using so called rare-variants, as previously applied to the Anglo-Saxon-era samples from the UK by Schiffels et al.[61].

As in Schiffels et al.[61] we assembled allele frequencies for four 1000 Genomes European populations (GBR, FIN,IBS and TSI), as well 200 unrelated Dutch (NED) chromosomes[99] (randomly downsampled from 998) and 40 unrelated Danish (DAN) chromosomes[100] for every autosomal position on human reference genome build GR37, restricting to mappable regions determined by the 1000 genomes Phase 3 mask. We then called both pseudo-haploid and diploid genotypes as described in Supplementary Note 5 for our medieval whole genomes in the same regions, restricting to sites with 7x depth or greater. Any analysis involving a particular medieval sample was restricted to these regions across all samples. Alleles were determined as ancestral or derived using the 1000 Genomes ancestral sequence.

In an initial analysis we identified a set of sites with a derived allele count of <10 across both NED and TSI. Then for each of the nine medieval individuals we scored whether they possessed the derived allele at theses sites using the pseudo-haploid calls. For each total derived count bin, we then estimated the ratio of number of sites shared with NED and TSI. We then plotted this following Schiffels et al.[61], with error estimated using standard error propagation (Supplementary Figure 61). Samples were color coded based on their relative NED (blue) versus

TSI (red) ancestry at singleton sites. Despite being based on rare variants rather than SNPs with more common allele frequencies, the relative sharing of these 9 WGS highly concordant with their position in a PCA of modern POPRES samples using 1240K capture SNPs (Supplementary Figure 62), demonstrating that these sixth century samples are closely related to modern populations (i.e. a medieval sample with northern ancestry shares more private variants with modern northern European samples than a modern southern European would do).

In order to more formally model these relationships we analyzed our nine medieval WGS using the software rarecoal. We first re-estimated parameters for the population genetic model for the six modern reference populations using the Monte Carlo Markov Chain method while modelling rare variants with a total count of 4 or less across all populations. Our estimates were generally similar to that of Schiffels et al.[61], though we estimated a deeper split time for TSI and IBS, lower ancestral N_e and higher modern day N_e. Schiffels et al.[61] previously noted these parameters were problematic because of possible post-divergence gene flow (Supplementary Table 4). For consistency, we performed our analysis using the original Schiffels et al.[61] model.

We then attempted to place each of the nine WGS on to one of branches of this model, ignoring any derived site that was only found in the ancient sample to guard against false positives (Supplementary Figure 63). The three most extreme northern samples (SZ2, SZ4 and SZ11) were placed on either the Danish or Dutch branches (with high relative likelihood for both). SZ3, the fourth most northern sample was found close to the root of GBR, DAN, NED, and FIN branch point, while SZ36 and SZ43 were placed closest to the root of the tree separating northern and southern Europe, presumably because of their increased southern ancestry. SZ5 and SZ45 had a likelihood space across most of the tips of the branches, perhaps as a result of their

intermediate ancestry. Using diploid calls (still ignoring singletons) appeared to improve our ability to place samples on the southern portion of the tree (Supplementary Figure 64), with SZ36 placed on the shallow TSI branch, and the likelihood distribution being much sharper across the tree space, while SZ43 is placed at a root node, but the next highest likelihood space is found along the TSI/IBS branch. However, we must be cautious in interpreting these results due to the variable WGS coverage that may result in overconfident placement due to unaccounted diploid calling error.

SUPPLEMENTARY NOTE 13. BIOLOGICAL KINSHIP INFERENCE

Carlos Eduardo G. Amorim, Krishna R. Veeramah

To infer biological kinship between each pair of individuals, we used lcMLkin[101], a software implemented in C++ and with modifications implemented in Python. The method implemented in this software considers genotype likelihoods instead of a single best genotype for each individual to infer biological kinship, and so it is optimal for use with ancient DNA (aDNA) and other potentially low coverage DNA samples (e.g. in forensics), for which an excess of false homozygotes is usually an artifact. lcMLkin can infer biological kinship down to 5th degree relatives (e.g. second degree cousins), even when coverage is as low as 2x[101]. The software uses population frequencies to estimate the probability of identity-by-descent (IBD) given the observed genotypes likelihoods, and outputs the k probabilities that two diploid individuals share zero (k_0), one (k_1) or two (k_2) alleles at a given loci IBD, such that $k_0 + k_1 + k_2 = 1$. Individual SNP log likelihoods are summed for a given pair of individuals. If the three k parameters can be estimated, one can infer the degree of relationship between each pair of individuals and calculate the kinship coefficient φ. See Table 1 in Weir et al.[102] for a list of expected values for k_0, k_1, and k_2, for different degrees of relationship.

Population allele frequencies will determine the probability of IBD given identity-by-state (IBS). lcMLkin implements two methods: (a) one that considers allele frequencies in an external reference population and (b) another that uses the target samples themselves to estimate the frequencies. Though we have further developed lcMLkin incorporate drift based on a specific F_{ST} via a Balding-Nichols model[103], we find that kinship inference will generally be robust for inferring major degrees of relatedness when the assumed population

allele frequencies are diverged from the true frequencies for realistic levels of genetic drift occurring within European populations such as in this study[101].

We implemented four types of runs in lcMLkin using different samples to estimate the population allele frequencies: (i) CEU, (ii) TSI or (iii) merged CEU and TSI as reference populations (since these can account for the major genetic component in our medieval samples according to Fig 2A), and (iv) without a reference population set. Allele frequencies for the latter case were estimated without individuals identified as relatives based on a preliminary run.

The values for the kinship coefficient ϕ between all possible pairs (both within and across cemeteries) using no reference set (run iv) were very similar to the other three types of runs (Supplementary Figure 85). r^2 for ϕ in run iv versus runs i, ii, and iii are 0.81, 0.79, and 0.81 respectively, with p-value < 1 x 10e-15 in all cases. This suggests that kinship inference was be robust to our assumptions regarding the population allele frequencies for our data (indeed, all major relationships identified in this study were observable using all four runs).

The value of k_0 describes the probability of two individuals not sharing an allele in a given site by IBD. If this value is next to 1, then individuals are not related. Based on the value of k_0 associated with the kinship coefficient ϕ, we arbitrarily subset the dataset in two groups: one with individuals that we could discard as relatives (i.e. those that had k_0 close to 1 and ϕ close to 0), regardless of them being from the same cemetery or not; and another with potential relatives. Since we have no reason to believe that individuals coming from distinct cemeteries are actual relatives, we used the distribution of ϕ in that group (which is centered around zero) as a benchmark for ϕ values among non-related individuals. The distribution of ϕ for putative non-relatives coming from the same cemetery closely matched that for different cemeteries

(Supplementary Figure 86), suggesting this former group is mainly composed by unrelated individuals as well. The distribution of ϕ for the putative relatives is broader, with a peak at zero (for individuals that are potentially unrelated or not closely related) and other smaller ones at 0.2 and 0.45 (Supplementary Figure 86). The density away from zero should largely reflect ϕ for individuals that are first, second or third degree relatives. We focused on the subset of potentially related individuals (see Supplementary Data 5 for kinship coefficients for each of these pairs) to infer pedigree structures, as follows.

We started by identifying non-ambiguous relationships (e.g. parent and child) and then extended the family tree to the more ambiguous cases (e.g. k_0 = 0.5, k_1 = 0.5, and k_2 = 0 may be of the type avuncular, first cousins and grandparent-grandchild). When a parent-child relationship was inferred, we defined the parent as the individual who was the oldest at the moment of death. The same is valid for grandparent-grandchild. In large pedigrees (e.g. Kindreds SZ1, CL1, and CL2), the relationship between one pair of individuals was always corroborated by the relationship of each one of the individuals in this pair to a third individual (except for individual SZ6, see below). For example, CL146 and CL145 are brothers and both can be assigned as nephews of CL93 (Fig 3B). The various possibilities in the ambiguous cases were examined one by one, by two of the investigators in this work until consensus was reached.

We next ran PRIMUS[104] on the whole dataset (potential relatives and non-relatives, considering different reference sets for inferring the population allele frequencies) to confirm the "manually" inferred pedigree structure and identify additional potential cryptic relationships. By doing so, we confirmed all initially inferred pedigrees. We also confirmed the distant but significant relationship between members for two nuclear families: one composed by individuals

CL145, CL145, CL93, and CL92; and the other by individuals CL97, CL87, CL83, CL84, and CL102. The relationship between these nuclear families is unclear, but it excludes individual CL151 and seems to be more distant than that of third degree. We indicate this more cryptic relationship by dashed lines connecting both pedigrees (Fig 3B). We note that inbreeding in CL97 is suggested by the high value of k_1 but 0 for k_2 in relation to his likely nephews CL83 and CL84. i.e. there are more alleles with IBD=1 than would be expected for a second degree relative, but less than would be expect for a parent offspring relationship. If CL97 was inbred we would expect this to occur because alleles transmitted to the nephews would already have IBD within CL97 (i.e. Jacquard identity modes S3 or S5[105]).

We also detected biological relationship between individual SZ6 and individuals SZ15 and SZ19. The degree to which these individuals are related is unclear, but this signal is not seen between SZ15 or SZ19 and any other individuals in that kindred (Kindred SZ1), not even SZ8 and SZ14, to which SZ6 is most related (almost to the degree of siblings but k_2 is too low). We confirmed the relationship and general k values between SZ6 and the other individuals using both pairwise PCA and a modified version of lcMLkin that took into account admixture based on the model from Moltke and Albrechtsen[106] and incorporating ancestry estimates from the ADMIXTURE analysis above (note, we could not apply this method to all pairs because it is computationally intensive). No standard biological relationship could fit the estimated kinship coefficients, which may could be a consequence of SZ6 being of low coverage (0.048x) or some degree of inbreeding in this individual's history. Therefore we connected SZ6, SZ15 and SZ19 to Kindred SZ1 via dashed lines to indicate our uncertainty (Fig 3B).

This approach allowed us to identify four kindred in Szólád and three in Collegno (Fig 1B-C). We established the most likely pedigree for each one of the three largest kindreds (Fig 3). We have also plotted the distribution of these three kindred in a PCA against modern reference samples (Supplementary Figure 87). For the remaining four small kindreds, possible pedigree structures were many and because of the small number of individuals, we were not able to cross-validate exact relationships and choose a single best structure. The following relationships are likely: SZ41/SZ42=first cousins, CL110/CL121=first cousins, SZ18/SZ23=half siblings. The remaining five relationships are unclear.

SUPPLEMENTARY NOTE 14. SPATIAL ANCESTRY ANALYSIS

Krishna R. Veeramah

Spatial Ancestry Analysis (SPA)[107] is a model-based framework that, amongst other things, allows the inference of the relative geographic location of an individual's ancestors for an arbitrary number of generations in the past (though typically this will be applied to infer this location for an individual's two parents. This can allow the identification of individuals with mixed ancestry. It does this by fitting genotype data to a spatial model of allele gradients (determined by a logistic function) previously inferred using a reference set of unadmixed individuals.

This logistic function is parameterized for each SNP j like so:

$$f_j(x) = \frac{1}{exp^{(-a_j^T x - b_j)} + 1}$$

where a and b are function coefficients and x the geographic location being considered.

When inferring the parental locations of an admixed individuals we must consider two such locations, x and y and thus two functions, $p_j = f_j(x)$ and $m_j = f_j(y)$. The original probability distribution for this function when considering diploid genotype calls is as so:

$$P(g_j = 2 | x, y) = p_j m_j$$

$$P(g_j = 1 | x, y) = p_j(1 - m_j) + m_j(1 - p_j)$$

$$P(g_j = 0 | x, y) = (1 - p_j)(1 - m_j)$$

where g_j is the observed number of minor alleles. Inference can then be made by maximizing the likelihood function across all SNPs.

$$L(g;x;y) = \sum_{j} ln\, P(g_j | x, y)$$

However, in some cases full diploid genotypes are not possible due to low coverage (for example due to the use of ancient DNA as in this study). In such case only one allele of a true diploid genotype will be represented in the observed genotype. In this case we must adjust the probability distribution (note, that $P(g_j = 2 \mid x, y)$ is no longer possible):

$$P(g_j = 1 | x, y) = \frac{p_j(1-m_j)}{2} + \frac{m_j(1-p_j)}{2} + p_j m_j = \frac{p_j+m_j}{2}$$

$$P(g_j = 0 | x, y) = \frac{p_j(1-m_j)}{2} + \frac{m_j(1-p_j)}{2} + (1-p_j)(1-m_j) = \frac{2-p_j-m_j}{2}$$

In addition, if the ancestry of one of the parents of a child of mixed ancestry is known (i.e. x), it should be possible to identify the the ancestry of the other parent by searching for the maximum likelihood of y over a fairly simple space (for example a two dimensional space involving latitude-like and longitude-like coordinates. Assuming the European genetic variation today is approximately structured similarly to the fifth to sixth centuries, we aimed to exploit this to identify the approximate geographic ancestry of missing parents in our various pedigrees from Szólád and Collegno.

We first used the original diploid-based SPA software to estimate the *a* and *b* function coefficient for each SNP and 2-dimensional x coordinate for each individual in the POPRES imputed dataset assuming a single parental original (i.e. both parents from the same location). We then estimated the appropriate x for each medieval sample assuming a single parental origin using the haploid version of the likelihood function for all callable SNPs for that sample. This analysis was implemented in Python using the a nonlinear conjugate gradient algorithm (fmin_cg

in scipy.optimize) with 5 random start points. The resulting inference strongly resembled the PCA analysis (Supplementary Figure 88).

We then took every set of medieval individuals for which there was an inferred parent-offspring relationship but for which data from one parent was missing. We then estimated the likely relative geographical location of the missing parent, y, conditional on the offspring haploid data and a known x. We used the same optimization strategy above, except we used 10 random start points, as well as start points that matched the final estimated position for the known parent of offspring from above. When there were more than one offspring per parent, we maximized the summed likelihoods across offspring (this will tend to weight offspring with more SNP data). When there was no known parent, we used avuncular individuals as surrogates for the missing parent.

There were eight sets of parent/avuncular-offspring relationships analysed using this approach, three from Kindred_SZ1 (including one pair with the same offspring but using different avuncular surrogates, four from Fam_CL1 and one from Kindred_CL2. Three sets (one from Kindred_SZ1 and two from Kindred_CL1) show very little to no difference between the geographical placement of the known and unknown parent (Supplementary Figure 89-91), while the other five show small (at the scale of the European continent) but noticeable differences that suggest admixture between parents of different ancestry within Europe (Supplementary Figure 92-96). For four of these cases, our inferences are supported by having more than one offspring. Using both SZ14 and SZ22 as surrogates, individuals SZ8 and SZ14 appear to be the result of mating between an individual from central/northern Europe and an unknown parent that resembles individuals from modern France. This is supported by the notieceably increased TSI

ancestry in the ADMIXTURE analysis for these offspring. Offspring CL83 to CL84 appear to have an unknown parent with more Scandinavian-type ancestry than the other sampled parent, CL87. CL87 themselves appears to be the result of mating between CL102 and an unknown parent from Northwestern Europe, which would explain why CL102 has increased/reduced TSI/CEU+GBR ancestry compared to CL87. Finally, CL53 and CL47 are inferred to have an unknown parent with greater eastern European ancestry than the sampled parent, CL49.

SUPPLEMENTARY NOTE 15. COMPARING GENETIC ANCESTRY, GRAVE GOODS AND MORTUARY PRACTICES

Carlos Eduardo G. Amorim, István Koncz, Daniel Winger, Caterina Giostra

A visual inspection of Fig 1 (main text) reveals that the distribution of grave goods (yellow dots) as well as the grave types (green dots) are not uniform across graves of individuals of different genetic ancestries. To statistically test for an association between genetic ancestry and archeology, we implemented a series of Fisher exact tests on contingency tables as described below. We first (a) classified individuals into genetic ancestry groups, then (b) tested the association between these and grave furnishing and typology, and finally (c) tested whether particular artifacts were more often seen in graves with individuals of a given ancestry.

15.1. Defining groups based on genetic ancestry

We first classified individuals into three categorical variables *N, S,* and *I,* according to their genetic ancestry estimates (see Fig 3 main text, Supplementary Note 8). We classified an individual as *N* if the proportion of genetic ancestry from Northern/Central European populations from the 1000 Genomes Project[71], namely CEU, GBR or FIN, had reached a given threshold *T.* In doing so, we considered the sum of the ancestry proportion estimates corresponding to these three populations. Conversely, we classified an individual as *S* if the estimated proportion of genetic ancestry from Southern European (TSI) or Iberian (IBS) populations from the same dataset had reached the same threshold *T*, again considering the sum of ancestries for these populations. If the estimates for a given individual did not reach the threshold *T*, we defined it as *I* and further excluded it from the statistical tests (but see subsection "d" below). *T* was initially

set to 70%, but other more extreme values were also considered (e.g. 60% and 90%). The use of different thresholds only changes the sample size minimally (Supplementary Table 5) and did not have a considerable impact on the statistical significance (Supplementary Table 6).

In these analyses, we did not consider individuals CL57 and CL94 because their graves presented signals of disturbance and there are reasons to believe the grave goods found in their tombs during excavation of the site are not representative of the actual grave furnishing at the moment of the burial of these individuals. Sample CL36 was excluded because it does not belong to the first period of occupation of Collegno as the remaining samples and sample CL31 was excluded due high contamination (~27%).

15.2. Is grave furnishing/typology associated with genetic ancestry?

A burial is a performance that has an important role in creating the connections between persons (living or deceased). Mortuary practices can shape memories the community has about the deceased, but ultimately they communicate how the living individuals chose to represent the one being buried. In some cases, they may tell us much more about how living individuals want to display their status and power, than anything about the deceased. For a more thoroughly discussion on these issues see [108–112]. In this regard, although it is difficult to connect legal or social status to archeological findings in general, the uneven distribution of specific artifacts may reflect social and cultural differences among individuals and kindred. For instance, in Szólád, weapons are commonly seen in graves with male adults but also amongst those with adolescents at the time of death: individuals SZ7, SZ14, and SZ15, for instance, are thought to be 12-17 yo. at the time of death[28] and were buried with spears, shield and/or swords. This does not

necessarily mean these individuals were warriors, but that weapons in this case may have been socially symbolic, potentially indicating the assigned status of those buried with them. Clearly not all male adults in these communities (warrior or not) were buried with weapons, so there was a possible conscious choice to bury a person with such artifacts, which in turn could tell us about different social roles amongst individuals or about different cultures and funerary customs. As another example, knives, combs, purses (with objects for personal use) and belt buckles (the type used for clothing, not for holding weapons) are very common to different cultures and regions during the Migration Period, and cannot be associated to any sort of social condition. In contrast to that, we see that certain traditions of pottery and jewelry, as well some behaviors such as the use of food offerings, are restricted to certain groups and/or periods, and thus are thought to reflect the presence of a certain group defined in terms of material culture.

The analyses in this section attempts to examine whether shared a common genetic ancestry (whether indirectly known by the individuals themselves or not) was a relevant aspect in shaping customs and mortuary practices in these societies. We are not assuming the individuals buried with a certain material culture marker or genetic ancestry were or were not Longobards, but we are inquiring whether biological relationship (i.e. shared genetic ancestry) was perceived as a structural element in this society, assuming if that is the case, then mortuary practices should be distinct for individuals of different genetic ancestries.

In both cemeteries we found graves that were devoid of grave goods and some that presented at least one of such artifacts (Fig 1B-C, main text). As a common characteristic of both cemeteries, all *N* individuals were buried with grave goods (e.g. jewelry, pottery, food offerings, knives, combs, weapons etc.). This is always true regardless of the different values of *T*. Graves

with *S* individuals were more variable (Supplementary Table 6), but with the exception of a couple of cases (SZ19 and SZ31, see discussion below) grave goods are everyday objects used by everyone, some of which are common to many different cultures in the Migration Period (e.g. purse with personal objects, comb, belt buckle and other clothes elements, knife). We do not see weapons buried with *S* individuals.

To test for an association between the presence of grave goods and genetic ancestry, we focused on those artifacts that are more restricted to certain cultures and periods, particularly weaponry and related accessories, stamped pottery, food offerings, and jewelry, the reason being that these can be considered cultural markers of a culture or a group. In Szólád we specifically looked at the following grave goods: jewelry (e.g. amulet, bracelet, rings, brooches etc.), strike-a-light, pottery, weapons (e.g. spatha, spear, shields etc.), beads (from necklaces or pendants), food offerings (animal bones, eggshells, etc.), and spindle whorls. In Collegno, the following artifacts were considered: beads, stamped pottery, weapons, and strike-a-lights. In both cases, the presence of grave goods is significantly associated with genetic ancestry (p-value < 0.05, according to a Fisher exact test applied on contingency tables), regardless of the value of T chosen (Supplementary Table 6), showing that certain grave goods are mostly found in graves with individuals of *N* genetic ancestry.

We also implemented the same test considering the different grave typologies. Grave types were either (i) simple pits or (ii) graves with wooden constructions. The latter were never documented in Mediterranean regions and cultures, and are somewhat similar across cemeteries. In Szólád, they presented ledges in the sidewalls supported by wooden beams covering the coffins (see Supplementary Figures 5 and 6). These were significantly more often seen among *N*

graves (p-value < 0.0001; Supplementary Table 8). In Collegno, the second type of grave was constituted by a wooden chamber that was not seen in the simpler grave type. The association between grave type and genetic ancestry is also significant in Collegno (p-value < 0.05; Supplementary Table 8).

In summary, we see that in both cemeteries individuals are accorded different mortuary practices depending on their genetic ancestry. Obviously these individuals were not aware of their genetic ancestry in ways we understand it, but indirectly there could be the recognition between persons of similar origins (e.g. from Northern/Central versus Southern Europe), i.e. a certain acknowledgement of belonging to a group that potentially share common customs and, due to a common origin, also share genetic ancestry.

Despite the statistically significant association between archeology and genetic ancestry, there are some exceptions to the general trend of "*N* → richer graves / *S* → simple graves". These exceptions are described in more detail below:

- SZ19: female *S* (100%) individual buried in the half-ring structure surrounding *N* male individuals (see Supplementary Figure 2). This individual is the only female buried with a bracelet (see Supplementary Figure 10). This type of artifact is uncommon in cemeteries commonly termed Longobard, but was common in Mediterranean cultures, associated with a late antique tradition. Moreover, this grave is also of the simple pit type and shallow, and contains a reduced amount of offerings of grave goods when compared to other graves in the same half-ring structure in this cemetery. We note that this woman was distantly related to individual SZ6, member of the large Kindred SZ1.

- SZ31: female *S* (99%) individual buried in a ledge-wall-type of grave amongst *N* women, in the half-ring structure surrounding the *N* male warriors. Her daughter is buried nearby. Along with SZ19, she is the only *S* individual that has a relative buried in Szólád. Together, this could potentially indicate that the barrier between groups was somewhat permeable, at least to some females.
- SZ38: *N* (89%) female individual buried in a simple pit (in contrast to other *N* women in that cemetery that have a ledge-wall-type of grave). Despite being a simple grave, some artifacts were buried with this individual, namely a ring, a pair of S-brooches and beads.
- CL151: *N* (100%) woman of advanced age. As opposed to other adult women in Collegno, only a belt buckle was found in her grave and her grave is a simple pit (i.e. does not contain a wooden chamber). The dating of her burial is imprecise, but the lack of a wooden chamber could be a consequence of temporal changes in grave architecture that is seen in Collegno (see below) or particular family customs, while the lack of grave goods could be due to her advanced age.
- CL97: *N* (100%) individual with simple grave (no wood chamber). This individual is likely from the early stage of occupation of Collegno, a time period where the wooden chambers were used in the region (as seen in other graves). The reason for such a discrepancy is unclear. However, this grave, as others with *N* individuals, was furnished with several weapons.

15.3. What artifacts are more often seen in graves with *N* individuals?

We further performed the test for each type of grave good independently (T = 70%; Supplementary Table 7), as opposed to considering their general absence/presence as above. In these tests, we ignored artifacts that were present with only one individual (being that *N* or *S*), and focused on the eight types of artifacts in Szólád and the three types of artifacts in Collegno that were seen in at least two graves. If an artifact was not seen buried with members of one sex, we excluded all individuals from that sex from the analyses. We also excluded male individuals that were younger than 12 yo. when we analyzed weapons.

Three grave goods are significantly more often buried with *N* individuals: beads (female) and food offerings (both female and male) in Szólád, and weapons (male) in both Collegno and Szólád. Additionally, we observe that stamped pottery and the articulated 5-pieces belts used to hold weapons (often decorated with animalistic patterns) are only seen with *N* individuals. We note that a chronological factor may also be relevant in this case since the more articulated kind of belt only appears in the Longobard-associated cemeteries around the year 600 CE, hence the low frequency of these amongst first phase graves in Collegno.

15.4. Notes on Kindred CL2

Kindred CL2 is located in the eastern part of Collegno. Genetic evidence supports the relationship between CL47, CL49, CL53, and CL57 (Fig 4). The position of grave CL48 between CL47 and CL49, in a single row, suggests these individuals could potentially be related (biologically, due to first-second degree relationship, or socially, for instance, as members of the same household) to the female buried in graved CL48. In fact, as noted in Supplementary Note 3, this grave contains artifacts not common to the region, with likely transalpine origins, similarly

to CL47. Moreover, the fact CL48 and CL47 both present a form of hereditary scaphocephaly further strengthens the possibility of this relationship being also biological.

Members of this kindred did not reach the threshold *T* for being considered *N* or *S* in the previous analyses, but we consider it is worth mentioning their graves contain artifacts that seem to be, at least in part, from a different tradition in comparison to other graves in Collegno (Supplementary Note 3), such as jewelry and accessories seen in the transalpine area. We also see that members of this kin group are the only ones seen with golden crosses in this early stage of occupation of Collegno.

In resemblance to graves with *N* individuals, graves CL47, CL48 an CL49 have wooden chambers, and adult males CL49 and CL53 were buried with weapons. Because of the way we implemented this analysis, i.e. considering the classification of individuals in two categories *N* and *S*, we were not able to include members of this kin group in the tests, but we see that certain artifacts and traditions are unique to members of a single kindred, giving further evidence for the idea that biological relatedness might have had been an important structural element in these societies.

SUPPLEMENTARY NOTE 16. ISOTOPE ANALYSES IN COLLEGNO AND COMPARISON TO SZÓLÁD

Susanne Hakenbeck

16.1. Background

Isotope analysis was carried out on individuals from Collegno to identify first-generation migrants and to determine whether such migrants might have brought with them dietary habits that were different from those of the local population of northern Italy. The data were compared with published data from Szólád and other sites in Italy and Hungary. The degree to which migrants changed their dietary habits to those of the population among which they settled may provide important clues about relationships between newcomers and the existing population[113].

Strontium and oxygen isotope values are used to determine whether an individual grew up where he or she was buried. $^{87}Sr/^{86}Sr$ values in organisms reflect the values of the underlying geology, with older geological formations generally having higher $^{87}Sr/^{86}Sr$ ratios than younger ones. Bioavailable strontium enters the food chain through water. Strontium isotope ratios are measured in enamel apatite which forms during childhood and juvenility. Isotopic values of tooth enamel are thus a reflection of the place of residence in childhood. Contrasted with local bioavailable $^{87}Sr/^{86}Sr$ values, they can be used to determine a change in residence since childhood[114].

Oxygen isotope ratios ($\delta^{18}O$) also vary geographically, primarily due to differences in temperature, becoming more depleted from the equator to the poles and with increasing altitude[115]. Across Eurasia values are also depleted from West to East, along with the prevailing winds. Via an offset, organisms reflect the isotopic value of drinking water, which in turn usually

reflects rainwater. Attempts have been made to develop a conversion algorithm for the offset from drinking water to body tissue for different species, but these have not been fully satisfactory[116,117]. Furthermore, in many published studies intra-population variation of oxygen isotope ratios is greater than large-scale geographical variations of precipitation (e.g. from Scandinavia to the Mediterranean)[118]. This may be due to water sources other than rainwater and changes in isotopic ratios due to cooking, brewing or stewing[119]. Our study therefore uses the unconverted values from enamel carbonate and restricts itself conservatively to intra-population comparisons. It is important to note that only individuals who grew up in a location with different geological strontium or water oxygen ratios can be identified as non-local. Strontium and oxygen isotope analysis can therefore only indicate a non-local upbringing, but it cannot definitely indicate a local upbringing.

Carbon and nitrogen isotope ratios in bone collagen, reported as $\delta^{13}C$ and $\delta^{15}N$, reflect the relative contribution of terrestrial, marine and freshwater resources to diet. Carbon isotope ratios enable us to distinguish dietary contributions from C3 and C4 plants, since these plants have isotopic ratios that do not overlap[120,121]. Isotopic values are enriched by approximately 1 to 2‰ from diet to body tissue[122]. Most plants, in particular most domesticated plants, utilise the C3 pathway, while the C4 pathway is used by plants in hot and dry climates, mostly grasses and sedges. They key cultivars in pre-colonial Europe are millet and sago, while maize plays an important role in the Americas. In Europe millet is considered one of the minor crops and has been used preferentially in unstable climatic or economic conditions[123].

Nitrogen isotope values allow us to determine the relative amount of animal protein (meat or milk, including breast milk) consumed by an organism. $\delta^{15}N$ is enriched by about 3‰

with each trophic level[124,125], though the enrichment can be as high as 6‰ in some instances[126]. Both freshwater and marine organisms are elevated in $\delta^{15}N$, though freshwater fish in particular are strongly affected by their ecological context[127].

16.2. Environmental context and samples

The site of Collegno is located on the floodplain of the river Po and beside a small tributary, the river Dora. The floodplain is made up of Pleistocene gravels. To the west are the Alpine foothills; this area is made up of Pleistocene glacial deposits and an oophiolite complex of serpentinite and green schist. To the east there is an outcrop of Miocene clays and marls. To determine 'local' bioavailable strontium values, samples of water, vegetation and soil were collected both at the site of the cemetery of Collegno and at locations considered to be about a day's walk from the site that are geologically different from the plain (Supplementary Figure 98), following the procedure outlined in Maurer et al.[128]. Water was collected from the rivers Po and Dora and from a small lake to the west of Collegno. Soil samples were taken with an auger from the base of the topsoil, reaching depths of 0.25 to 0.70 m. Fields under cultivation were avoided to mitigate the impact of fertiliser use. Similarly, tree leaves were collected only from areas far from cultivated fields.

Fauna can provide a local ecological baseline for human diet. We chose six from different species from an excavation in Piazza Castello in Turin, dating from the first to third/fourth centuries AD, since no contemporary fauna was available. Bone samples from fauna were variable, though cortical bone was preferred. Human bone samples (0.5-1.5 g) were taken from ribs where available, otherwise from long bones. For tooth samples, the second premolar

was preferred since its crown begins to form from two years of age and is completed by seven years[129]. It therefore retains information about childhood residence.

16.3. Analytical methods

Human teeth and bones were collected from burials at Collegno (Supplementary Note 3) targeting the first period. Environmental samples for strontium analysis were pre-treated following the methods described in Maurer et al.[128]. Water samples were filtered through 0.2um nylon filters into 60ml acid cleaned HDPE bottles and acidified with distilled ultra-pure nitric acid to maintain a pH = 2. Soil leachates were obtained by shaking 2 g of soil in 20 ml deionised ultrapure water for 24 h in acid-cleaned polypropylene tubes, followed by 1 h in an ultrasonic bath. The solution was centrifuged for 15 minutes at 2000 rpm and filtered through 0.2um nylon filters. Water and soil leachates were evaporated to dryness. Leaves were rinsed with deionised ultrapure water and dried in an oven at 50°C overnight. Approximately 1 g of dried leaves was then ground and ashed in acid-washed silica crucibles at 550C for 12 h. All samples were then transferred into acid-cleaned Teflon vials. For strontium isotope analysis of tooth enamel, the enamel surface of the tooth was abraded to a depth of >100 microns using a tungsten carbide dental burr and the removed material discarded. Tooth enamel powder was then collected, and the samples were transferred to a clean (class 100, laminar flow) working area for further preparation. In a clean laboratory, the samples were first washed in high purity acetone to remove any grease that might have come from handling the enamel. Then the sample was cleaned ultrasonically in high purity water, rinsed, dried and then weighed into pre-cleaned

Teflon vials. The samples were mixed with ^{84}Sr spike solution and dissolved in Teflon distilled 8M HNO3.

Strontium isotope samples were analysed at the Isotope Geochemistry Laboratory of the Department of Earth Sciences, University of Cambridge. Strontium was separated using Dowex 50 x 8 (200-400 mesh) cation exchange resin and the strontium isotopic ratios were measured on single Ta filaments on the VG Sector 54 TIMs using triple collector dynamic algorithm, normalised to ^{86}Sr/^{88}Sr 0.1194 with an exponential fractionation correction[130]. The 46 analyses of NBS 987 during the two year period up to and including these analyses gave a mean value of 0.710257+/-0.000006 (1 sigma).

For oxygen isotope analysis of enamel apatite, tooth enamel powder was obtained using a using a dental drill with a diamond drill attachment. The exterior of the enamel was mechanically abraded to remove any dirt, and the drill bit was cleaned before each sample was taken. The bioapatite extraction method is described in Balasse et al.[131]. Enamel was treated with sodium hypochlorite 2–3% (24 h) to remove organic matter and then with 0.1 M acetic acid (4 h, 0.1 ml/mg) to remove exogenous carbonate. The samples were lyophilised to remove any remaining liquid. The samples were then transferred into vials sealed with a screw cap holding a septa and PCTFE washer to make a vacuum seal, and the samples reacted with 100% orthophosphoric acid at 90°C using a Micromass Multicarb Sample Preparation System. The carbon dioxide produced was dried and transferred cryogenically into a VG SIRA mass spectrometer for isotopic analysis. Results are reported with reference to the international standard VPDB calibrated through the

NBS19 standard[132,133]. The precision is better than ±0.08‰ for $^{13}C/^{12}C$ and better than ±0.10‰ for $^{18}O/^{16}O$.

Collagen was extracted from human and faunal bones following the standard laboratory protocol of the Department of Archaeology, University of Cambridge, based on the method detailed by Privat et al.[134]. The surfaces of the bone pieces were cleaned by sand-blasting. The pieces were demineralized in 0.5 M aq. HCl at 4°C until all mineral was dissolved (7-14 days). Samples were rinsed with distilled water and then gelatinized in acidic solution (pH 3) at 75–78°C for 48 h. The liquid fraction containing the gelatinized protein was frozen and lyophilised. Approximately 0.8 mg portions of this final 'collagen' product were used for each analysis. Samples were analysed in triplicate at the Godwin Laboratory, University of Cambridge, using an automated elemental analyser (Costech) coupled in continuous flow mode to an isotope ratio-monitoring mass spectrometer (Finnigan MAT253). Results are reported using the delta scale in units of 'per mil' (‰) relative to internationally accepted standards, VPDB for carbon and AIR for nitrogen. Measurement errors are less than ±0.2‰ for $\delta^{13}C$ and $\delta^{15}N$, as determined by replicate analyses of international and laboratory standards.

16.4. Results

The full results of human and fauna samples together with relevant osteological and genomic data can be found in Supplementary Tables 9-11. All collagen samples produced collagen of good quality. The atomic C/N ratios were between 3.09 and 3.22, well within the range of 2.9-3.6 considered to be indicative of good collagen preservation[135]. Every collagen sample

yielded carbon in excess of >13% and nitrogen in excess of 4.8%; many samples were in the range of modern human collagen (40-50% carbon and 15-18% nitrogen), as defined by Ambrose[136].

Evidence for migrants at Collegno.

Bioavailable strontium isotope values local to the cemetery and hypothetical settlement at Collegno were determined by soil and vegetation values near the site, with the values of Po and Dora as likely endpoints (Fig 4). Environmental values from the Alpine foothills and the marl outcrop further away are elevated compared to these, in line with the greater antiquity of the underlying geology. The four sub-adults at Collegno fall within the local strontium isotope range, indicating that they grew up nearby. A number of adults, however, lie well outside the local range. Of these, one female individual (CL147) and two males (CL23 and CL94) have isotopic values that do not occur near Collegno, while several others are very close to the local environmental end values (female individuals CL48 and CL102 and males CL49, CL93 and CL9). These likely did not grow up locally, but moved to Collegno from another location. This interpretation is supported by the oxygen isotope data (Supplementary Figure 99). Here CL94 and CL147 are also outliers and at opposite ends of the data range. The woman from grave CL25 has an oxygen isotope value that is similarly enriched to that of the female individual CL147. However, her strontium value is local. This woman might be a migrant, but the evidence is not conclusive.

When considering the genomic ancestry groups together with the isotopic evidence, we find that the four individuals with >70% TSI ancestry are likely local to Collegno (with uncertainty surrounding CL25). The two men with >50% 'Iberian + southern' ancestry clearly did not grow up locally, while two of the adults with 50-70% 'northern' admixture (CL102 and 49) are probably not local. The strontium isotope values of the individuals with a contribution of >50% 'northern' ancestry have a greater range than those of the individuals with a greater contribution of 'southern' ancestry, suggesting generally higher levels of mobility. The rest are genetically undetermined, but of these, several did not grow up locally. Excluding the subadults, about 28% of the sampled population can be considered not to have grown up near Collegno.

Considering kinship relationships at Collegno (Fig. 3C, Supplementary Figure 100), the woman in grave CL102 who is of the first known generation of Kindred CL2 has a low $^{87}Sr/^{86}Sr$ value, making it likely that she did not grow up locally. Her daughter, in grave CL87, also has a low strontium isotope value, though not quite as low as her mother's, also making her a potential migrant. She may have grown up elsewhere, or perhaps she moved with her mother while her permanent teeth were still forming. Similarly, the man in grave CL93 may not have grown up in Collegno, but his son in grave CL92 apparently never moved. In Kindred CL2 the man in grave CL49, of the first known generation, also had a likely non-local $^{87}Sr/^{86}Sr$ value, making him a migrant to Collegno. His son, daughter and nephew however (CL53, CL47 and CL57) had local strontium isotope values, suggesting that they spent their entire lives close to Collegno. This means that the woman in grave CL102 and the man in grave CL49 were first-generation migrants to Collegno, while all individuals who were in the third generation in either kin group were likely born locally.

A comparison of ancestry groups with the evidence of mobility at Szólád, as previously published in Alt et al.[28], shows much greater heterogeneity than at Collegno. Like at Collegno, most children display local isotopic values. However, at Szólád there is a distinct cluster of children with >70% 'northern' ancestry who clearly all grew up together (identified as Range II in [55]), whereas the two children with >70% 'southern' ancestry (SZ36 and 40) grew up in a different location from them (in Range I). The adults are highly variable. Individuals with >70% 'northern' ancestry have a greater range of strontium isotope values than those with >70% 'southern' ancestry. The latter group may have been local to the Balaton environs, based on bioavailable reference data from the region, but their strontium isotope values do not overlap with the tight cluster of children in 'range II'.

At Szólád an evaluation of mobility across generations is more difficult, since not enough isotope data are available for the complex pedigree of Kindred SZ1. However, a comparison of individuals from the second and the third generation shows that all adults were highly mobile (SZ13, SZ22 and ZZ14) and only the children can be linked to Szólád. This suggests a kin group moving together and only settling in Szólád when the children SZ6 and SZ8 were born (but presumably before the birth of SZ14).

Dietary variation

To determine the diet of the population of Collegno the isotopic values of humans were considered in the context of the local fauna. The faunal results are within the expected ranges for terrestrial mammals in a temperate ecosystem[137] (Supplementary Tables 12-13). The herbivores

fed primarily on C3 plants, while isotopic values of the omnivores (dog, chicken) are very similar to those of humans, indicating considerable input of human-provided fodder or remains of human diet. The pig, also an omnivore, has a $\delta^{13}C$ value more like the herbivores, suggesting that it foraged in the wild for some of its life. In line with commonly observed offsets between humans and fauna in the same ecosystem, there is an increase of about 3‰ in $\delta^{15}N$ and 3 to 4‰ in $\delta^{13}C_{coll}$ values from the two herbivores to the average human diet[125,138,139]. This may indicate that roughly around 60% of the protein was derived from animal proteins [125]. There is no indication that fish played a significant role in diet. The two-year old child in grave CL83 had an elevated $\delta^{15}N$ value, likely because she was still breastfed at or close to her death. The $\delta^{13}C_{coll}$ values of humans suggests a diet of mostly C3 plants with an approximate contribution of 20 to 30% of C4 plants[139].

Considering the diet of individuals at Collegno and Szólád in the context of their migration status and ancestry, we can see that the non-local individuals are not marked out by a distinctly different diet (Supplementary Figure 97, 101). At Collegno both $\delta^{13}C_{coll}$ and $\delta^{15}N$ values are distributed across the entire data range, while at Szólád the diet of non-local individuals firmly lies in a central cluster. We note however that isotopic information from their childhood diets, prior to their migration, would be obscured by continued bone remodelling in adulthood[140]. Conversely, the three individuals who were dietary outliers at Szólád (graves SZ5, SZ23 and SZ44) were probably local, according to their strontium isotope values.

At both Collegno and Szólád individuals with >70% ‘southern’ ancestry had lower levels of $\delta^{15}N$ than other ancestry groups, if we exclude infants whose values are elevated due their

consumption of breastmilk (Supplementary Figure 102-103). This suggests that members of this ancestry group had less access to animal protein than others at these sites.

16.5. Discussion

As was already suggested[28], the population at Szólád was heterogeneous and composed of different kin groups that experienced several shifts in residence prior to settling and dying at Szólád. The most mobile individuals were those with >70% 'northern' ancestry, though there are exceptions (e.g. the female in grave SZ28). Conversely, all individuals with >70% 'southern' ancestry had relatively homogenous $^{87}Sr/^{86}Sr$ values, suggesting a possibly shared background that was nevertheless in a different place from what has been interpreted to be local for Szólád[28]. In Collegno, on the other hand, there is evidence for the migration and settlement of specific kin groups. We can identify several first-generation migrants to Collegno (individuals in graves CL49, 102 and perhaps 93). The following two generations are then firmly settled at Collegno. Here, as at Szólád, individuals with >70% 'northern' ancestry display greater levels of mobility than those with >70% 'southern' ancestry, suggesting that the 'southern' group was largely local. Two individuals with >50% Iberian and some 'southern' ancestry (in graves CL23 and CL94) are also clearly not local to Collegno, though they are not related to the other kindred. At both sites women experienced somewhat higher levels of mobility than men. This is in keeping with patterns that have been identified elsewhere and quite likely relates to exogamous social structures that seem to have been prevalent in many regions throughout the early medieval period[141–143].

At both sites, individuals with greater degrees of 'southern' admixture had less access to animal protein, suggesting that they may have been less privileged compared to members of other ancestry groups. Social distinction through preferential access to animal protein has been observed in early medieval Bavaria[144,145], as well as more generally among elites in prehistory and the medieval periods[146]. At Szólád and Collegno the 'southern' individuals, who were also more local, may have been in a socially inferior position to the more mobile individuals with different ancestry. This suggests that the migration of the latter groups did not diminish their social standing. Instead, they were able to maintain a position of privilege. This is also evident in the high levels of burial wealth deposited in some of their graves, as well as an abundance of weaponry. The older female buried in grave CL48, for example, was buried with a rich assemblage, made up of a pair of fire-gilded brooches and a gold foil cross[37]. She had the most enriched $\delta^{15}N$ value of all individuals at Collegno and had not grown up locally.

Finally, the migration of these groups does not appear to have brought about large scale dietary changes in their host environment (Supplementary Figure 104, Supplementary Tables 10-11). Unfortunately there are so far no data available from late antique cemeteries in northern Italy that would allow us to track potential dietary shifts over time. A comparison with Cividale (Gallo and S. Stefano) and Romans, sixth- and seventh-century sites in Friuli that have been identified with Longobards[147], shows that the population from Collegno consumed fewer C4 plants than those populations, while their animal protein consumption was similar. The most likely C4 contribution came from millet. Castiglioni and Rottoli identified a small amount at Collegno, but there was considerable evidence for millet at other sites in the Piedmont[123]. In Pannonia, a comparison of the data from Szólád with two fifth-century cemeteries in the Balaton

region, Hács-Béndekpuszta and Keszthely-Fenékpuszta, shows that local diet remained relatively consistent in the fifth and sixth centuries, though with the caveat that extremely high levels of mobility have also been identified at Hács-Béndekpuszta and Keszthely-Fenékpuszta[148–150]. The populations at all three sites consumed a considerable amount of C4 plants, most likely millet, with a slight drop-off in the sixth century. Individuals at Szólád had slightly more access to animal protein than those at Hács-Béndekpussta and Keszthely-Fenékpuszta, but the pattern is skewed by the large number of children with elevated $\delta^{15}N$ due to breastfeeding who were sampled in Szólád but not at the other sites.

Across all six sites, there is no clear evidence of a distinctly different diet in Pannonia and in Italy. All populations consumed C4 plants, with considerable amounts at Cividale and Romans. The populations of the Italian sites seem to have consumed slightly less animal protein than those in Pannonia, though this may also be due to the slightly different ecological context of northern Italy. The isotopic values of $\delta^{13}C_{coll}$ and $\delta^{15}N$ at Collegno are fairly tightly clustered, suggesting that diet was relatively homogeneous for the population there. It is notable that both Pannonian and Italian sites were enriched in $\delta^{13}C_{coll}$ compared to early medieval sites north of the Alps[113,151–153]. In the absence of comparative data from late antique other sites from northern Italy it is so far still difficult to ascertain whether these dietary habits, especially preference for millet consumption, were broadly similar in Pannonia and in northern Italy as a consequence of migration.

16.6. Conclusion

At both Szólád and Collegno there is a pattern of migration by elite kindred who have high percentages of 'northern' ancestry. At Szólád several of these individuals experienced more than one change in residence before settling together with a group of individuals with high percentages of 'southern' ancestry who were also not local to Szólád. At Collegno it has been possible to identify first-generation migrants with 'northern' ancestry, who were followed by two or three generations of stable settlement. They settled among individuals with high percentages of 'southern' ancestry. At both sites, the individuals with higher levels of 'southern' ancestry appear to have been a less privileged local class, as indicated by their reduced access to animal protein and fewer grave goods. The migration and settlement of the more privileged outsiders appears not to have brought about a dietary shift, suggesting that the new elites may not have concerned themselves with subsistence agriculture directly, having instead the ability to access local resources through their privileged status.

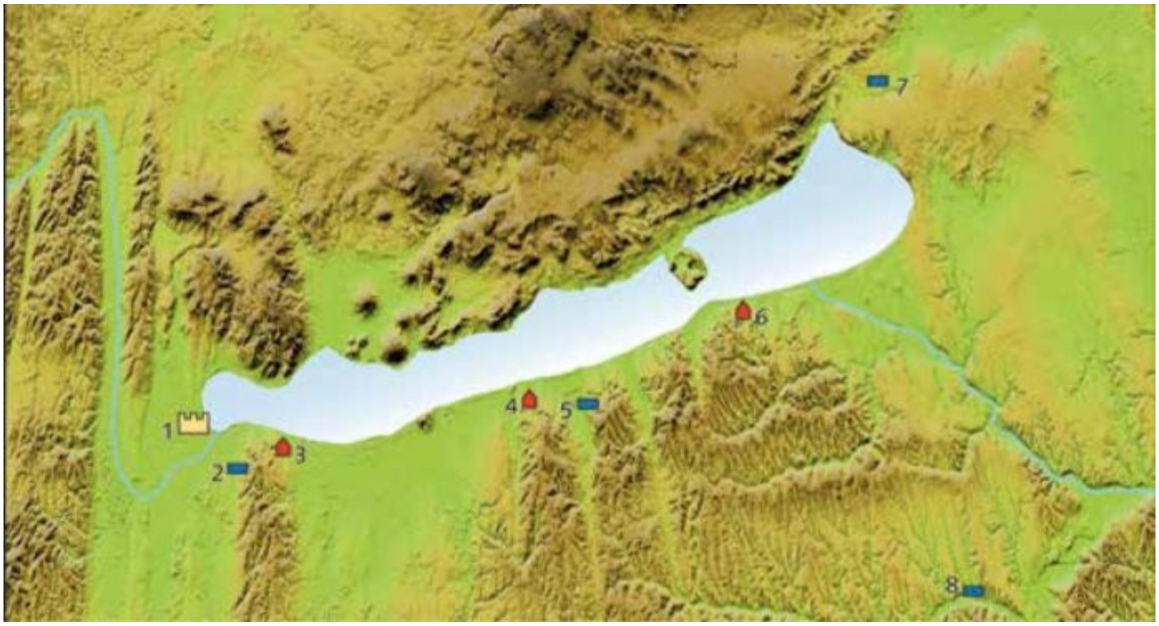

Supplementary Figure 1. Map of the Longobard period Balaton lake region. Color coding: yellow = late antique; blue = cemeteries, red = settlement. (1) Fortification of Keszthely-Fenékpuszta; (2) Vörs, (3) Balatonkeresztúr, (4) Balatonlelle, (5) Szólád, (6) Zamárdi, (7) Várpalota, and (8) Tamási. The image is edited from a publicly available digital elevation model from NASA's Shuttle Radar Topography Mission (https://www2.jpl.nasa.gov/srtm/).

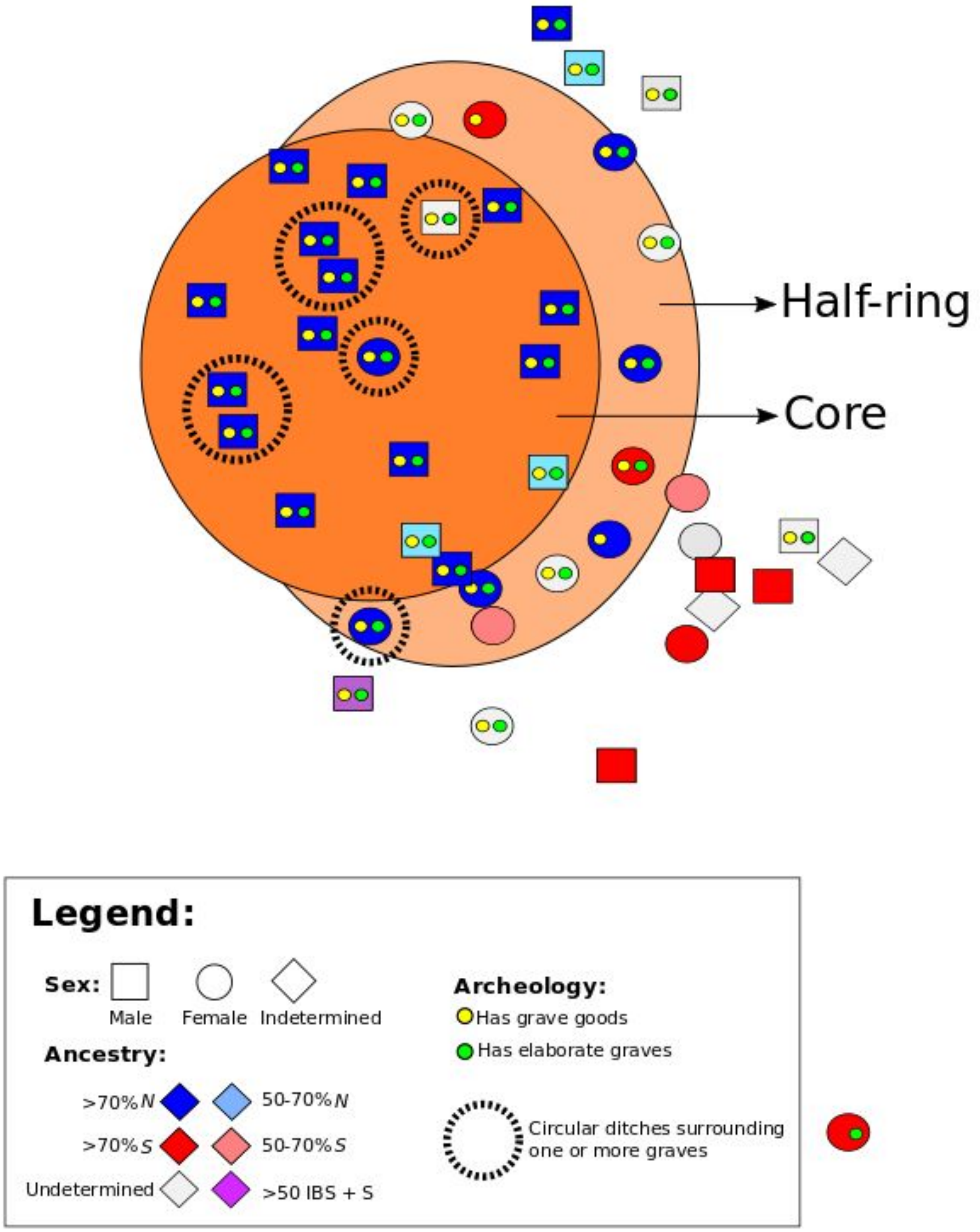

Supplementary Figure 2. Core/half-ring structure of graves in Szólád. Symbols follow the same specification as defined for Fig 1 in the main text.

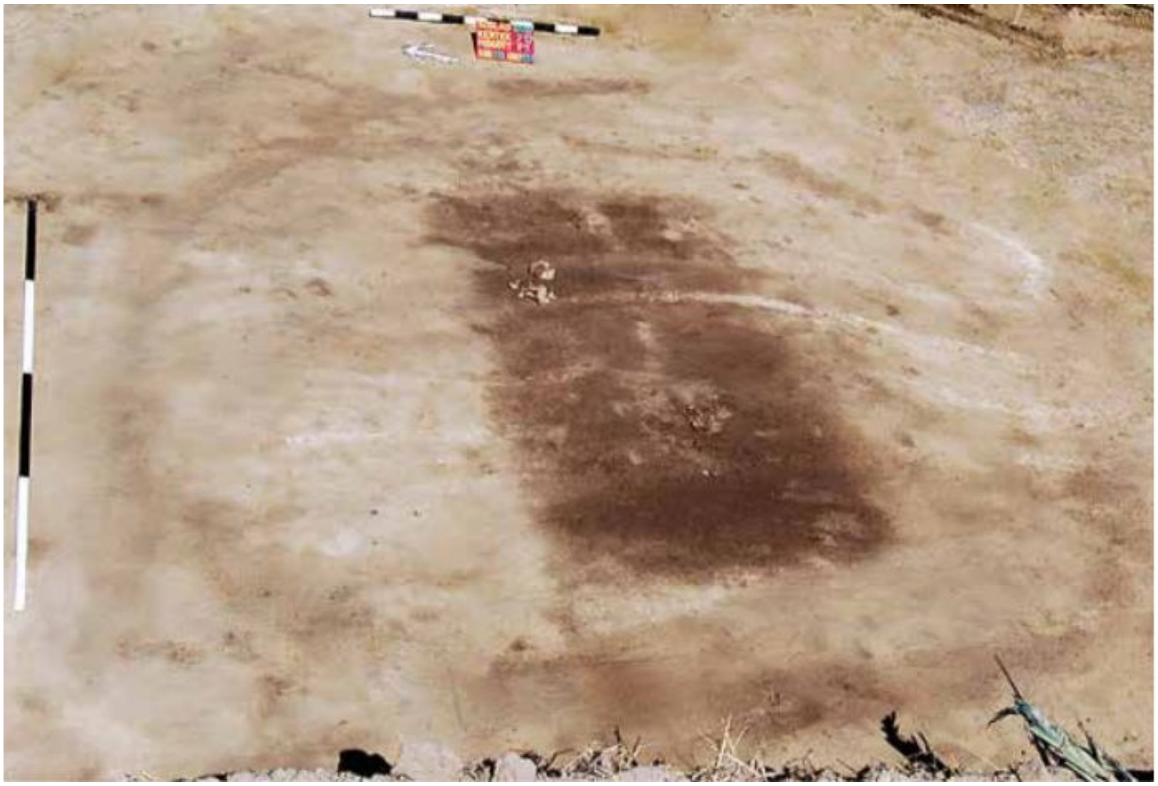

Supplementary Figure 3. Szólád grave 30 during the excavation ringed by a circular ditch.

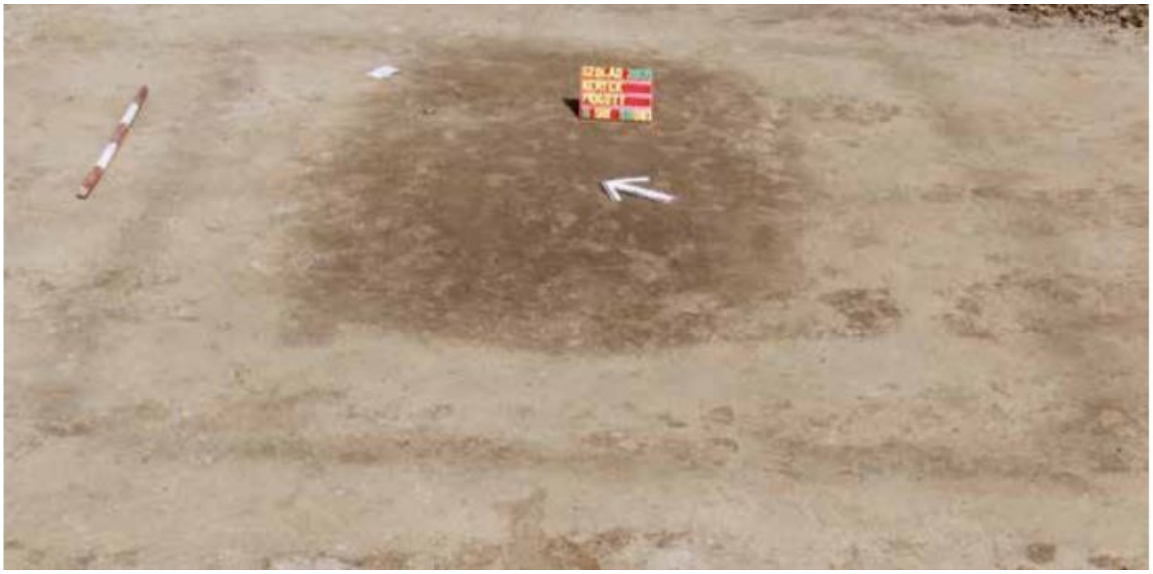

Supplementary Figure 4. Szólád grave SZ5 during the excavation surrounded by a rectangular ditch.

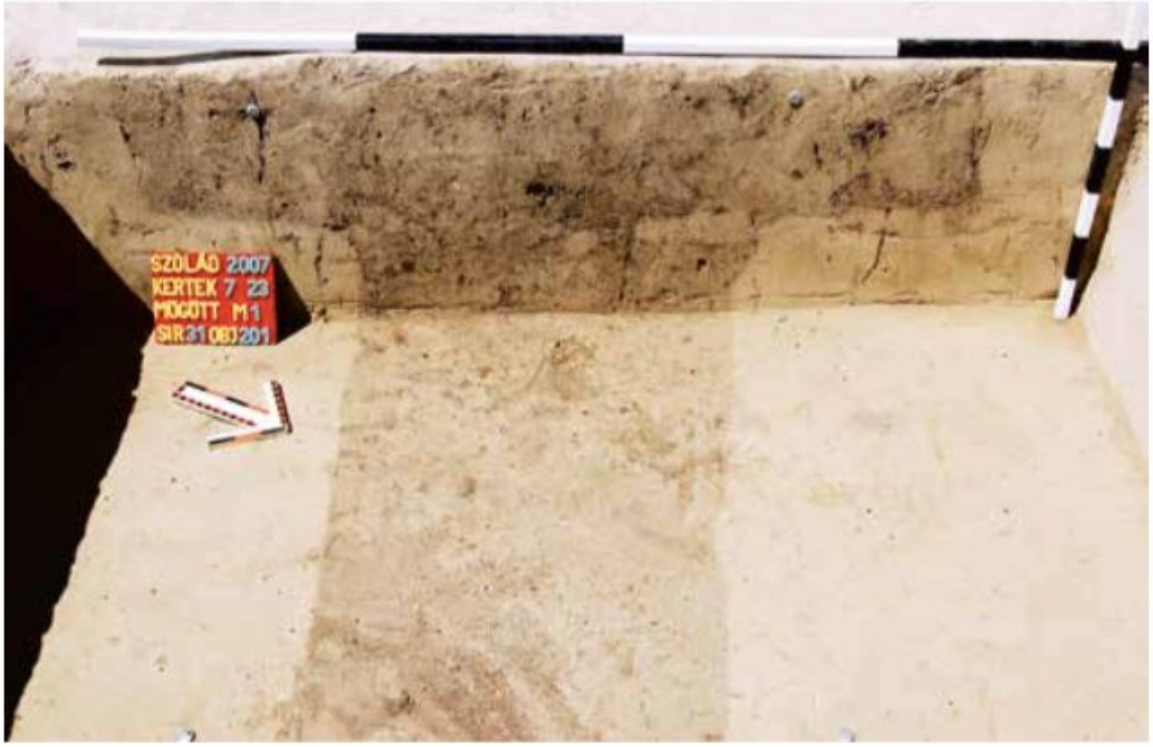

Supplementary Figure 5. Szólád grave SZ31 profile of the ledges.

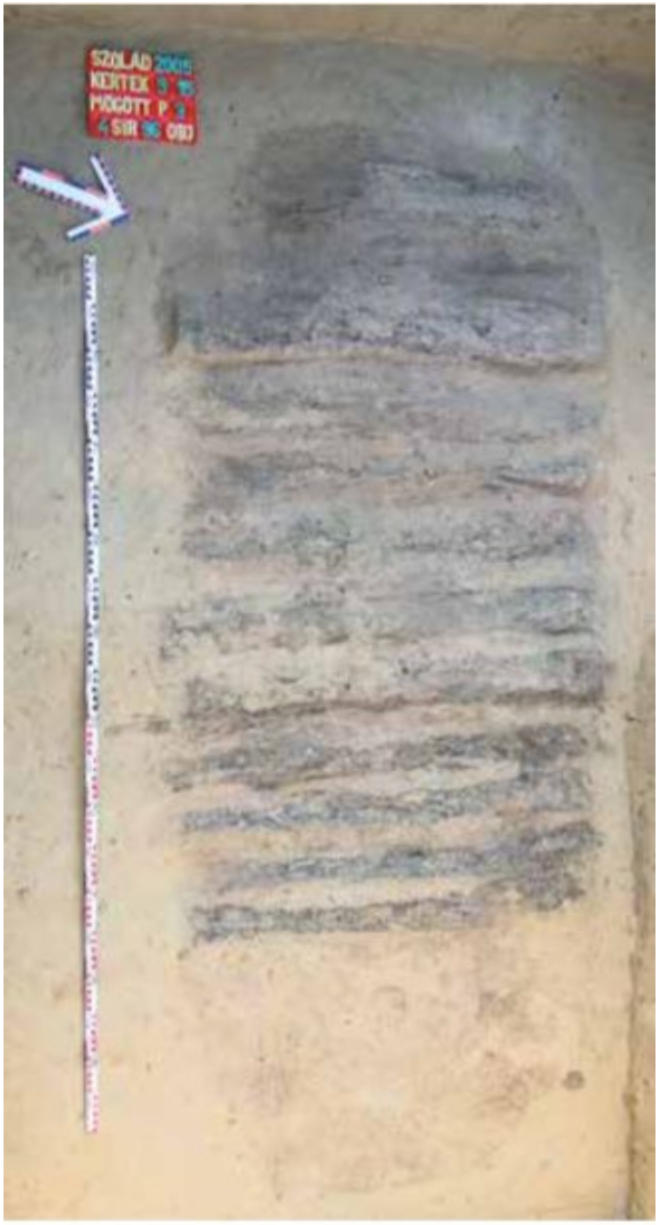

Supplementary Figure 6. Szóládgrave SZ4 during the excavation with an intact wooden layer lying on the ledges above the grave.

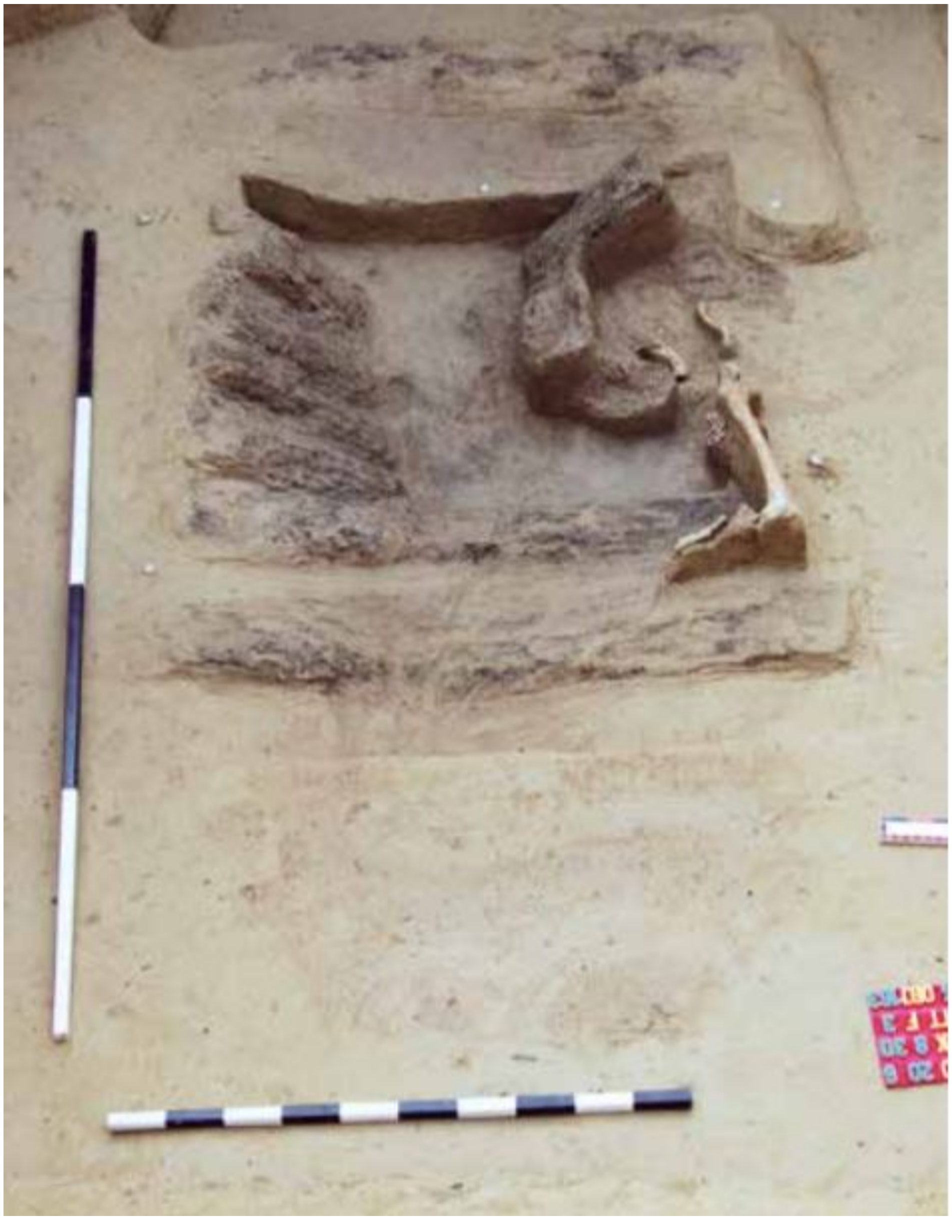

Supplementary Figure 7. Szólád grave SZ25 during the excavation with wooden broken layer due to antique grave robbery.

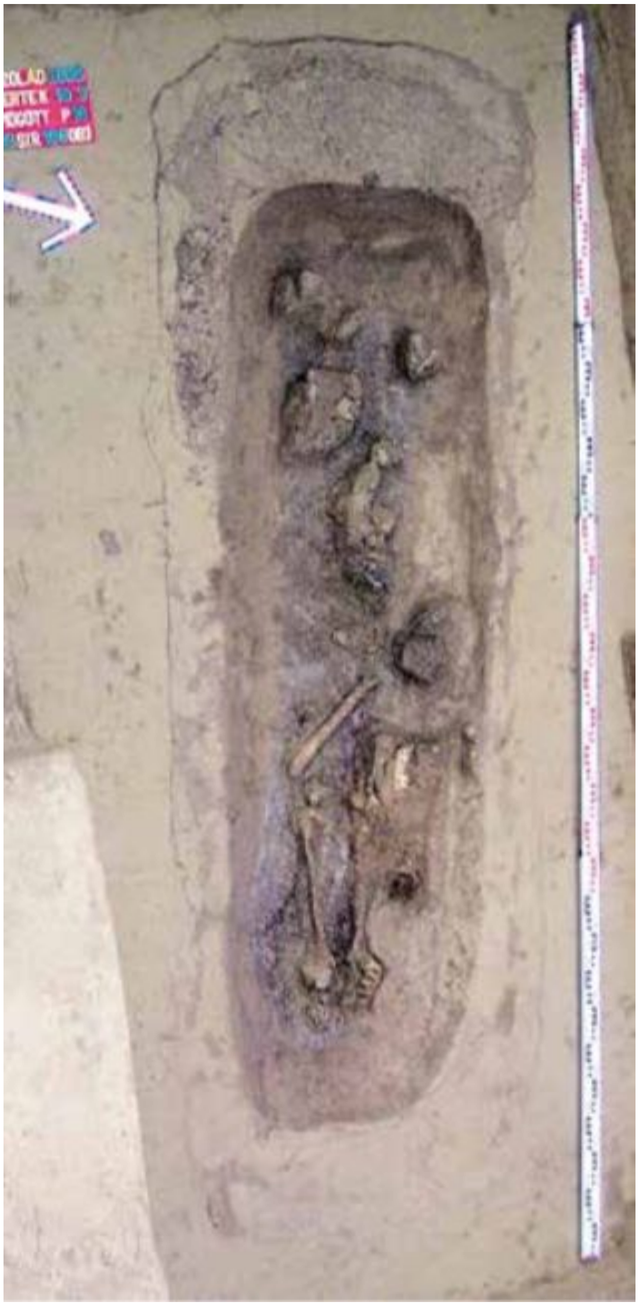

Supplementary Figure 8. The rounded corners are the result of tree coffins, as seen here in the main grave of Szólád (SZ13).

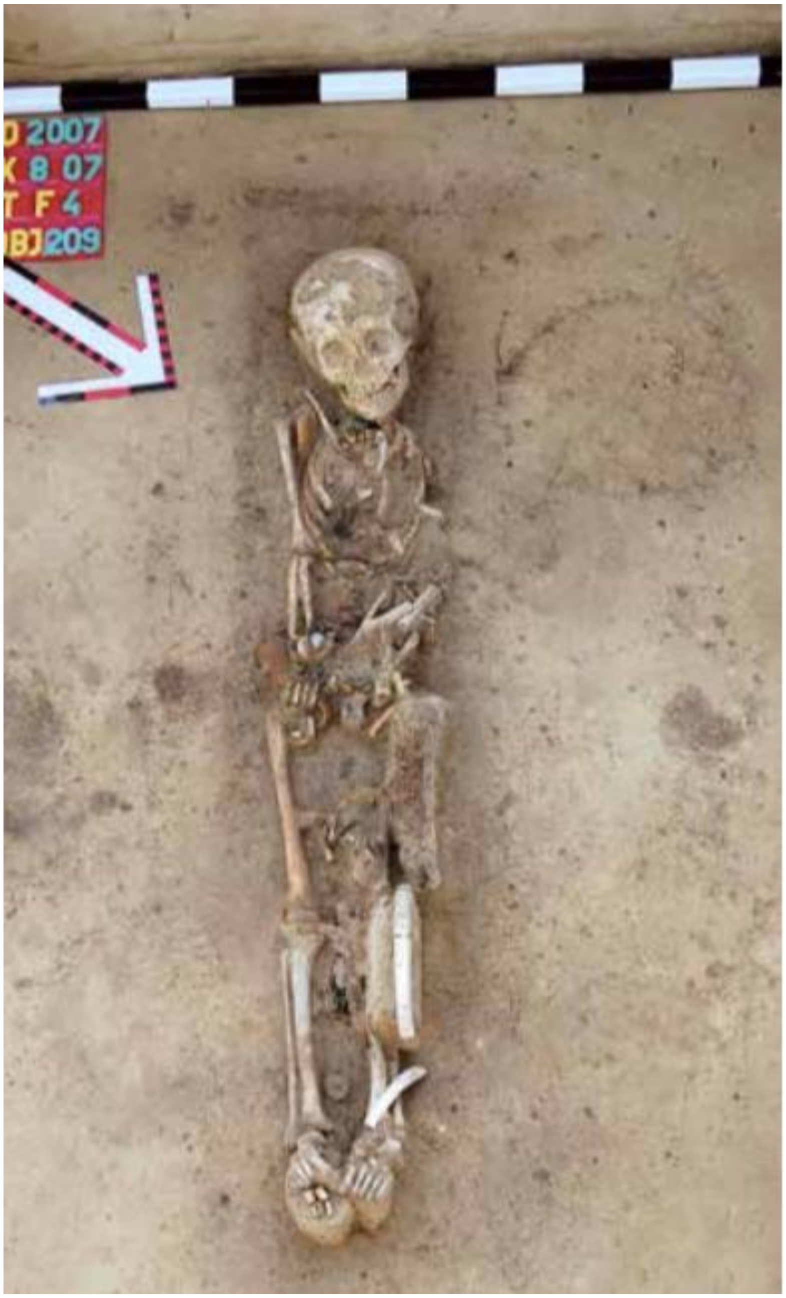

Supplementary Figure 9. Opposite tree coffins there are rectangular wooden coffins used, for example, in the case of the rich equipped girl in Szólád grave SZ38.

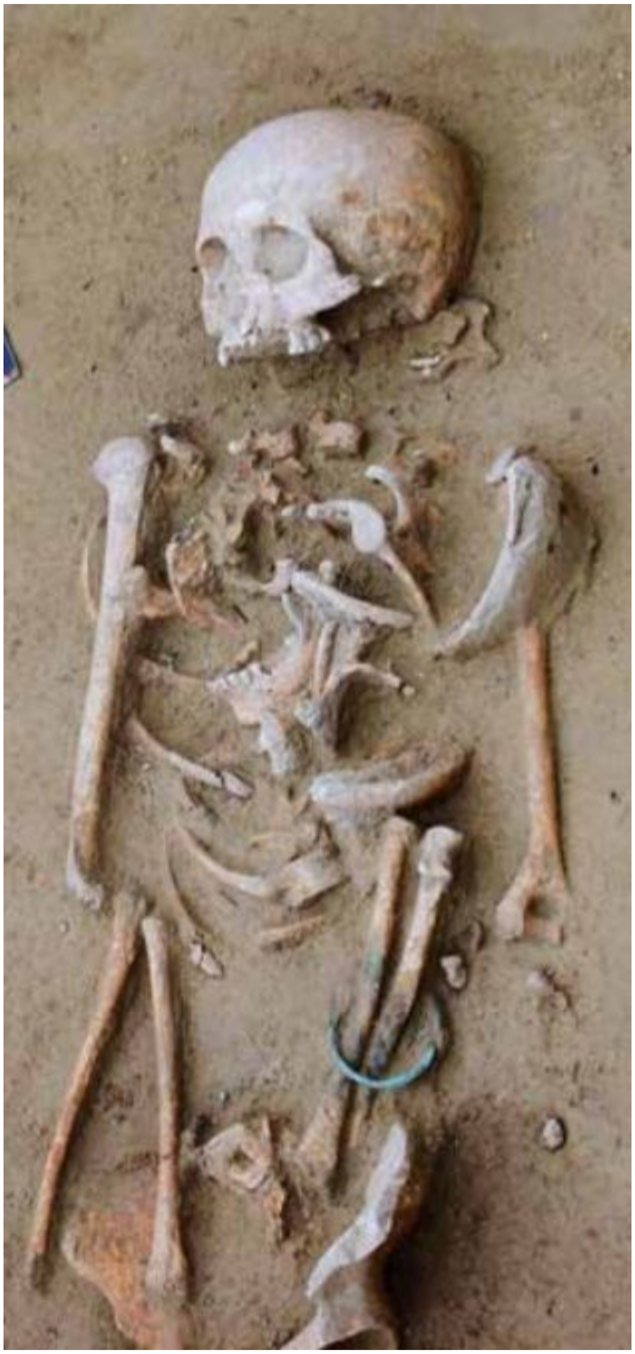

Supplementary Figure 10. Szólád grave SZ19 (female) with straight walls and a bracelet. Grave construction and goods in this case are different from the typical Longboard period graves.

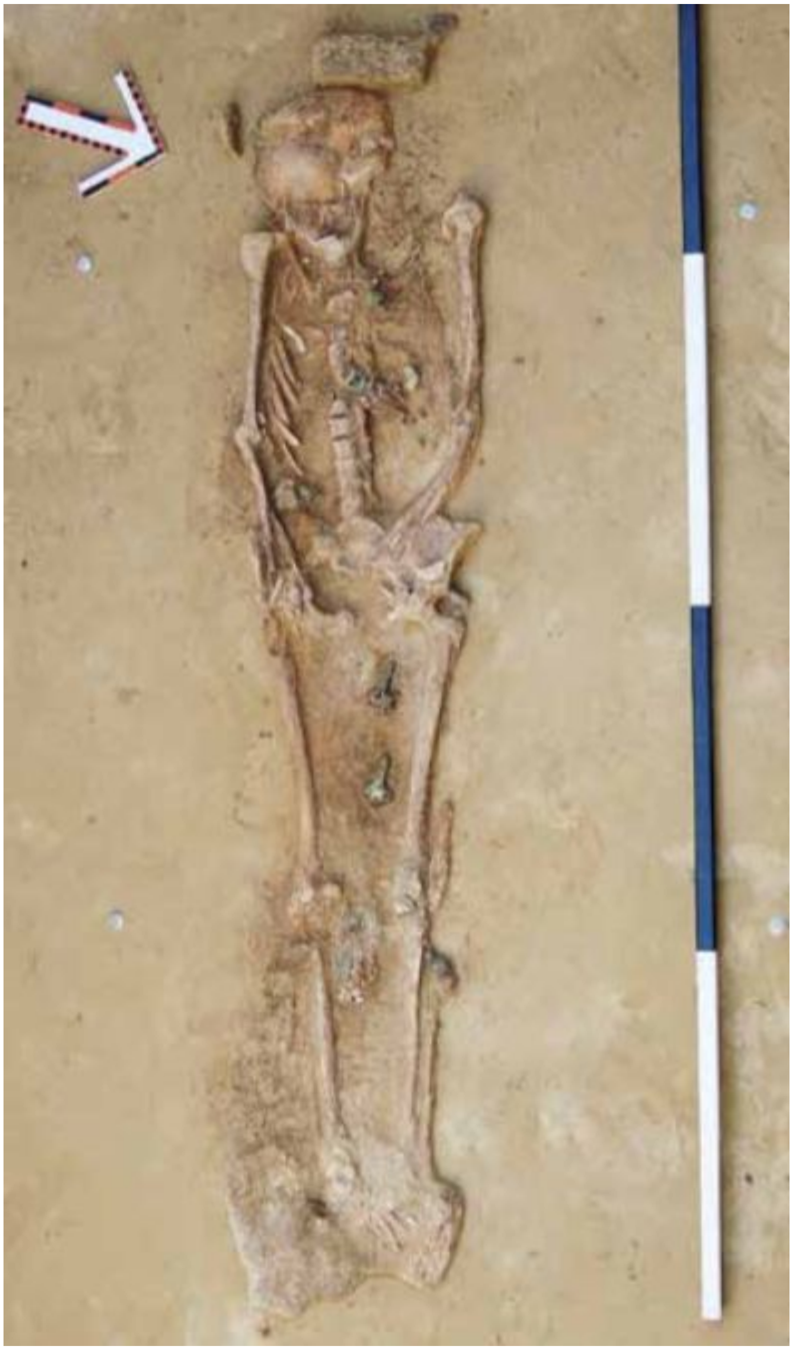

Supplementary Figure 11. Four brooch costume in Szólád grave SZ21.

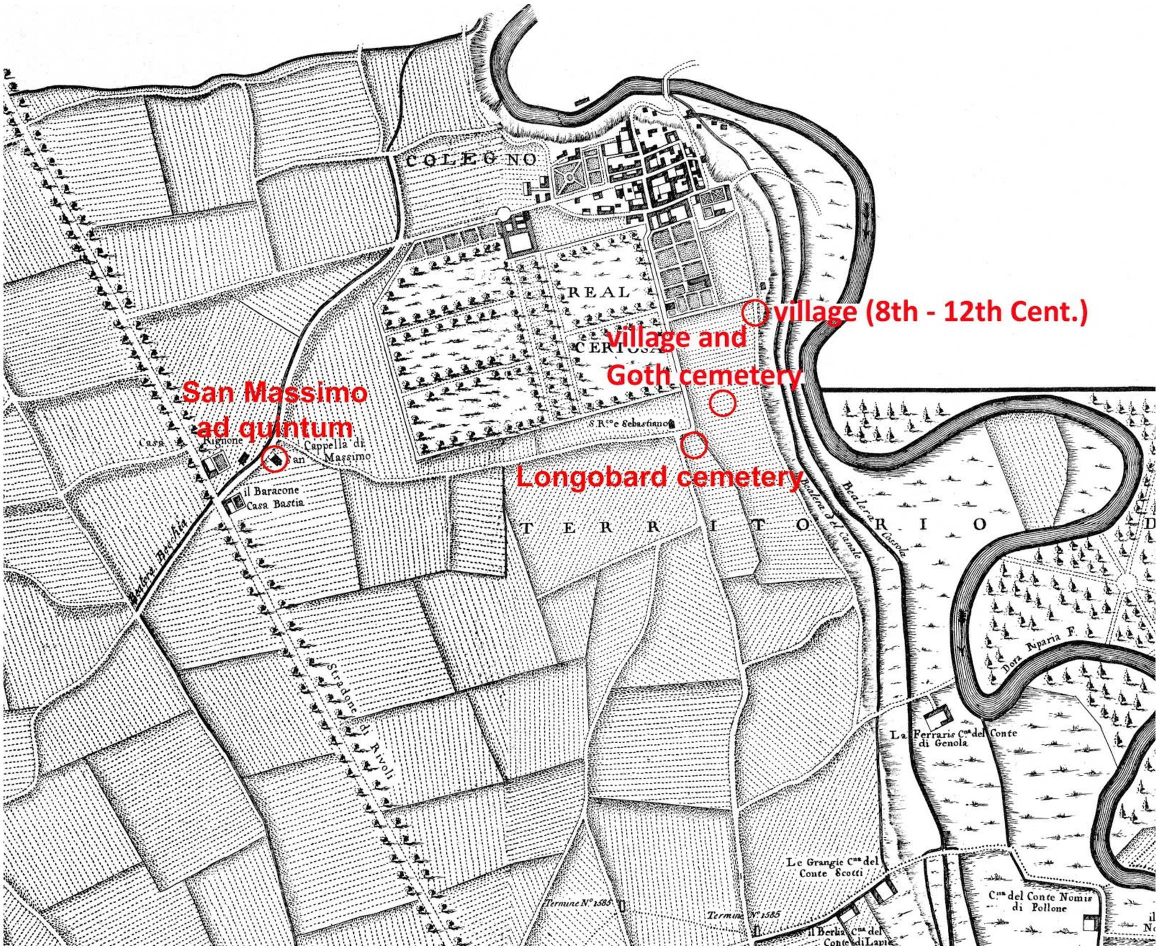

Supplementary Figure 12. Map of the site at Collegno. Modified from 1790 map by Giovanni Amedeo Grossi[154].

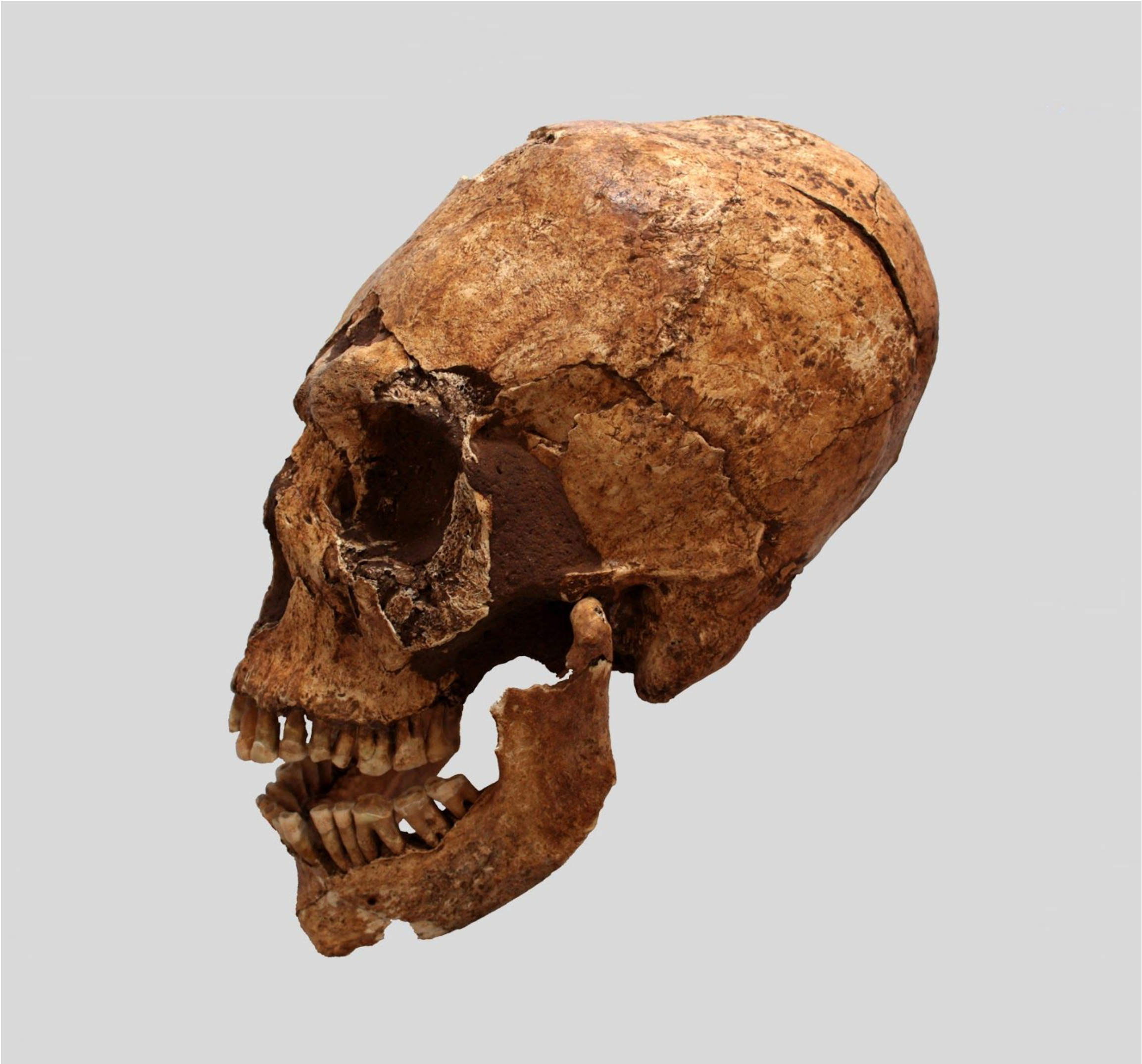

Supplementary Figure 13. An artificially modified skull from the Gothic-era cemetery found at Collegno. Image source: Soprintendenza Archeologia, Belle Arti e Paesaggio per la Città Metropolitana di Torino, archivio fotografico.

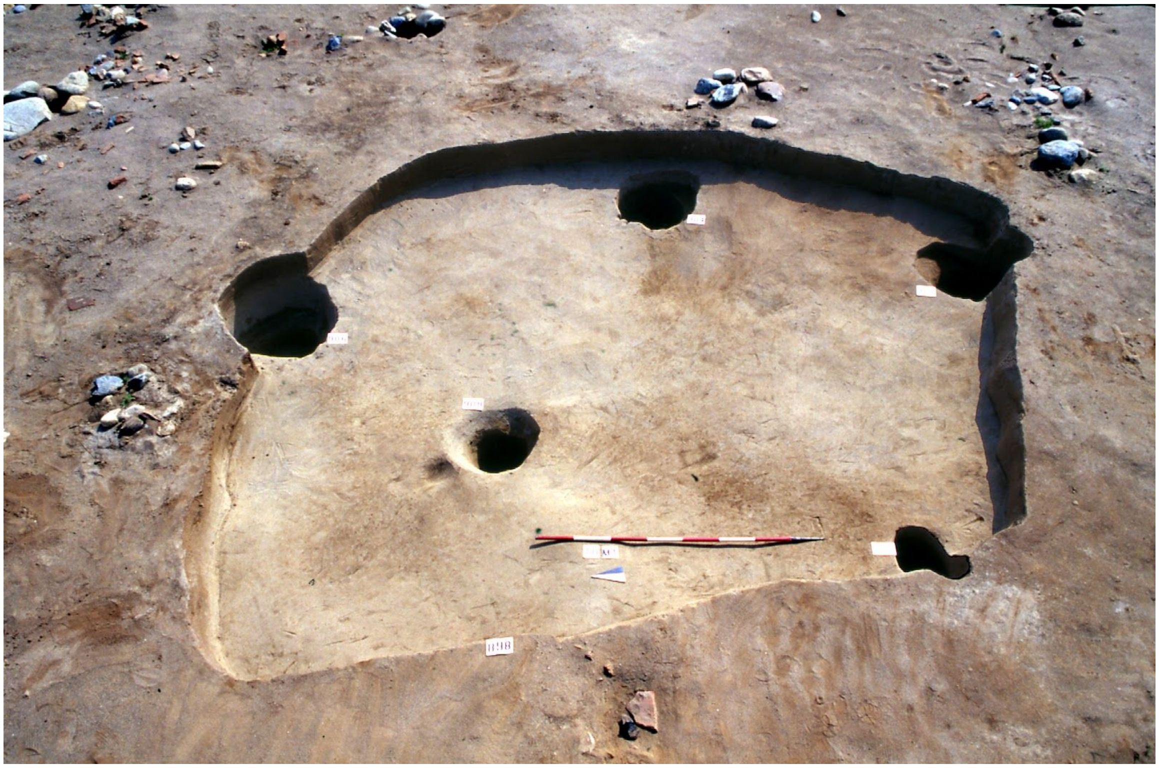

Supplementary Figure 14. A sunken-feature building at the Collegno settlement in the Longobard period. Image source: Soprintendenza Archeologia, Belle Arti e Paesaggio per la Città Metropolitana di Torino, archivio fotografico.

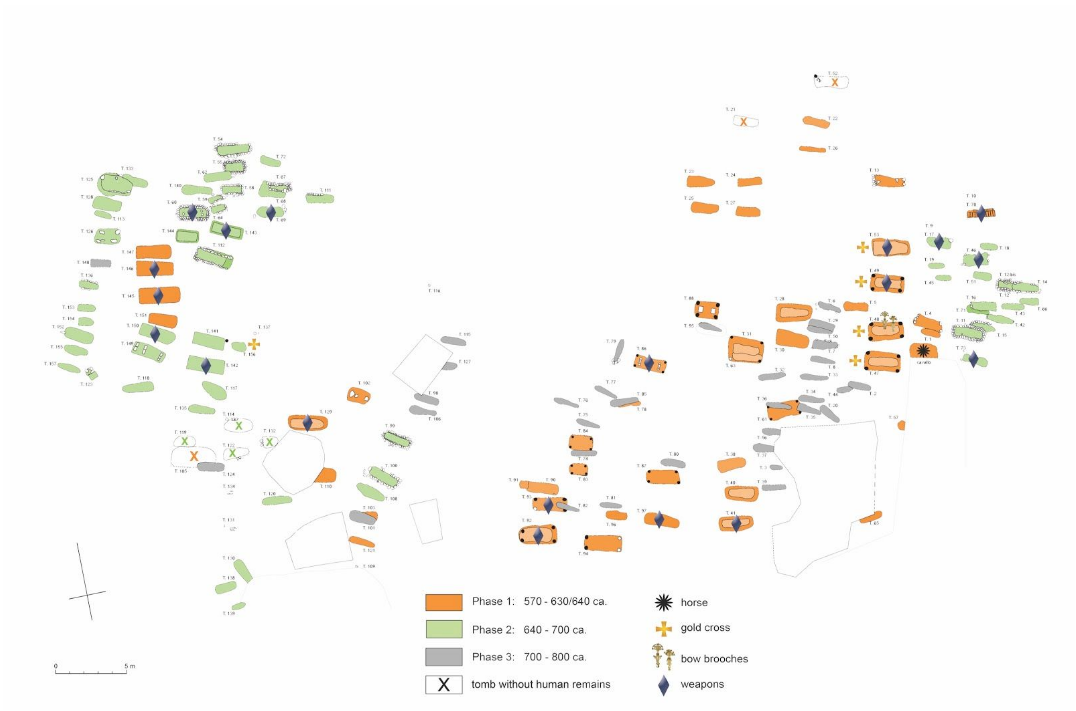

Supplementary Figure 15. Plan of the cemetery of Collegno with three major periods highlighted in colour.

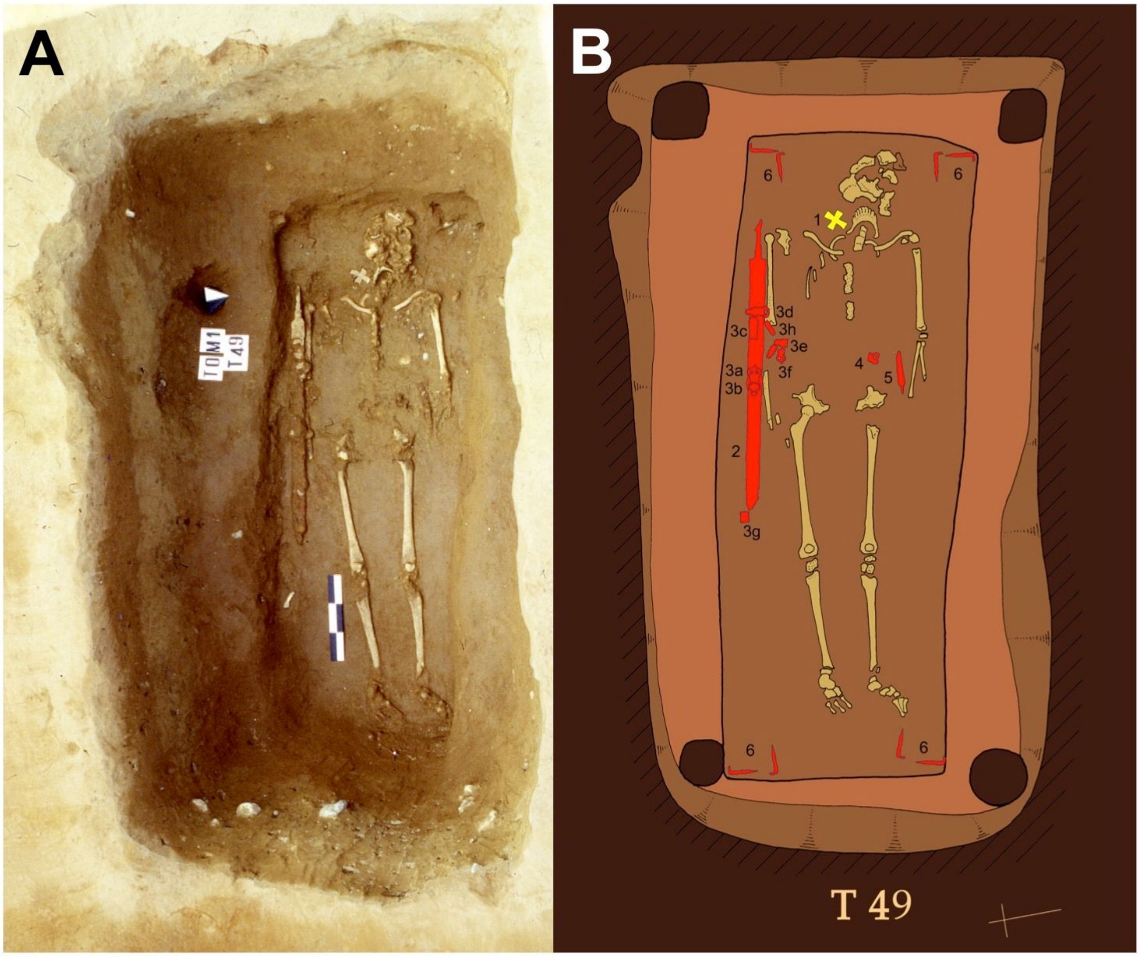

Supplementary Figure 16. Grave 49 at Collegno during the excavation (A) and plan of the grave (B). Image source: (A) Soprintendenza Archeologia, Belle Arti e Paesaggio per la Città Metropolitana di Torino, archivio fotografico; (B) current work.

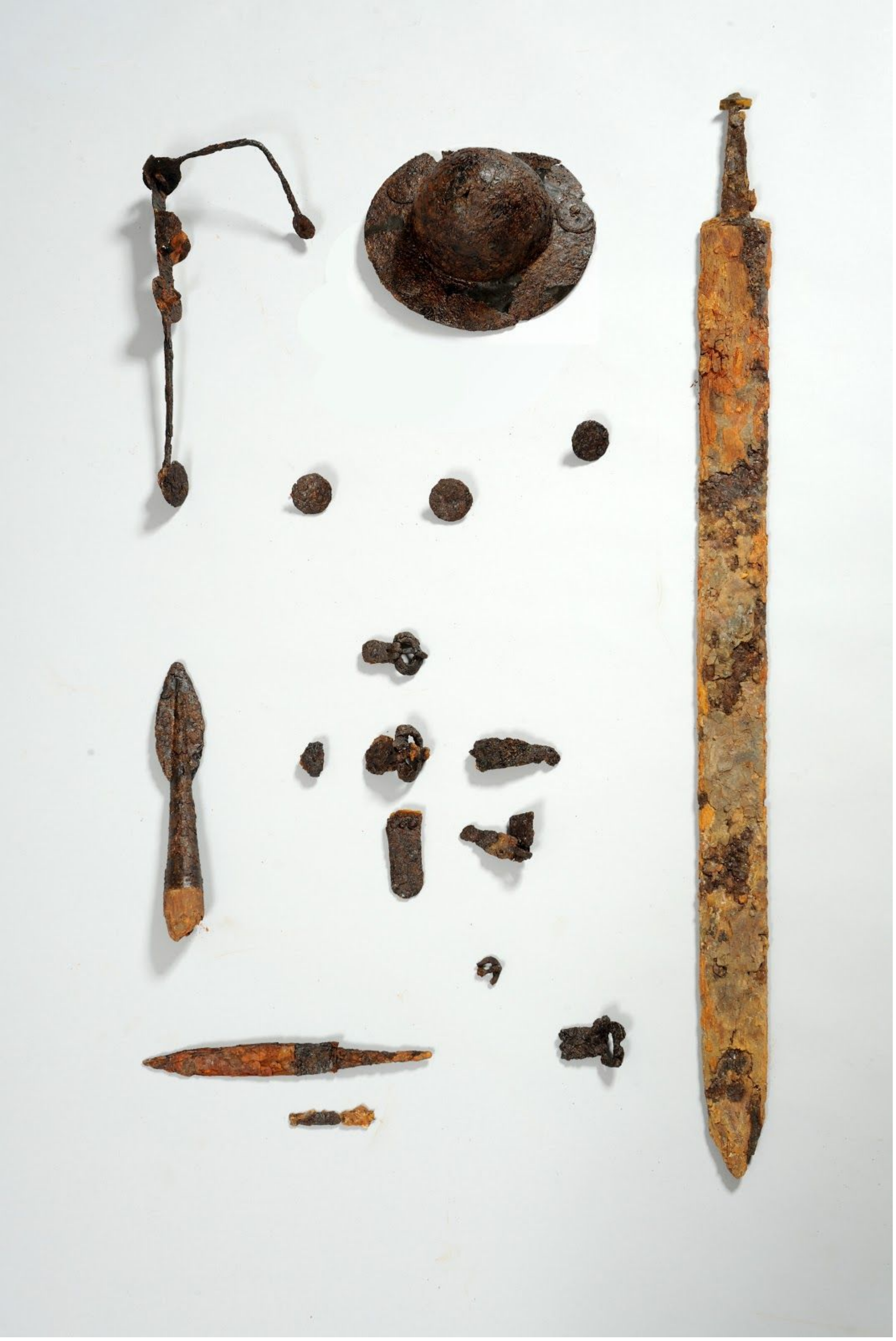

Supplementary Figure 17. The male grave goods for grave 97 (c. 580-600 CE) at Collegno. Image source: Soprintendenza Archeologia, Belle Arti e Paesaggio per la Città Metropolitana di Torino, archivio fotografico.

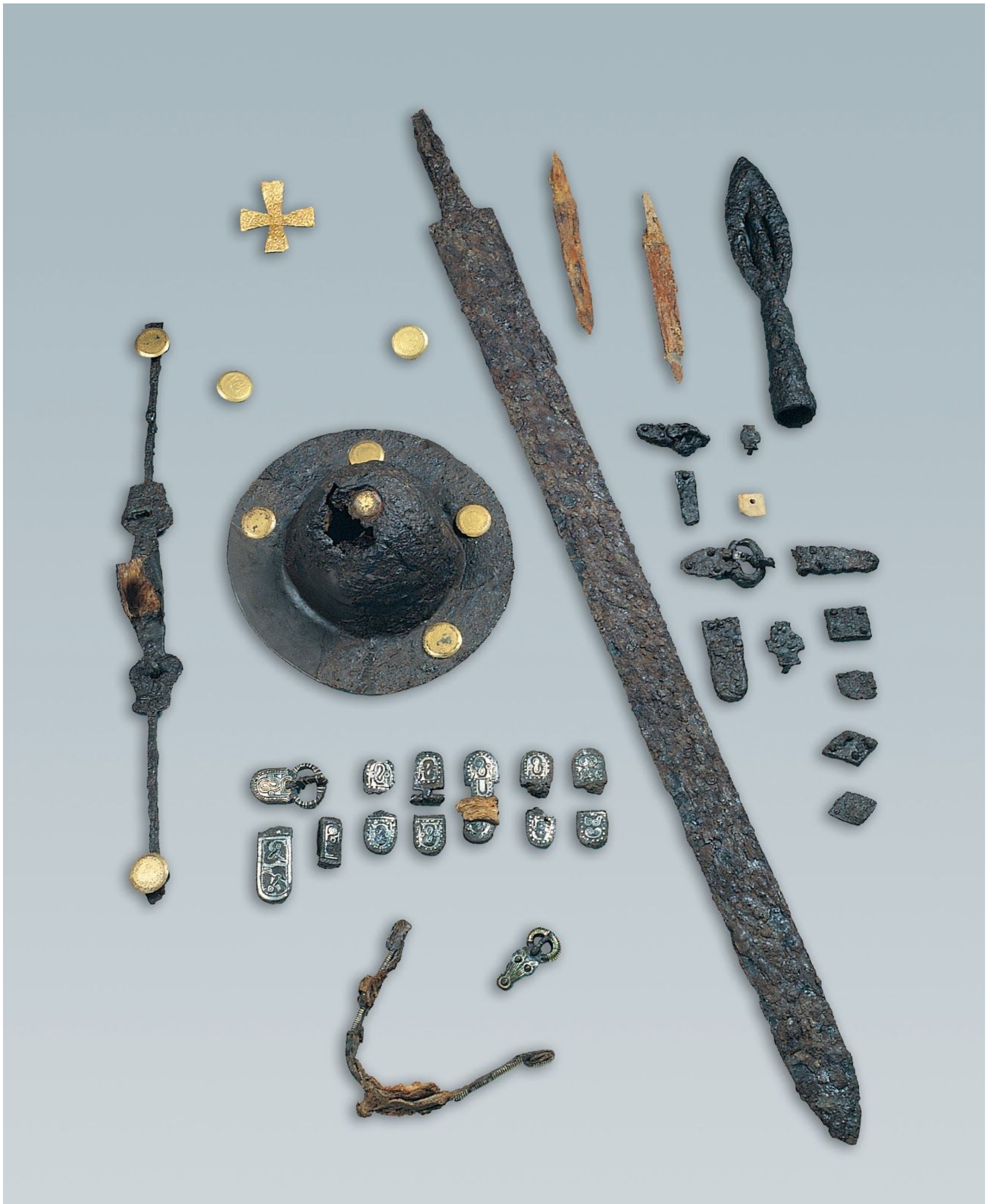

Supplementary Figure 18. The male grave goods for grave 53 (c. 600-630 CE) at Collegno. Image source: Soprintendenza Archeologia, Belle Arti e Paesaggio per la Città Metropolitana di Torino, archivio fotografico.

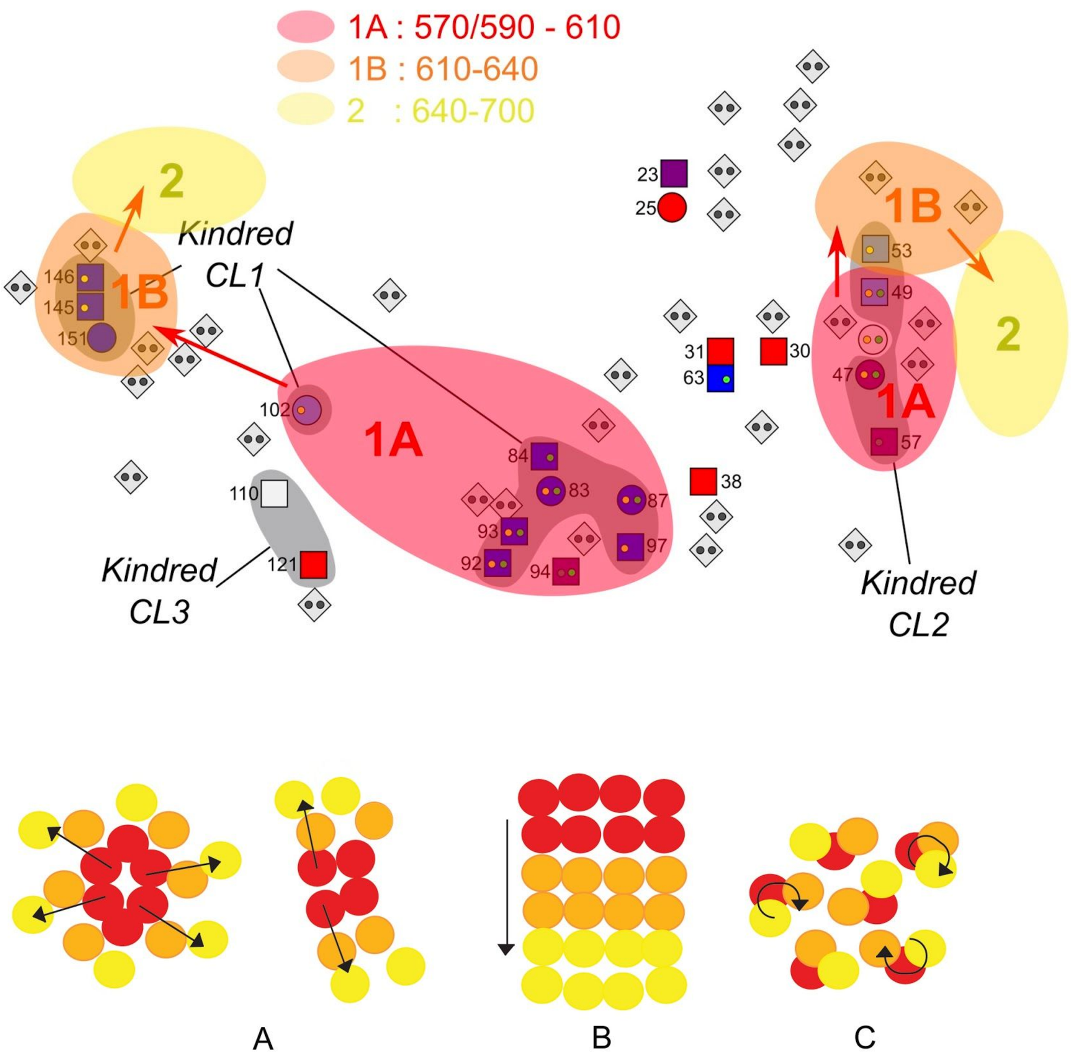

Supplementary Figure 19. The development of the cemetery at Collegno. The two main kindreds occupy a new sector after each generation. They move outwards: one to the west, the other to the east. For phase 1B we have fewer samples. The shift continues afterwards into second period where we currently lack genomic data. Compared to the two main kindred, the *S* individuals have marginal positions between the two main kindred groups. Bottom row shows possible developments of Longobard cemeteries in Italy. (red: phase 1, orange: phase 2: yellow phase 3; the circles are the kindred nucleus)[155].

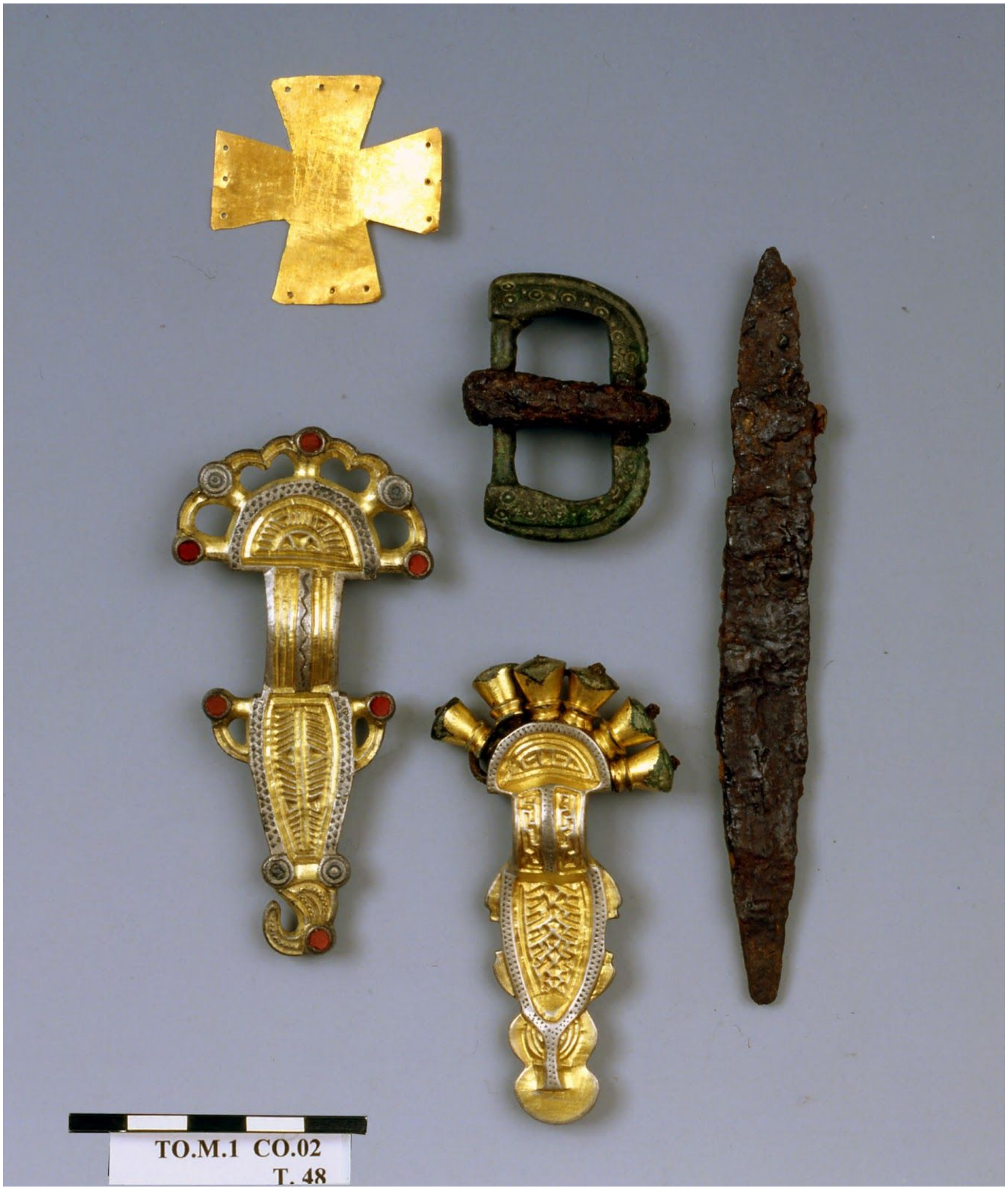

Supplementary Figure 20: Female grave goods for grave 48, with bow brooch (on the left) and buckle produced north of the Alps at Collegno. Image source: Soprintendenza Archeologia, Belle Arti e Paesaggio per la Città Metropolitana di Torino, archivio fotografico.

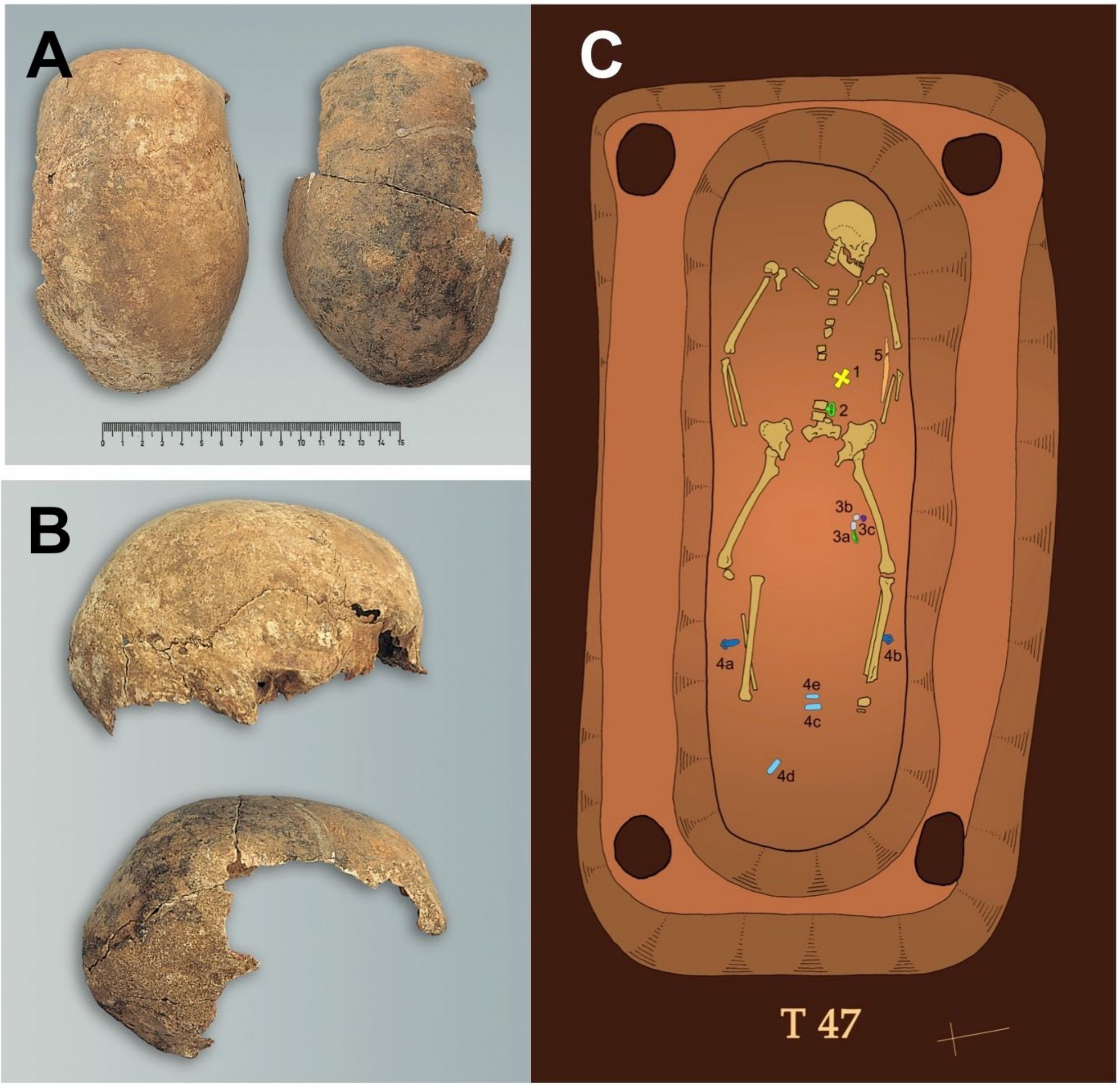

Supplementary Figure 21. Superior and right lateral view of the scaphocephalic female skulls (A) CL47 and (B) CL48 (left and below), and (C) the plan of the grave for CL47 at Collegno. Image source: Soprintendenza Archeologia, Belle Arti e Paesaggio per la Città Metropolitana di Torino, archivio fotografico.

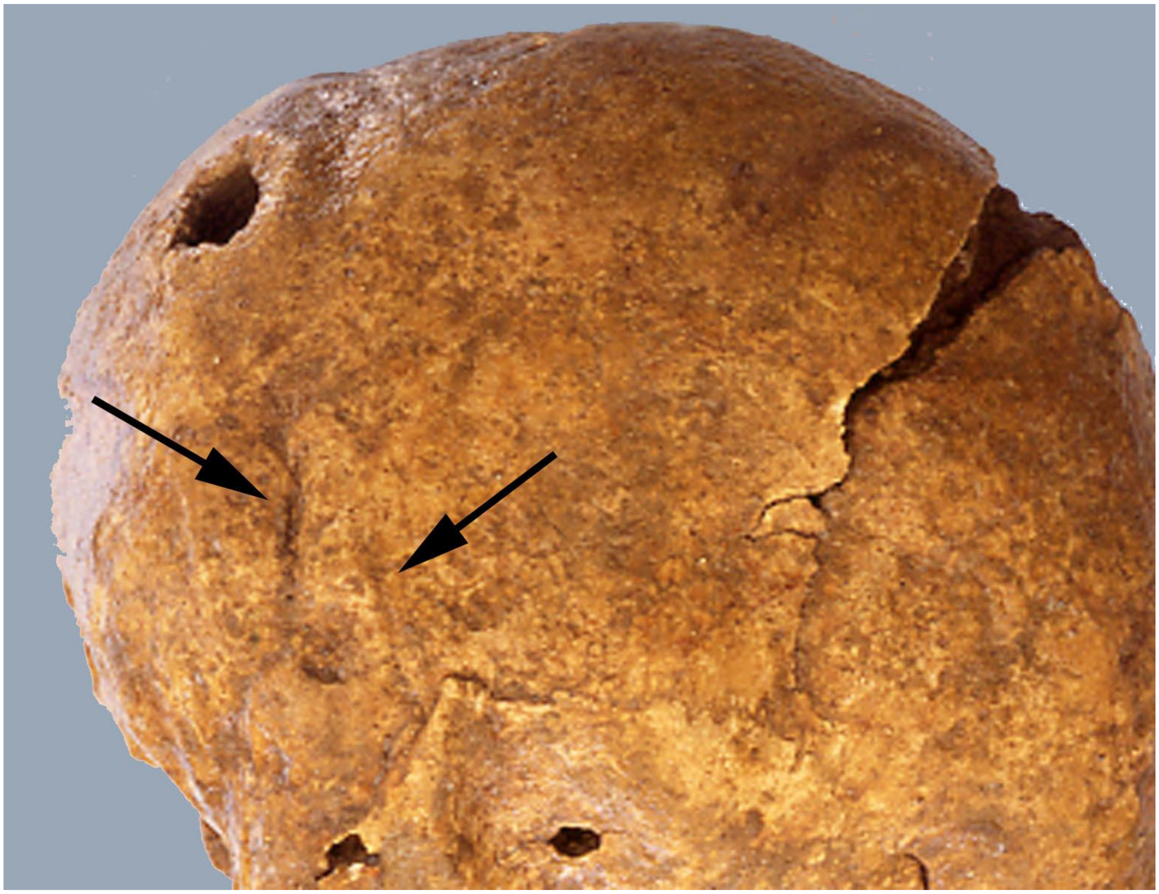

Supplementary Figure 22. Grave 70 at Collegno, an elderly male with two ante mortem cranial wounds caused by an edged weapon on the right temporo-occipital region, and peri mortem wound on the right half of the lambdoid suture caused by a pointed weapon. Image source: Soprintendenza Archeologia, Belle Arti e Paesaggio per la Città Metropolitana di Torino, archivio fotografico.

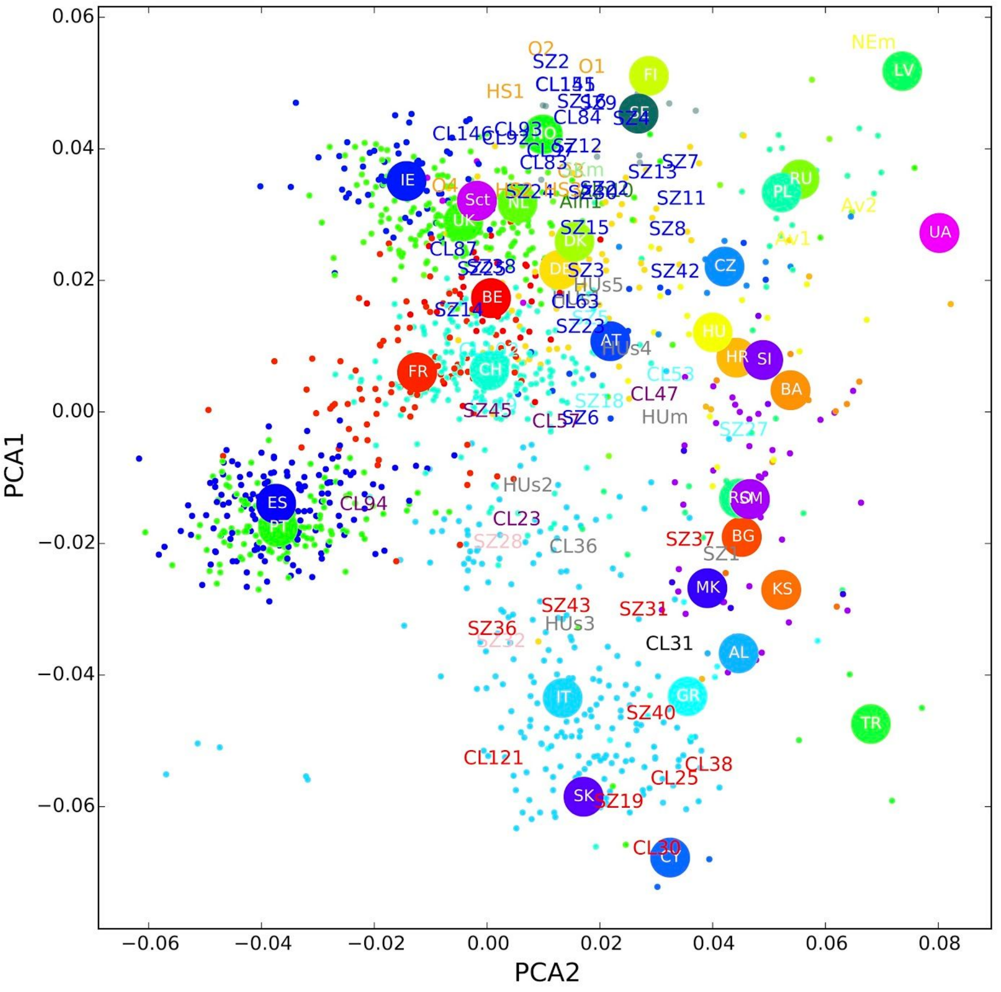

Supplementary Figure 23. Procrustes transformed PCA of medieval ancient samples against POPRES imputed SNP dataset. Color coding of medieval samples is same as in Figs 1 and 2. Two letter and three codes for POPRES samples: AL=Albania, AT=Austria, BA=Bosnia-Herzegovina, BE=Belgium, BG=Bulgaria, CH=Switzerland, CY=Cyprus, CZ=Czech Republic, DE=Germany, DK=Denmark, ES=Spain, FI=Finland, FR=France, GB=United Kingdom, GR, Greece, HR=Croatia, HU=Hungary, IE=Ireland, IT=Italy, KS=Kosovo, LV=Latvia, MK=Macedonia, NO=Norway, NL=Netherlands, PL=Poland, PT=Portugal, RO=Romania, SM=Serbia and Montenegro, RU=Russia, Sct=Scotland, SE=Sweden, SI=Slovenia, SK=Slovakia, TR=Turkey, UA=Ukraine.

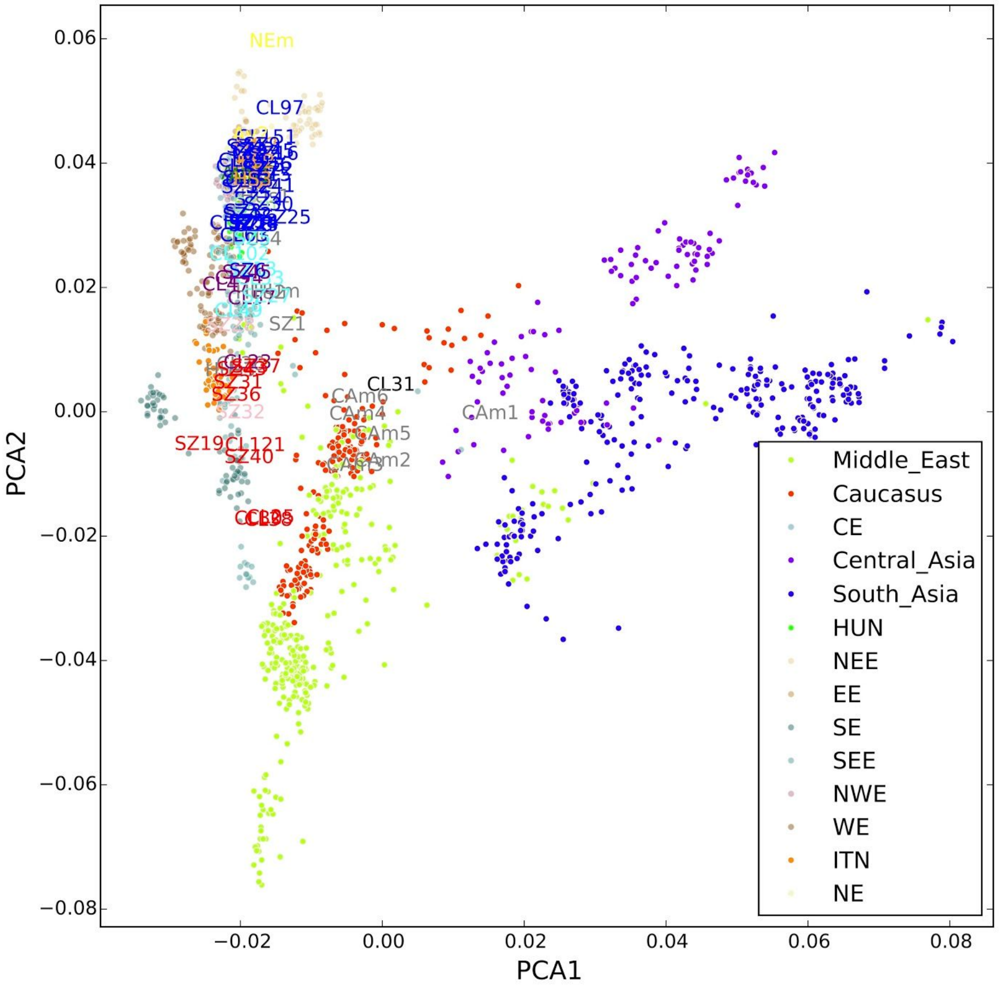

Supplementary Figure 24. Procrustes transformed PCA of medieval samples against HellBus imputed SNP dataset. Color coding of medieval samples is same as in Figs 1 and 2 (in addition to Caucasus medieval samples).

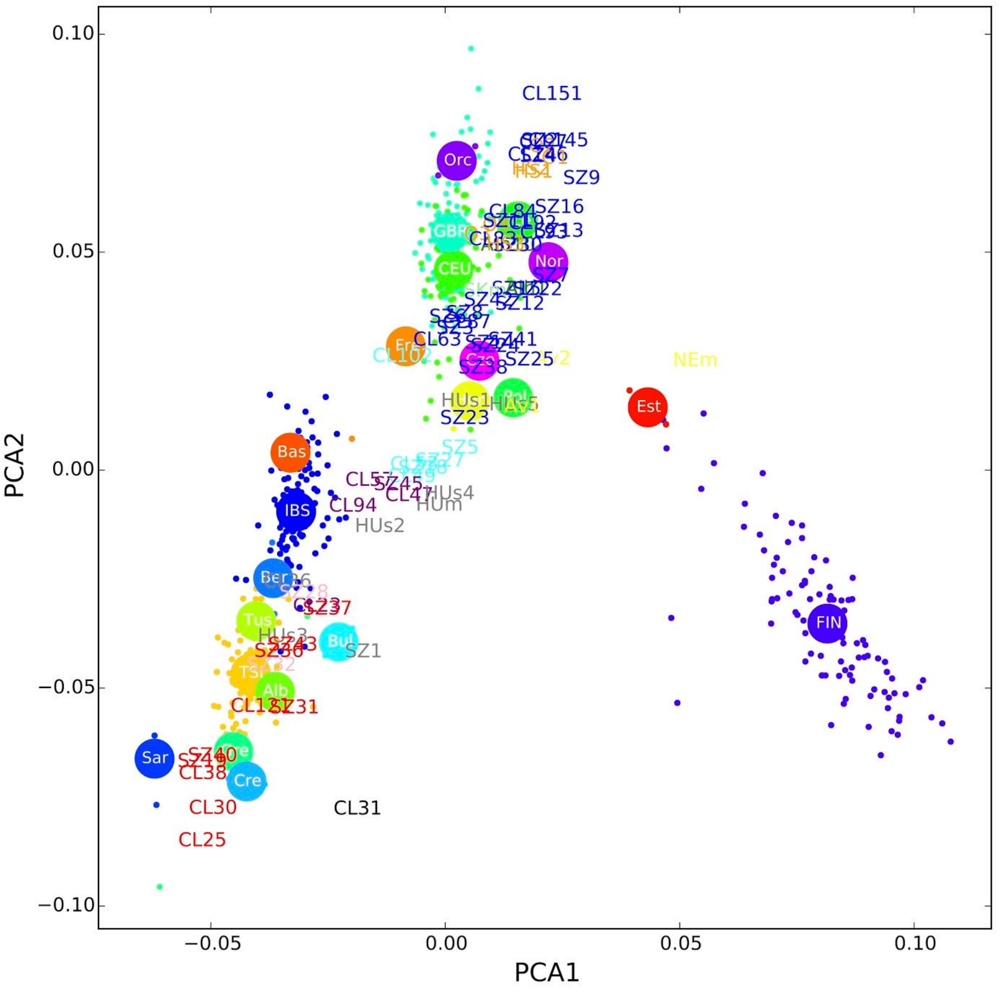

Supplementary Figure 25. Procrustes transformed PCA of medieval samples against 1000 Genomes + SGDP European samples. Color coding of medieval samples is same as in Figs 1 and 2. Three letter codes: Alb = Albania, Bas=Basque, Ber=Bergamo,Bul=Bulgaria, CEU=Northern and Western European Ancestry from 1000 Genomes , Cre = Crete, Cze=Czech Republic, Est=Estonia, Fre=France, FIN=Finland from 1000 Genomes, GBR=Britain from 1000 Genomes, Gre=Greece, Hun=Hungary, Ice=Iceland, IBS=Iberia from Spain 1000 Genomes, Nor=Norway, Orc=Orcadians, Pol=Poland, Sar=Sardinian, Spa=Spain, Tus=Tuscan, TSI=Tuscany from 1000 Genomes.

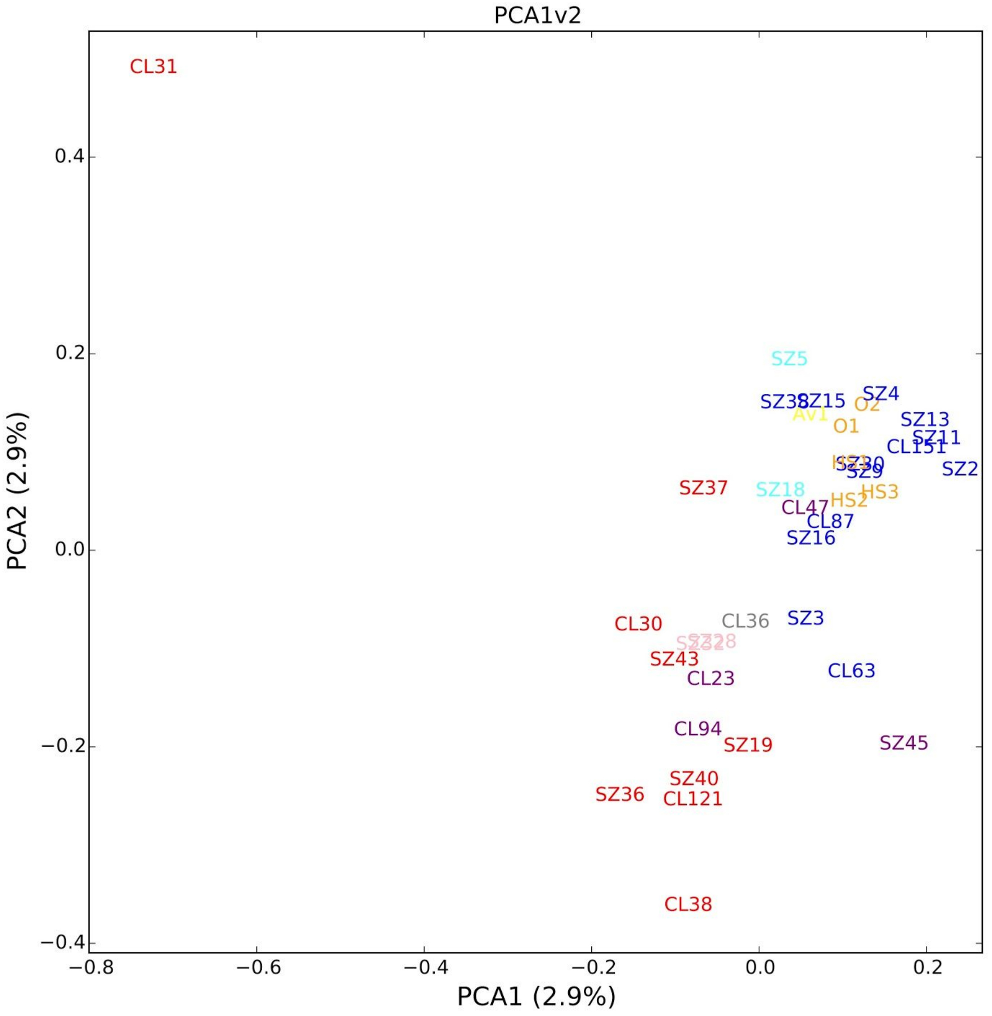

Supplementary Figure 26. PCA of unrelated medieval samples with no missing SNP positions amongst samples. Colour coding of medieval samples is same as in Figs 1 and 2.

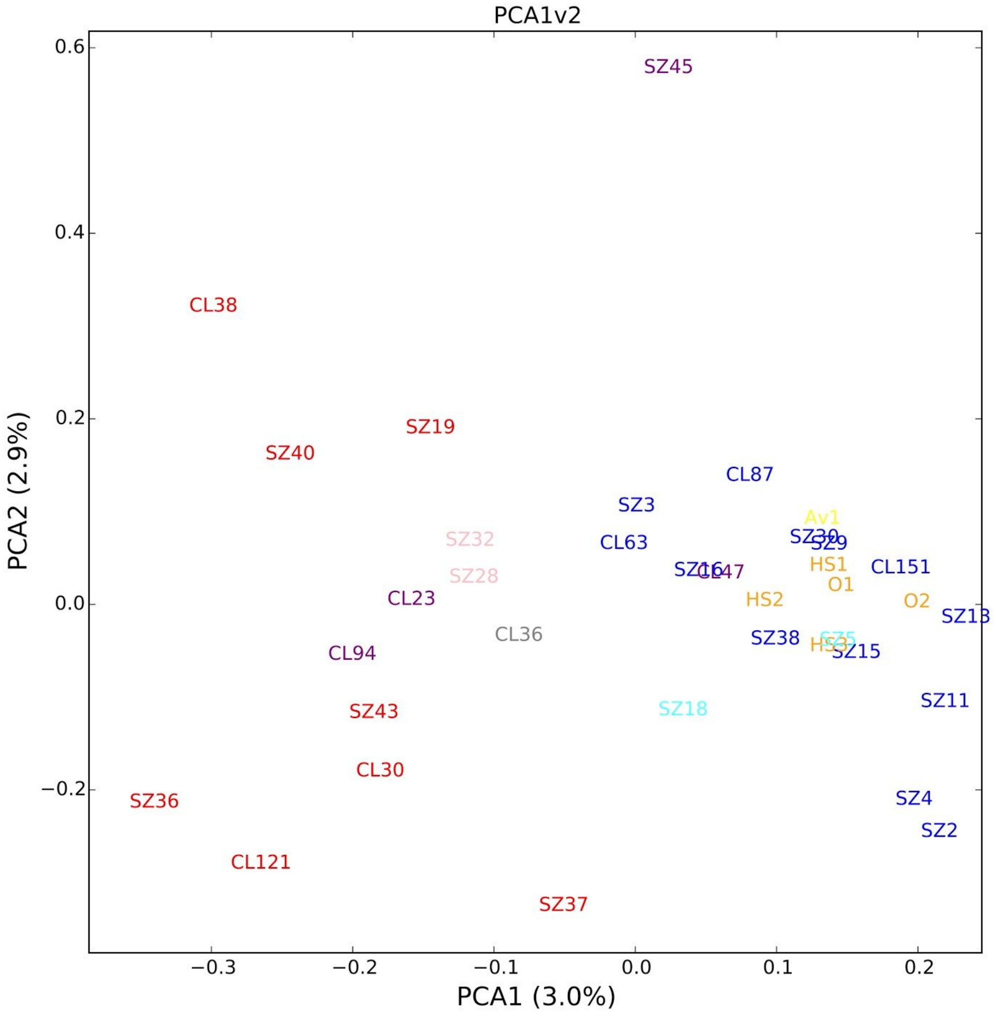

Supplementary Figure 27. PCA of unrelated medieval samples, excluding CL31, with no missing SNP positions amongst samples. Colour coding of medieval samples is same as in Figs 1 and 2.

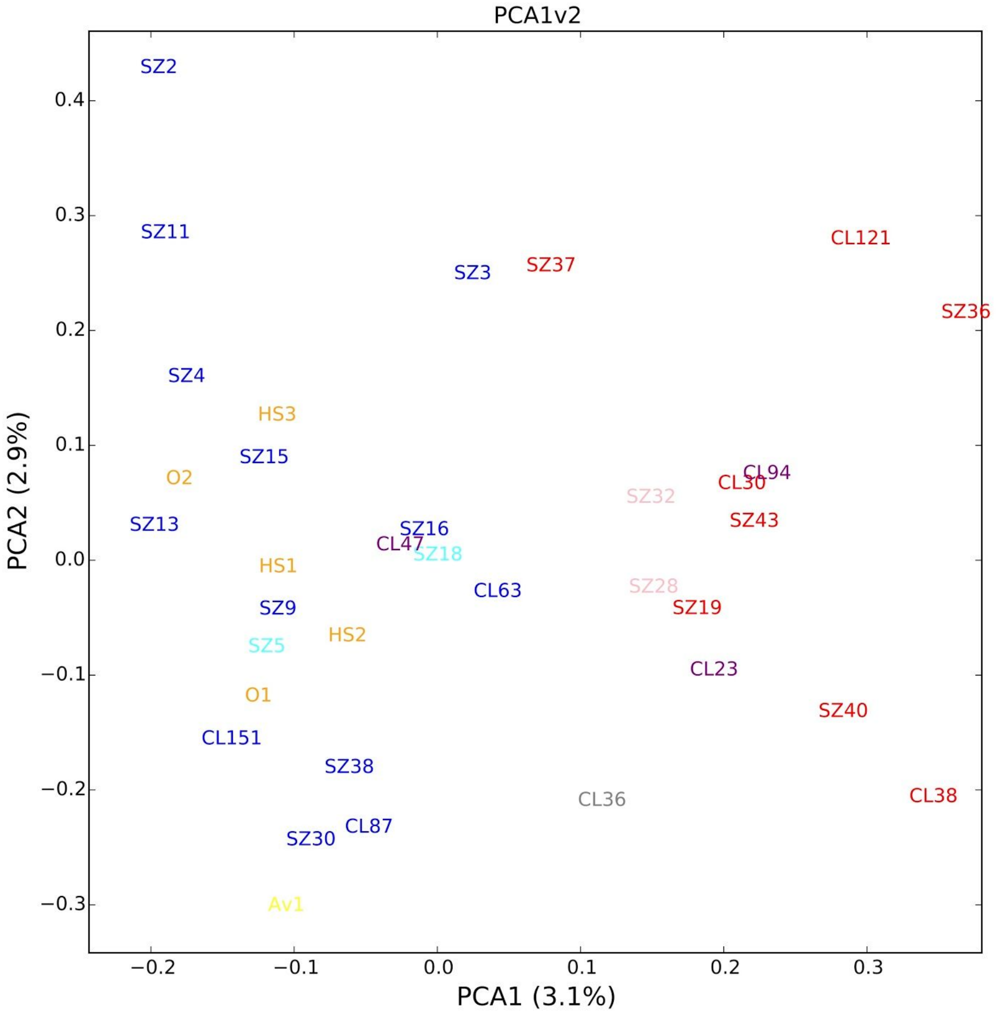

Supplementary Figure 28. PCA of unrelated medieval samples, excluding CL31 and SZ45, with no missing SNP positions amongst samples. Colour coding of medieval samples is same as in Figs 1 and 2.

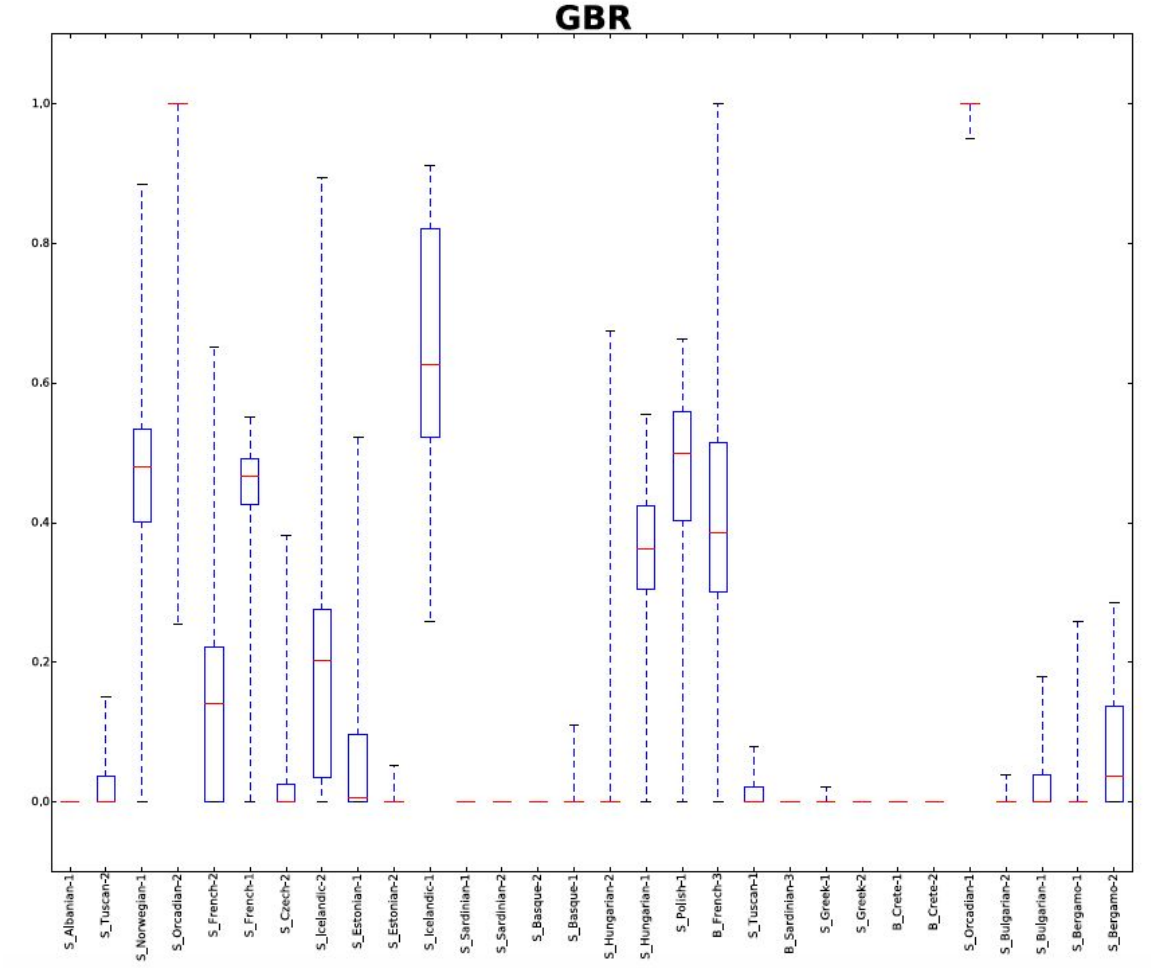

Supplementary Figure 29. Boxplot of GBR Ancestry coefficients for SGDP European samples analyzed alongside individual medieval samples.

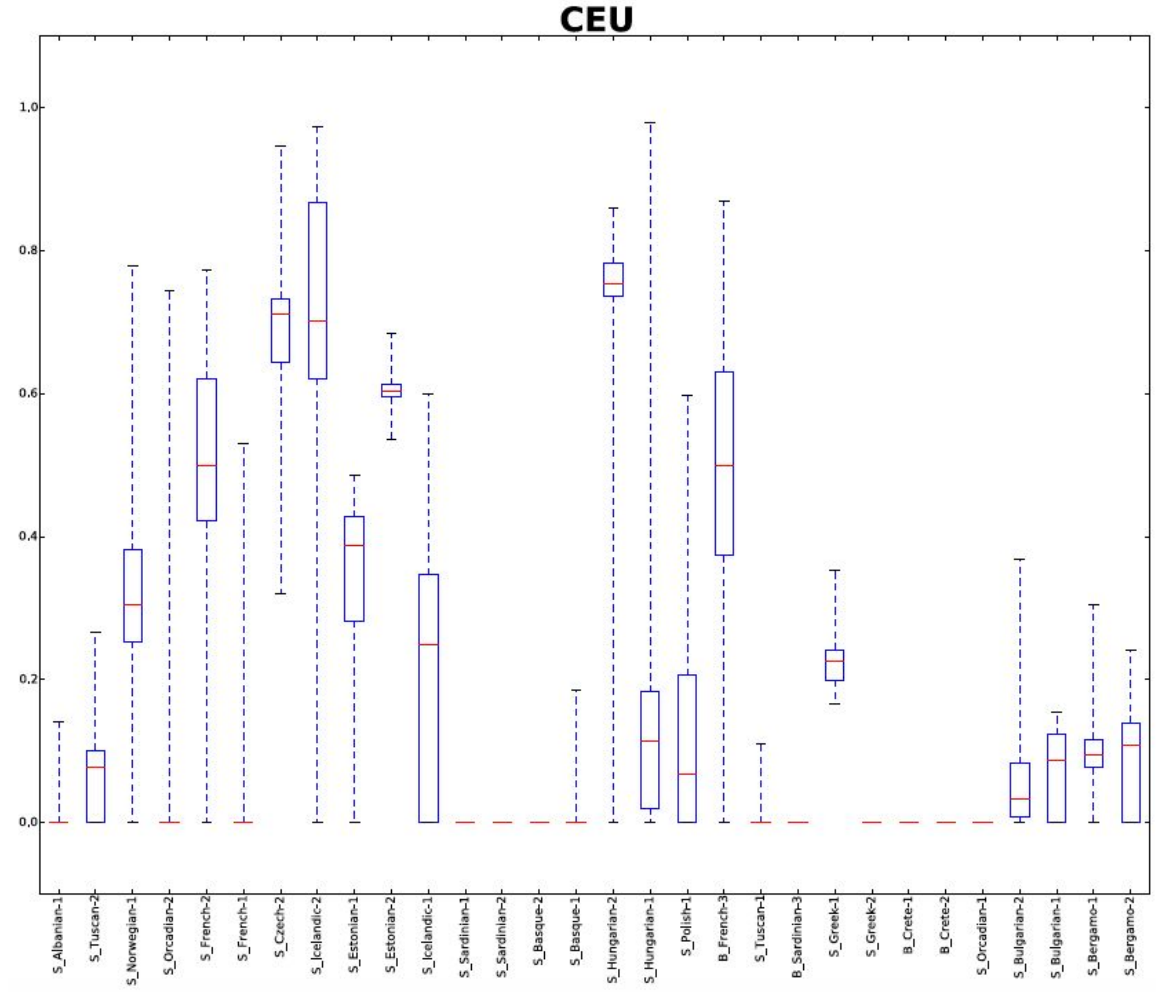

Supplementary Figure 30. Boxplot of CEU Ancestry coefficients for SGDP European samples analyzed alongside individual medieval samples.

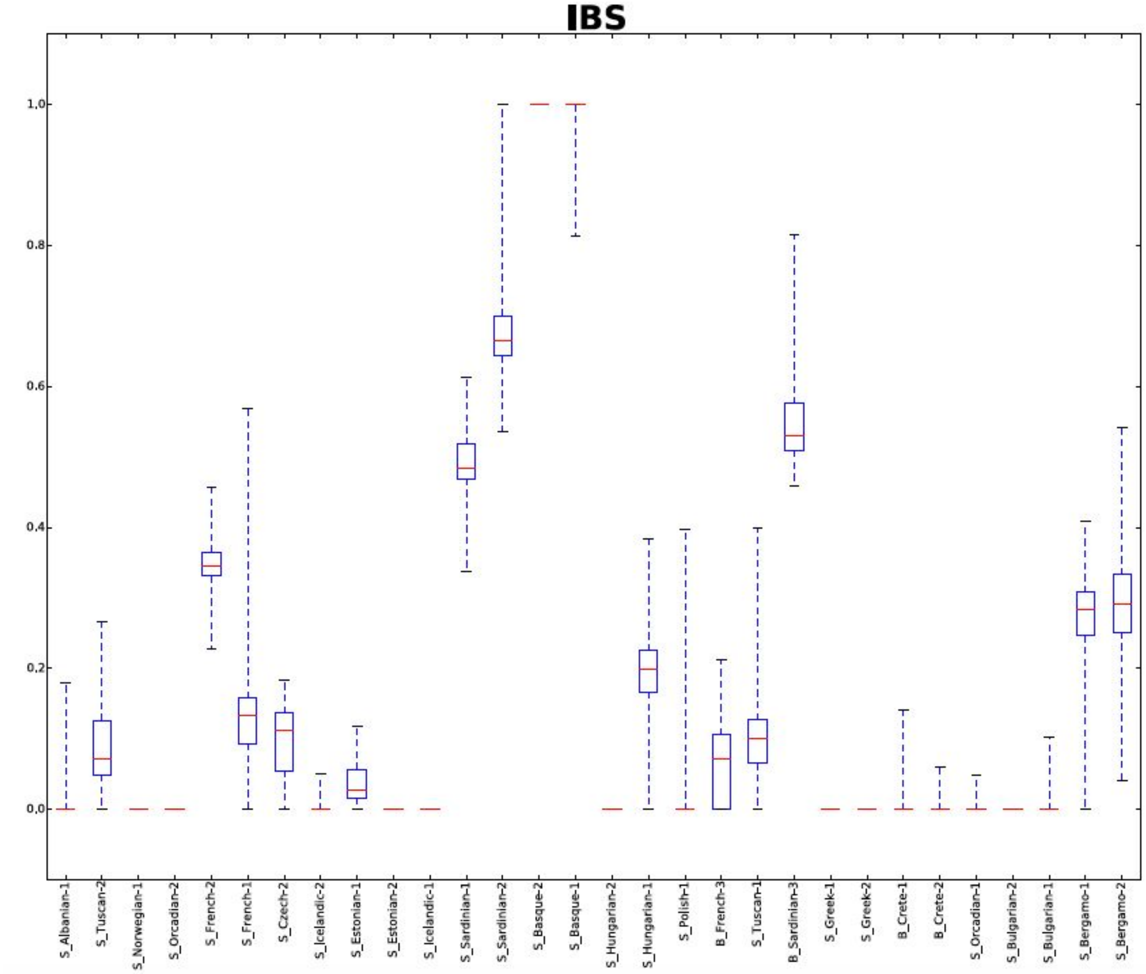

Supplementary Figure 31. Boxplot of IBS Ancestry coefficients for SGDP European samples analyzed alongside individual medieval samples.

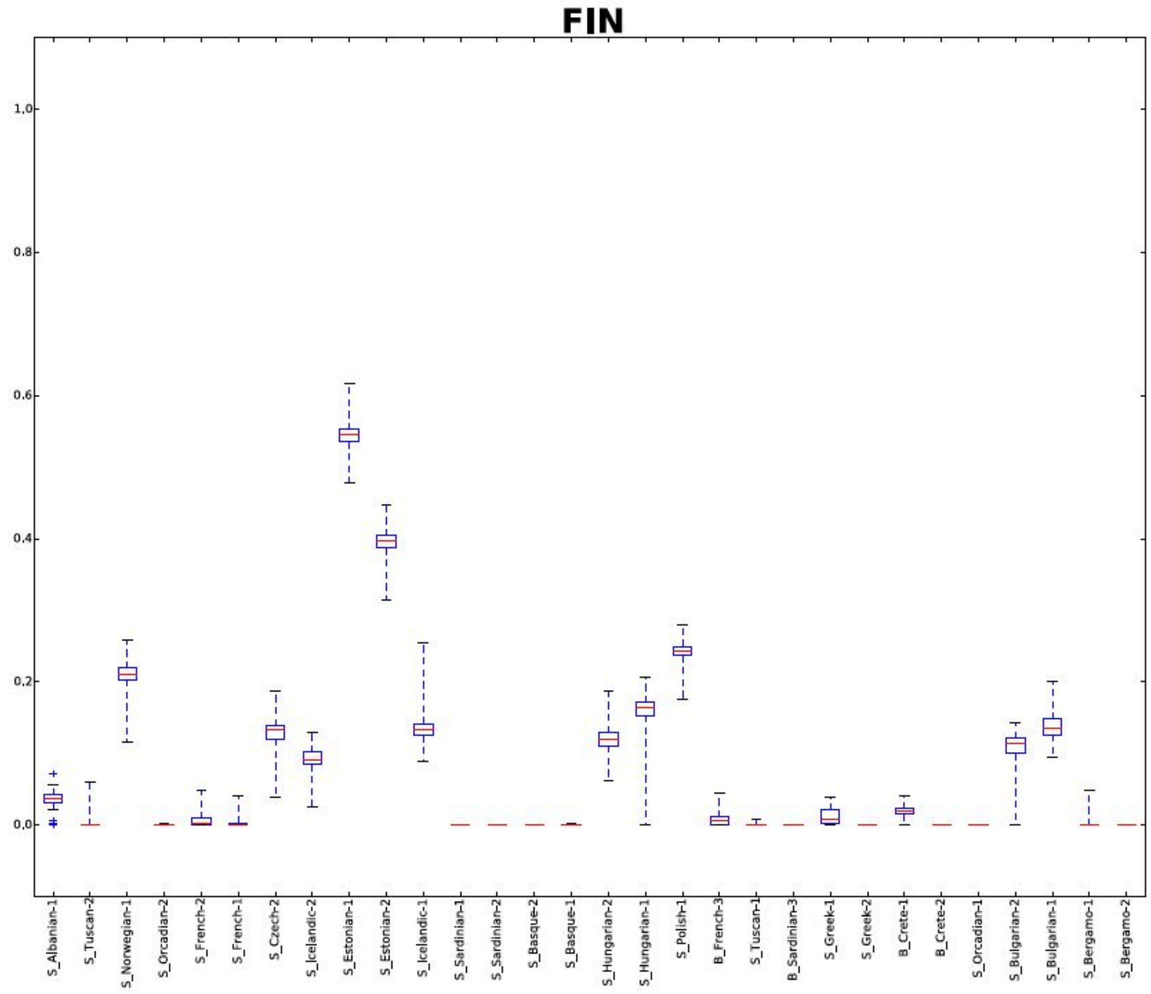

Supplementary Figure 32. Boxplot of FIN Ancestry coefficients for SGDP European samples analyzed alongside individual medieval samples.

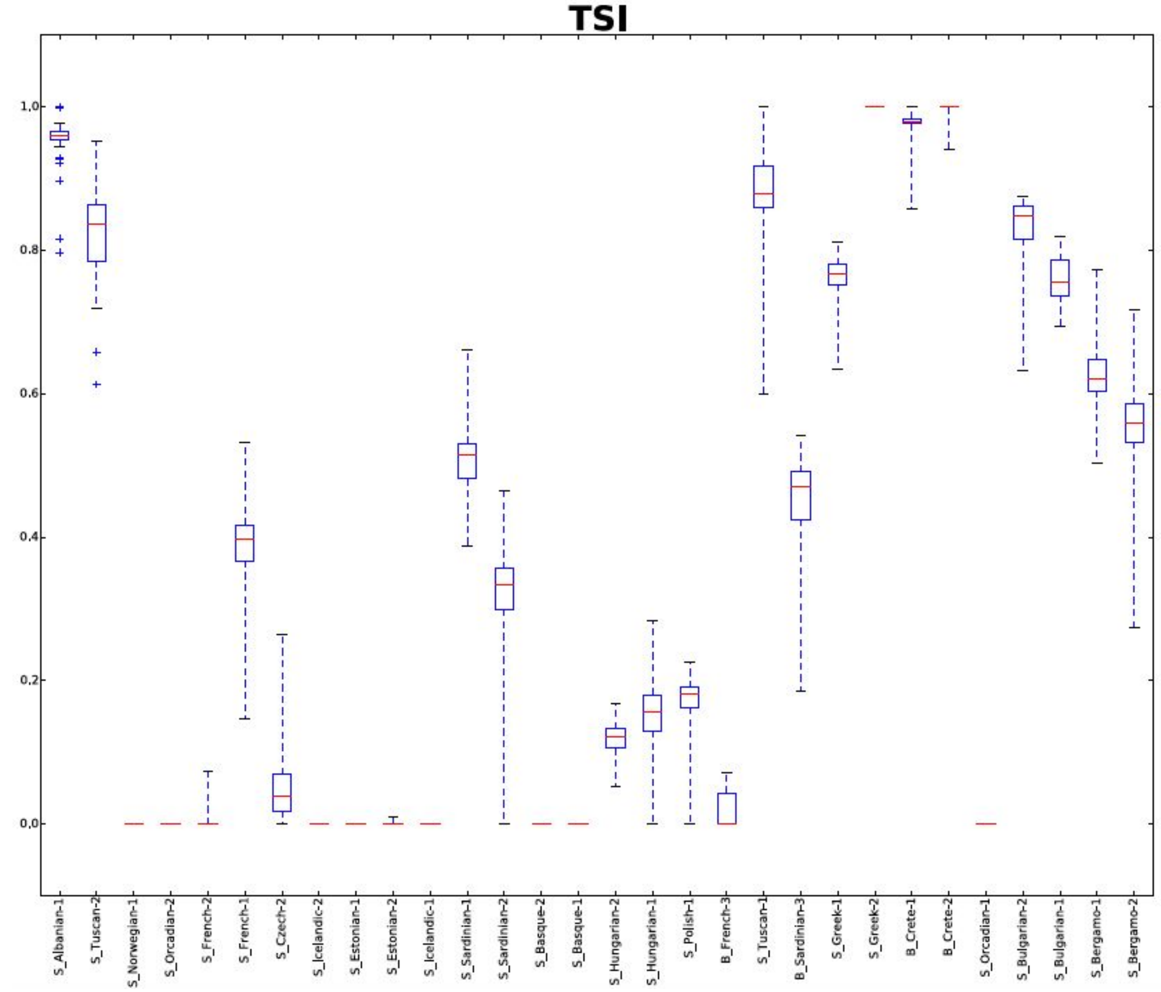

Supplementary Figure 33. Boxplot of TSI Ancestry coefficients for SGDP European samples analyzed alongside individual medieval samples.

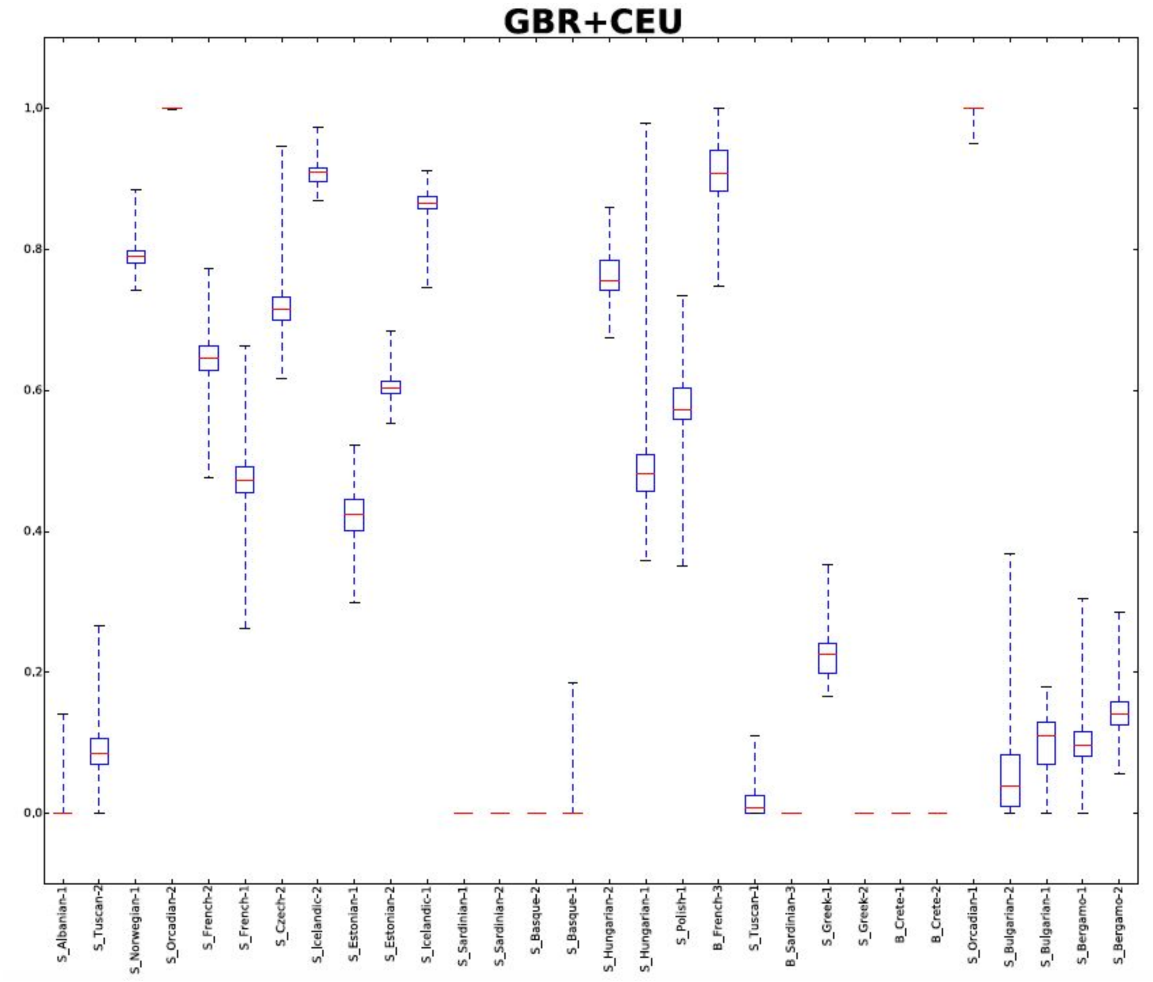

Supplementary Figure 34. Boxplot of GBR+CEU Ancestry coefficients for SGDP European samples analyzed alongside individual medieval samples.

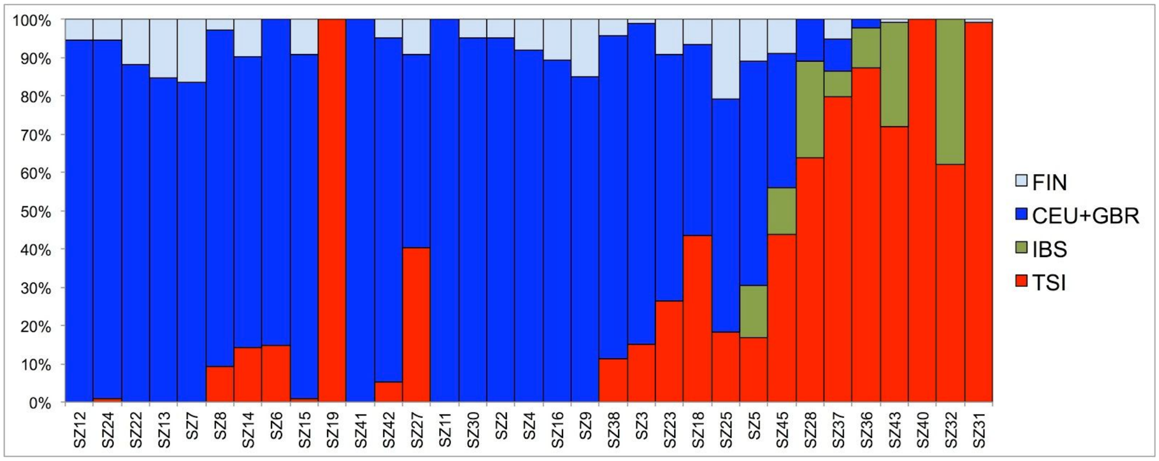

Supplementary Figure 35. Supervised ADMIXTURE ancestry estimation for K=5 for Szólád sixth century samples.

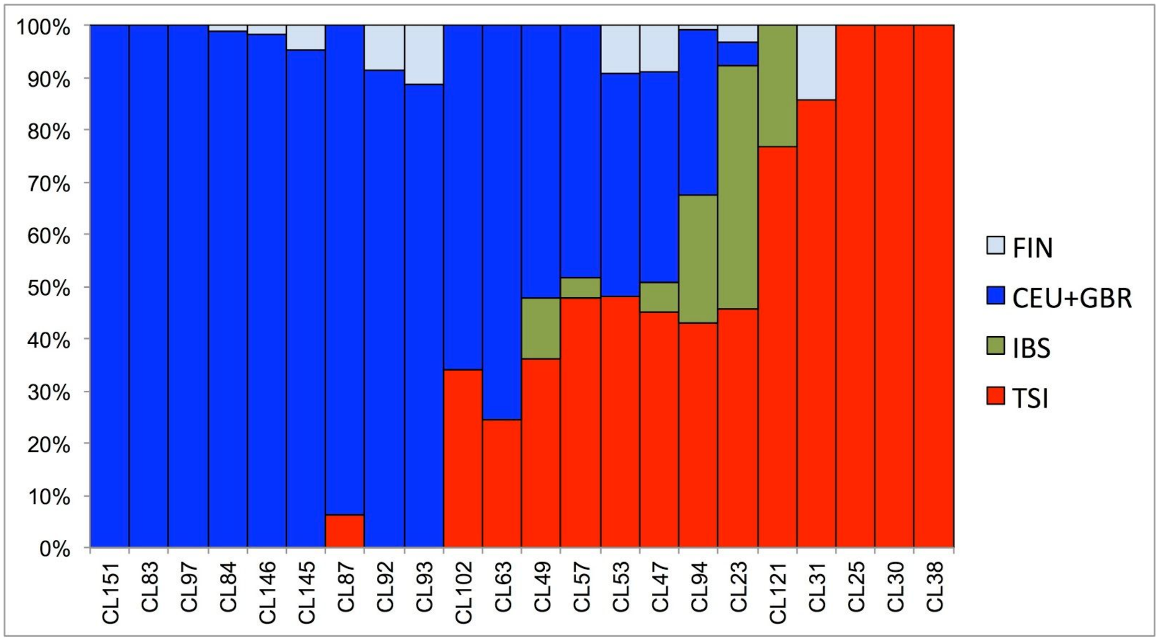

Supplementary Figure 36. Supervised ADMIXTURE ancestry estimation for K=5 for Collegno first period samples.

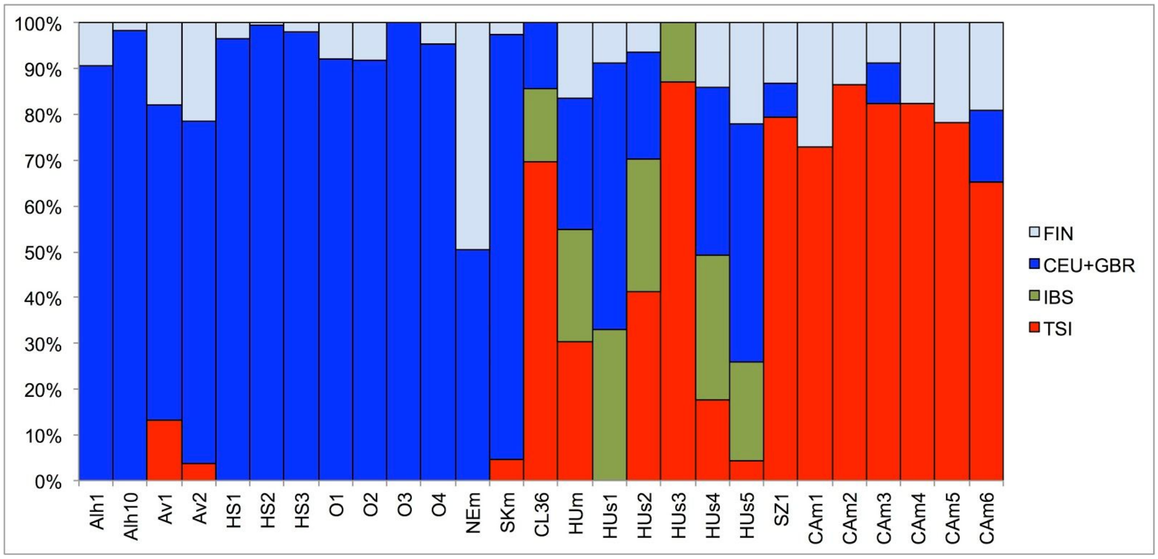

Supplementary Figure 37. Supervised ADMIXTURE ancestry estimation for K=5 for other medieval samples and SZ1.

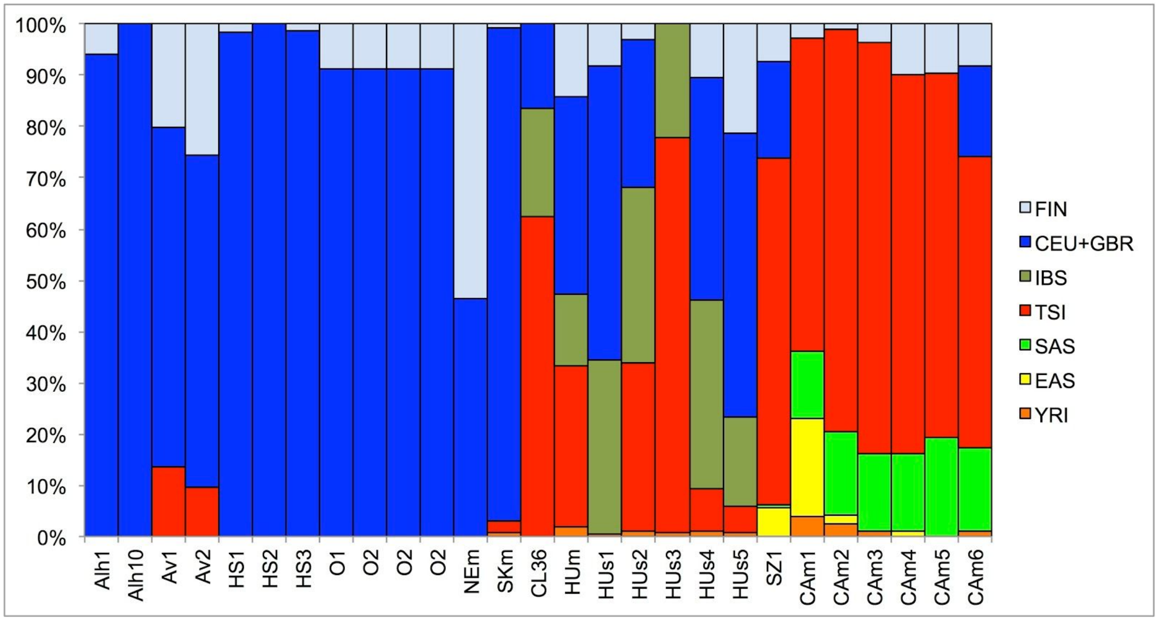

Supplementary Figure 38. Supervised ADMIXTURE ancestry estimation for K=8 for other medieval samples and SZ1.

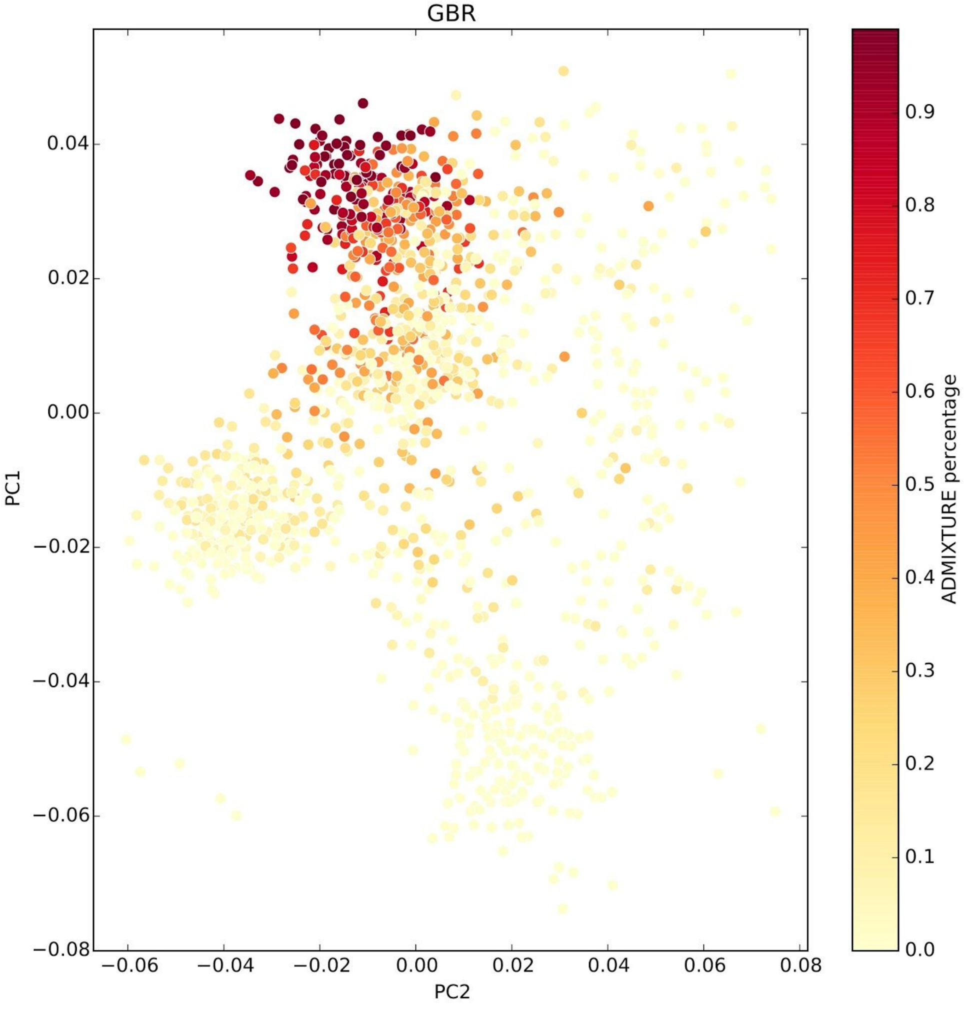

Supplementary Figure 39. Supervised ADMIXTURE estimates for GBR when K=5 for POPRES samples overlaid on PCA.

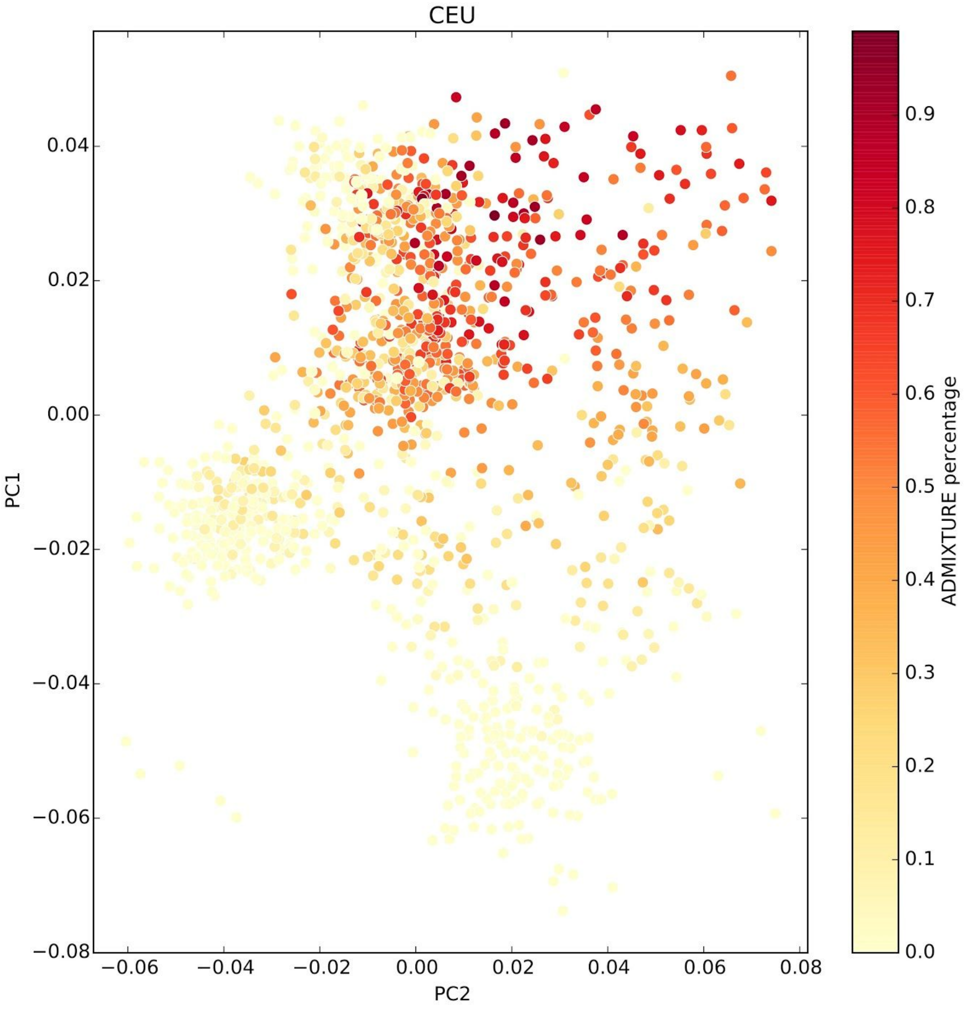

Supplementary Figure 40. Supervised ADMIXTURE estimates for CEU when K=5 for POPRES samples overlaid on PCA.

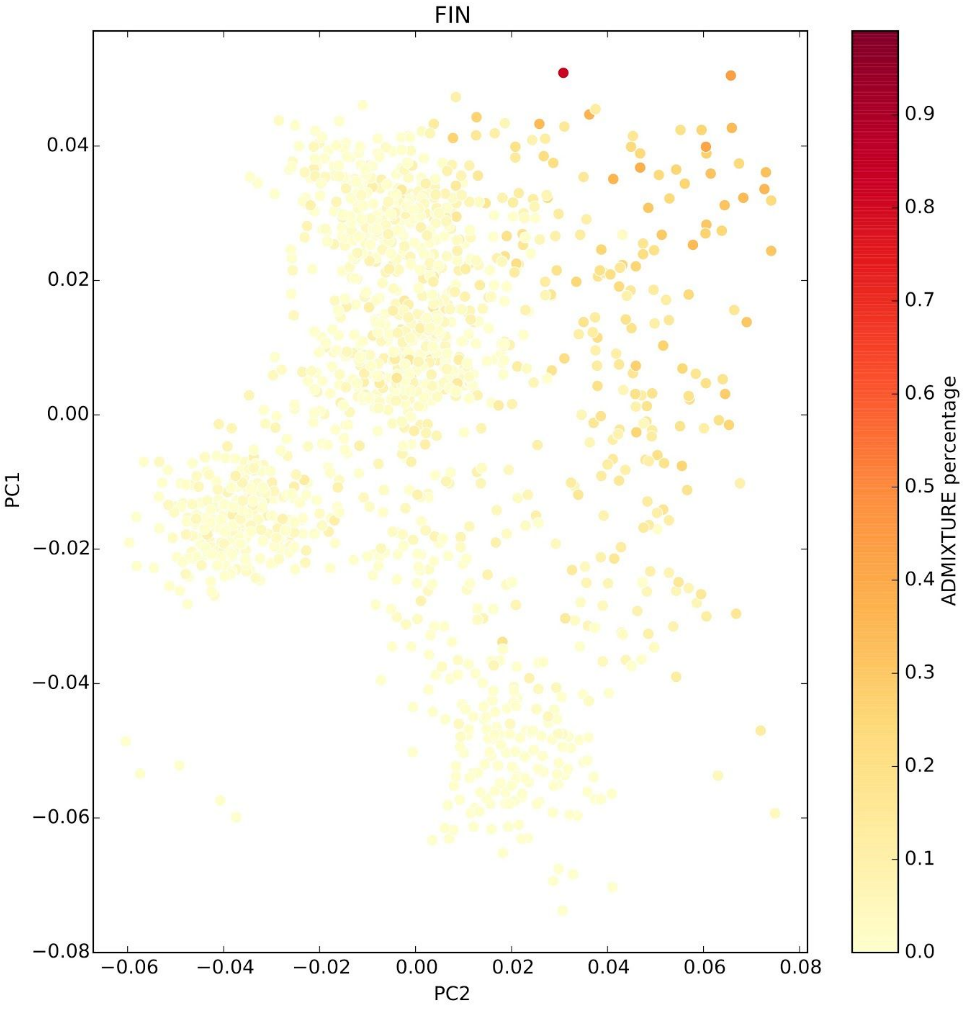

Supplementary Figure 41. Supervised ADMIXTURE estimates for FIN when K=5 for POPRES samples overlaid on PCA.

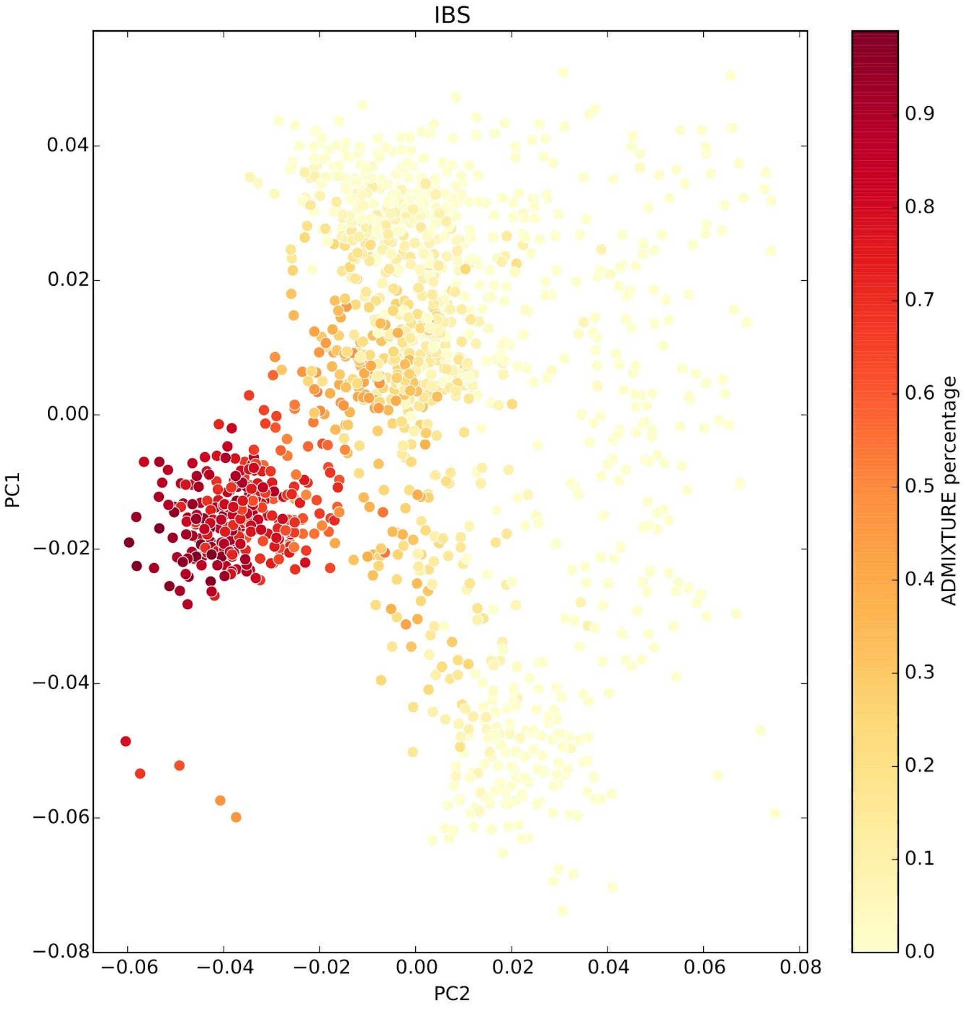

Supplementary Figure 42. Supervised ADMIXTURE estimates for IBS when K=5 for POPRES samples overlaid on PCA.

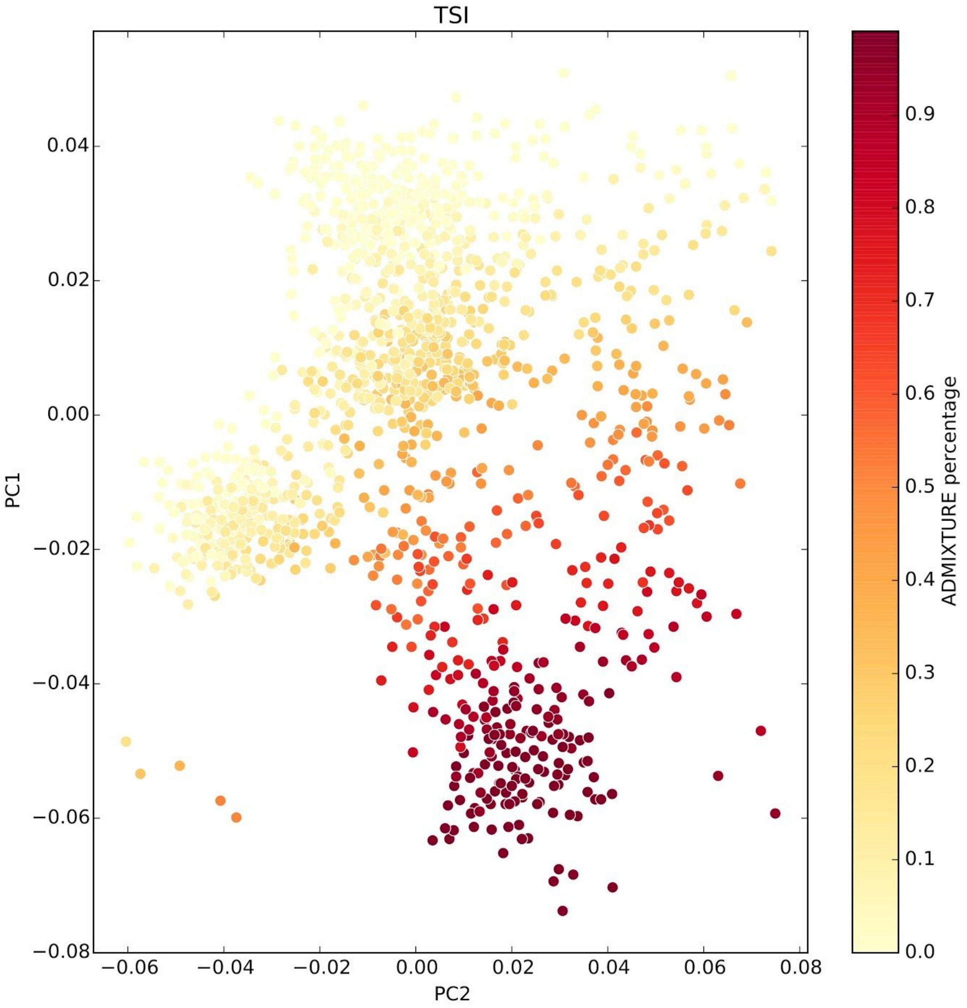

Supplementary Figure 43. Supervised ADMIXTURE estimates for TSI when K=5 for POPRES samples overlaid on PCA.

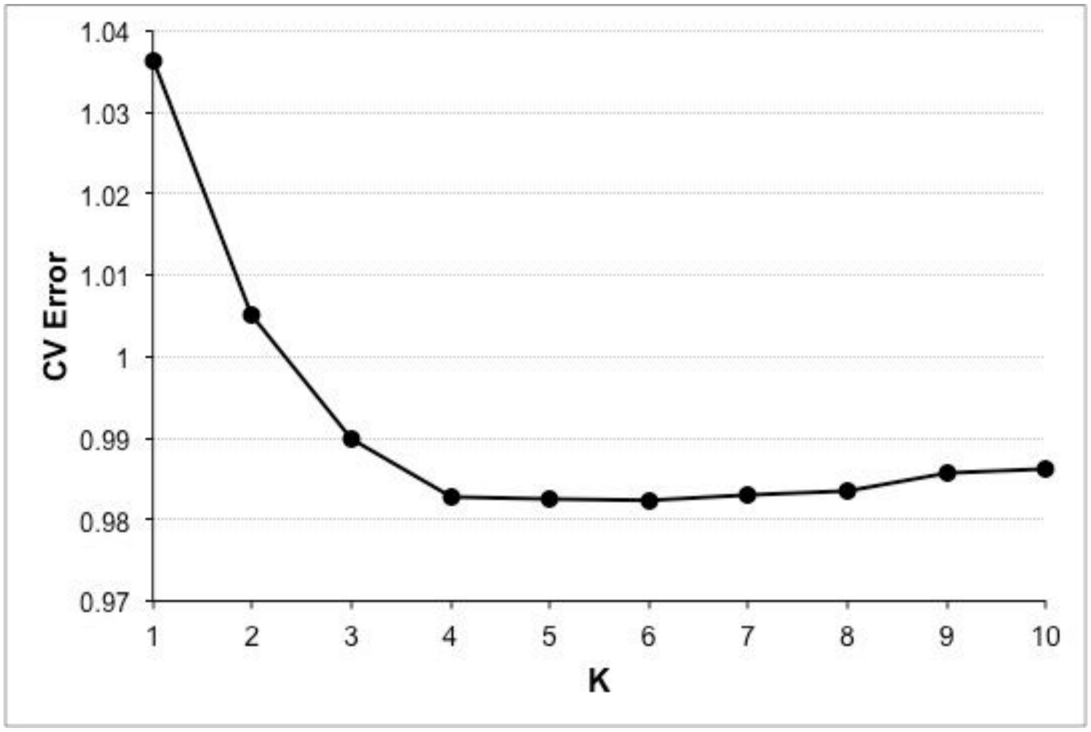

Supplementary Figure 44. Five-fold cross-validation error for *K*=1 - 10 for medieval and 1000 Genomes Eurasian+YRI samples.

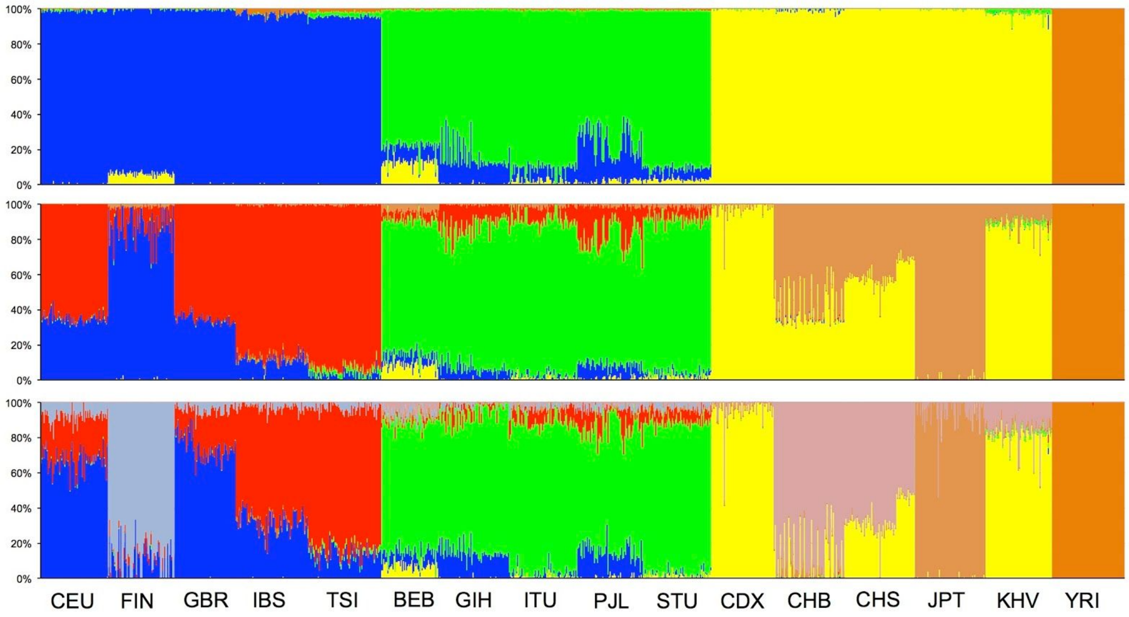

Supplementary Figure 45. Clustering for 1000 Genomes Eurasian+YRI samples for unsupervised ADMIXTURE analysis for K=4 (top row), 6 (middle row) and 8 (bottom row).

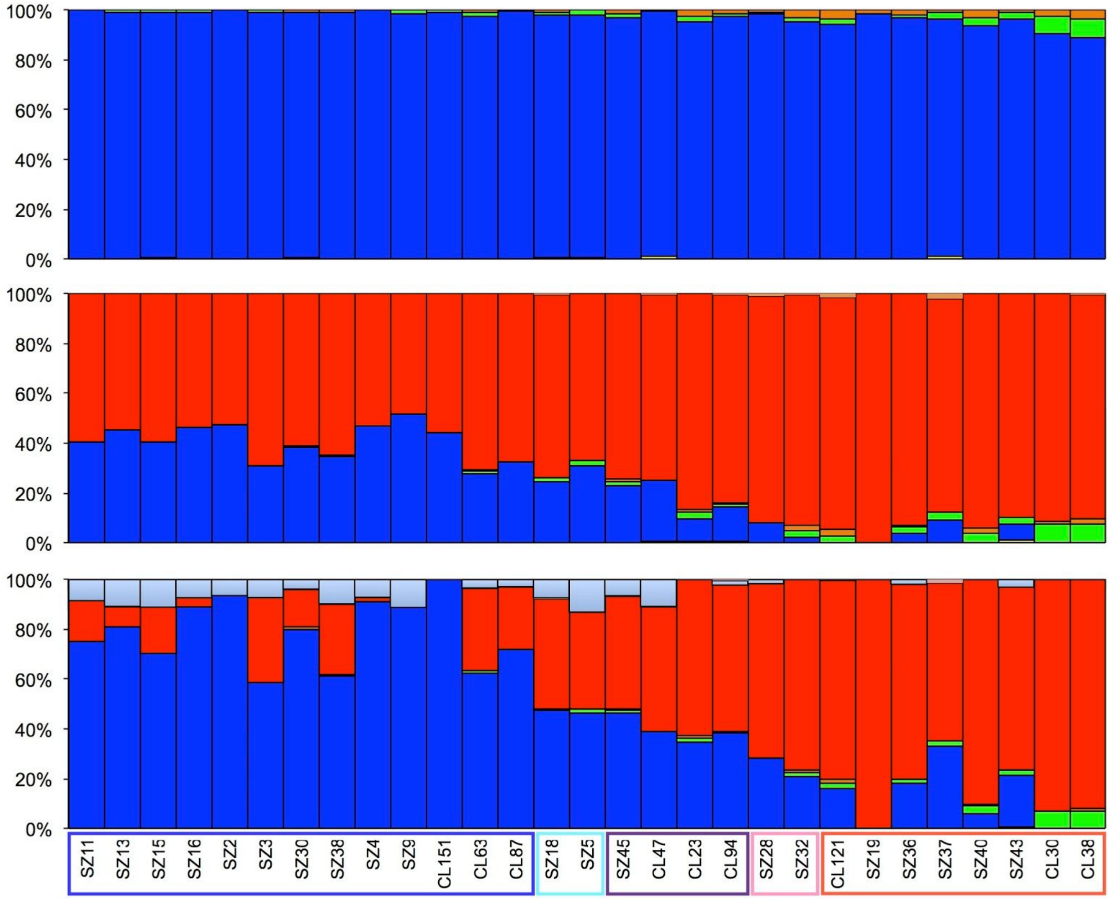

Supplementary Figure 46. Clustering for Szólád and Collegno samples for unsupervised ADMIXTURE analysis for *K*=4 (top row), 6 (middle row) and 8 (bottom row). Ordering of samples is based on assignment from supervised analysis. The color of the boxes around samples' names corresponds to their ancestry group following Fig 2 in the main text.

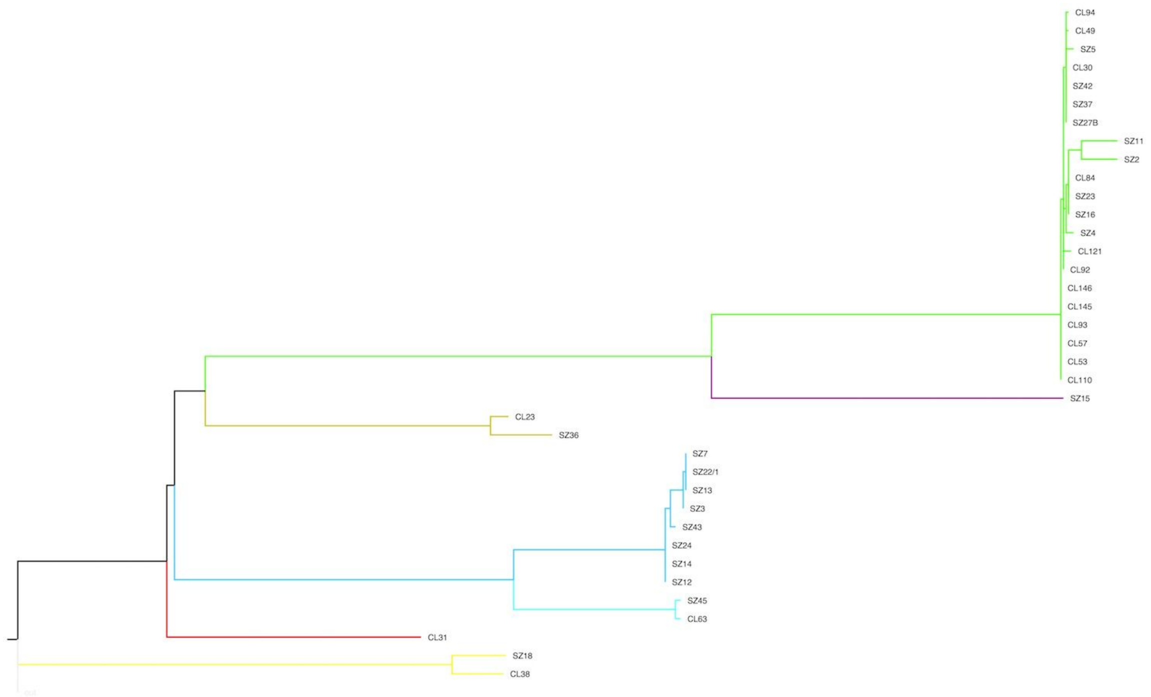

Supplementary Figure 47. Parsimony-based phylogenetic tree of 37 ancient (CL = Collegno; SZ = Szólád) Y-chromosome sequences. Colored branches represent different Y-chromosome haplotypes: E1b = yellow; G2a = red; I1a = light blue; I2a = blue; T1a = light green; R1a = purple; R1b = green.

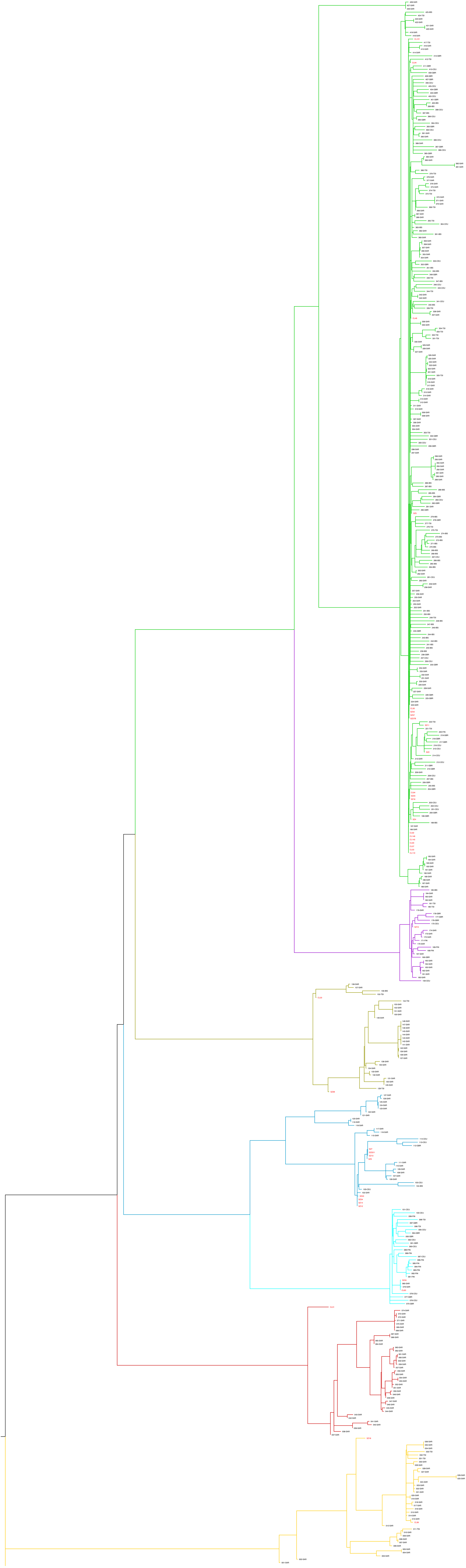

Supplementary Figure 48. Parsimony-based phylogenetic tree of 37 ancient samples (CL = Collegno; SZ = Szólád) and 428 modern European (CEU = Central Europeans from Utah; FIN = Finnish; GBR = Britons; IBS = Iberians; SAR = Sardinians; TSI = Tuscans) Y-chromosome sequences. Colored branches represent different Y-chromosome haplotypes: E1b = yellow; G2a = red; I1a = light blue; I2a = blue; T1a = light green; R1a = purple; R1b = green.

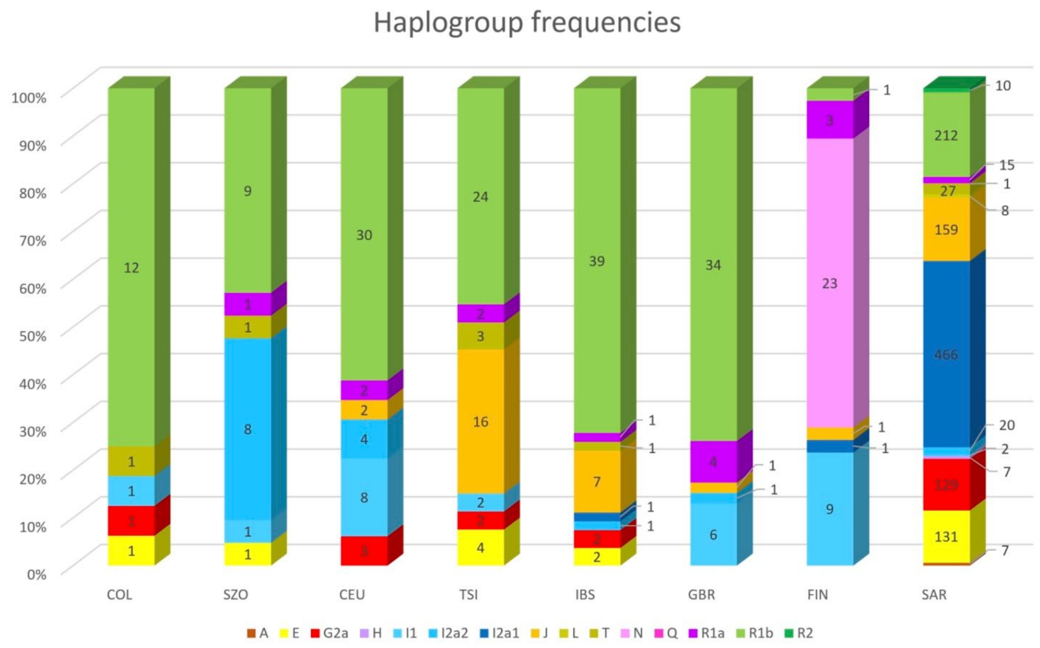

Supplementary Figure 49. Relative and absolute haplogroup frequencies: COL = Collegno; SZO = Szólád; CEU = Central European from Utah; FIN = Finnish; GBR = Britons; IBS = Iberians; SAR = Sardinians; TSI = Tuscans.

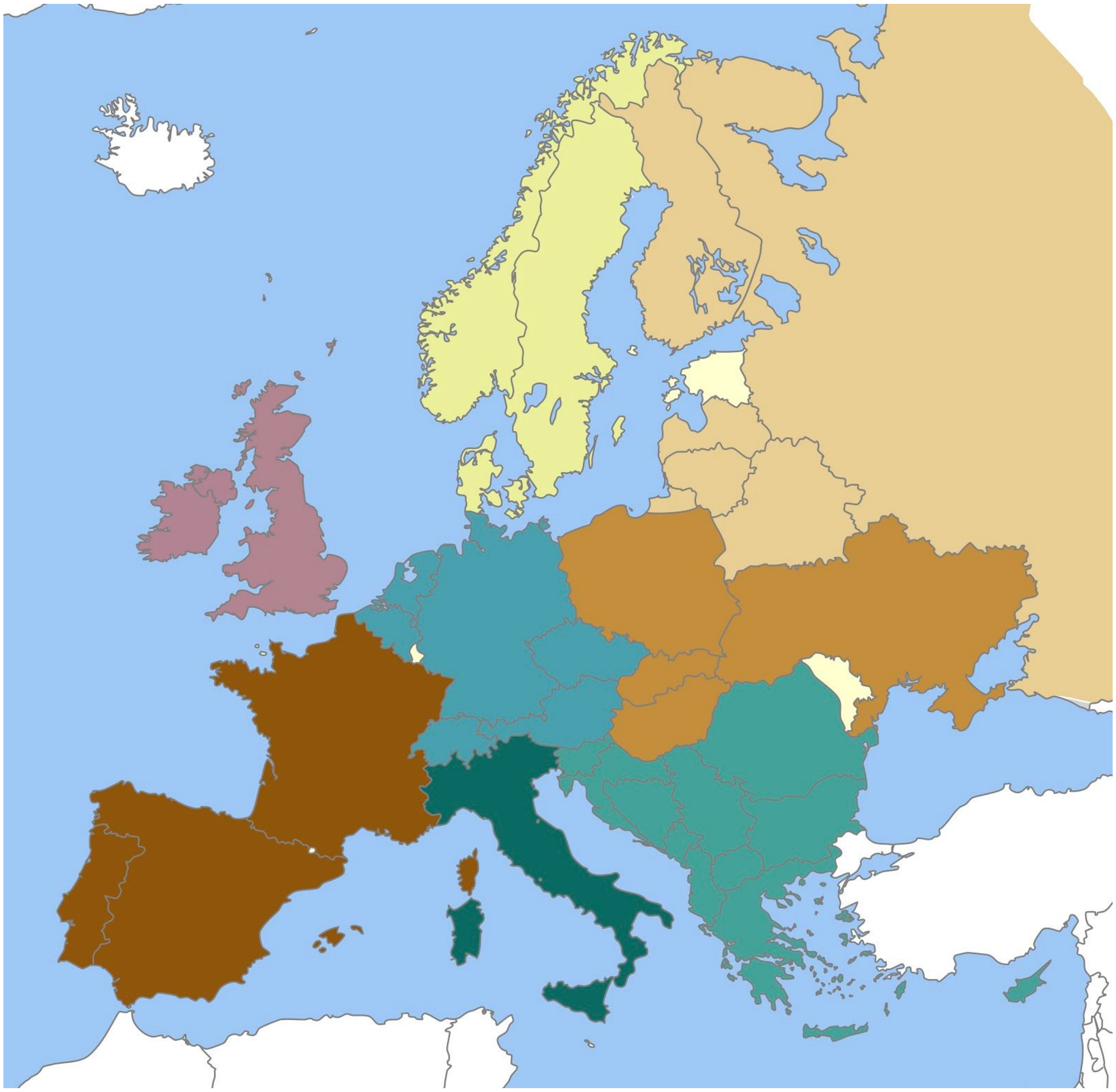

Supplementary Figure 50. Correspondence between modern political borders and European region definition utilized in this study. Northwest Europe (NWE) = pale violet, Northern Europe (NE) = khaki, Northeastern Europe (NEE) = light brown, Central Europe (CE) = aquamarine, Eastern Europe (EE) = brown, Western Europe (WE) = dark brown, Southern Europe (SE) = teal, Southeastern Europe (SEE) = light green, Hungary (HUN) = light gold. This image is modified from the original 'Blank Map of Europe -w boundaries.svg' (https://commons.wikimedia.org/wiki/File:Blank_Map_of_Europe_-w_boundaries.svg) by Madman2001 (https://commons.wikimedia.org/wiki/User:Madman2001) and is licensed under the Creative Commons Attribution-Share Alike 3.0 Unported, 2.5 Generic, 2.0 Generic and 1.0 Generic licenses.

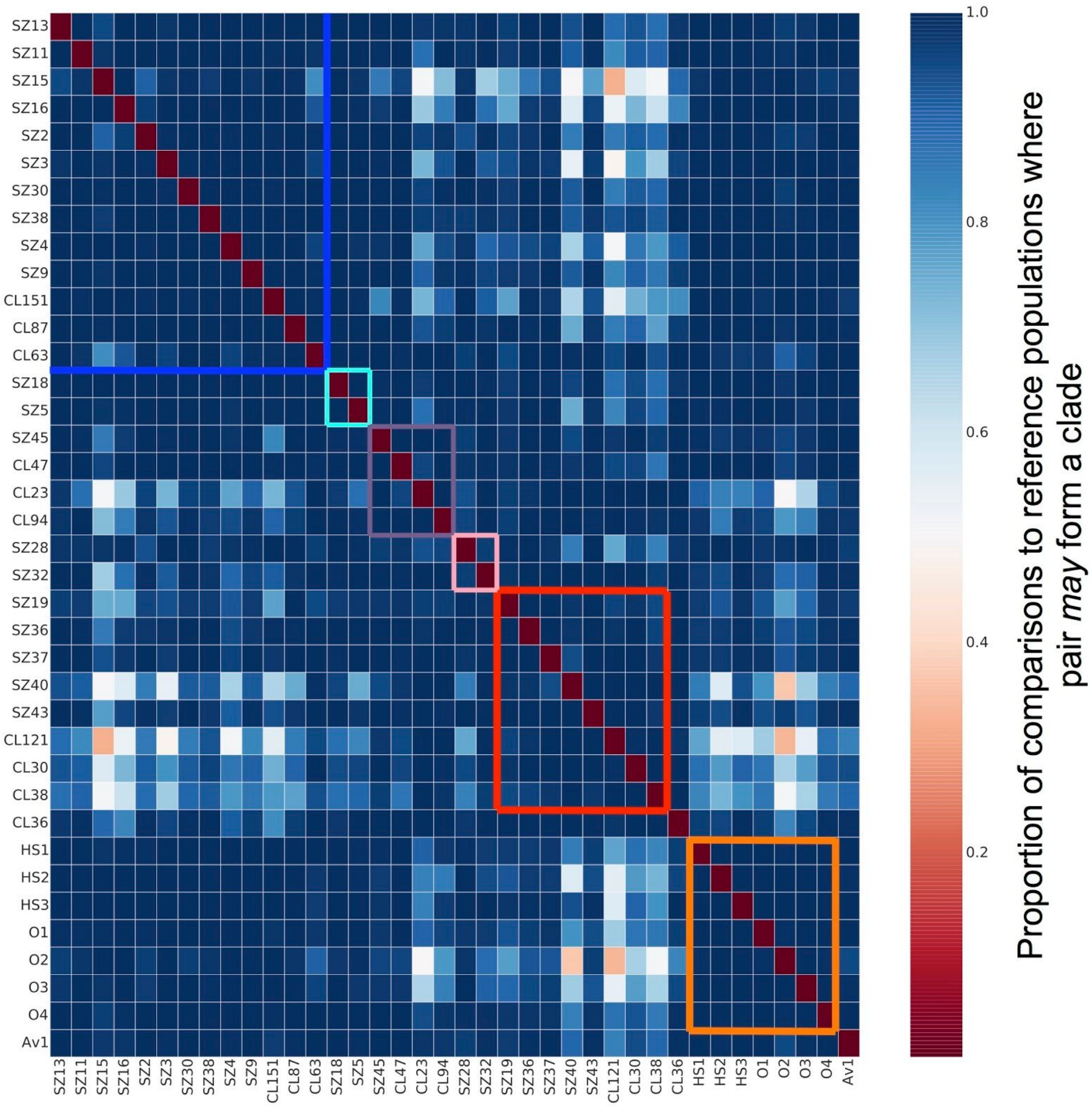

Supplementary Figure 51. Heat map pairwise matrix showing proportion of comparisons to SGDP reference populations where ancient pair may form a clade from an D-statistic test, using B_Yoruba-3 as an outgroup. Cells are highlighted with dark blue, light blue, purple, pink, and red squares according to their grouping based on labelling in Fig 2 in the main text. The orange square indicates the Anglo-Saxon samples from Schiffels et al.

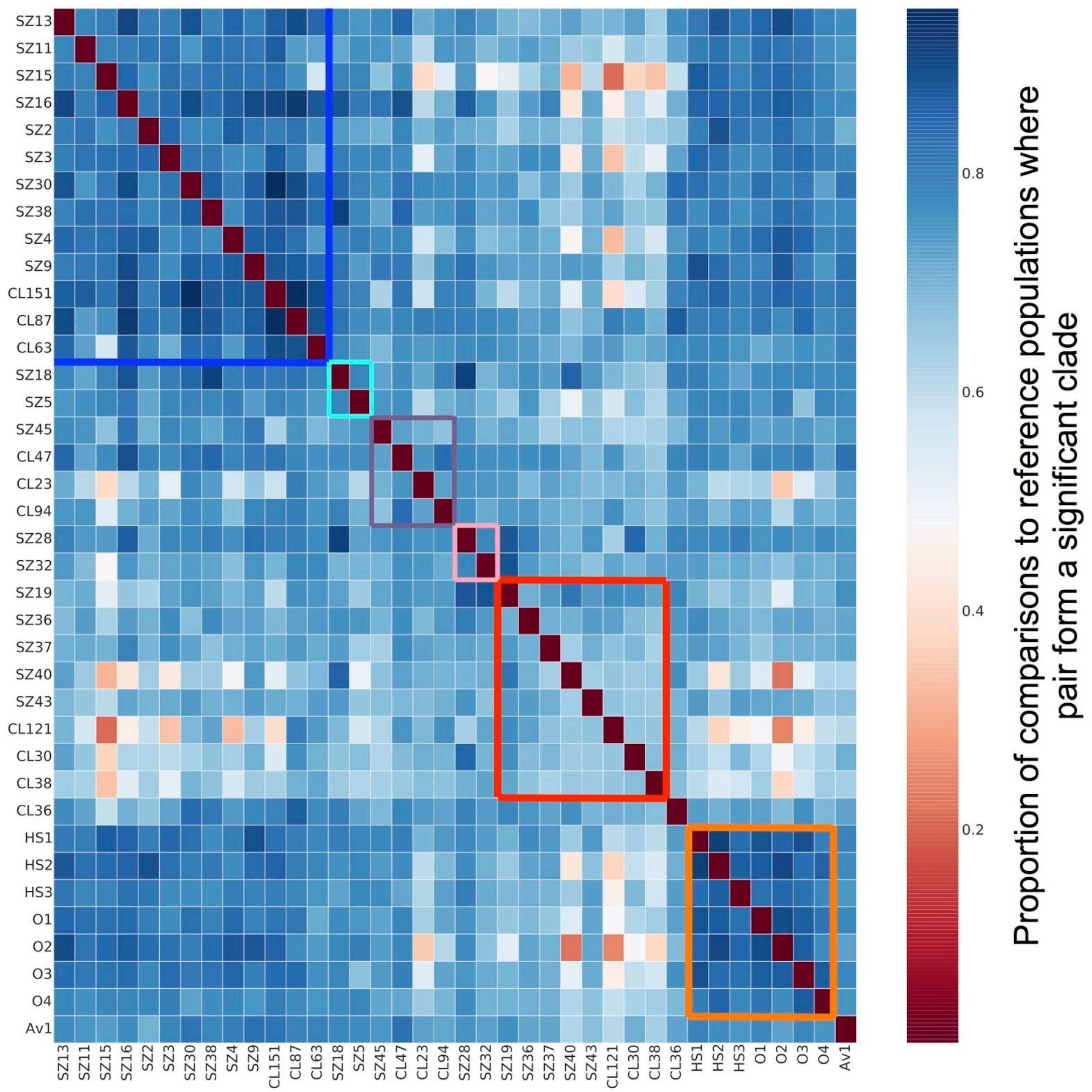

Supplementary Figure 52. Heat map pairwise matrix showing proportion of comparisons to SGDP reference populations where ancient pair form a significant clade from a D-statistic test, using B_Yoruba-3 as an outgroup. Cells are highlighted with squares according to Supplementary Figure 51.

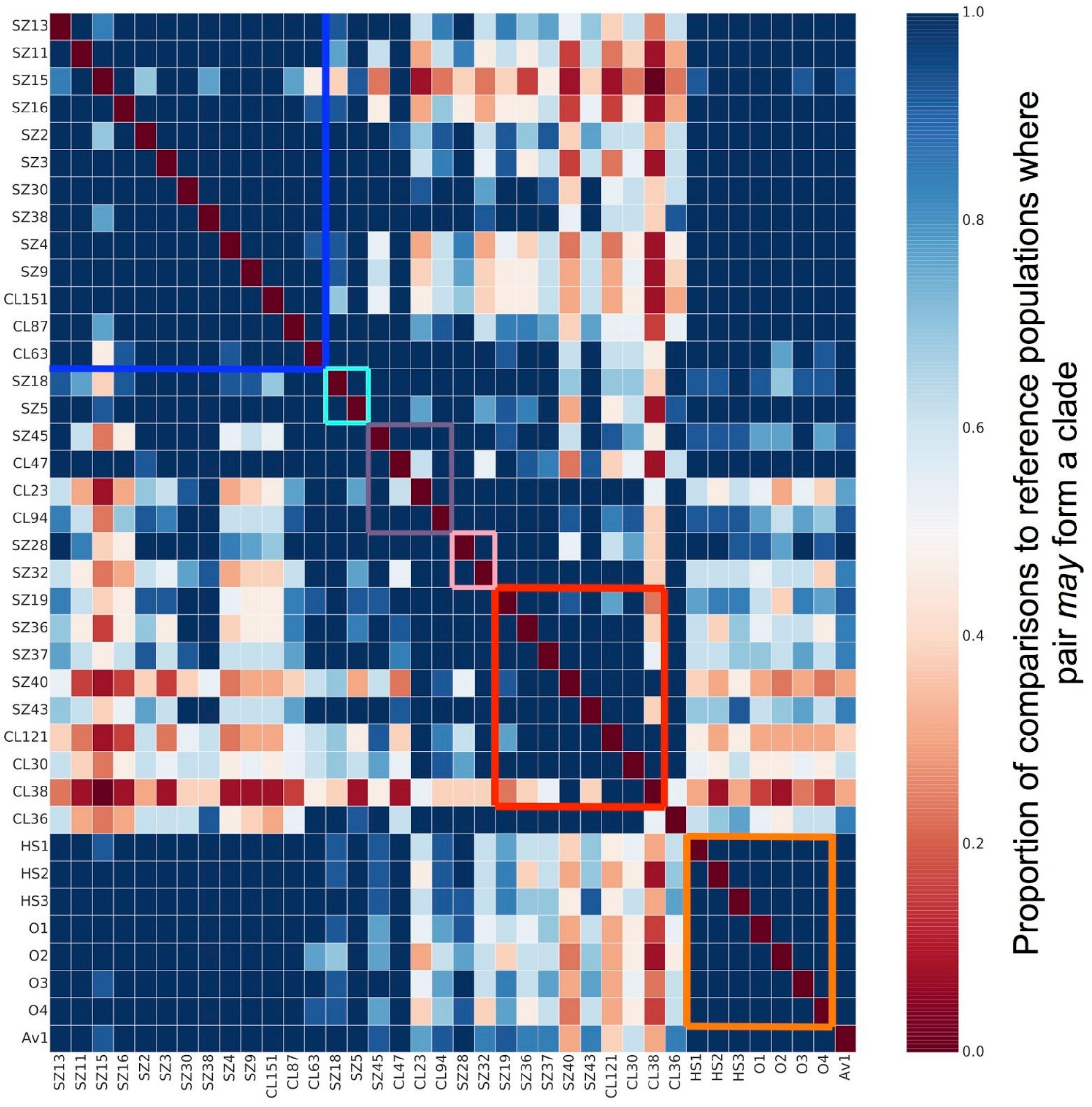

Supplementary Figure 53. Heat map pairwise matrix showing proportion of comparisons to HellBus region reference populations where ancient pair may form a clade from a D-statistic test, using Yoruba as an outgroup. Cells are highlighted with squares according to Supplementary Figure 51.

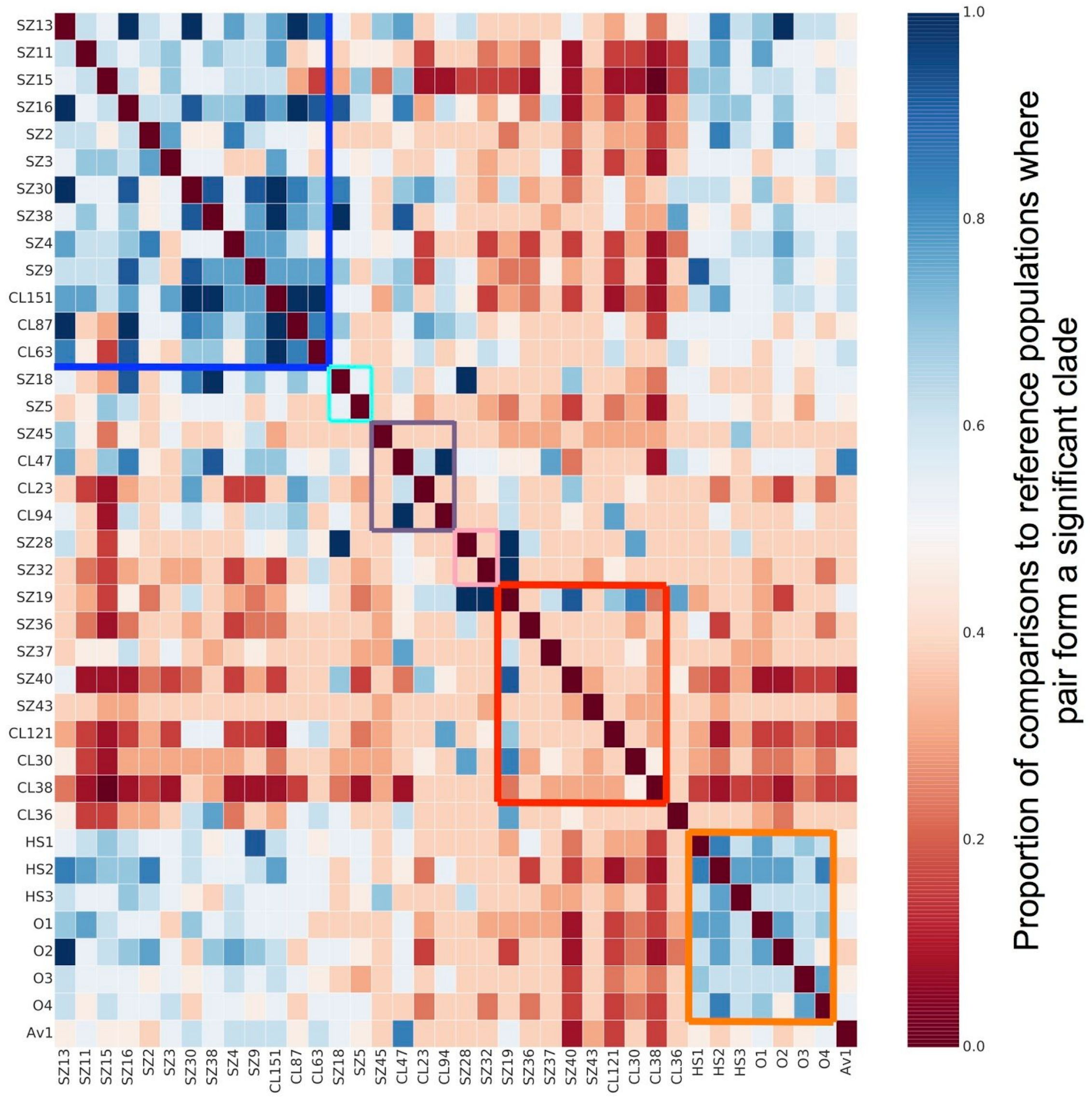

Supplementary Figure 54. Heat map pairwise matrix showing proportion of comparisons to HellBus region reference populations where ancient pair form a significant clade from a D-statistic test, using Yoruba as an outgroup. Cells are highlighted with squares according to Supplementary Figure 51.

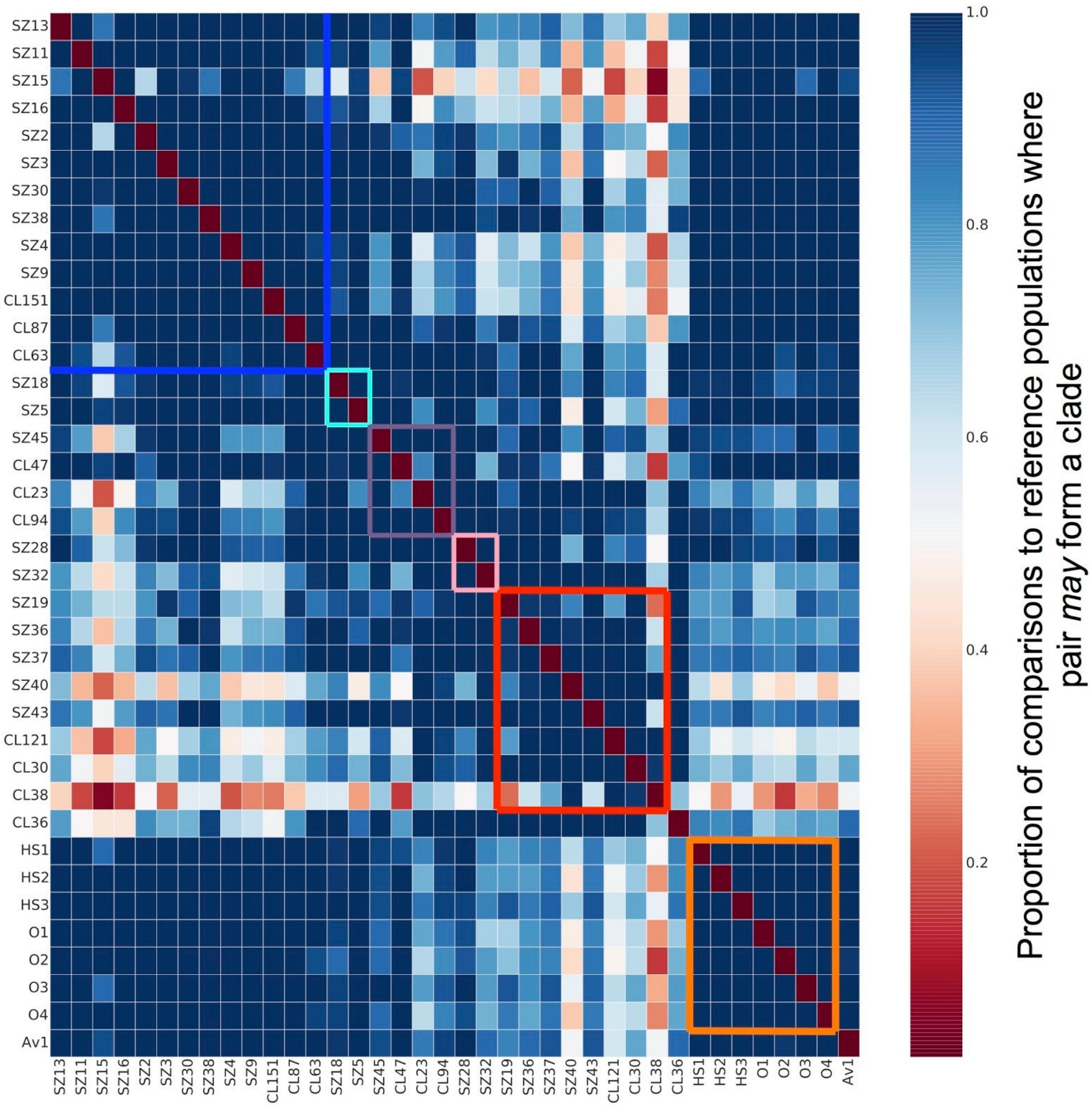

Supplementary Figure 55. Heat map pairwise matrix showing proportion of comparisons to HellBus country reference populations where ancient pair may form a clade from a D-statistic test, using Yoruba as an outgroup. Cells are highlighted with squares according to Supplementary Figure 51.

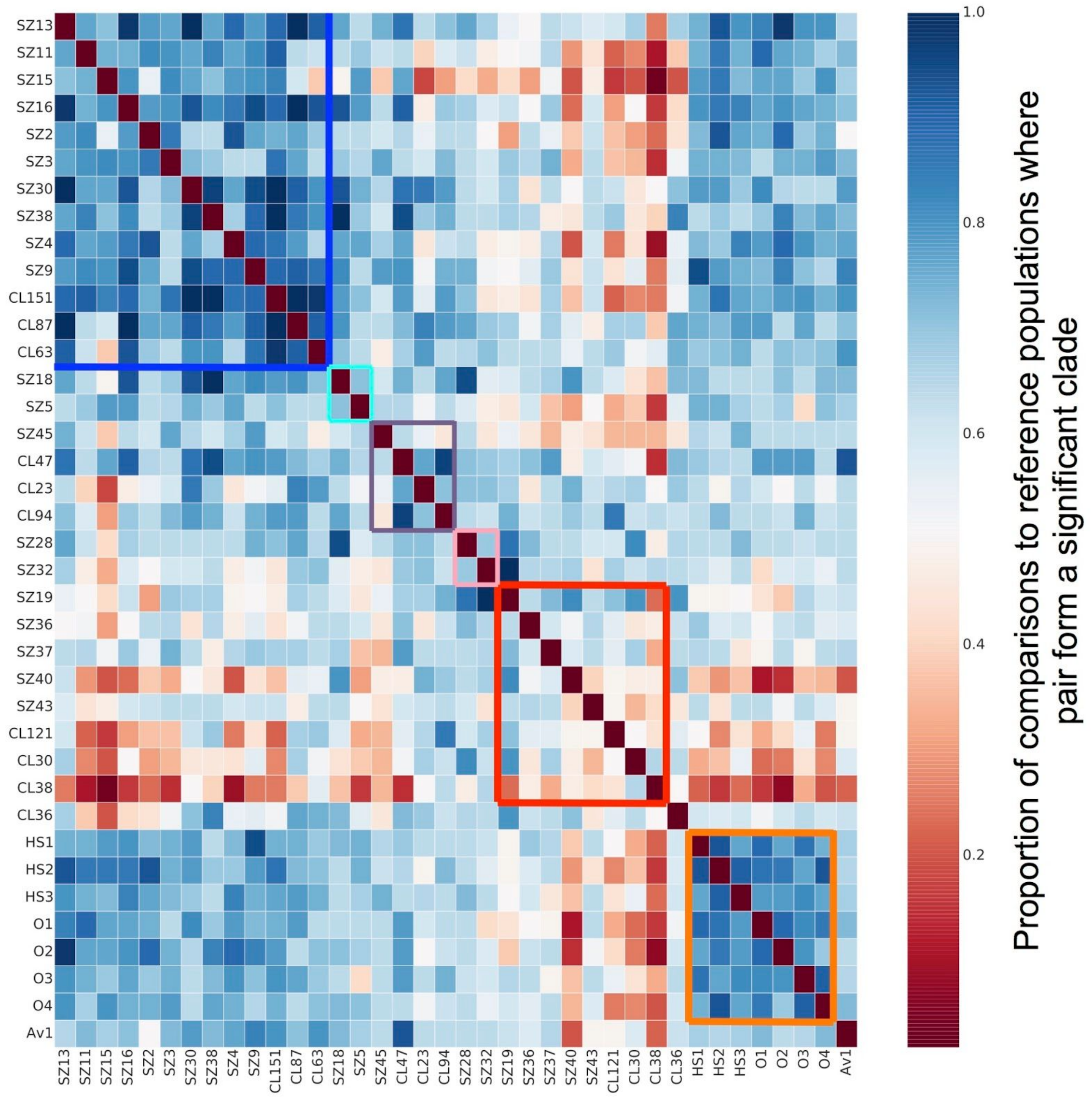

Supplementary Figure 56. Heat map pairwise matrix showing proportion of comparisons to HellBus country reference populations, where ancient pair forms a significant clade from a D-statistic test, using Yoruba as an outgroup. Cells are highlighted with squares according to Supplementary Figure 51.

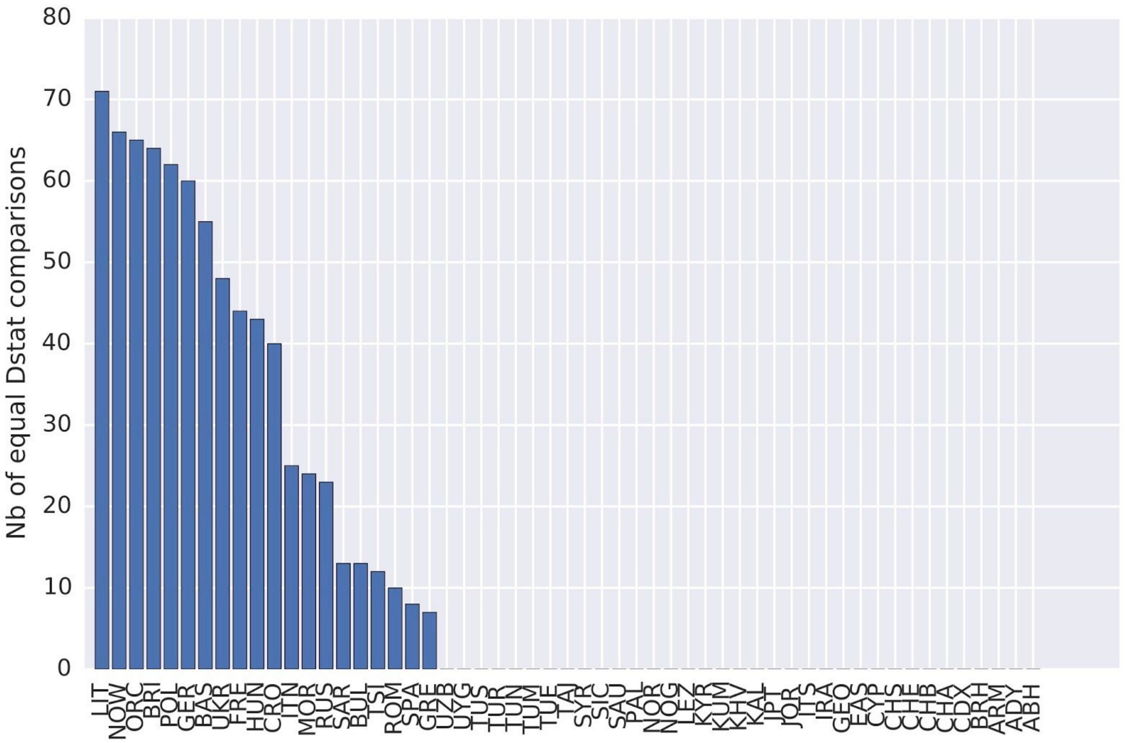

Supplementary Figure 57. Number of pairwise comparisons involving ancient populations of predominantly central/northern European ancestry (see Fig 1) where the two ancient populations could not be significantly distinguished from a particular HellBus country based on a D-statistic test.

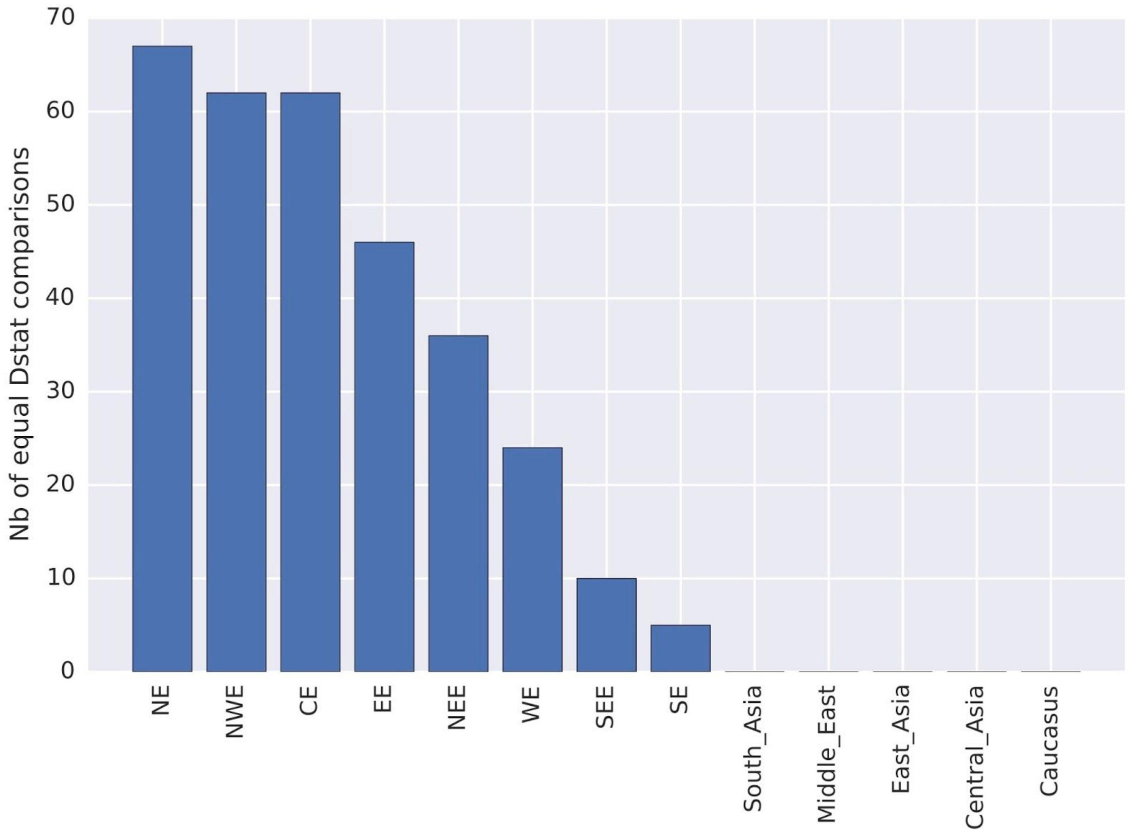

Supplementary Figure 58. Number of pairwise comparisons involving ancient populations of predominantly central/northern European ancestry (see Fig 1) where the two ancient populations could not be significantly distinguished from a particular HellBus region based on a D-statistic test.

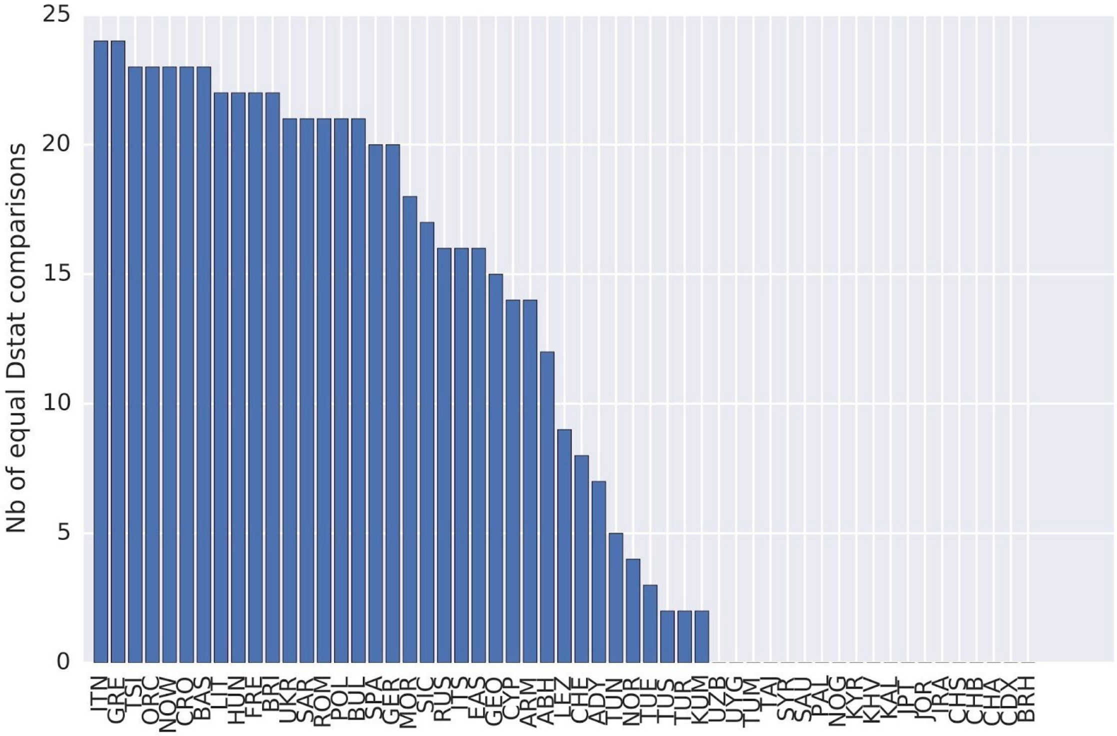

Supplementary Figure 59. Number of pairwise comparisons involving ancient populations of predominantly southern European ancestry (see Fig 1) where the two ancient populations could not be significantly distinguished from a particular HellBus country based on a D-statistic test.

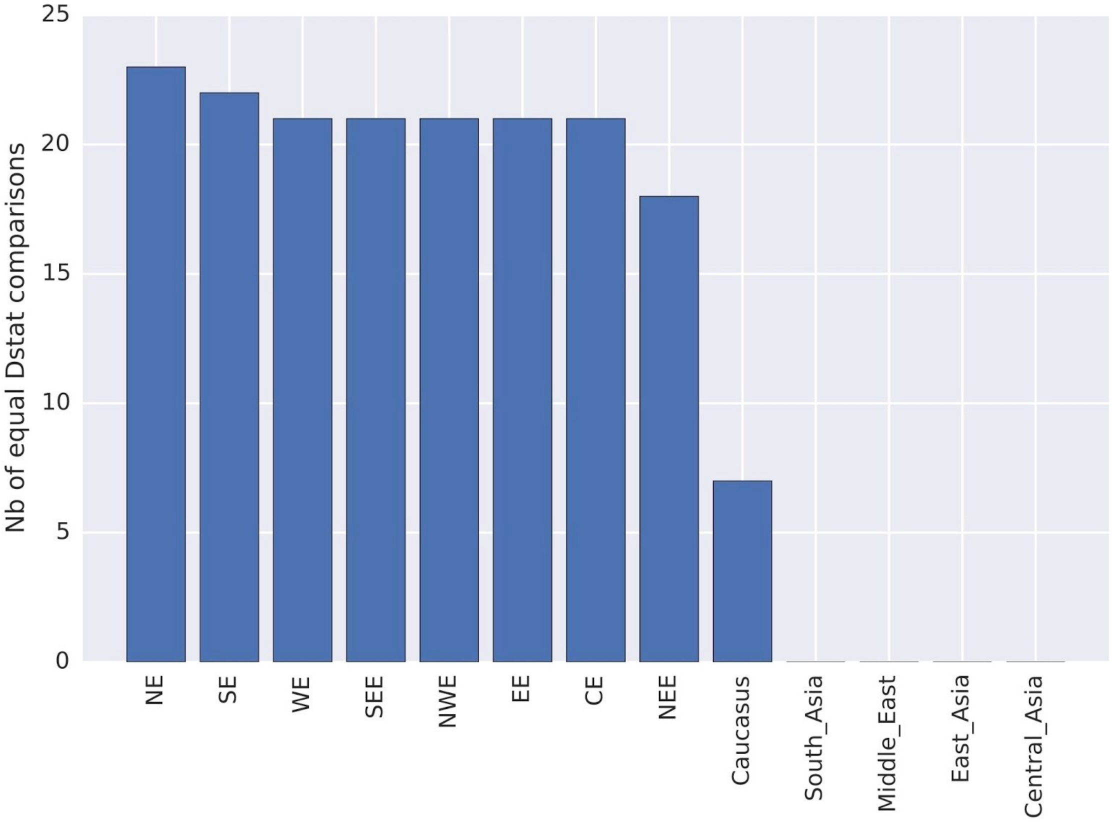

Supplementary Figure 60. Number of pairwise comparisons involving ancient populations of predominantly southern European ancestry (see Fig 1) where the two ancient populations could not be significantly distinguished from a particular HellBus country based on a D-statistic test.

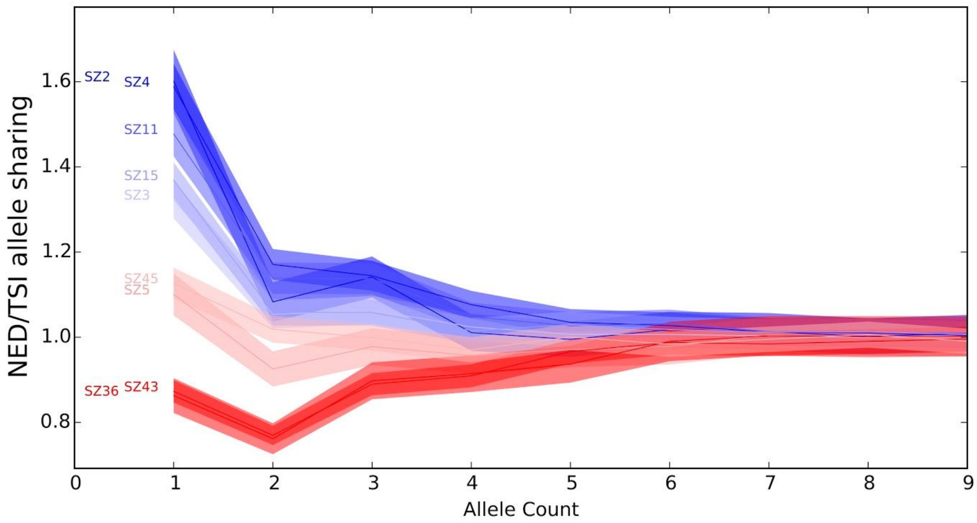

Supplementary Figure 61. Relative NED/TSI allele sharing for medieval whole genomes for rare variants (derived allele count <10).

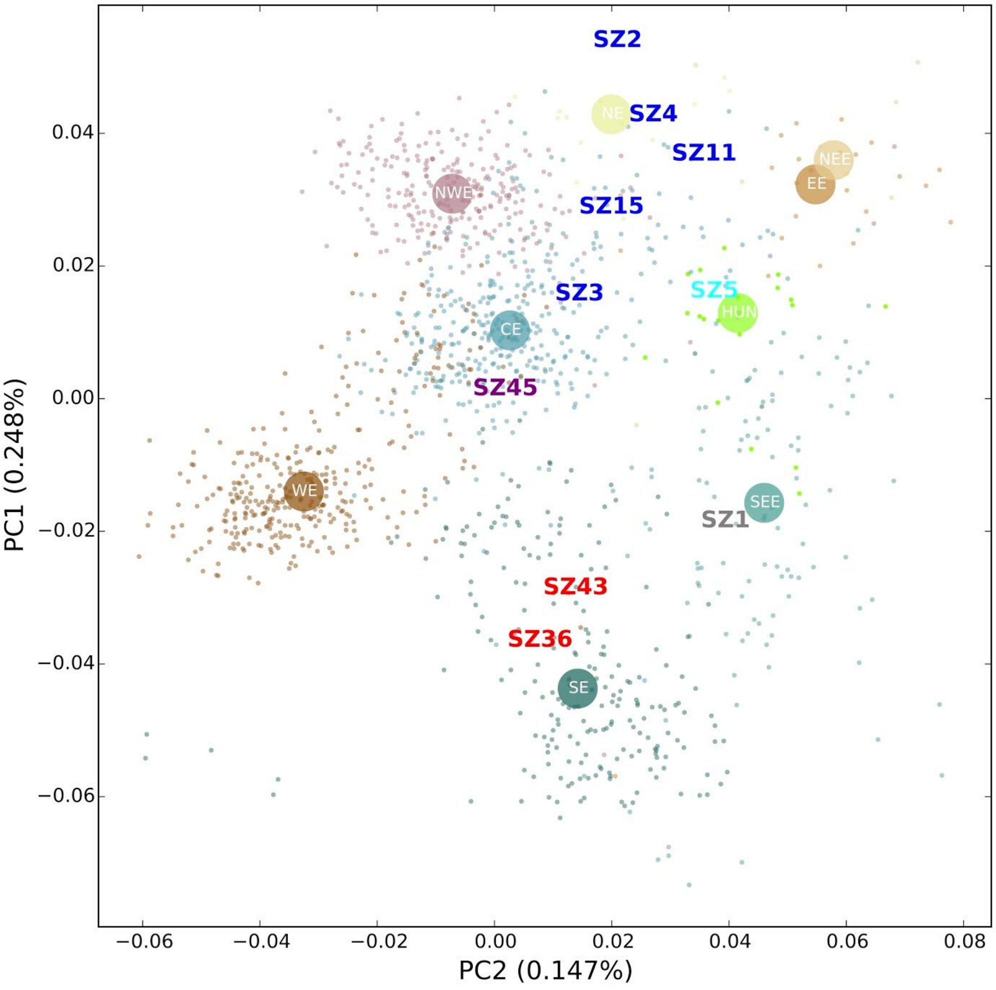

Supplementary Figure 62. PCA of POPRES samples and 10 whole genome sequences generated in the study. Color coding of medieval samples is same as in Figs 1 and 2.

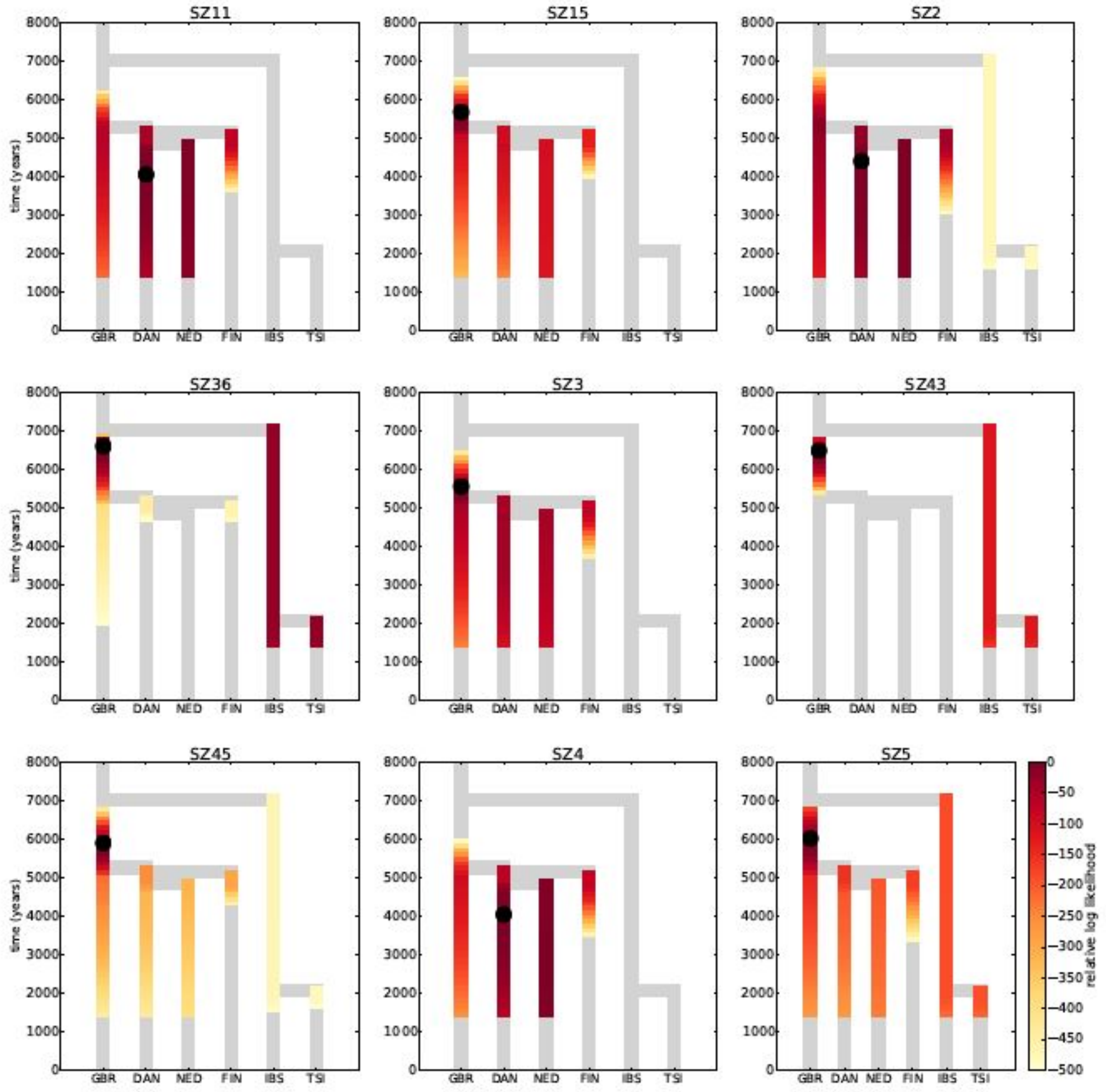

Supplementary Figure 63. rarecoal analysis, placing each of the 9 medieval whole genomes with pseudo-haploid calls on a branching model of European demographic history. Black dot represent ML estimate. Scaled time from rarecoal converted to years assuming mutation rate of 1.25 x 10^{-8} per generation and generation time of 29 years.

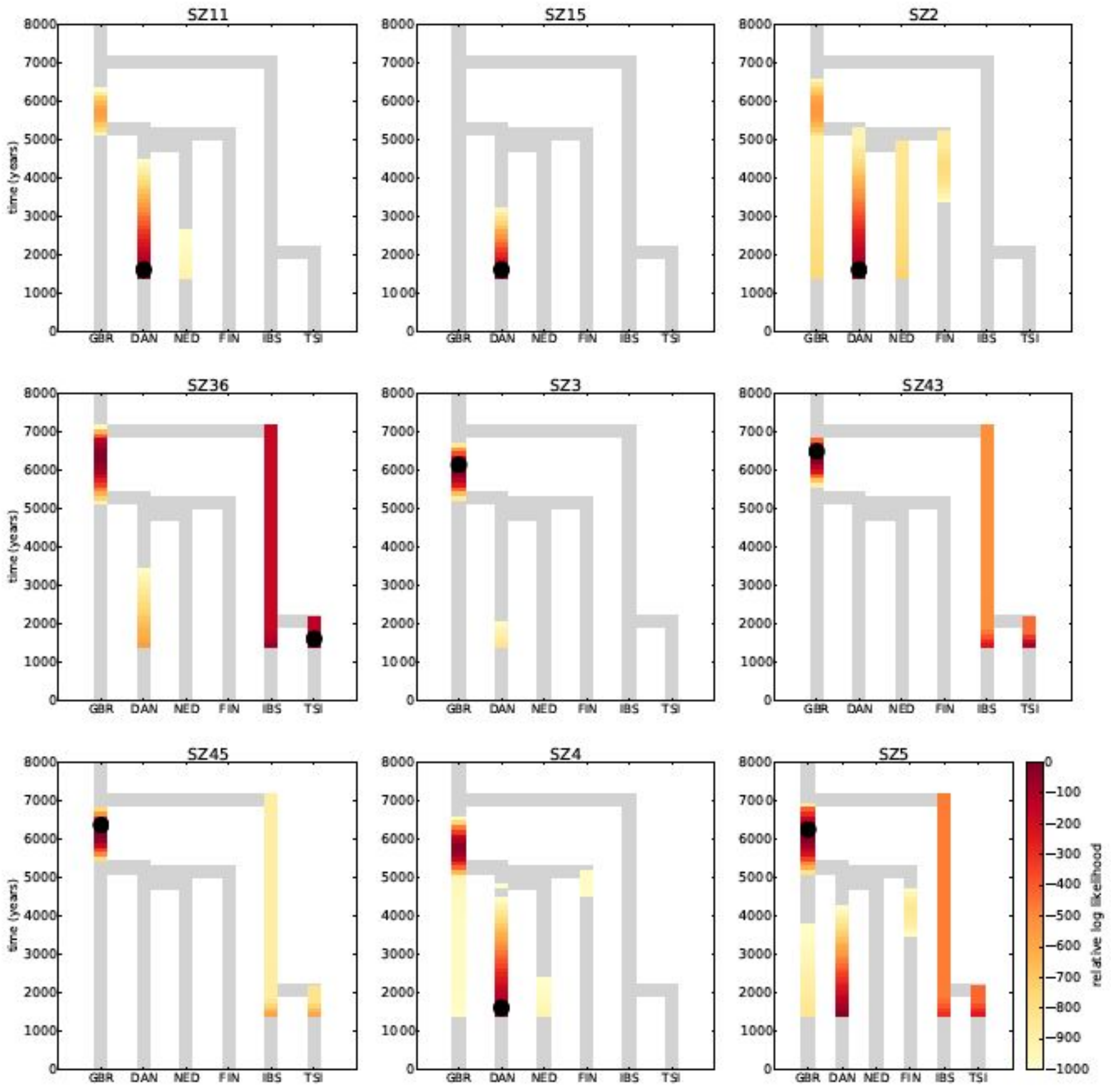

Supplementary Figure 64. rarecoal analysis, placing each of the 9 medieval whole genomes with diploid calls on a branching model of European demographic history. Scaled time from rarecoal converted to years assuming mutation rate of 1.25 x 10^{-8} per generation and generation time of 29 years.

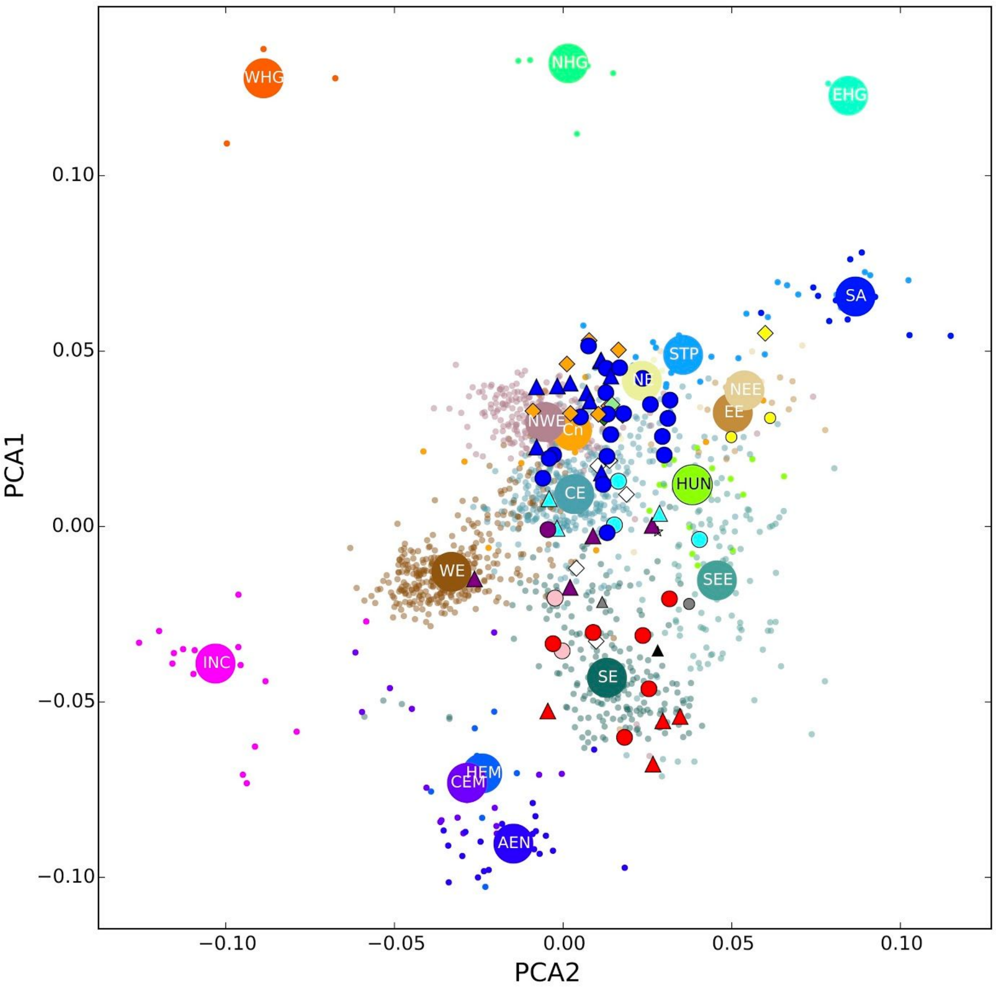

Supplementary Figure 65. Procrustes transformed PCA of medieval and pre-Bronze age samples from Mathieson et al. (2015) against POPRES imputed SNP dataset. Color coding of medieval samples is same as in Figs 1 and 2. Labels for POPRES regions: NWE = northwest Europe, NE = modern north Europe, NEE = modern northeast Europe, CE =central Europe, EE = eastern Europe, WE =Western Europe, SE = southern Europe, SEE = southeast Europe. Labels for prehistorical samples: WHG=Western hunter-gatherers, NHG=Northern hunter-gatherers, EHG=Eastern hunter=gatherers, CEM=Central European Early and Middle Neolithic, HEM=Hungarian Early and Middle Neolithic, INC=Iberian Neolithic and Chacolithic, AEN=Anatolian Neolithic, SA=Steppe Ancestry.

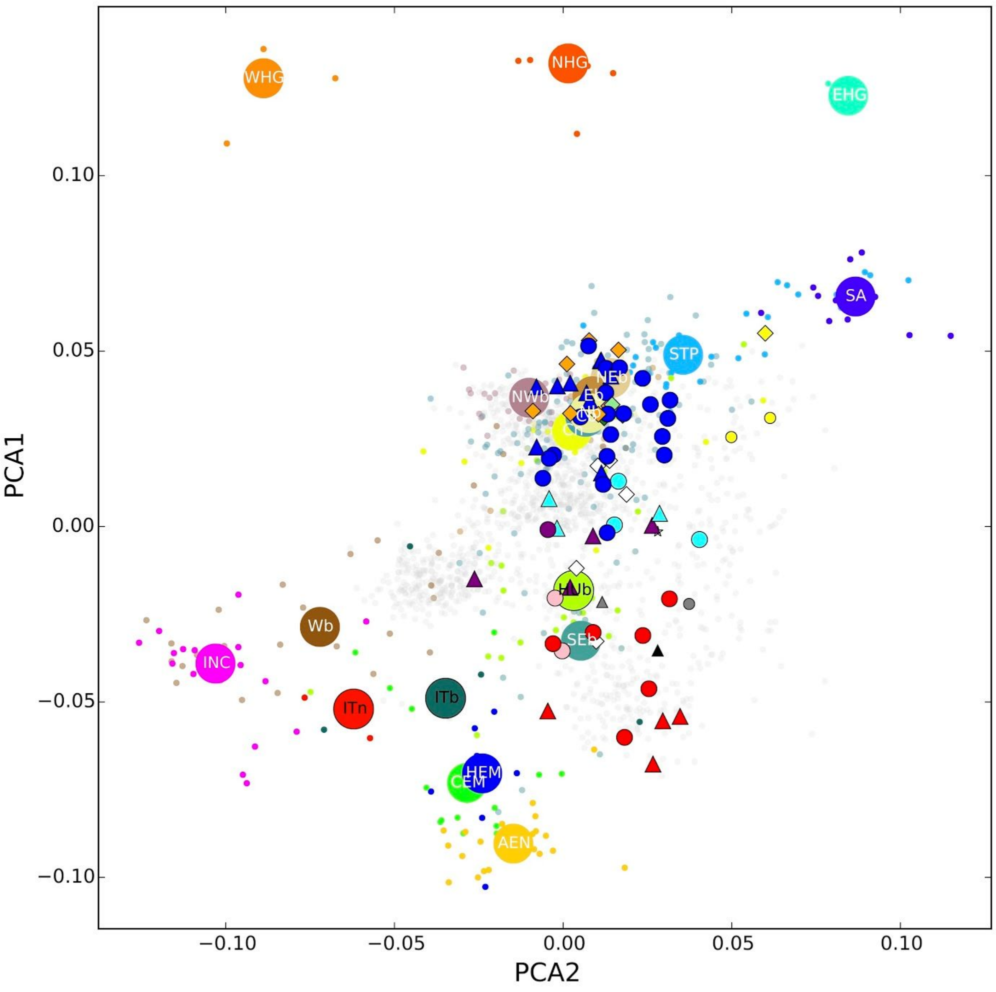

Supplementary Figure 66. Procrustes transformed PCA of medieval and prehistorical samples against POPRES imputed SNP dataset. Color coding of medieval samples is same as in Figs 1 and 2. POPRES samples shown in light grey. Labels for prehistorical samples: Nb=Northern European Bronze Age, NWb=Northern Western European Bronze Age, NEb=Northern Eastern European Bronze Age, Eb=Eastern European Bronze Age, SEb=South Eastern European Bronze Age, Sb = Southern European Bronze Age, Wb=Westerm European Bronze Age, Cb=Central European Bronze Age, HUb=Hungarian Bronze Age. Labels from Ancient Reference: WHG=Western hunter-gatherers, NHG=Northern hunter-gatherers, EHG=Eastern hunter=gatherers, CEM=Central European Early and Middle Neolithic, HEM=Hungarian Early and Middle Neolithic, INC=Iberian Neolithic and Chacolithic, AEN=Anatolian Neolithic, SA=Steppe Ancestry.

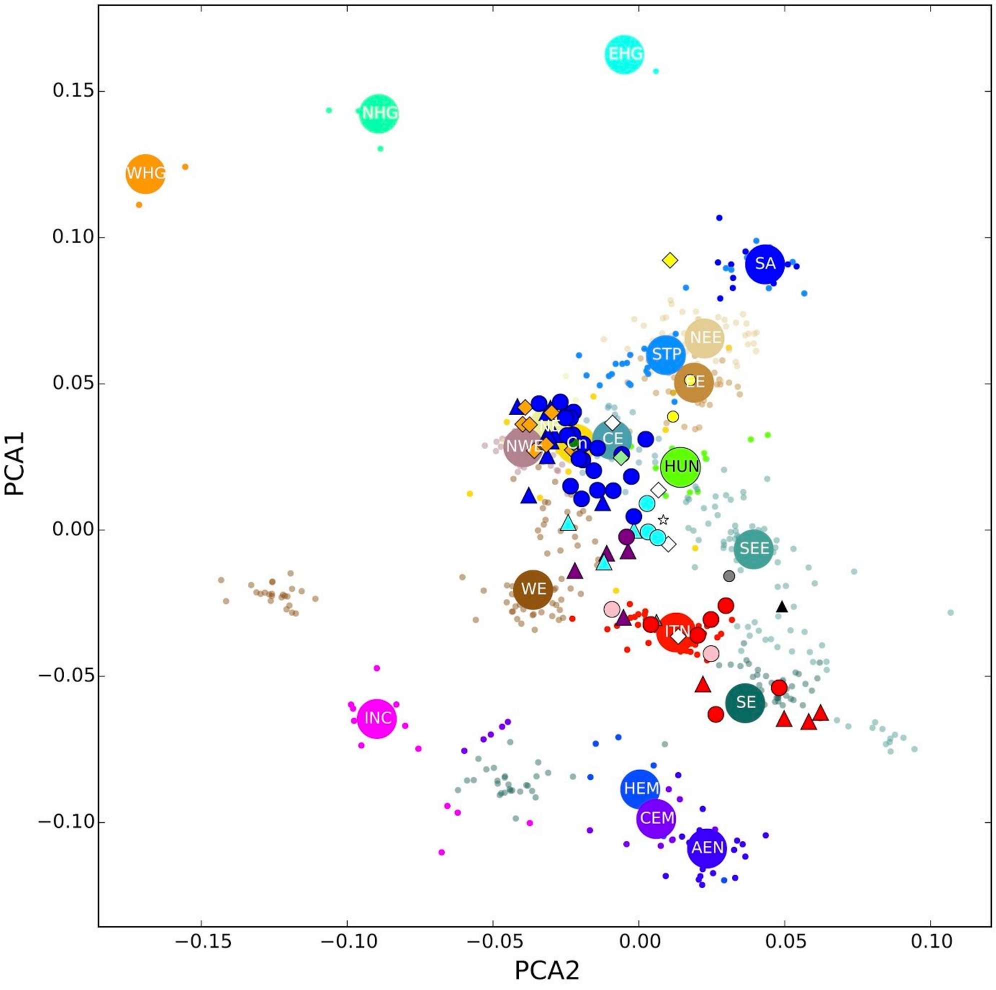

Supplementary Figure 67. Procrustes transformed PCA of medieval and pre-Bronze age samples from Mathieson et al. (2015) against HellBus imputed SNP dataset. Color coding of medieval samples is same as in Figs 1 and 2. Other coding same as for Figure 2

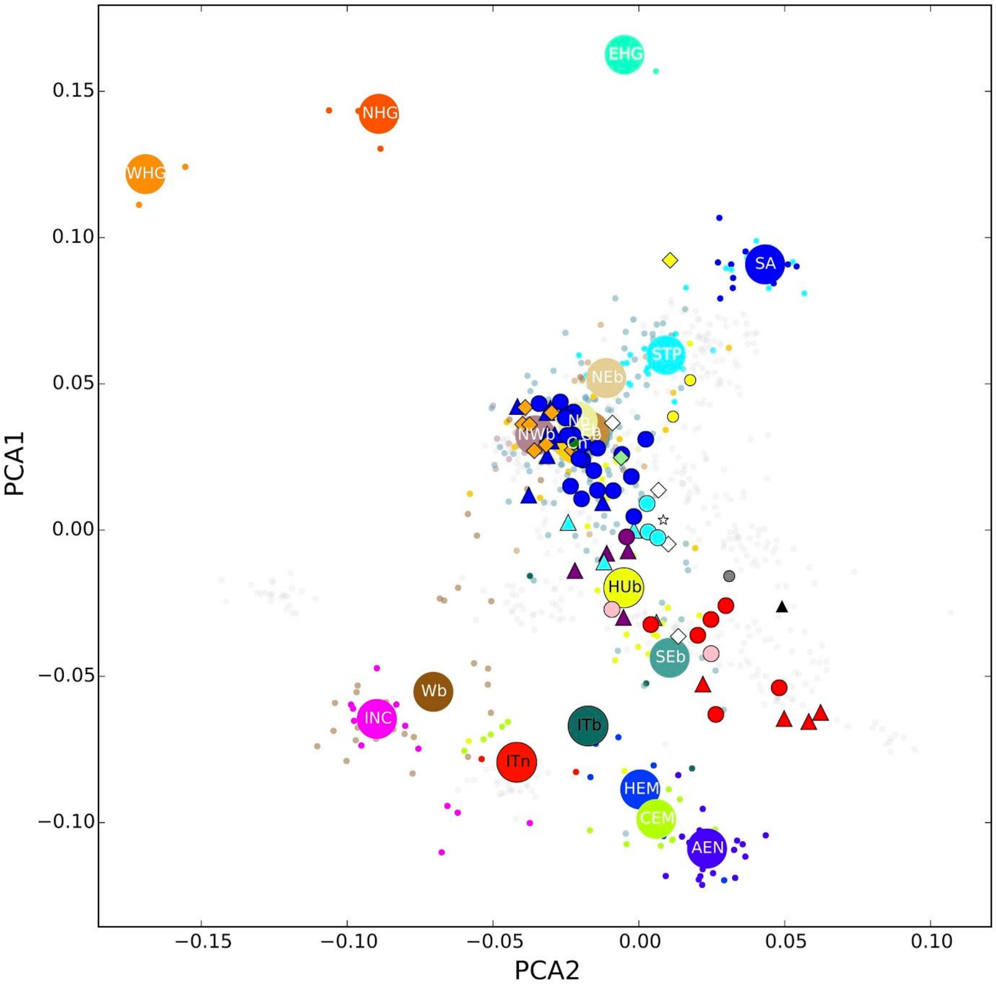

Supplementary Figure 68. Procrustes transformed PCA of medieval and prehistorical samples against HellBus imputed SNP dataset. Color coding of medieval samples is same as in Figs 1 and 2. HellBus samples shown in light grey. Other coding same as for Figure 2.

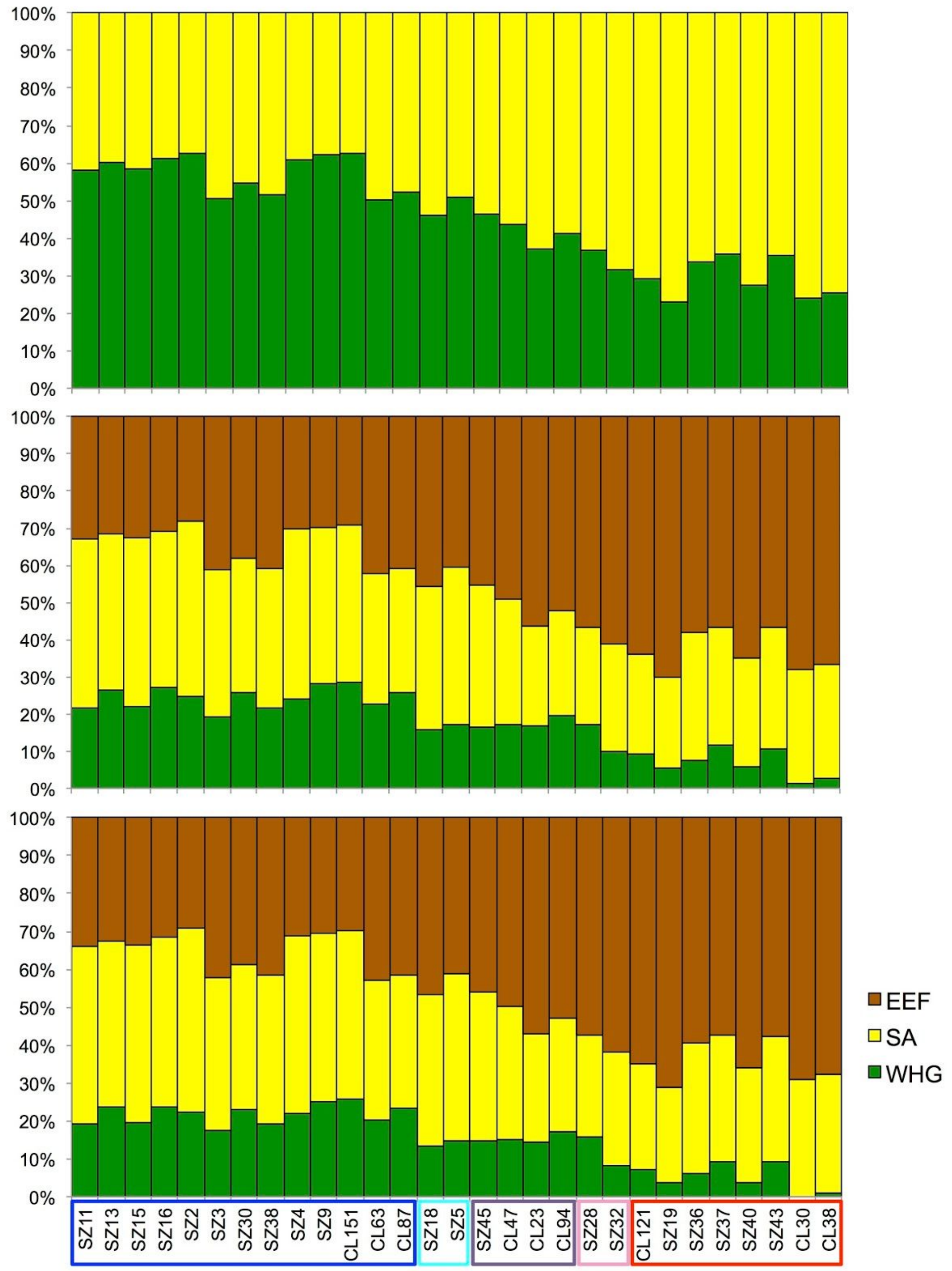

Supplementary Figure 69. ADMIXTURE estimates for unrelated Szólád and Collegno samples for unsupervised *K*=2 (top row), unsupervised *K*=3 (middle row) and supervised *K*=3 (bottom row) using prehistorical parental populations as reference samples. Ordering of samples is based on assignment from supervised analysis using modern reference populations. The color of the boxes around samples' names in the bottom corresponds to their ancestry group following Fig 2 in the main text.

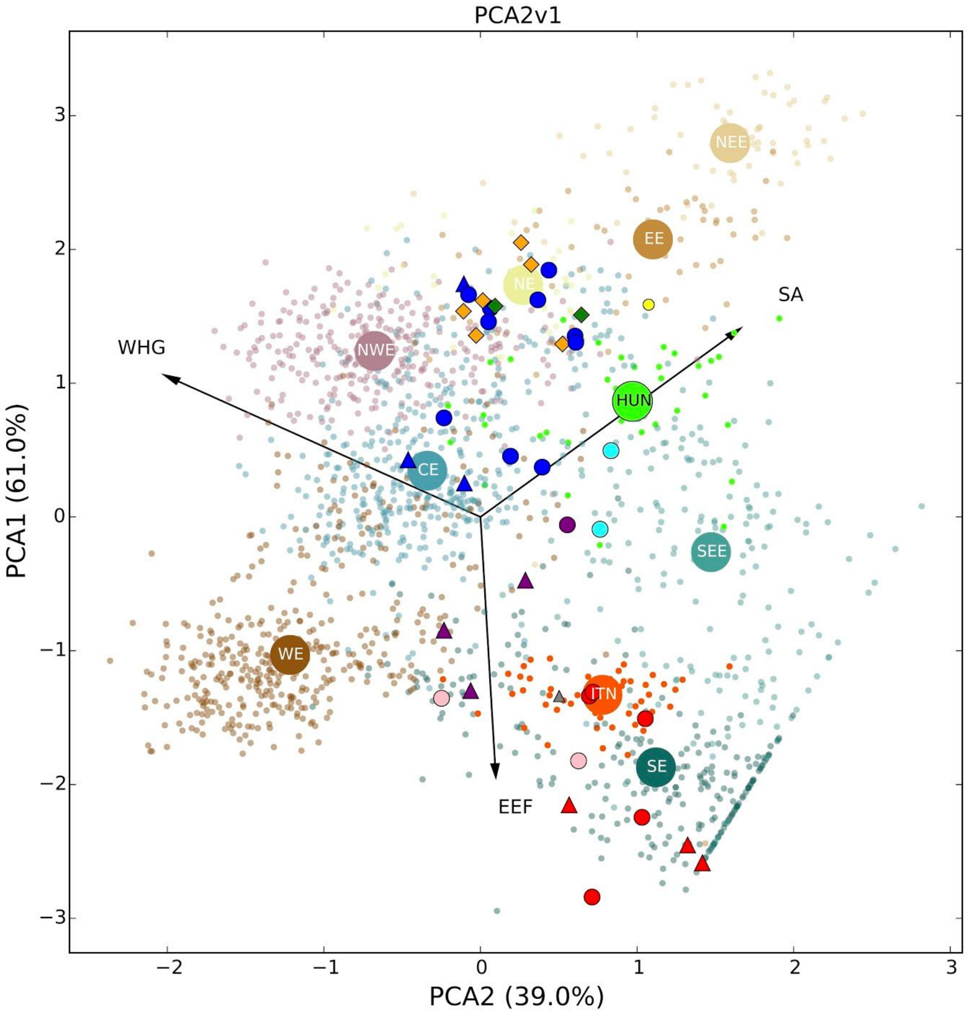

Supplementary Figure 70. PCA of supervised ADMIXTURE estimates for unrelated medieval samples and POPRES and European HellBus individuals for K=3 using prehistorical parental populations. Colour coding of medieval samples is same as in Figs 1 and 2.

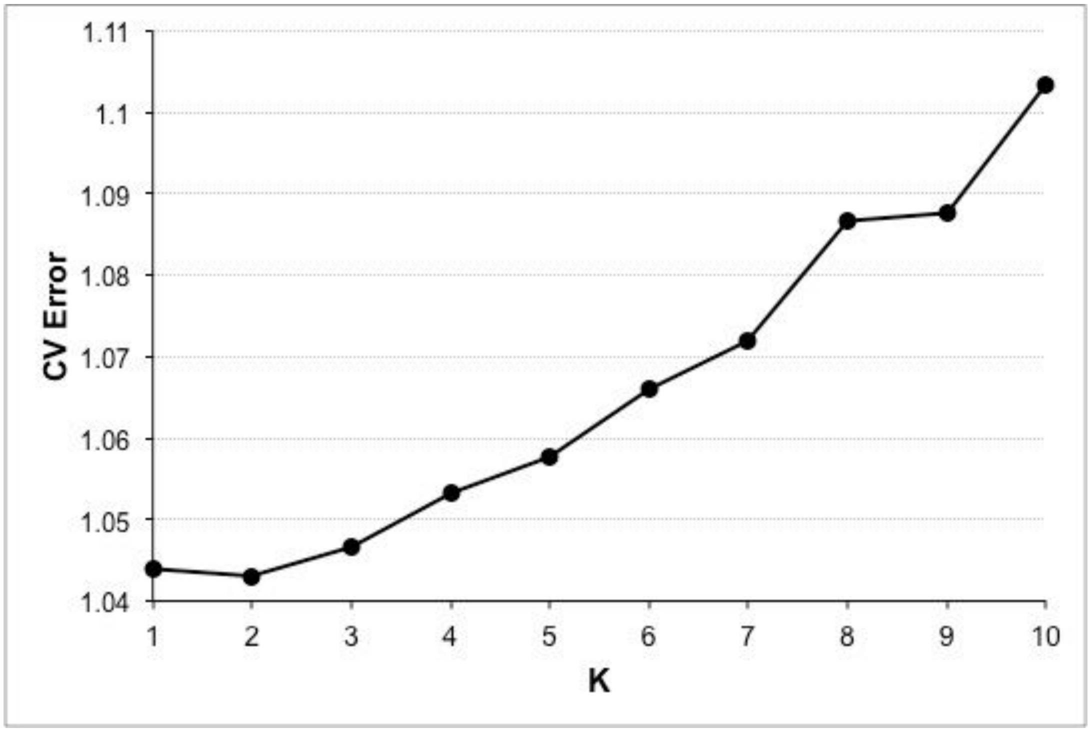

Supplementary Figure 71. Five-fold cross-validation error for *K*=1 - 10 for prehistorical, medieval and HellBus samples.

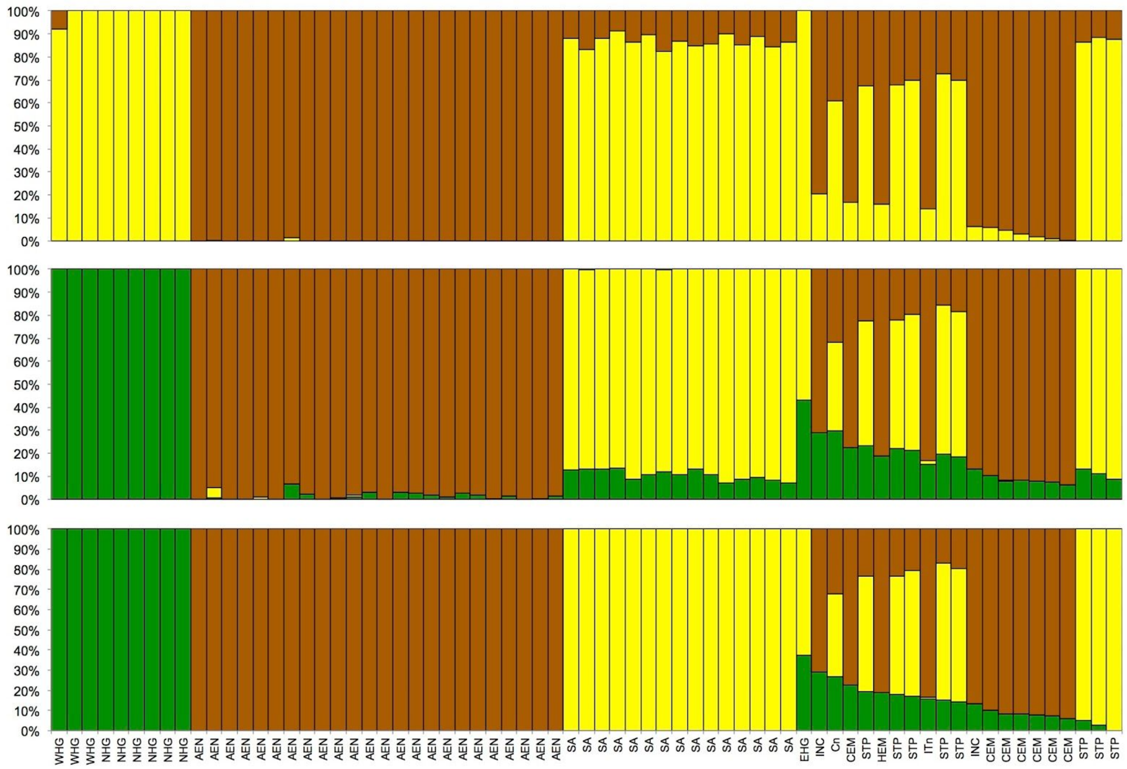

Supplementary Figure 72. ADMIXTURE estimates for pre-Bronze age samples for unsupervised *K*=2 (top row), unsupervised *K*=3 (middle row) and supervised *K*=3 (bottom row) using WHG, AEN and SA as parental populations.

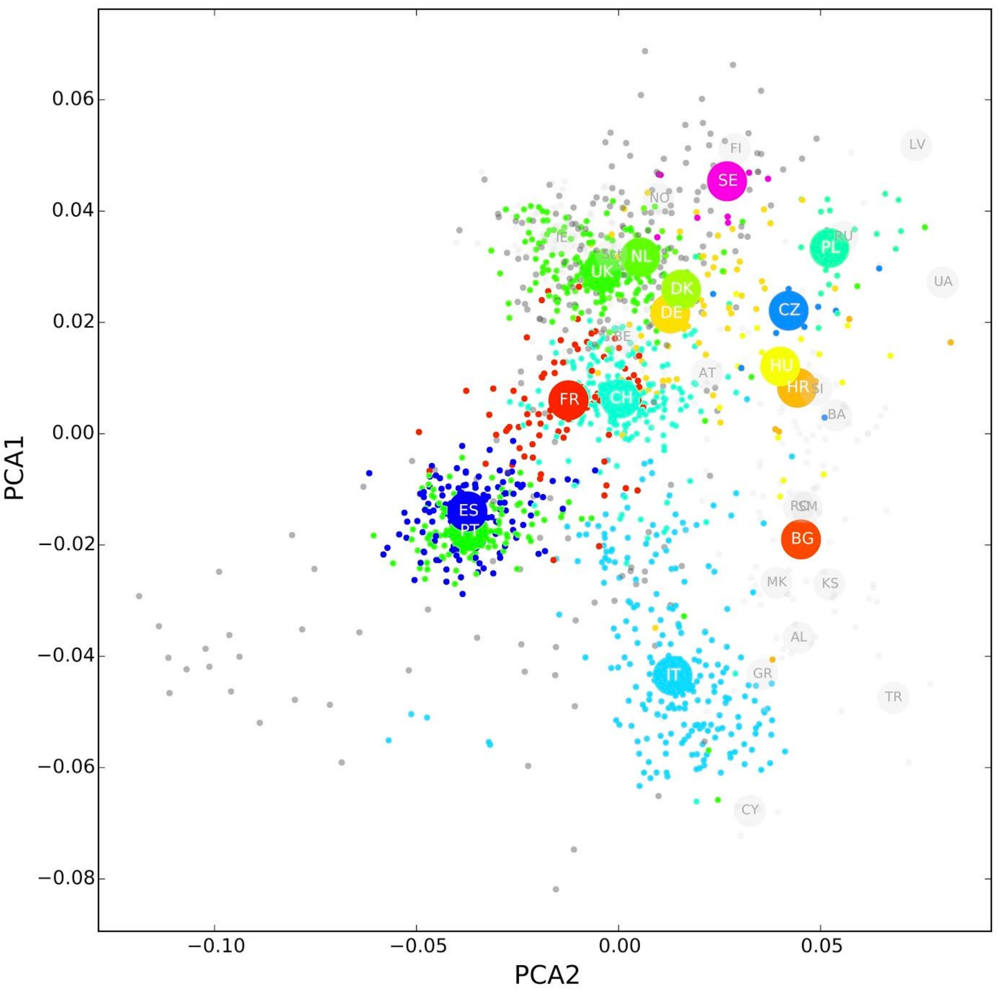

Supplementary Figure 73. Procrustes transformed PCA of Bronze Age samples POPRES imputed SNP dataset labelled by country of sampling . Two letter population codes are the same as in Figure 23. POPRES samples without matching sampling locations for the Bronze Age samples are shown in light grey, with median positions in larger labelled circles. Bronze Age samples shown as dark grey points.

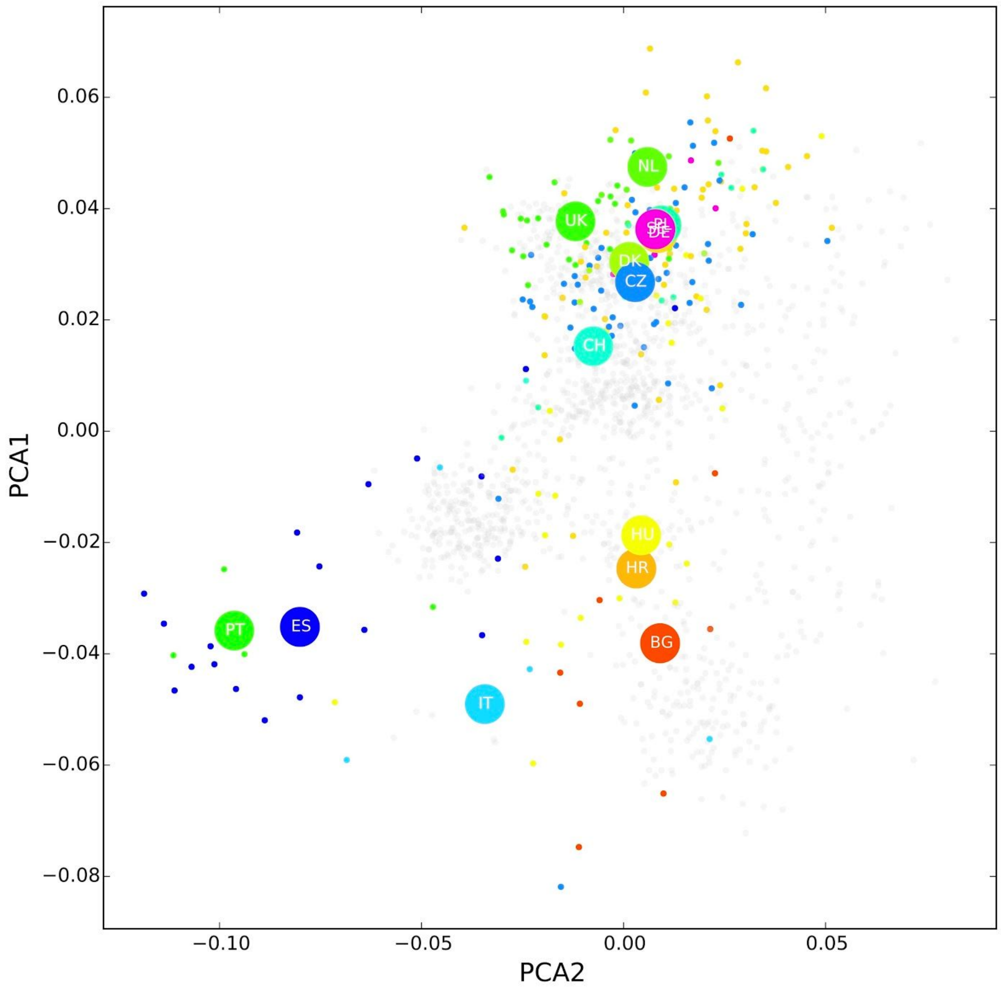

Supplementary Figure 74. Procrustes transformed PCA of Bronze Age samples labelled by country of sampling against POPRES imputed SNP dataset. Two letter population codes are the same as in Supplementary Figure 23 but apply to Bronze Age samples. POPRES samples shown as light grey points.

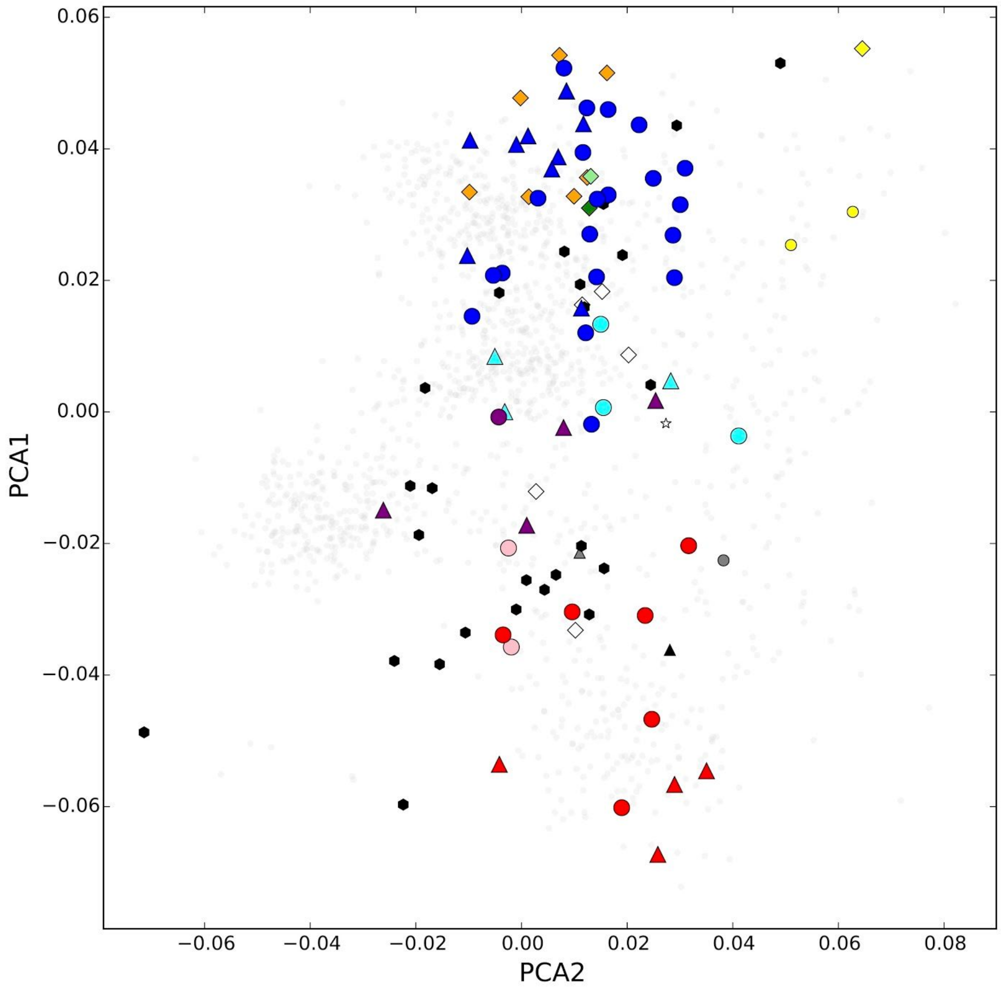

Supplementary Figure 75. Procrustes transformed PCA of medieval samples and Bronze Age Hungarian samples against POPRES imputed SNP dataset. Color coding of medieval samples is same as in Figs 1 and 2. POPRES samples shown in light grey. Bronze Age Hungarians are shown with black hexagons.

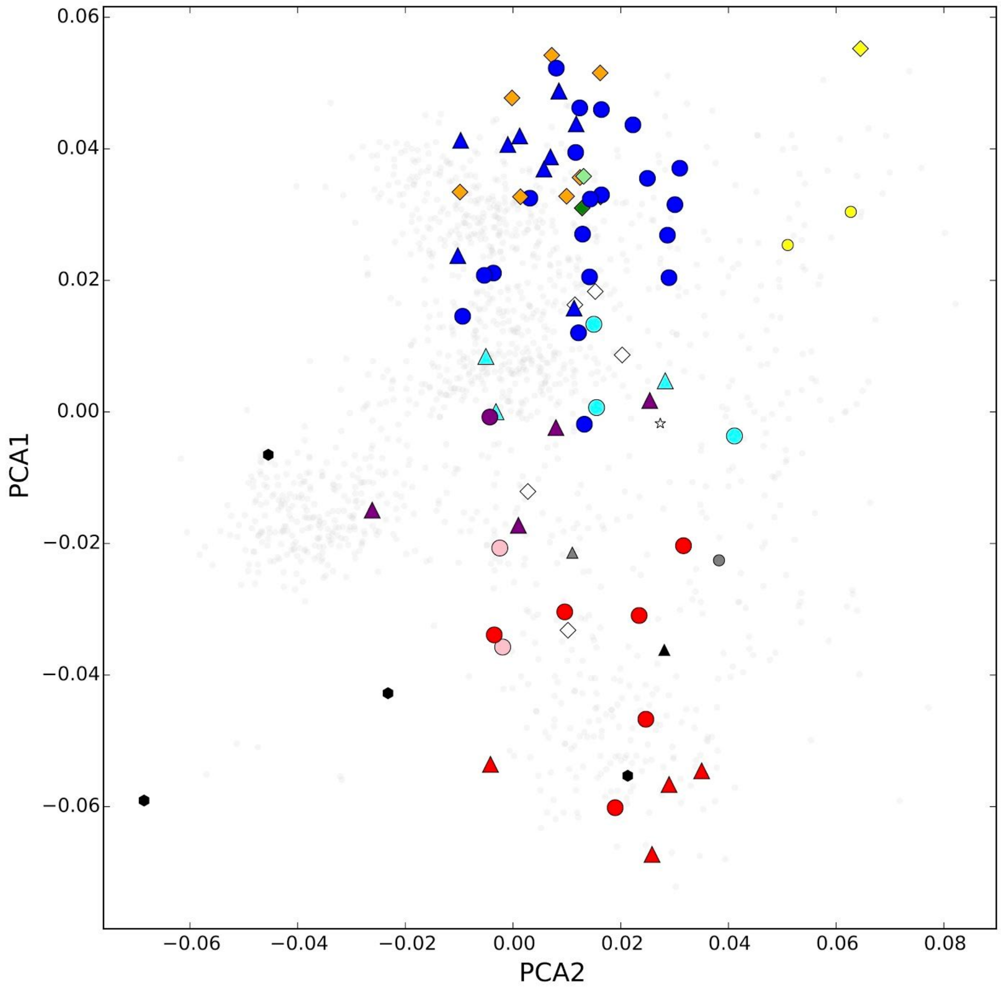

Supplementary Figure 76. Procrustes transformed PCA of medieval samples and Bronze Age Italian samples against POPRES imputed SNP dataset. Color coding of medieval samples is same as in Figs 1 and 2. POPRES samples shown in light grey. Bronze Age Italians are showns with black hexagons.

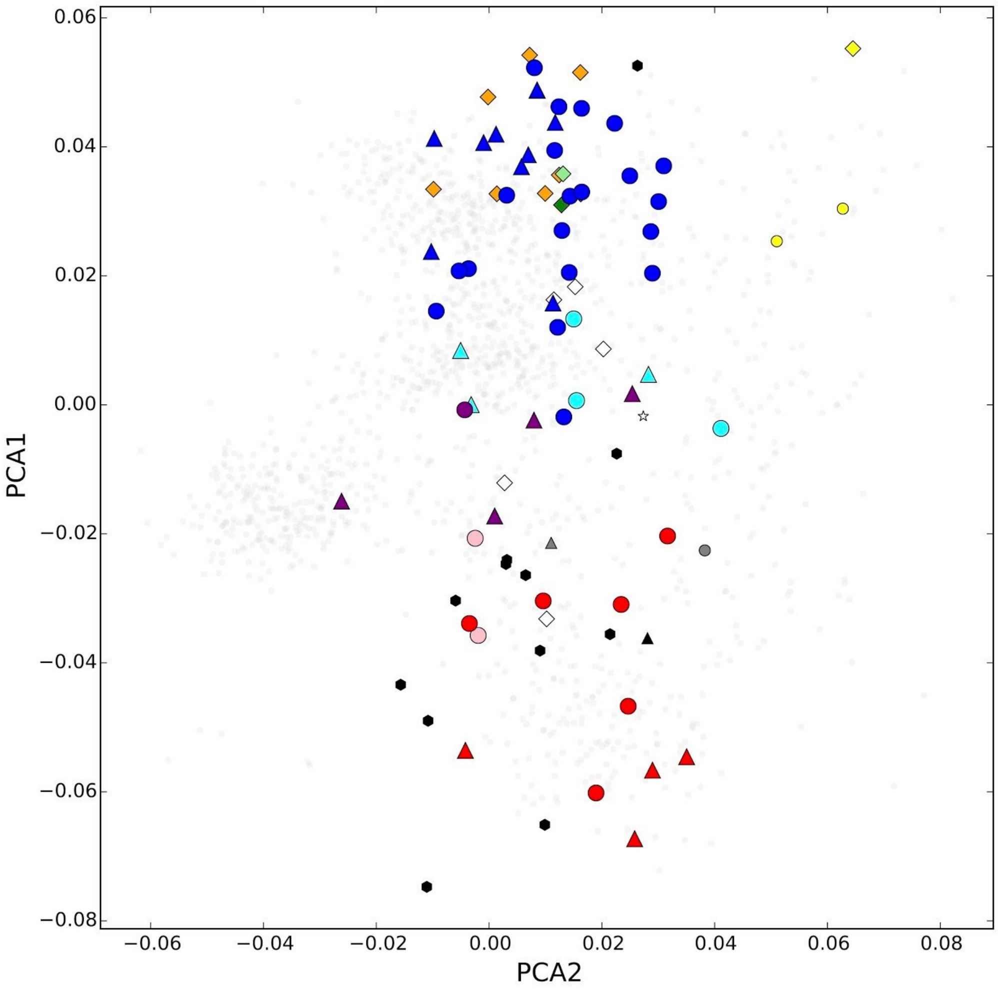

Supplementary Figure 77. Procrustes transformed PCA of medieval samples and Bronze Age southeastern European samples against POPRES imputed SNP dataset. Color coding of medieval samples is same as in Figs 1 and 2. POPRES samples shown in light grey. Bronze Age southeastern Europeans are shown with black hexagons.

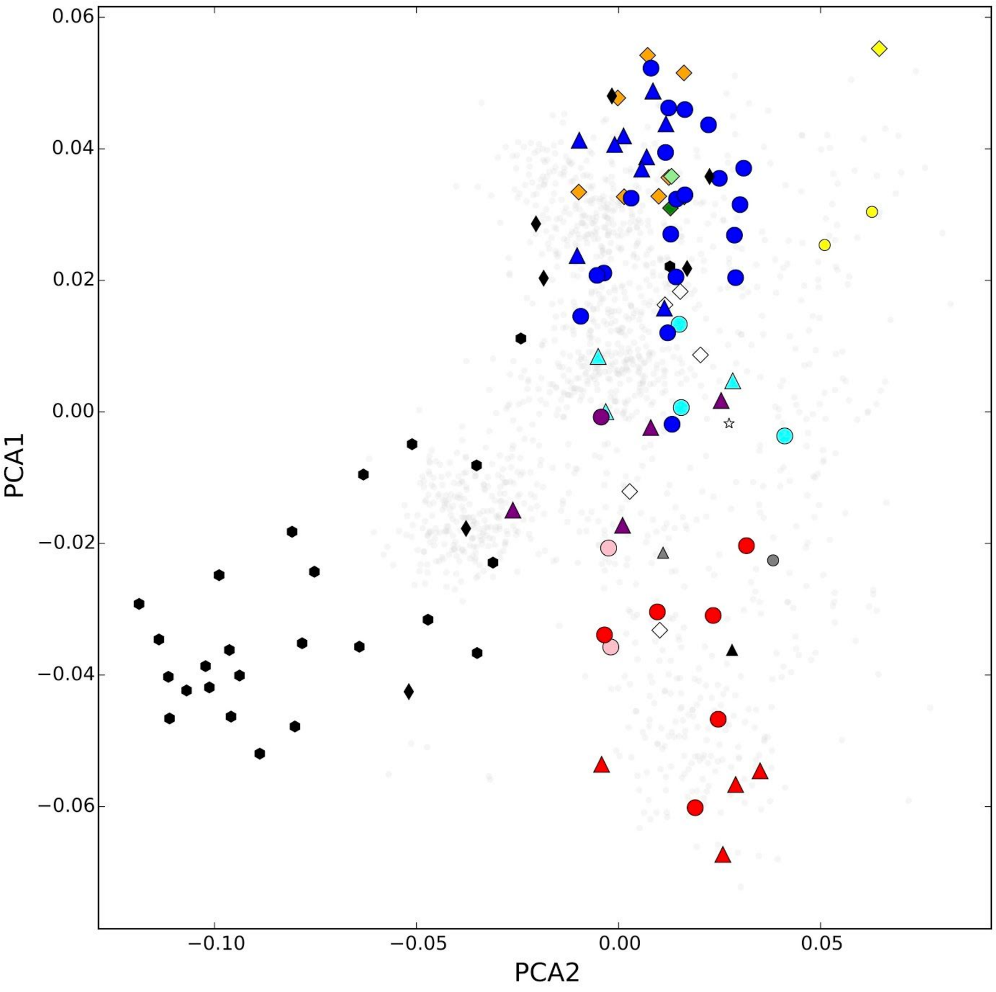

Supplementary Figure 78. Procrustes transformed PCA of medieval samples and Bronze Age western European samples against POPRES imputed SNP dataset. Color coding of medieval samples is same as in Figs 1 and 2. POPRES samples shown in light grey. Bronze Age Iberians and French an are shown with black hexagons and thin black diamonds respectively.

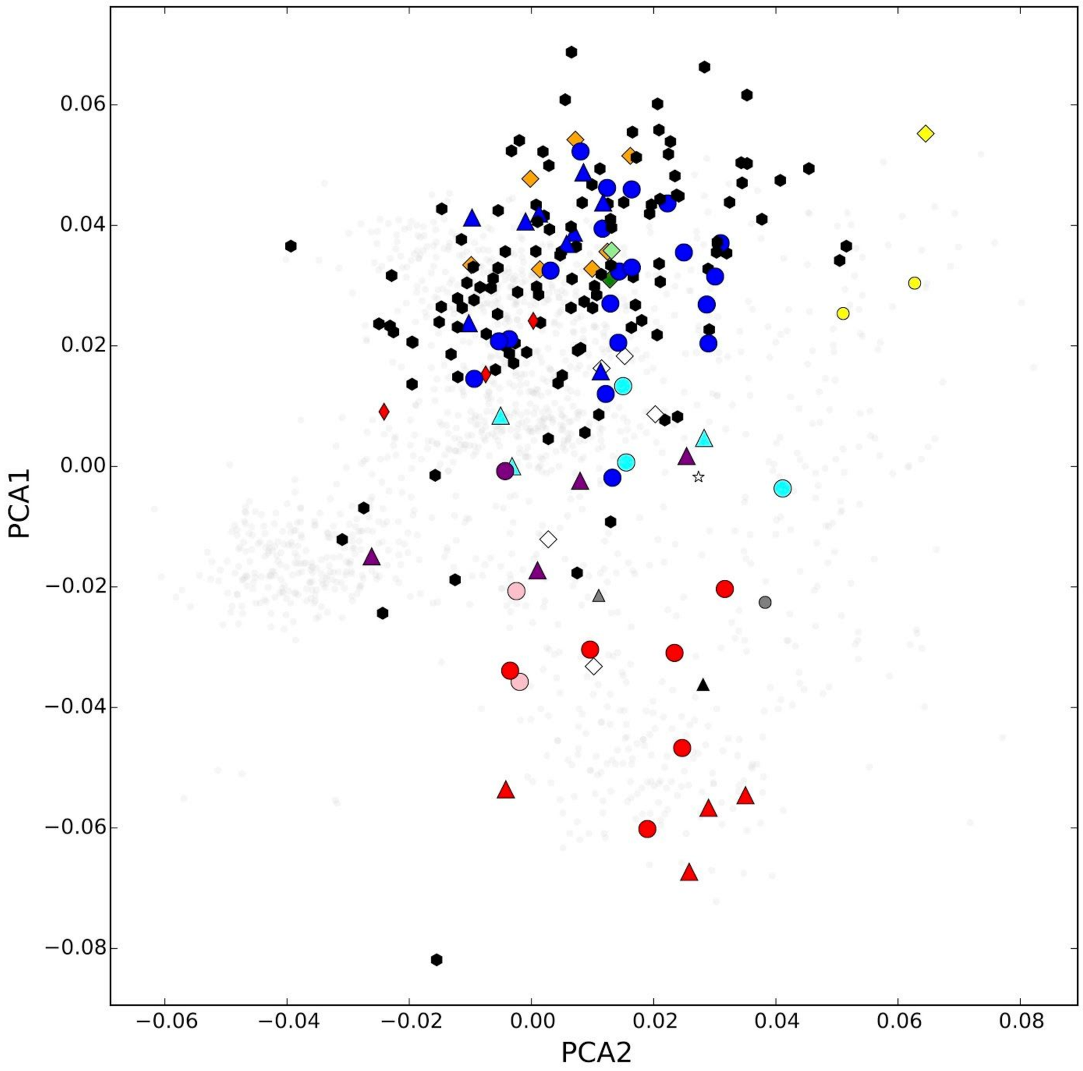

Supplementary Figure 79. Procrustes transformed PCA of medieval samples and Bronze Age central European samples against POPRES imputed SNP dataset. Color coding of medieval samples is same as in Figs 1 and 2. POPRES samples shown in light grey. Bronze Age central Europeans from Germany and Czech Republic are shown with black hexagons, three from Switzerland shown with red diamonds.

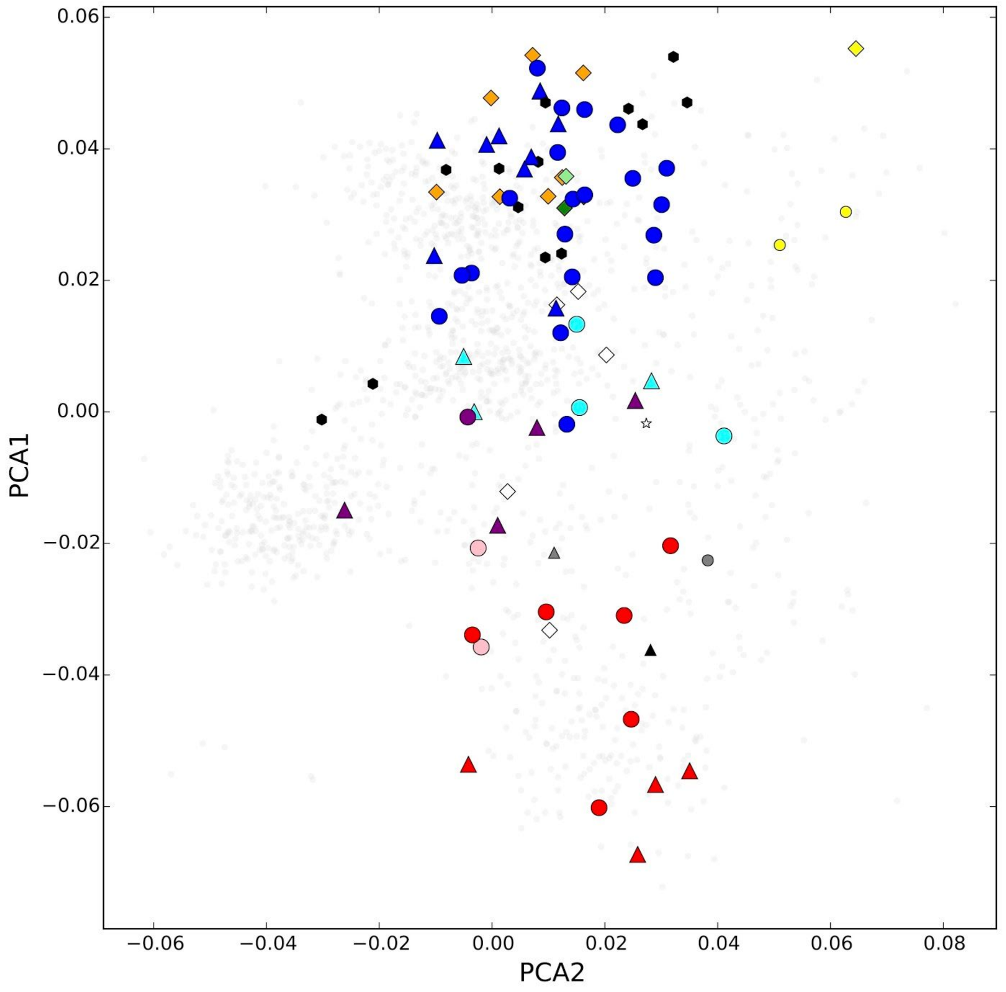

Supplementary Figure 80. Procrustes transformed PCA of medieval samples and Bronze Age eastern European samples against POPRES imputed SNP dataset. Color coding of medieval samples is same as in Figs 1 and 2. POPRES samples shown in light grey. Bronze Age eastern Europeans are shown with black hexagons.

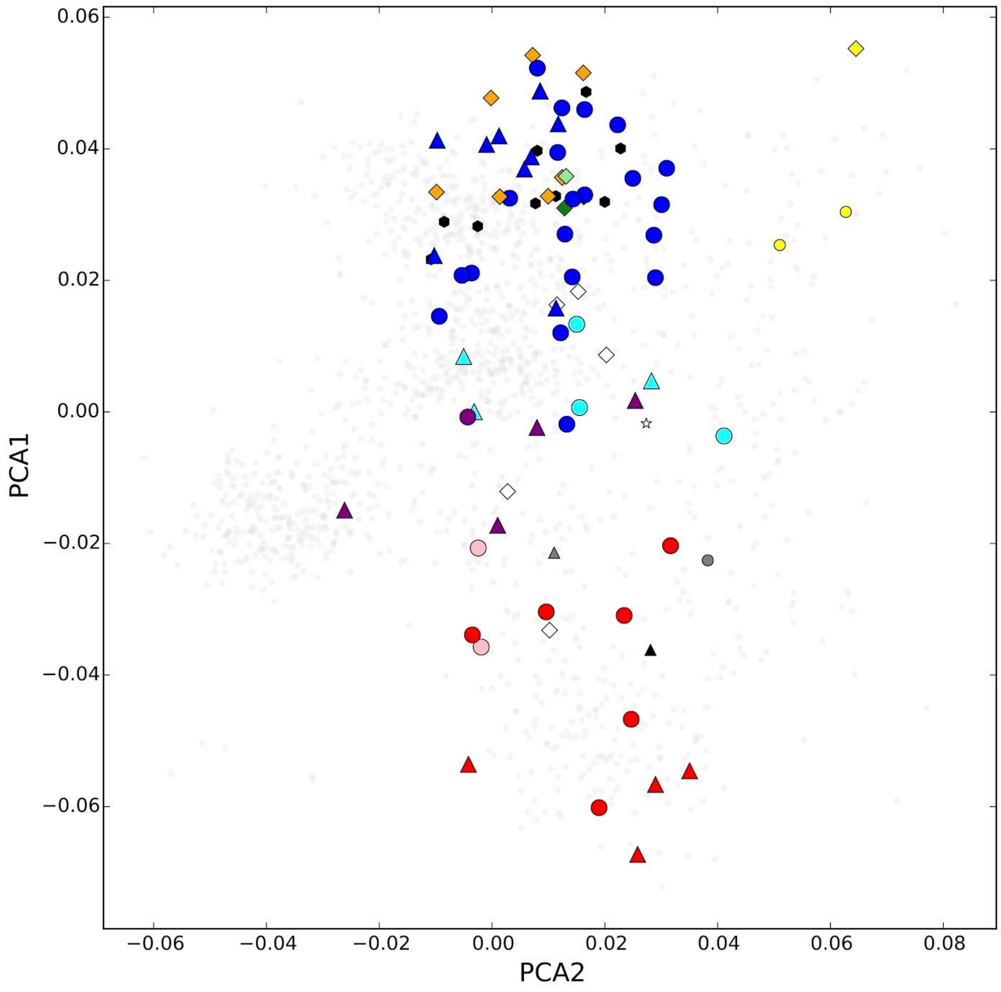

Supplementary Figure 81. Procrustes transformed PCA of medieval samples and Bronze Age northern European samples against POPRES imputed SNP dataset. Color coding of medieval samples is same as in Figs 1 and 2. POPRES samples shown in light grey. Bronze Age northern Europeans are shown with black hexagons.

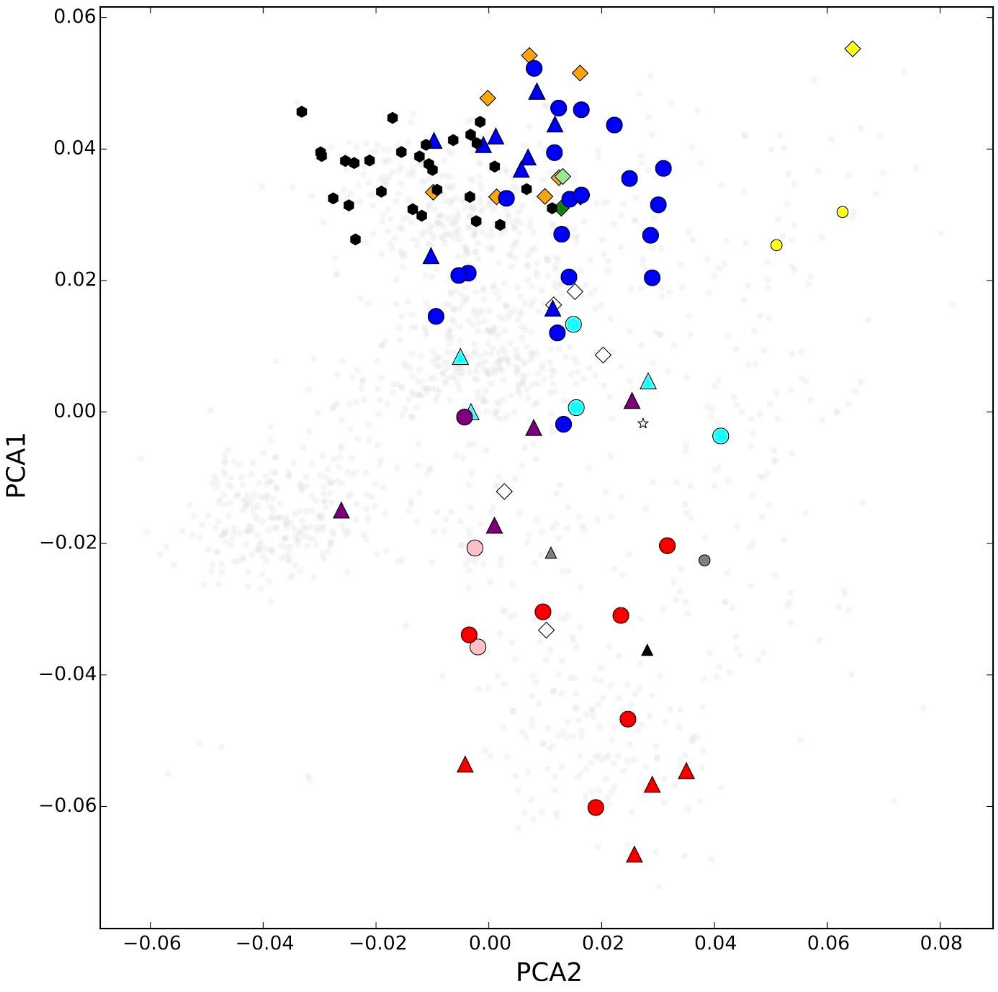

Supplementary Figure 82. Procrustes transformed PCA of medieval samples and Bronze Age northwestern European samples against POPRES imputed SNP dataset. Color coding of medieval samples is same as in Figs 1 and 2. POPRES samples shown in light grey. Bronze Age northwestern Europeans are shown with black hexagons.

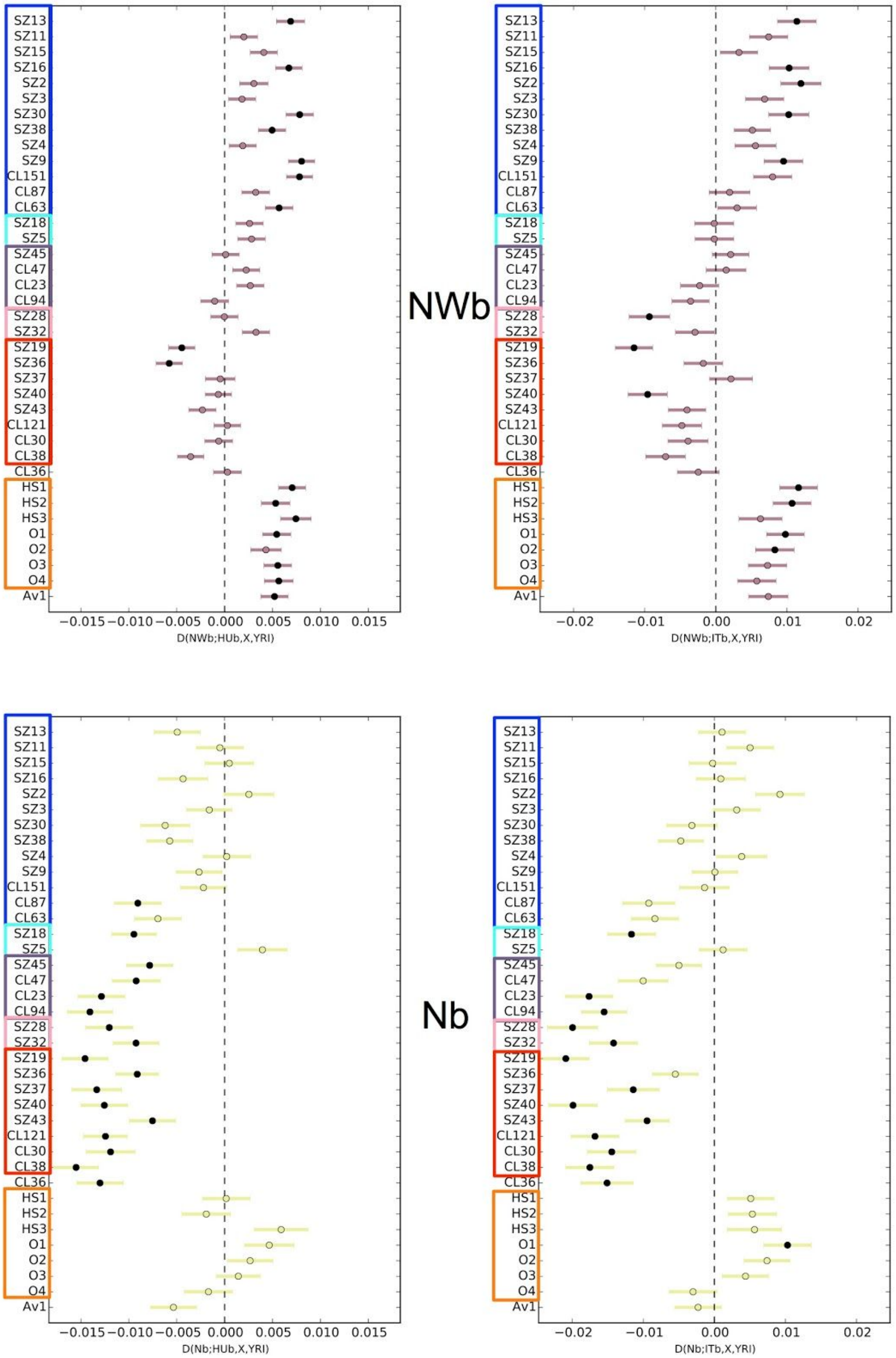

Supplementary Figure 83. Results of D-statistic test where each unrelated medieval sample is compared to a clade containing either Bronze Age Hungarians (left column) and Italians (right column) versus other test Bronze Age European regions. Positive score means medieval sample is closer to test population, negative score means medieval sample is closer to Hungarians/Italians. Error bars indicate standard error of the mean (s.e.m). Z score > | 3 | shown with black mean. Sample labels are highlighted with boxes according to Supplementary Figure 51.

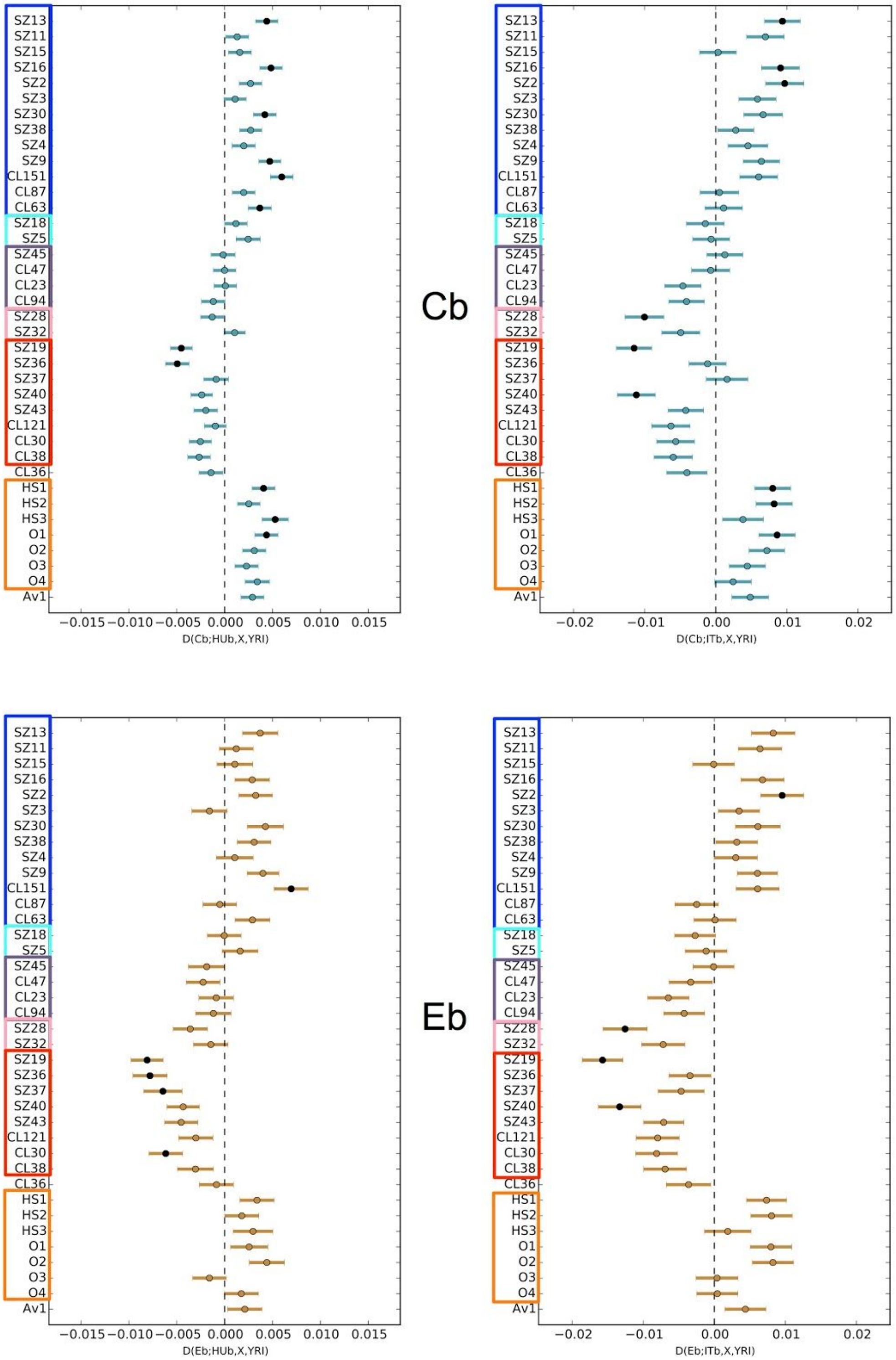

Supplementary Figure 83 continued. Results of D-statistic test where each unrelated medieval sample is compared to a clade containing either Bronze Age Hungarians (left column) and Italians (right column) versus other test Bronze Age European regions. Positive score means medieval sample is closer to test population, negative score means medieval sample is closer to Hungarians/Italians. Error bars indicate standard error of the mean (s.e.m). Z score > | 3 | shown with black mean. Sample labels are highlighted with boxes according to Supplementary Figure 51.

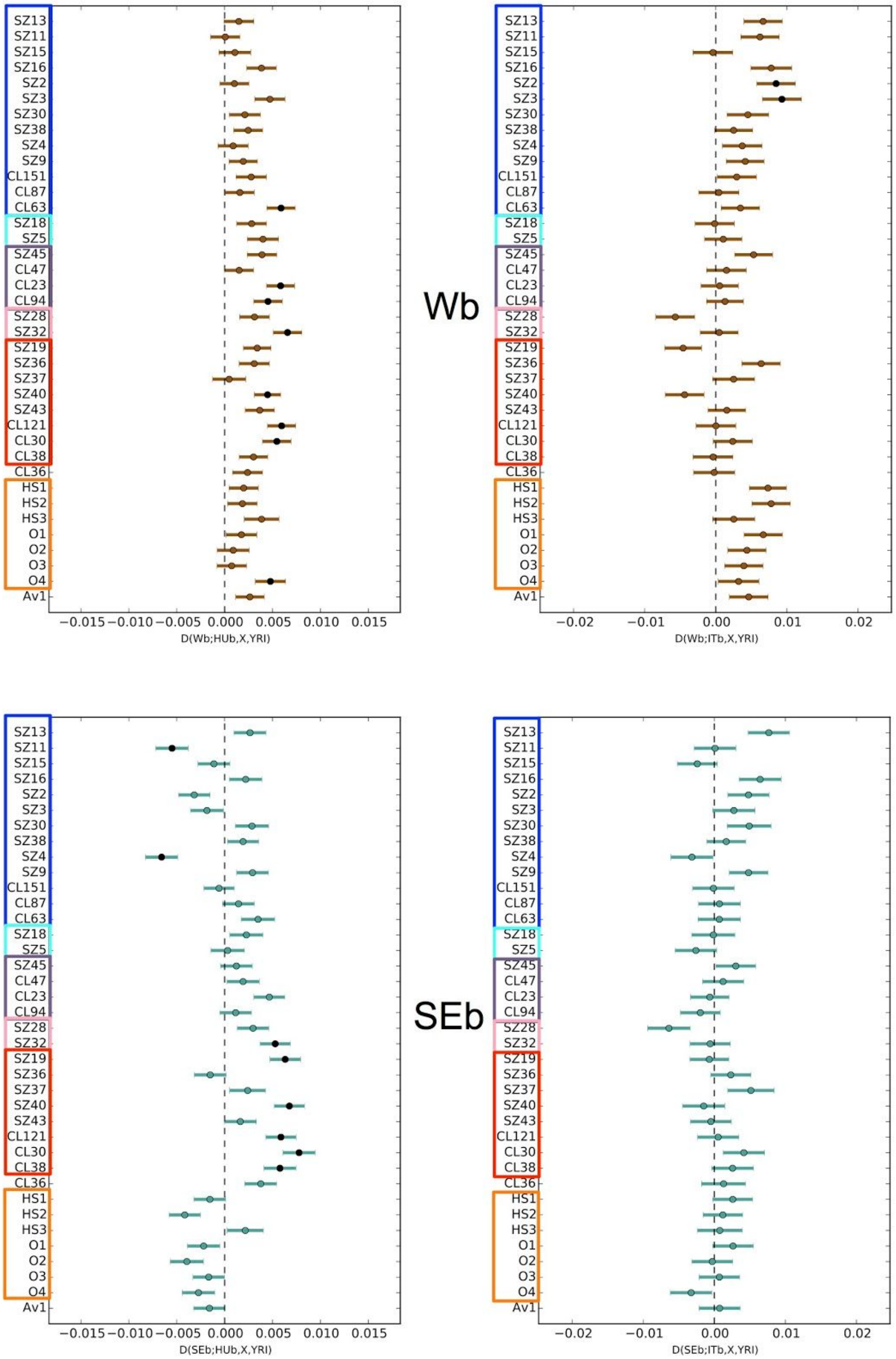

Supplementary Figure 83 continued. Results of D-statistic test where each unrelated medieval sample is compared to a clade containing either Bronze Age Hungarians (left column) and Italians (right column) versus other test Bronze Age European regions. Positive score means medieval sample is closer to test population, negative score means medieval sample is closer to Hungarians/Italians. Error bars indicate standard error of the mean (s.e.m). Z score > | 3 | shown with black mean. Sample labels are highlighted with boxes according to Supplementary Figure 51.

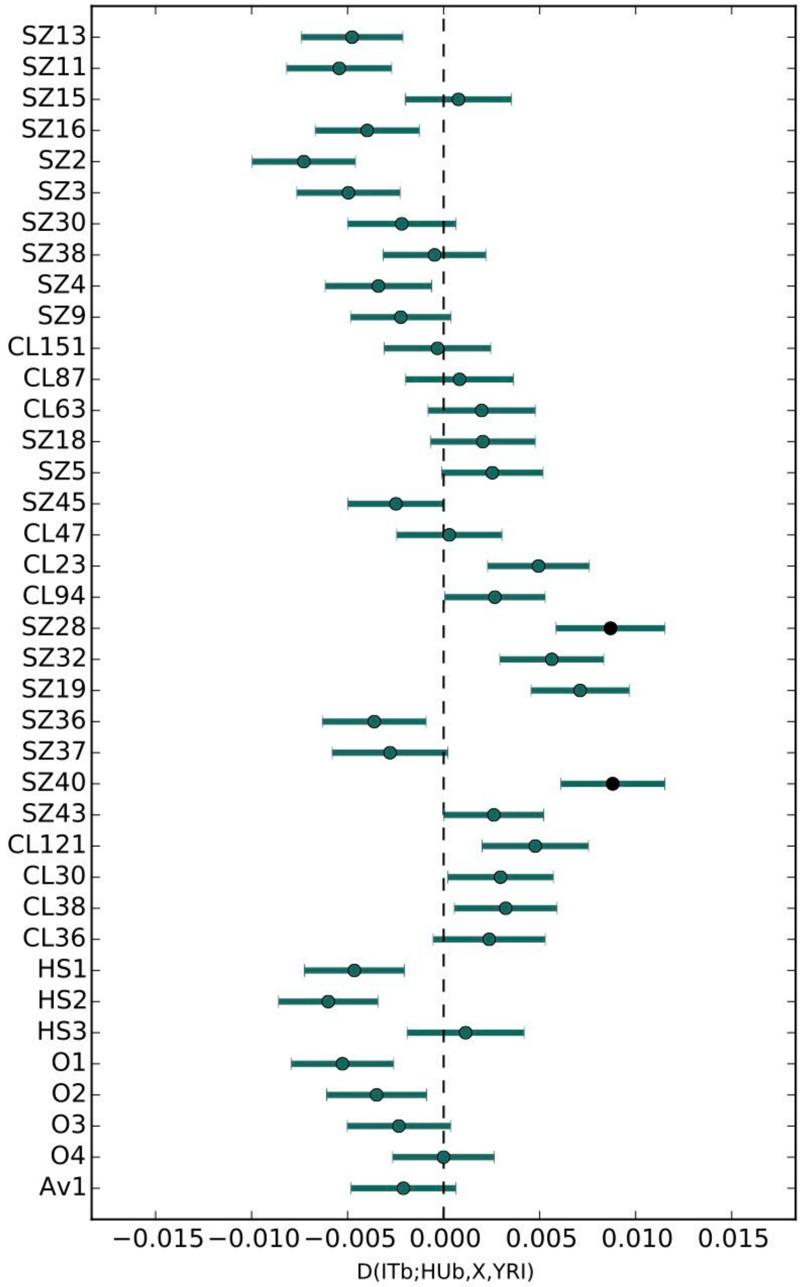

Supplementary Figure 84. Results of D-statistic test where each unrelated medieval sample is compared to a clade containing Bronze Age Italians versus Hungarians. Positive score means medieval sample is closer to Italians, negative score means scample is closer to Hungarians. Error bars indicate standard error of the mean (s.e.m). Z score > | 3 | shown with black mean.

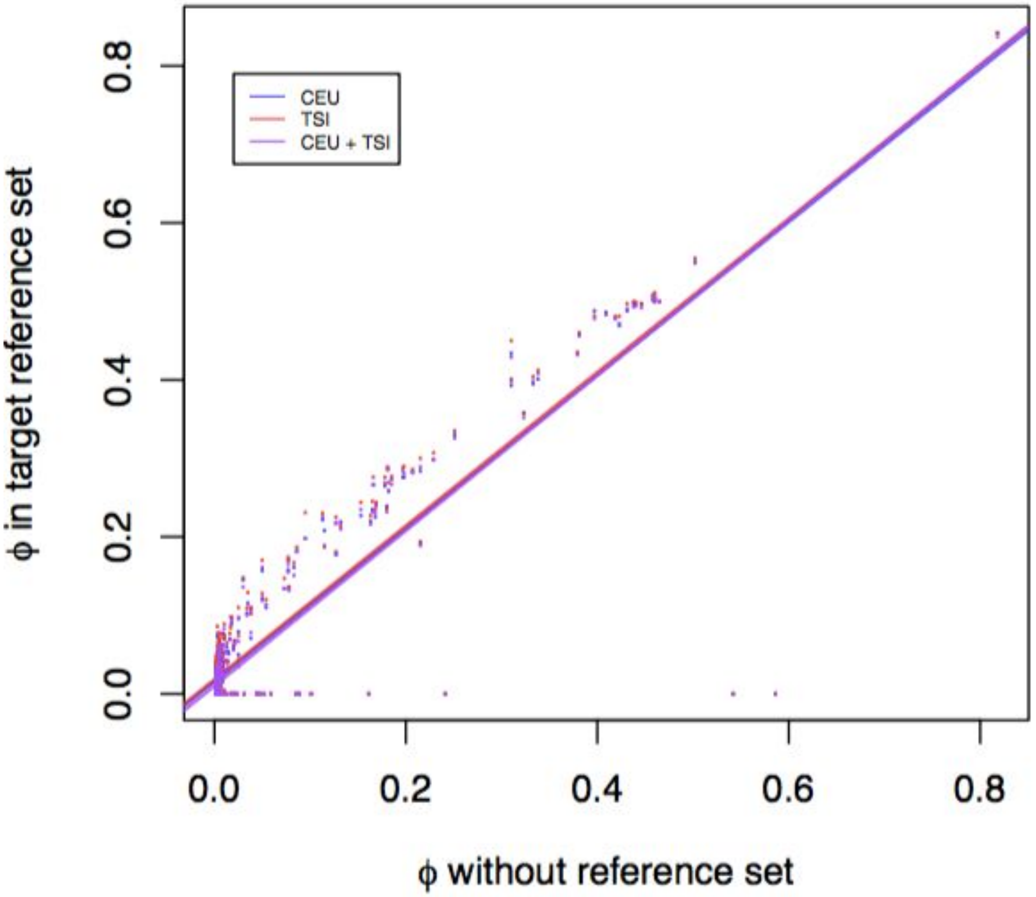

Supplementary Figure 85. Distribution of kinship coefficient ϕ inferred using different population reference sets (CEU, TSI or both merged) against values inferred using no reference set (i.e. estimating allele frequencies from the target ancient samples).

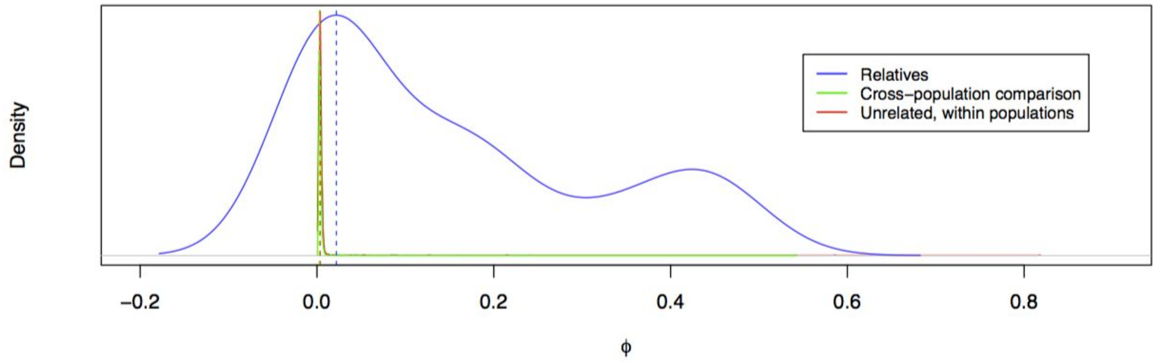

Supplementary Figure 86. Distribution of kinship coefficient ϕ for pairs of individuals from different cemeteries (green curve), for potentially unrelated individuals from the same cemetery (red curve), and for potentially related individuals (blue curve).

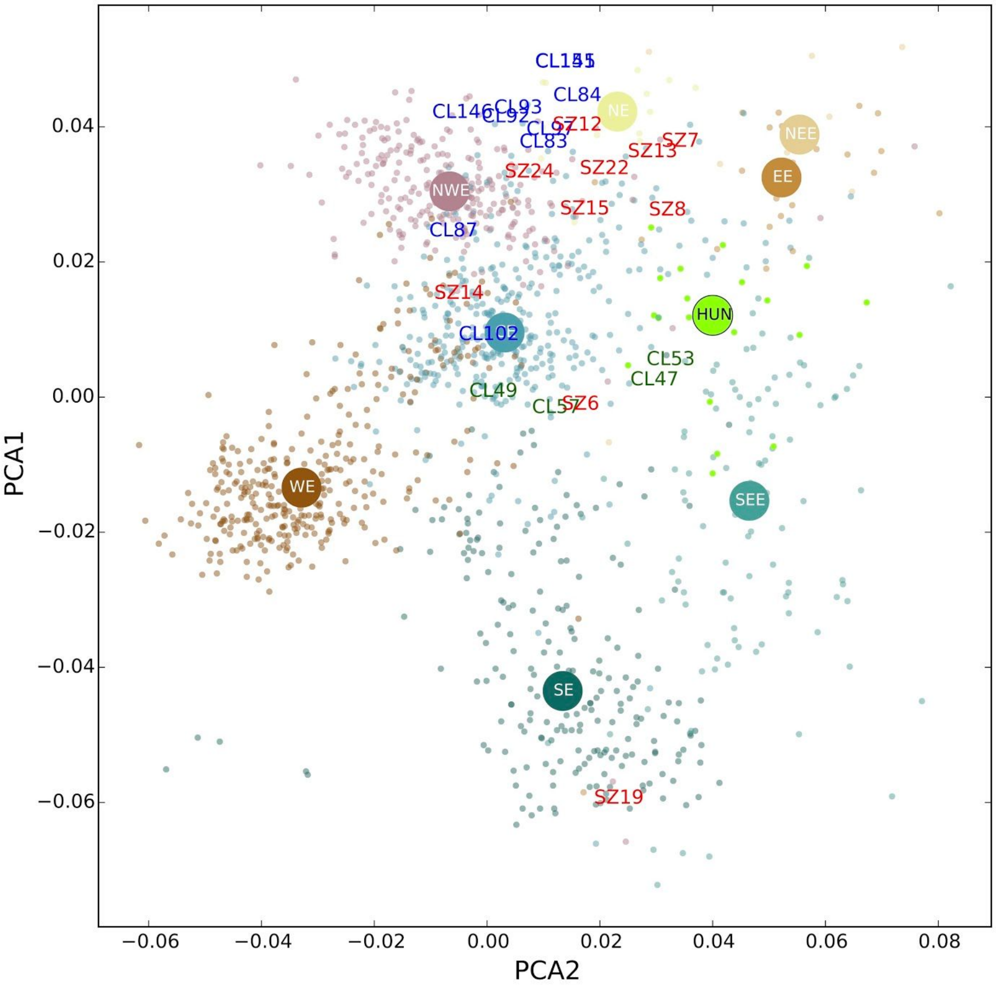

Supplementary Figure 87. Procrustes transformed PCA of three major medieval ancient kindred against POPRES imputed SNP dataset. Kindred SZ1 = red , Kinded CL1 = blue, Kindred CL2 = green.

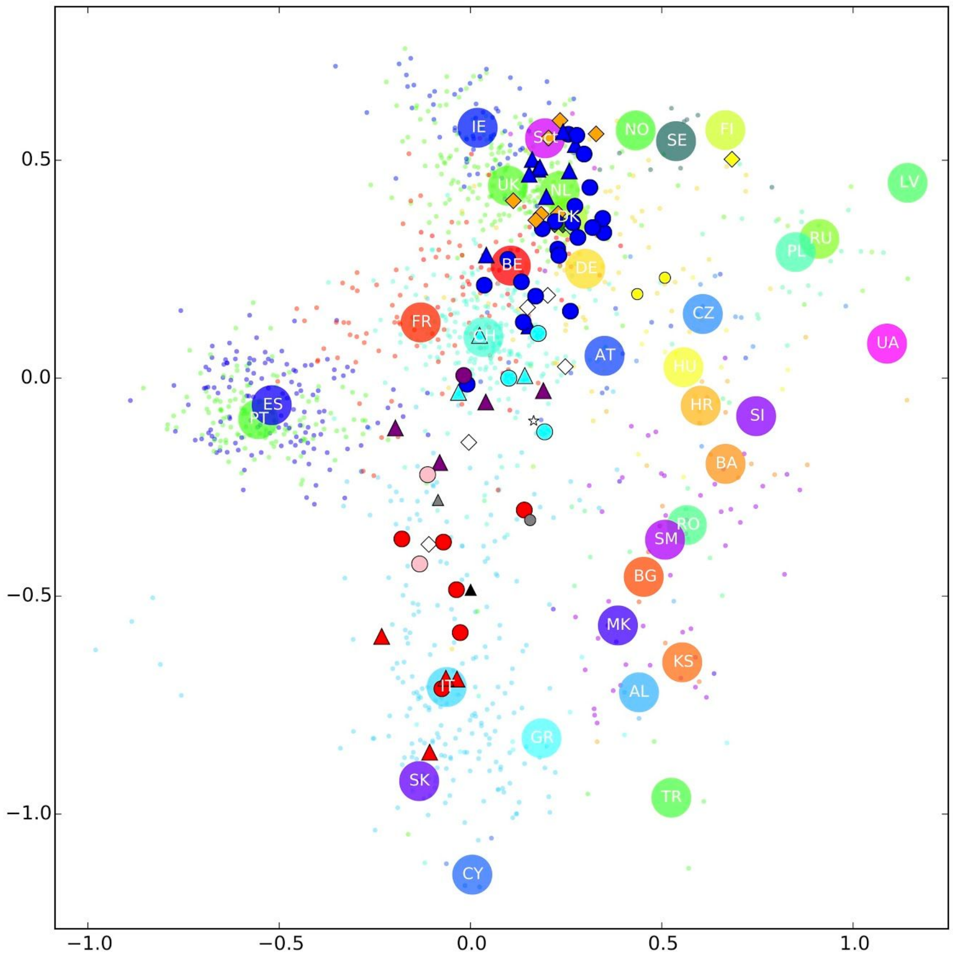

Supplementary Figure 88. SPA analysis allowing 2-dimensional coordinates for diploid POPRES individuals and haploid medieval samples assuming a single parental origin.

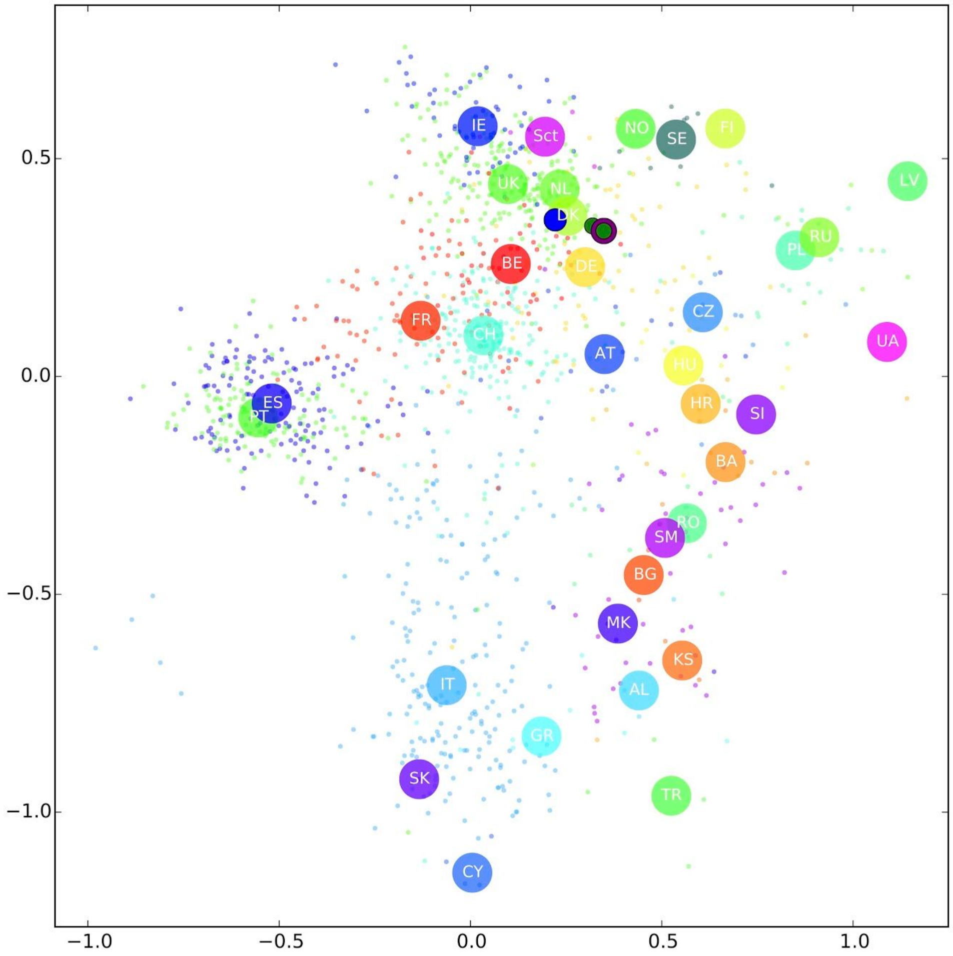

Supplementary Figure 89. SPA-based analysis of unknown parent for parent/avuncular (SZ24) and offspring (SZ13, SZ22). Known parent = blue filled circle, unknown parent = purple filled circle, offspring = green filled circles.

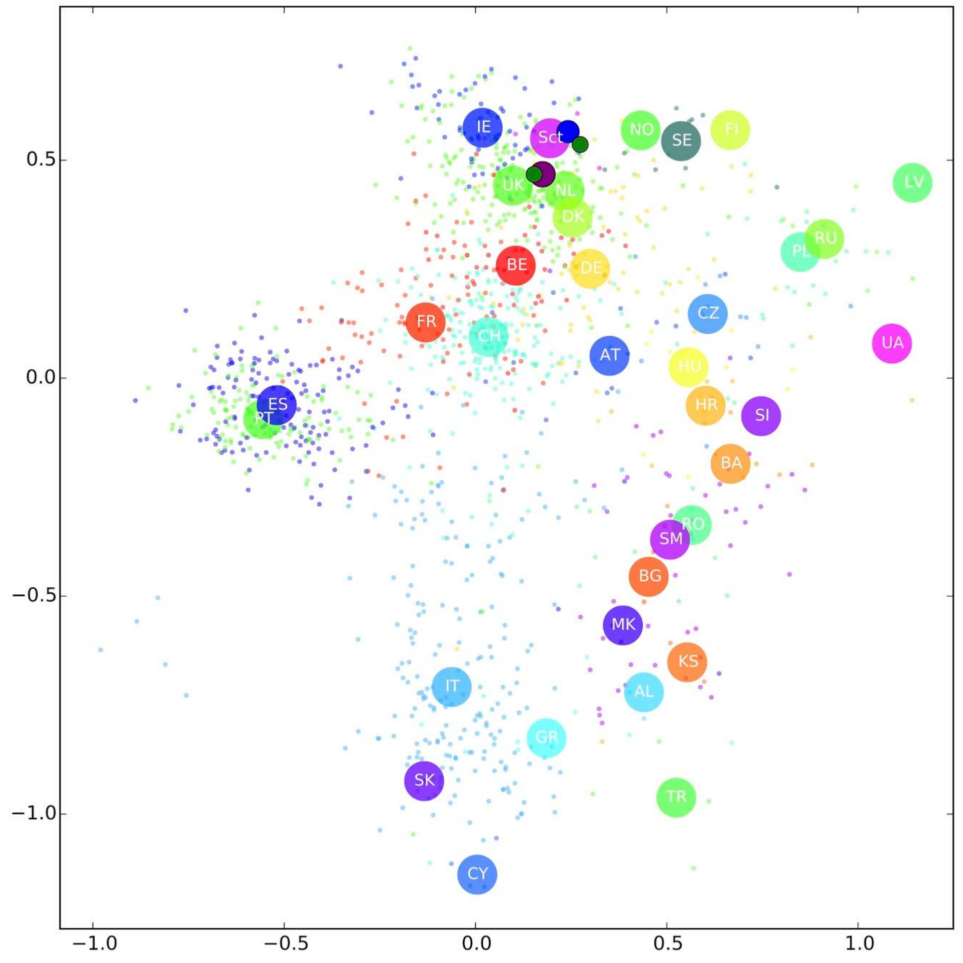

Supplementary Figure 90. SPA-based analysis of unknown parent for parent/avuncular (CL151) and offspring (CL145, CL146). Known parent = blue filled circle, unknown parent = purple filled circle, offspring = green filled circles.

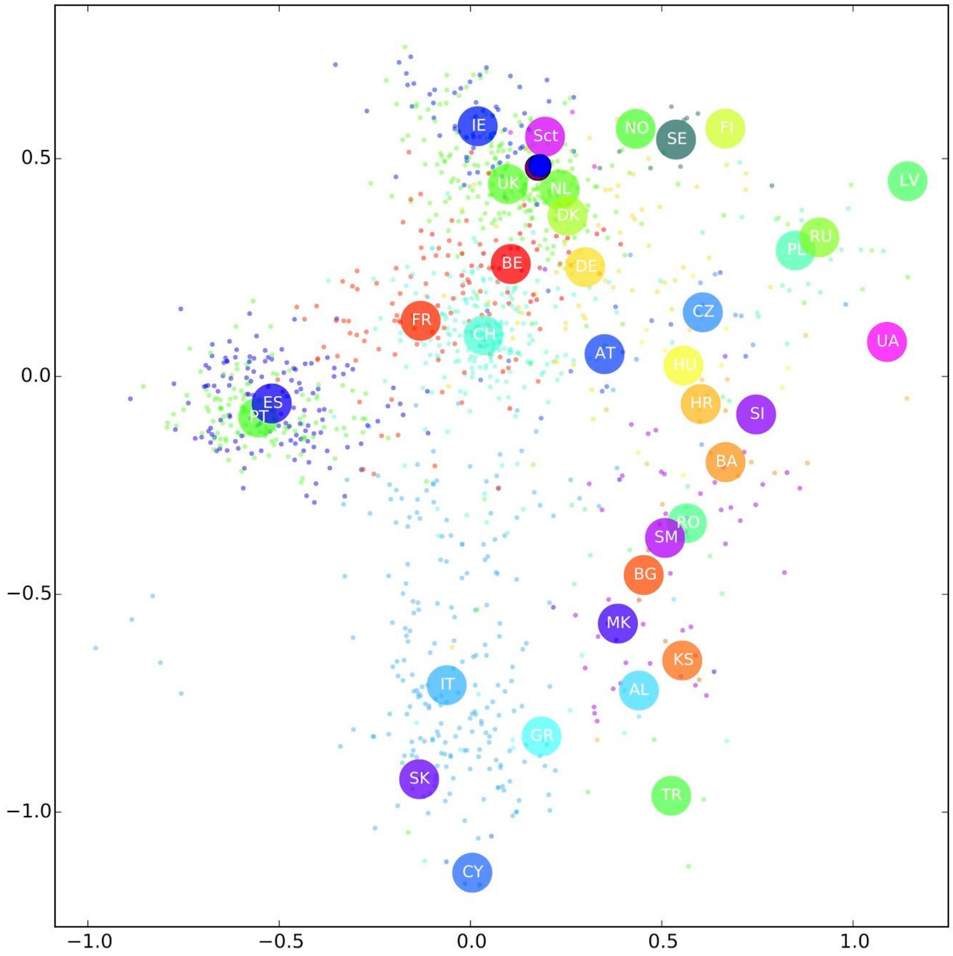

Supplementary Figure 91. SPA-based analysis of unknown parent for parent/avuncular (CL93) and offspring (CL92). Known parent = blue filled circle, unknown parent = purple filled circle, offspring = green filled circles.

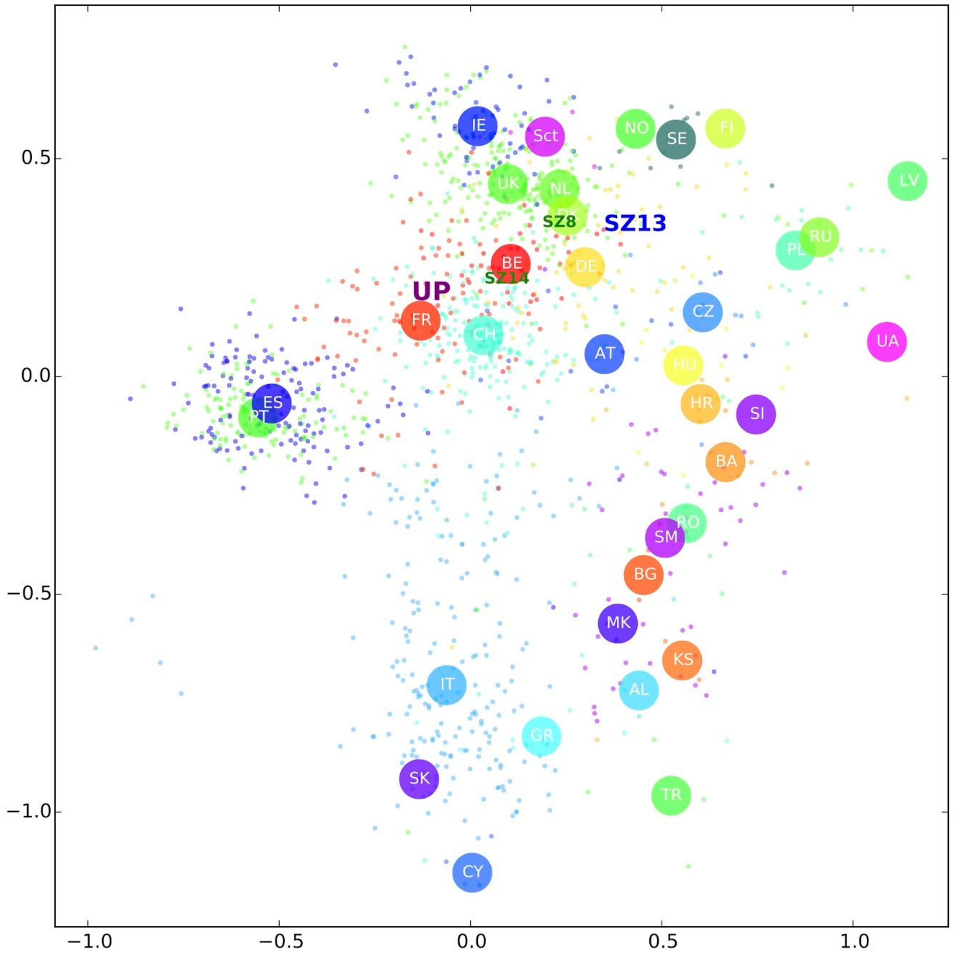

Supplementary Figure 92. SPA-based analysis of unknown parent for parent/avuncular (SZ13) and offspring (SZ8, SZ14). Known parent = blue, unknown parent = purple UP, offspring = green.

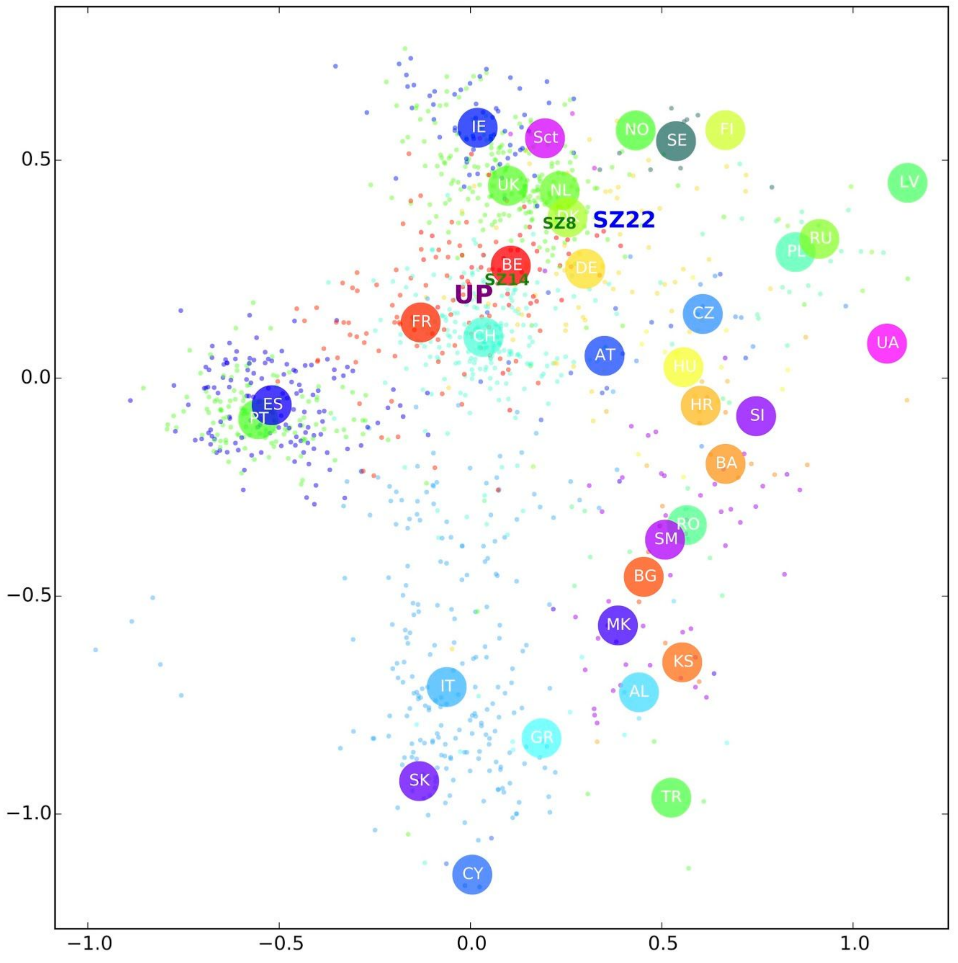

Supplementary Figure 93. SPA-based analysis of unknown parent for parent/avuncular (SZ22) and offspring (SZ8, SZ14). Known parent = blue, unknown parent = purple UP, offspring = green.

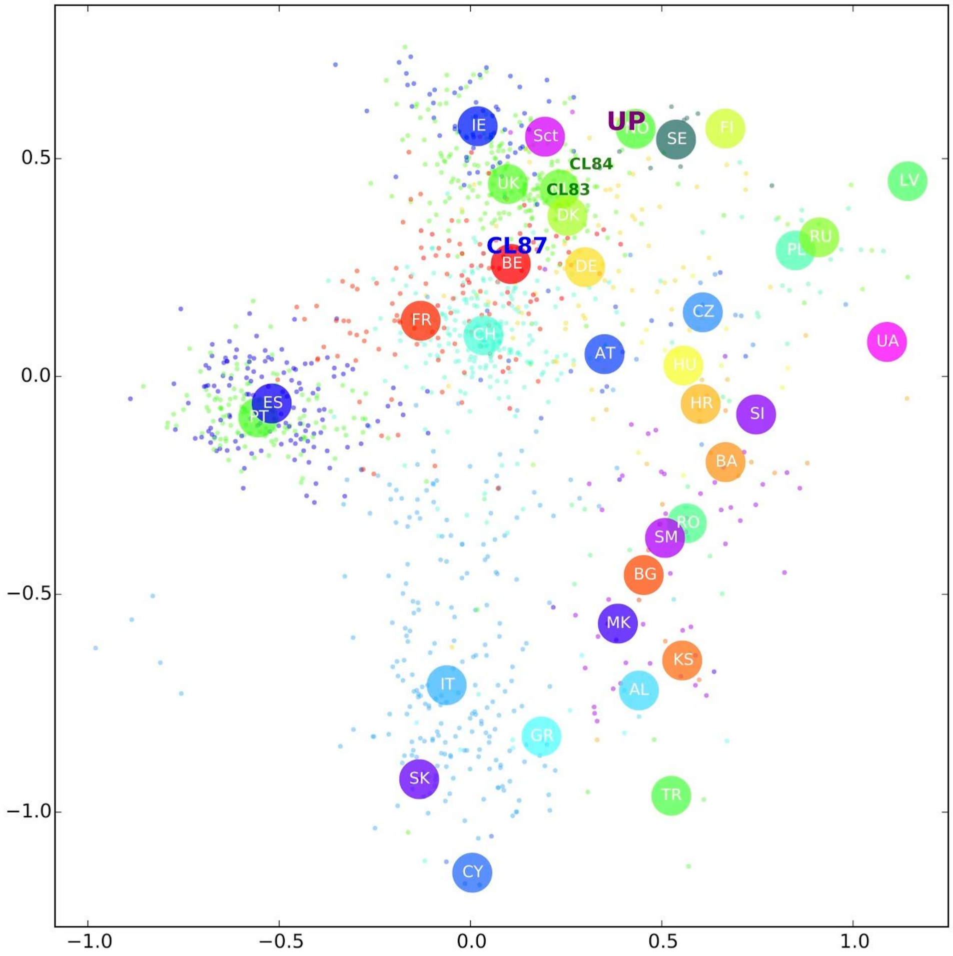

Supplementary Figure 94. SPA-based analysis of unknown parent for parent/avuncular (CL87) and offspring (CL83, CL84). Known parent = blue, unknown parent = purple UP, offspring = green.

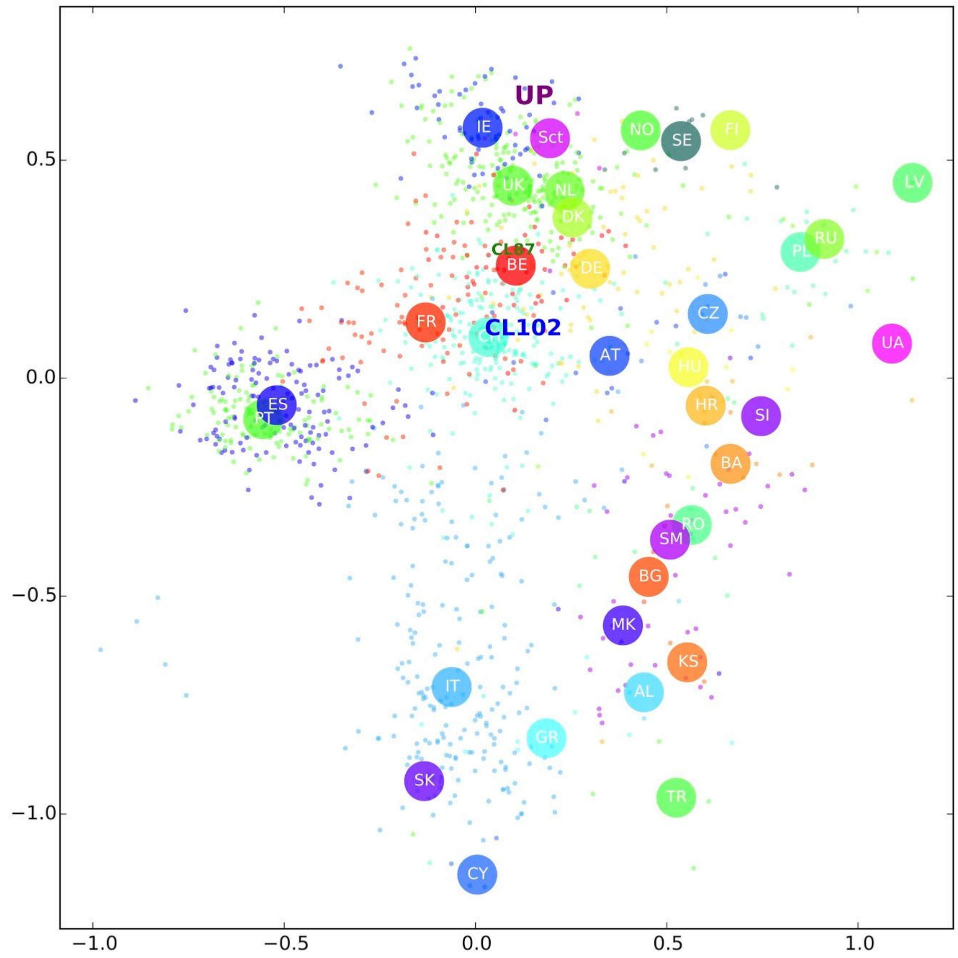

Supplementary Figure 95. SPA-based analysis of unknown parent for parent/avuncular (CL102) and offspring (CL87). Known parent = blue, unknown parent = purple UP, offspring = green.

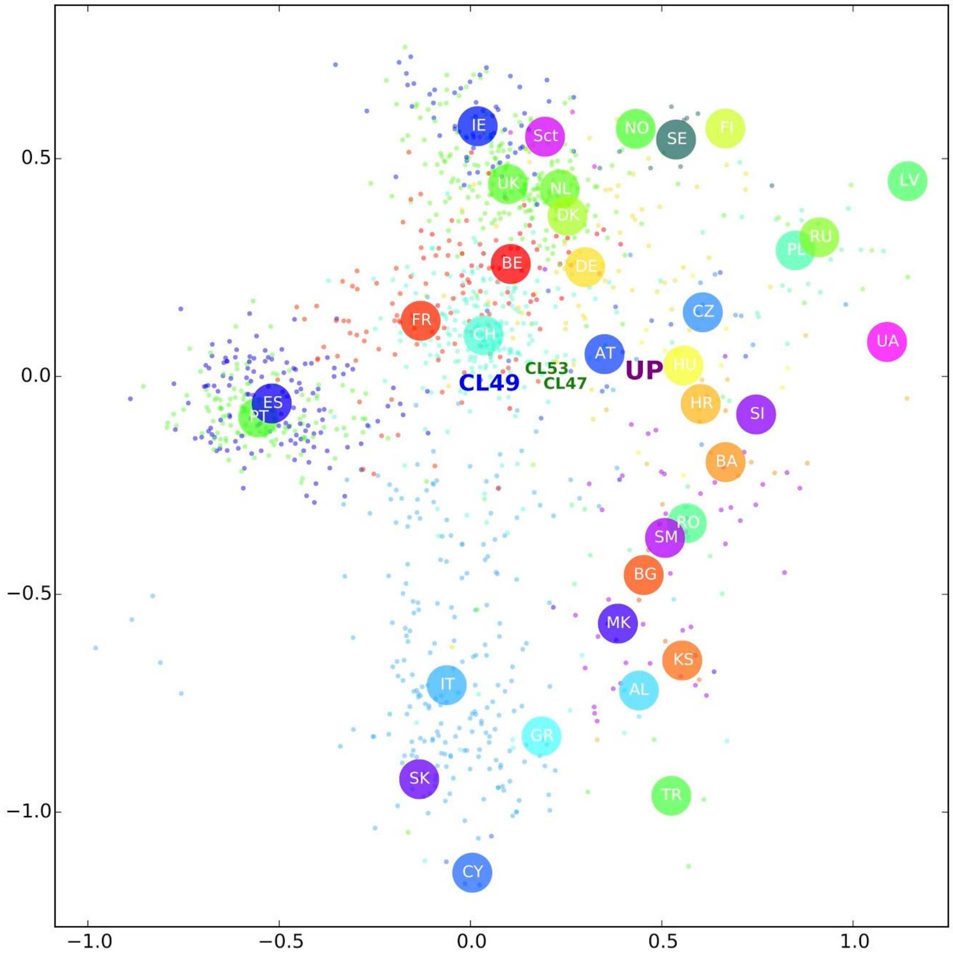

Supplementary Figure 96. SPA-based analysis of unknown parent for parent/avuncular (CL49) and offspring (CL53, CL47). Known parent = blue, unknown parent = purple UP, offspring = green.

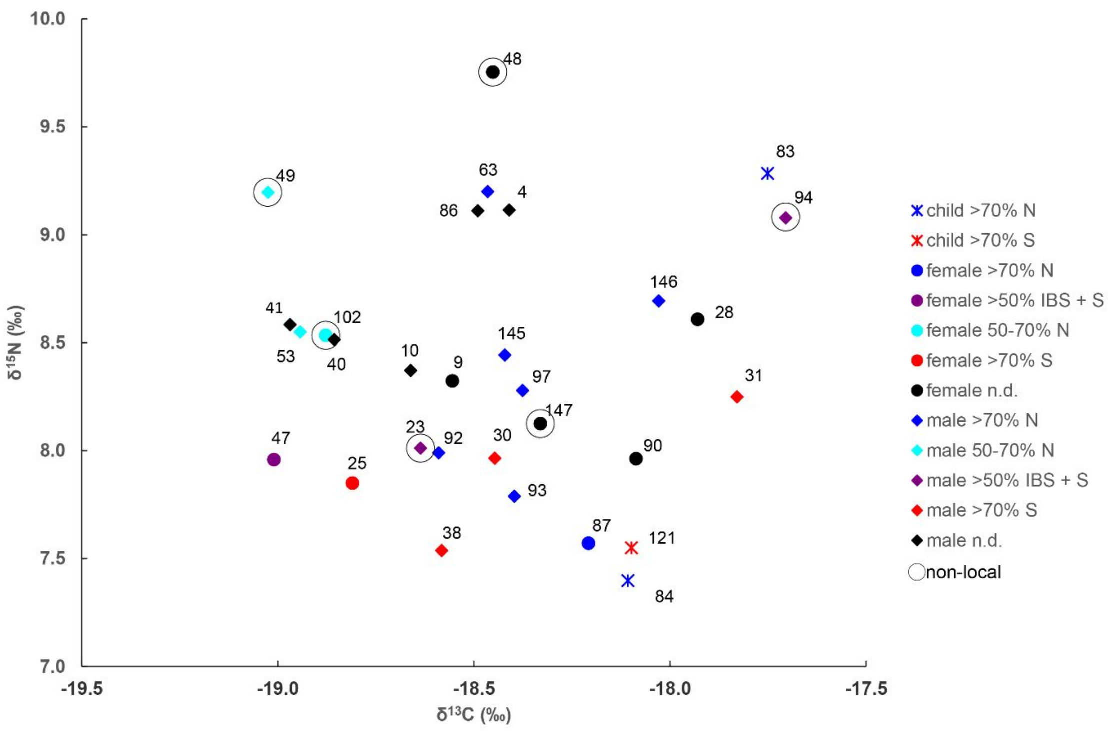

Supplementary Figure 97. Dietary evidence at Collegno, as indicated by $\delta^{13}C_{coll}$ and $\delta^{15}N$ values of bone collagen. Ancestry groups and migration status are highlighted.

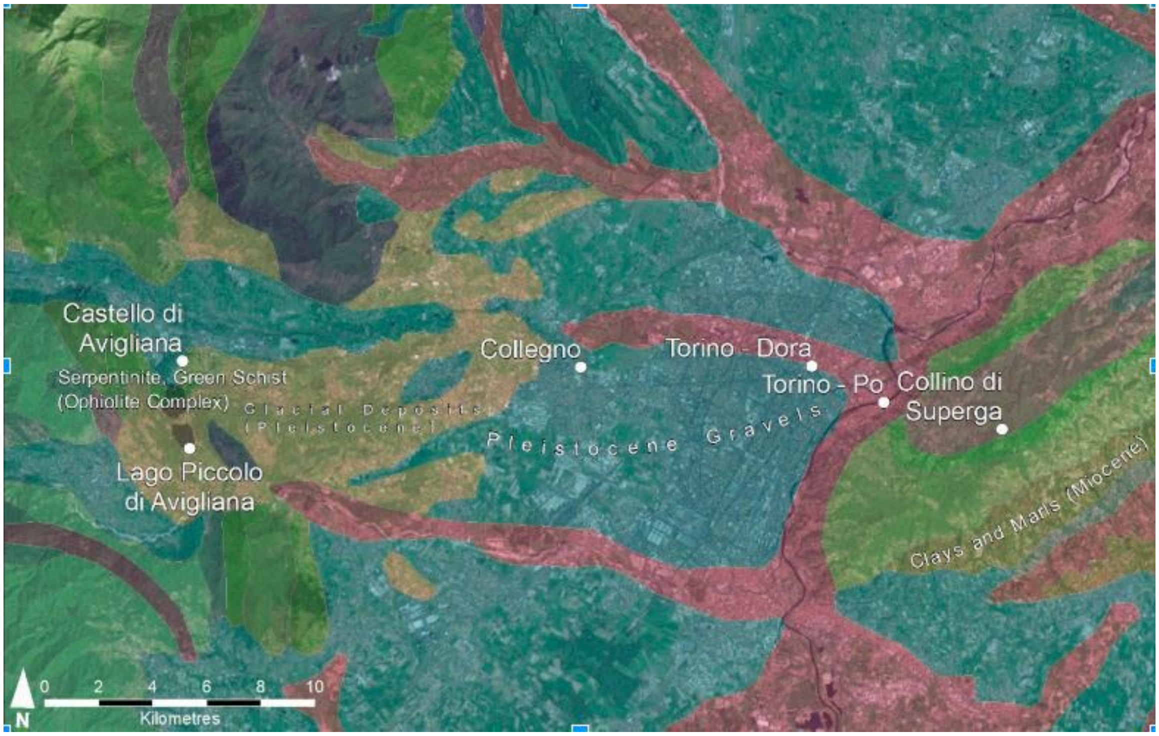

Supplementary Figure 98. Map of the geological environment of Collegno with sampling locations (Drawn by D. Redhouse). Geological data from the Geoportale Nazionale (http://www.pcn.minambiente.it/) under a Creative Commons Attribution --- Share Alike 3.0 Italy Licence. Imagery from ArcGIS 10.2. Source: Esri, DigitalGlobe, GeoEye, Earthstar Geographics, CNES/Airbus DS, USDA, USGS, AEX, Getmapping, Aerogrid, IGN, IGP, swisstopo, and the GIS User Community.

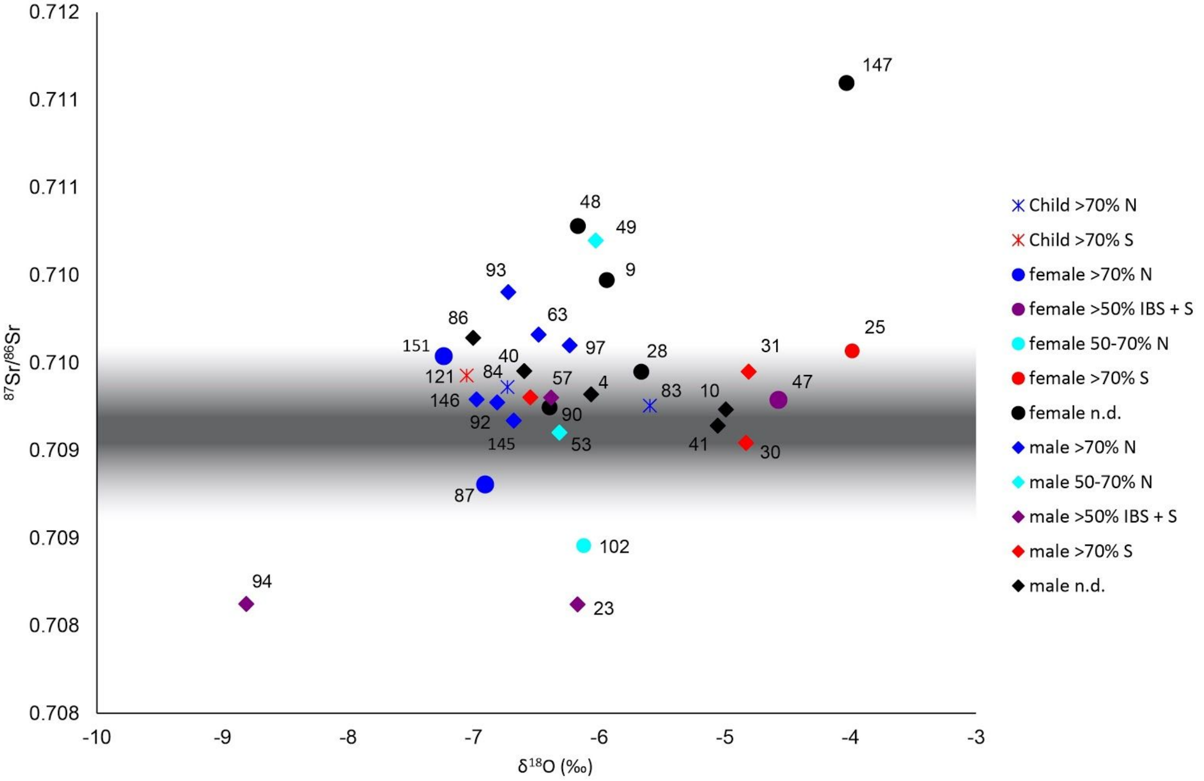

Supplementary Figure 99. Evidence for migrants at Collegno revealed by $^{87}Sr/^{86}Sr$ and $\delta^{18}O$ (‰) values of human tooth enamel. The grey band denotes the local bioavailable strontium range. Key: blue: >70% N; cyan: 50-70% N; red: >70% S; purple: >50% IBS+S; black: no ancestry data.

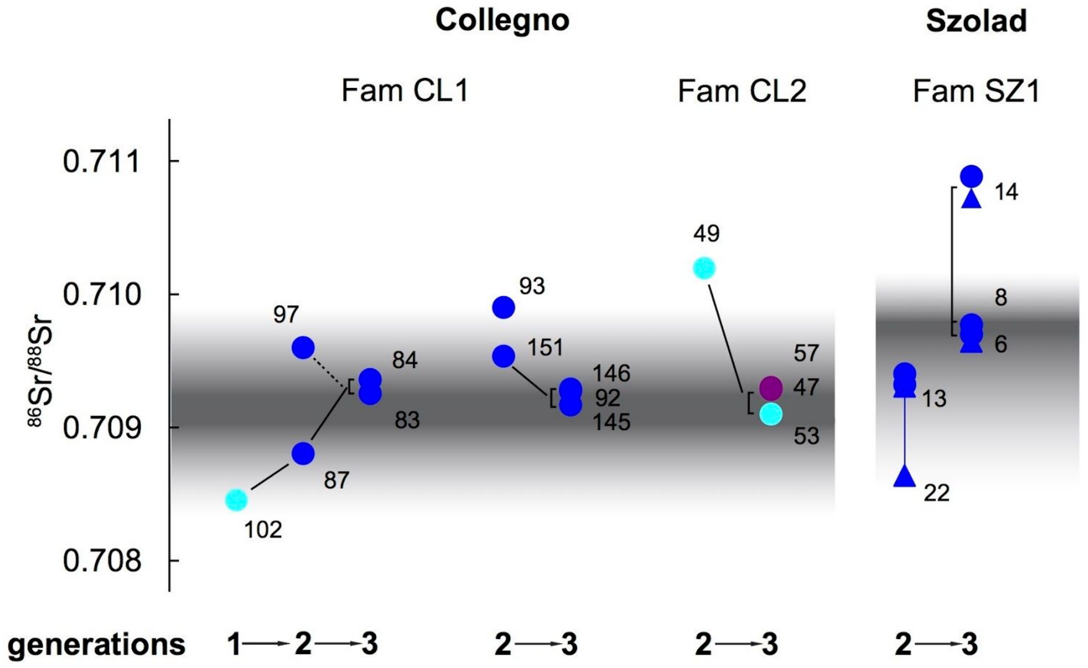

Supplementary Figure 100. First generation migrants at Collegno and Szólád, as suggested by $^{87}Sr/^{86}Sr$ values of individuals from known kinship groups. The grey band denotes the local bioavailable strontium range. Generational relationships are indicated with lines.

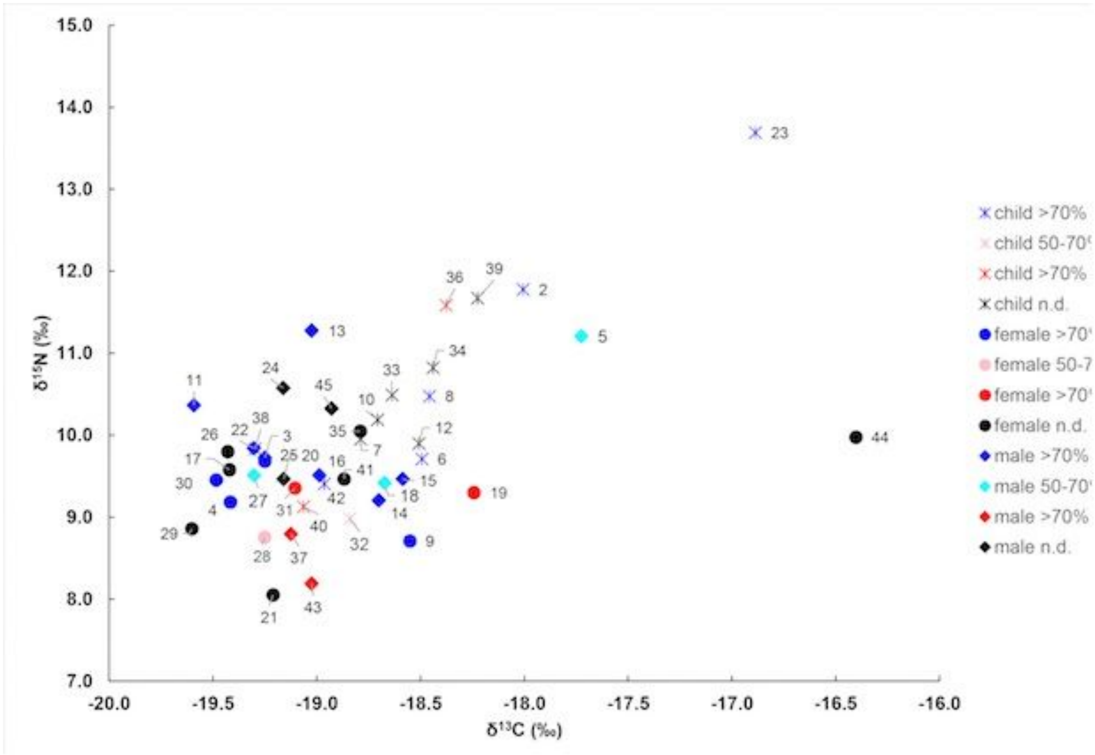

Supplementary Figure 101. Dietary evidence at Szólád, as indicated by $\delta^{13}C_{coll}$ and $\delta^{15}N$ values of bone collagen. Ancestry groups and migration status are highlighted.

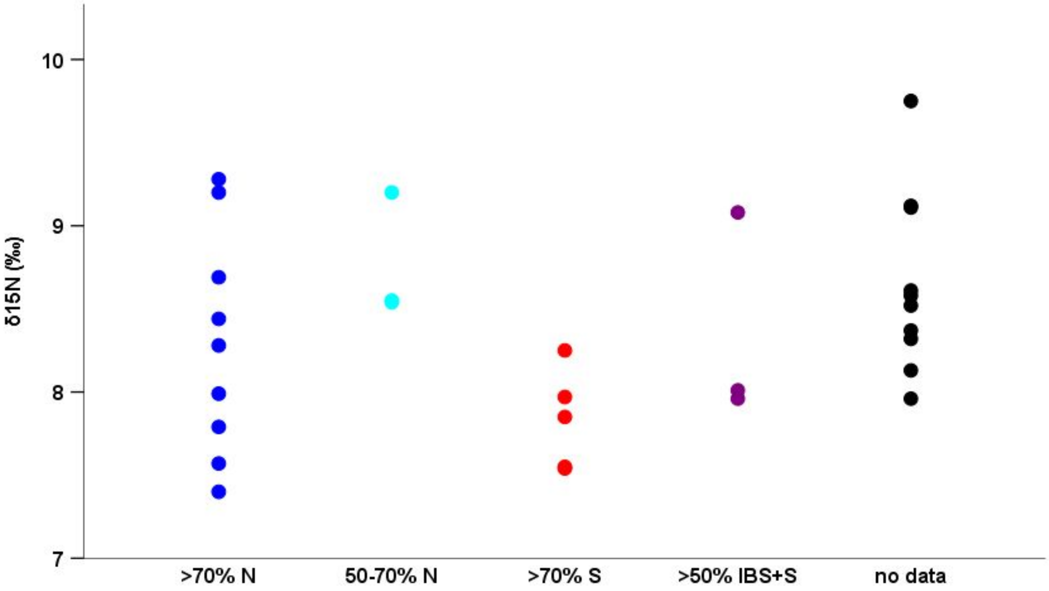

Supplementary Figure 102. Variation in $\delta^{15}N$ values of adults of different ancestry groups at Collegno. Key: blue: >70% N; cyan: 50-70% N; red: >70% S; purple: >50% IBS+S; black: no ancestry data.

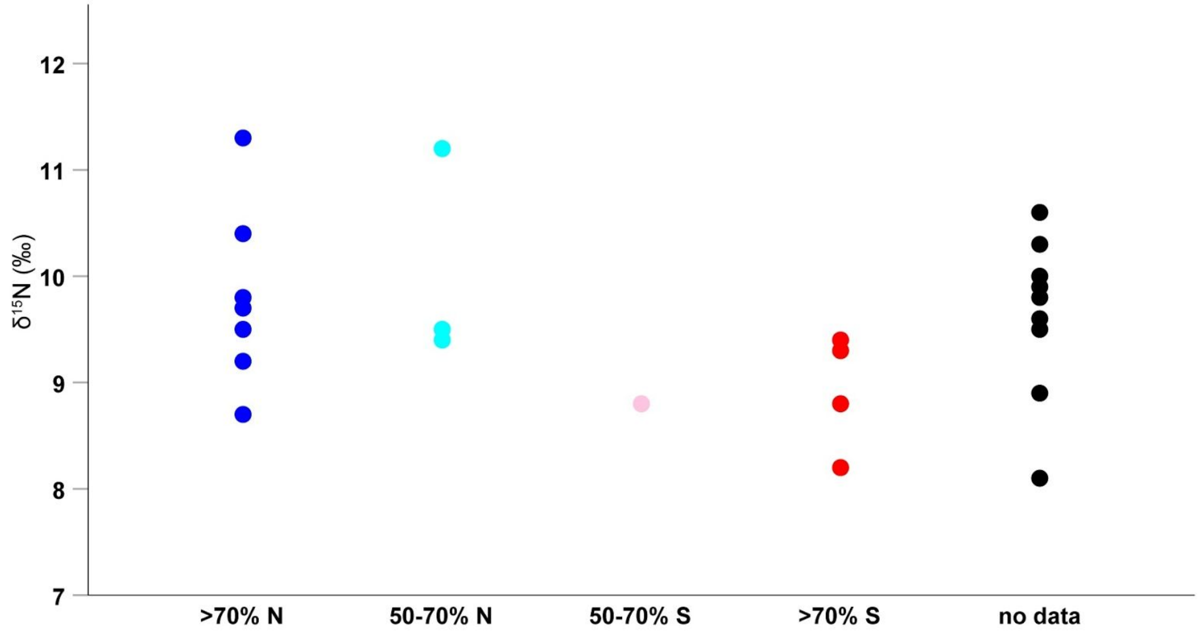

Supplementary Figure 103. Variation in $\delta^{15}N$ values of adults of different ancestry groups at Szólád. Key: blue: >70% N; cyan: 50-70% N; pink: 50-70% S; red: >70% S; black: no ancestry data.

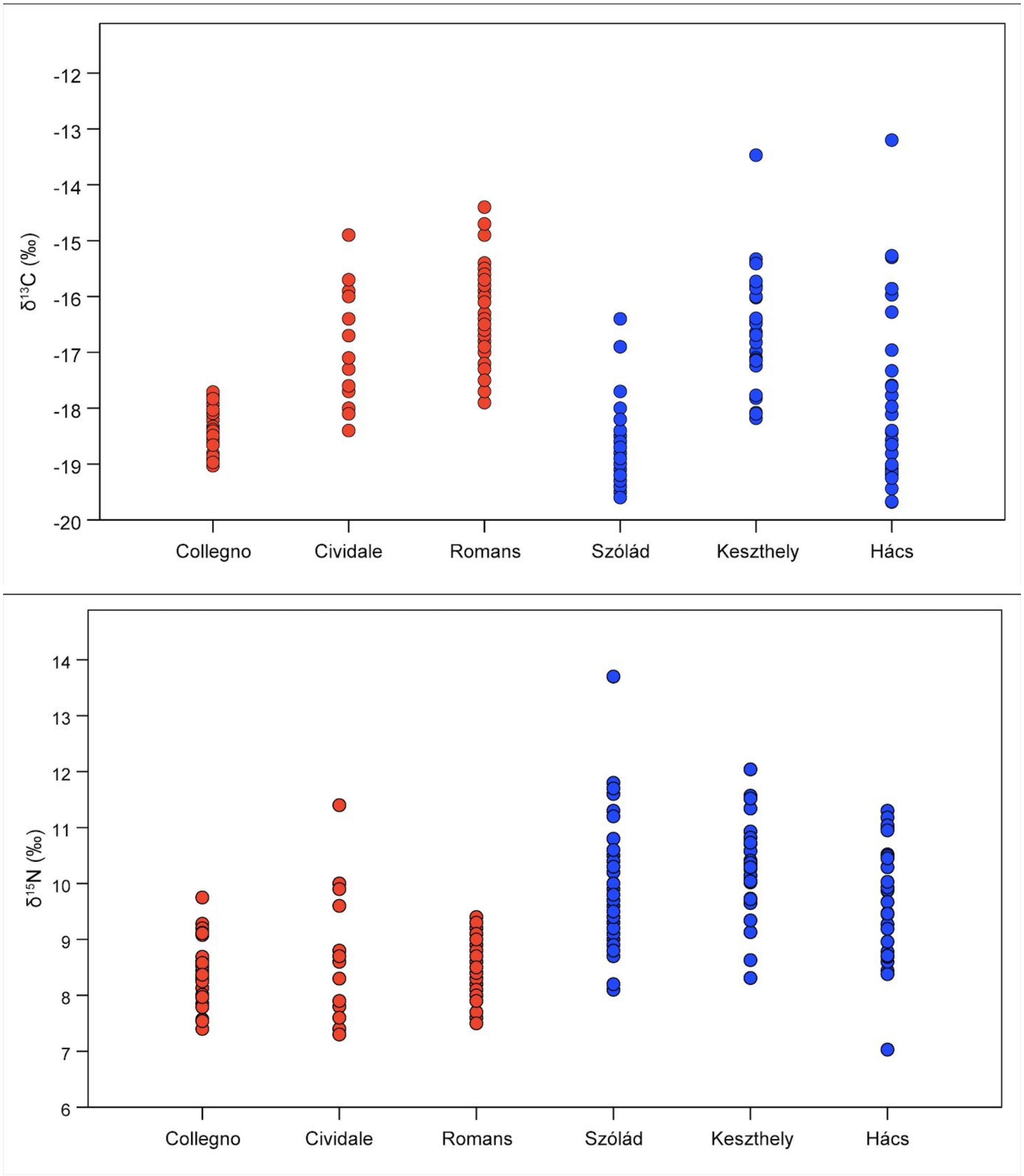

Supplementary Figure 104. Range and distribution of $\delta^{13}C$ and $\delta^{15}N$ values from bone collagen at Collegno (red), Szólád (blue) and comparative sites in northern Italy and Pannonia (Data from Hakenbeck et al.[150] and Iacumin et al. [147])

Supplementary Table 1. 14C results for 8 samples from Szólád.

Sample Name	14C age (yr BP) +-	Cal 1-sigma (CE)	Cal 2-sigma (CE)	C:N	C (%)	Collagen (%)
**SZ13**	1579±25	433-533	422-541	3.2	16.3	1.9
**SZ19**	1567±24	435-537	426-549	3.6	33.0	3.8
**SZ21**	1524±24	470-591	434-602	4.1	45.2	4.0
**SZ27B**	1595±27	423-532	412-538	4.3	35.4	5.2
**SZ37**	1544±26	437-560	430-577	3.2	19.4	2.1
**SZ43**	1521±25	475-595	435-604	4.2	52.7	1.6
**AV1**	1487±26	556-605	541-637	3.2	18.9	2.8
**AV2**	1472±27	566-619	548-641	4.4	57.6	1.8

Supplementary Table 2. Y-chromosome haplogroup attribution for 37 medieval and 1 Bronze age individuals.

Site	Label	Haplogroup	Last downtream SNP	Major distribution
Collegno	CL38	E1b1b1a1b1a3	PF2211	Balkans, Southern Italy
	CL31	G2a1a1	Z6644	Caucasus, Balkans, Central Europe, Italy, Sardinia
	CL63	I1a3	Z79	Central Eastern Europe
	CL23	T1a2b	L446	Southern Europe
	CL110	R1b1a2a	M694	Europe
	CL53	R1b1a2a	PF6434	Europe
	CL57	R1b1a2a1a	L151	Western Europe
	CL93	R1b1a2a1a	L151	Western Europe
	CL145	R1b1a2a1a	L151	Western Europe
	CL146	R1b1a2a1a	L151	Western Europe
	CL92	R1b1a2a1a	L52	Western Europe
	CL84	R1b1a2a1a2c1g1a1	Z381	Central Europe
	CL30	R1b1a1a2a1a2	S116	Central Europe, Italy
	CL49	R1b1a1a2a1a2b1a	Z367	Central Europe, Italy
	CL94	R1b1a1a2a1a2f	S11987	Central Europe, Italy
	CL121	R1b1a2a2	Z2103	Eastern Europe, Caucasus
Szólád	SZ18	E1b1b1a1b2	CTS2817	Italy, Iberia, North Africa
	SZ45	I1a1b1	L22	Central Europe, Scandinavia
	SZ12	I2a2a1	CTS9183	Balkans, Central Europe
	SZ14	I2a2a1	CTS9183	Balkans, Central Europe
	SZ24	I2a2a1	CTS9183	Balkans, Central Europe
	SZ43	I2a2a1a2a1a	S391	Central Europe
	SZ3	I2a2a1b2a2	S390	Germanic region
	SZ13	I2a2a1b2a2a2	ZS20	Germanic region
	SZ22	I2a2a1b2a2a2	ZS20	Germanic region
	SZ7	I2a2a1b2a2a2	ZS20	Germanic region
	SZ36	T1a1a	PF5620	Italy, Iberia
	SZ15	R1a1a1b1a3a	S200	Northern and Eastern Europe
	SZ4	R1b1a2a1a1b	Z16	Central and Western Europe
	SZ16	R1b1a2a1a1c	Z381	Central Europe
	SZ23	R1b1a2a1a1c	Z381	Central Europe
	SZ2	R1b1a1a2a1a1c2b2a1b1a	L130	Central and Western Europe
	SZ11	R1b1a1a2a1a1c2b2b1a1a1	Z351	Central Europe, Italy
	SZ27B	R1b1a1a2a1a2	S116	Central Europe, Italy
	SZ37	R1b1a1a2a1a2	S116	Central Europe, Italy
	SZ42	R1b1a1a2a1a2	S116	Central Europe, Italy
	SZ5	R1b1a1a2a1a2a1b	CTS1595	Central Europe, Italy
Szólád (bronze age)	SZ1	R1a1a1b2a2a	Z2123	Eastern Europe, Caucasus

Supplementary Table 3. Ancient pairwise comparisons that form a significant clade in comparison to all tested reference populations.

Dataset	Pop 1	Pop 2
HellBus region + country	SZ13	CL87
HellBus region + country	SZ13	SZ30
HellBus region + country	SZ16	CL87
HellBus region + country	SZ30	CL151
HellBus region + country	SZ38	CL151
HellBus region + country	CL151	CL87
HellBus region + country	SZ18	SZ38
HellBus region + country	SZ19	SZ32
HellBus region	SZ13	SZ16
HellBus region	SZ13	O2
HellBus region	SZ18	SZ28
HellBus region	CL63	CL151
HellBus region	CL47	CL94

Supplementary Table 4. Comparison of Schiffels et al. and our estimates of demographic parameters for modern European populations using rarecoal.

Parameter	Schiffels ML estimate	Our ML estimate	Lower 95% CI	Upper 95% CI
$t_{(NED,DAN)}$	4.13E-03	3.72E-03	3.70E-03	3.73E-03
$t_{FIN(NED,DAN))}$	4.38E-03	4.00E-03	3.97E-03	4.01E-03
$t_{F(FIN,(NED,DAN)),GBR)}$	4.49E-03	4.05E-03	4.05E-03	4.08E-03
$t_{(IBS,TSI)}$	1.74E-03	5.54E-03	5.52E-03	5.55E-03
$t_{(((FIN,(NED,DAN)),GBR,(IBS,TSI))}$	6.01E-03	5.55E-03	5.53E-03	5.56E-03
N_{FIN}	0.60	0.53	0.53	0.53
N_{GBR}	4.87	4.21	4.15	4.25
N_{IBS}	3.93	9.23	9.15	9.32
N_{TSI}	3.26	7.44	7.39	7.52
N_{NED}	9.96	9.50	9.20	9.68
N_{DAN}	1.95	2.42	2.36	2.50
$N_{(NED,DAN)}$	0.57	0.68	0.66	0.72
$N_{FIN(NED,DAN))}$	0.71	0.65	0.62	0.71
$N_{(FIN,(NED,DAN)),GBR)}$	0.64	0.65	0.65	0.65
$N_{(IBS,TSI)}$	997.00	5.62	1.17	6.33
$N_{(((FIN,(NED,DAN)),GBR,(IBS,TSI))}$	1.02	0.91	0.91	0.91

Supplementary Table 5. Individuals considered for the different ancestry groups.

**Ancestry group**	**Individuals (*T* = 70%)**	**Excluded if *T* = 90%**	**Included if *T* = 60%**
N	SZ7, SZ22, SZ13, SZ12, SZ4, SZ9, SZ41, SZ30, SZ11, SZ16, SZ2, SZ24, SZ15, SZ42, SZ8, SZ38, SZ14, SZ6, SZ3, SZ25, SZ23, CL93, CL145, CL146, CL84, CL92, CL151, CL83, CL97, CL87, and CL63	SZ38, SZ14, SZ6, SZ3, SZ25, SZ23, and CL63	SZ5, and CL102
S	SZ37, SZ28, SZ36, SZ31, SZ43, SZ40, SZ32, SZ19, CL23, CL110, CL121, CL30, CL38, and CL25	SZ37 and SZ28	-

Supplementary Table 6. Contingency tables describing the distribution of artifacts in graves of individuals of different genetic ancestries.

	T **(%)**	***N*** **with grave goods**	***N*** **no grave goods**	***S*** **with grave goods**	***S*** **no grave goods**	**p-value**
**Szólád**	**60**	22	0	2	6	0.0000
	**70**	21	0	2	6	0.0001
	**90**	15	0	2	5	0.0008
**Collegno**	**60**	9	2	0	6	0.0070
	**70**	8	2	0	6	0.0070
	**90**	7	2	0	6	0.0070

Northern (N) and Southern (S) European genetic ancestry.

Supplementary Table 7. Contingency tables for Fisher exact test for the association between the presence and absence of grave goods and genetic ancestry.

Artifact	*N* with	*N* without	*S* with	*S* without	Population	P-value
Amulet (F)	2	4	0	5	Szólád	0.4545
S-Brooch (F)	4	2	1	4	Szólád	0.2424
Beads (F)	6	0	1	4	Szólád	0.0152
Ring (F)	1	5	1	4	Szólád	1.0000
Spindle whorl (F)	2	4	0	5	Szólád	0.4545
Strike-a-light (M)	3	12	0	3	Szólád	1.0000
Weaponry (M)	10	0	0	2	Szólád	0.0152
Pottery (B)	7	14	1	7	Szólád	0.3805
Food offering (B)	17	4	0	8	Szólád	0.0001
Weaponry (M)	5	1	0	5	Collegno	0.0152
Belt for weapons (M)	3	3	0	5	Collegno	0.1818
Strike-a-light (M)	2	5	0	5	Collegno	0.4697

Individuals are classified according to their genetic ancestry as *N* (northern European) or *S* (southern European or Iberian). Each contingency table was stretched in one line. The first column describes the artifact that is being tested. In parenthesis a character describes if an artifact is restricted to females (F), males (M) or none (B). The following four columns describe how many individuals were seen with and without the corresponding grave good, given the genetic ancestry (*N* or *S* accordingly). The column "Cemetery" describes the site where specimens were sampled and the last column the p-value for a Fisher Exact test. Significant p-values are shared in dark grey.

Supplementary Table 8. Contingency tables for the different grave types containing individuals of different genetic ancestries.

Site	*N* simple pit	*N* wooden elements	*S* simple pit	*S* wooden elements	p-value
**Szólád**	1	20	7	1	<0.0001
**Collegno**	4	6	6	0	0.0338

Northern (N) and Southern (S) European genetic ancestry.

Supplementary Table 9. Isotope values from human samples in Collegno.

Sample ID	Tooth[1]	Bone	Tooth enamel				Bone collagen		C:N
			$^{87}Sr/^{86}Sr$	Sr ppm	$\delta^{18}O_{carb}$ (‰ vs. V-PBD)	$\delta^{13}C_{carb}$ (‰ vs. V-PBD)	$\delta^{13}C_{coll}$ (‰ vs. V-PBD)	$\delta^{15}N$ (‰ vs. AIR)	
CL004	M2 R	humerus	0.709320	9.34	-6.1	-8.5	-18.4	9.1	3.22
CL009	M2 L	rib	0.709972	4.82	-5.9	-5.9	-18.6	8.3	3.18
CL010	M2 L	rib	0.709234	11.49	-5.0	-12.6	-18.7	8.4	3.17
CL023	P2 L	humerus	0.708121	9.44	-6.2	-12.6	-18.6	8.0	3.11
CL025	P2 UL	rib	0.709568	177.21	-4.0	-13.5	-18.8	7.9	3.18
CL028	M2 L	metatarsal	0.709449	8.62	-5.7	-9.7	-17.9	8.6	3.11
CL030	P1 R	rib	0.709043	4.87	-4.8	-12.1	-18.4	8.0	3.12
CL031	P2 R	phalanx	0.709450	4.85	-4.8	-12.1	-17.8	8.2	3.09
CL036	P2 UR	rib	0.709478	130.74	-6.6	-11.2	-18.6	8.2	3.17
CL038	P2 UR	rib	0.709303	200.49	-6.5	-11.6	-18.6	7.5	3.15
CL040	P2 L	metacarpal	0.709453	7.49	-6.6	-9.6	-18.9	8.5	3.10
CL041	P2 L	rib	0.709141	5.89	-5.1	-11.0	-19.0	8.6	3.13
CL047	P2 R	ulna	0.709288	20.36	-4.6	-13.6	-19.0	8.0	3.11
CL048	P	tibia	0.710280	4.53	-6.2	-12.8	-18.5	9.8	3.11
CL049	P L	metatarsal	0.710198	5.44	-6.0	-12.1	-19.0	9.2	3.13
CL053	P2 UR	rib	0.709102	217.99	-6.3	-12.4	-18.9	8.6	3.19
CL057	P2 UL		0.709302	183.88	-6.4	-10.6			
CL063	P2 LR	rib	0.709661	185.53	-6.5	-12.3	-18.5	9.2	3.21
CL083	M2 UL	rib	0.709256	182.20	-5.6	-11.8	-17.8	9.3	3.16
CL084	M2 LL	rib	0.709362	150.63	-6.7	-10.3	-18.1	7.4	3.18
CL086	M2 L	ulna	0.709642	5.31	-7.0	-11.8	-18.5	9.1	3.13
CL087	P2 L	rib	0.708805	5.91	-6.9	-12.1	-18.2	7.6	3.10
CL090	P2 R	rib	0.709246	12.26	-6.4	-11.4	-18.1	8.0	3.12
CL092	P2 R	rib	0.709274	9.59	-6.8	-12.6	-18.6	8.0	3.11
CL093	M2 R	rib	0.709903	6.02	-6.7	-11.0	-18.4	7.8	3.13
CL094	P2 L	rib	0.708124	25.53	-8.8	-11.2	-17.7	9.1	3.11
CL097	M2 R	rib	0.709600	6.80	-6.2	-11.9	-18.4	8.3	3.15
CL102	P2 L	tibia	0.708457	5.48	-6.1	-12.7	-18.9	8.5	3.10
CL121	M2 UL	rib	0.709428	245.25	-7.1	-10.4	-18.1	7.6	3.17
CL145	P2 R	rib	0.709170	13.14	-6.7	-12.0	-18.4	8.4	3.13
CL146	P2 R	rib	0.709291	8.93	-7.0	-11.7	-18.0	8.7	3.12
CL147	M2 L	clavicle	0.711097	7.21	-4.0	-11.5	-18.3	8.1	3.12
CL151	P2 UR		0.709537	179.02	-7.2	-11.6			

[1]Teeth are recorded as: P1 - first premolar; P2 - second premolar; M2 - second molar

Supplementary Table 10. Isotope values from fauna for comparison to Collegno.

Sample ID	Species	$\delta^{13}C_{coll}$	$\delta^{15}N$	C/N
TOR F001	*Gallus gallus*	-17.6	7.2	3.16
TOR F002	*Bos taurus*	-22.4	5.8	3.13
TOR F003	*Felis domestica*	-18.5	8.9	3.15
TOR F004	*Sus scrofa*	-20.9	7.1	3.13
TOR F005	*Equus caballus*	-21.7	4.8	3.18
TOR F006	*Canis familiaris*	-17.1	8.8	3.09

Supplementary Table 11. Isotope values from fauna for comparison to Collegno.

Sample ID	Location	$^{87}Sr/^{86}Sr$	ppm	Lat	Long	Elevation (masl)	Type	Environmental context	Geology	Description
TOR E5	Lago Piccolo di Avigliana	0.710492		45.050341	7.394539	355	water	lake shore	Glacial deposits (Pleistocene)	Lago Piccolo di Avigliana
TOR E12	Dora (Turin)	0.708390		45.081358	7.685964	229	water	urban		Dora
TOR E13	Po (Turin)	0.709689		45.069733	7.720191	207	water	urban		Po
TOR E2	Parco Generale dall Chiesa, Collegno	0.708809		45.079862	7.577408	298	soil	civic park	Pleistocene alluvium	Clay silt, no inclusions; dry: orange mid brown
TOR E3	Parco Generale dall Chiesa, Collegno	0.709001	42.78	45.079643	7.577606	298	leaf	civic park	Pleistocene alluvium	Tilia cordata
TOR E4	Parco Generale dall Chiesa, Collegno	0.709123	20.72	45.079837	7.576593	299	leaf	civic park	Pleistocene alluvium	Celtis australis
TOR E6	Lago Piccolo di Avigliana	0.709800	18.32	45.050341	7.394539	355	leaf	lake shore	Glacial deposits (Pleistocene)	Carpinus betulus
TOR E7	Lago Piccolo di Avigliana	0.710176	0.12	45.050086	7.394854	358	soil	meadows	Glacial deposits (Pleistocene)	Silt with some clay: inclusions: subangular stones (< 1 cm) 1%; damp: dark grey brown; sticky
TOR E8	Castello di Avigliana	0.709416	5.71	45.079222	7.390282	402	leaf	top of rocky outcrop	Serpentinite, Green schist	Quercus
TOR E9	Castello di Avigliana	0.709227	0.23	45.079394	7.389884	405	soil	pasture	Serpentinite, Green schist	Sandy Silt; dry: medium orange brown; loose; inclusions: subangular shist frags (<1 cm) 5%
TOR E14	Collino di Superga	0.710312	0.02	45.061383	7.775918	478	soil	deciduous forest, no ground cover	Clays and marls (Miocene)	Clay with silt, no inclusions; sticky; light yellow-grey brown
TOR E15	Collino di Superga	0.710398	10.37	45.061383	7.775918	478	leaf	deciduous forest, no ground cover	Clays and marls (Miocene)	Carpinus betulus

Supplementary Table 12. Summary statistics of $\delta^{13}C_{collagen}$ (‰) values from bone collagen from Collegno, Szólád and comparative sites in Pannonia and Bavaria.

$\delta^{13}C_{collagen}$ (‰)	Mean	1 standard deviation	Min	Max	Range
Collegno	-18.4	0.4	-19.0	-17.7	1.3
Szólád	-18.8	0.6	-19.6	-16.4	3.2
Cividale (Gallo and S. Stefano)	-17.0	1.2	-18.4	-14.9	3.5
Romans	-16.4	0.8	-17.4	-14.4	3.5
Hács-Béndekpuzsta	-17.8	1.3	-19.2	-15.3	3.9
Keszthely-Fenékpuszta	-17.0	0.8	-18.2	-15.4	2.8

Supplementary Table 13. Summary statistics of $\delta^{15}N$ (‰) values from bone collagen from Collegno, Szólád and comparative sites in Pannonia and Bavaria.

$\delta^{15}N$ (‰)	Mean	1 standard deviation	Min	Max	Range
Collegno	8.4	0.6	7.4	9.8	2.4
Szólád	9.9	1.0	8.1	13.7	5.6
Cividale (Gallo and S. Stefano)	8.6	1.2	7.3	11.4	4.1
Romans	8.4	0.5	7.5	9.4	1.9
Hács-Béndekpuzsta	9.0	1.0	7.0	10.3	3.3
Keszthely-Fenékpuszta	10.0	0.7	8.3	11.0	2.7

Supplementary Table 14. Summary of different population genetic analyses performed, relevant datasets used and how medieval samples were called

Analysis	Relevant Figure/Table	Medieval genetic data type	Reference Dataset Used	Relevant Supplementary Notes
PCA - medieval vs modern/ancient reference dataset	Figures 2A-B, Supplementary Figures 23-25, 65-68,73-82, 87	Pseudo-haploid (best likelihood)	POPRES, HellBus, 1000G, POPRES + All ancient, HellBus + ancient	Supplementary Note 7
PCA - only ancient samples	Supplementary Figures 26-28	Pseudo-haploid (random read)	na	Supplementary Note 7
PCA - high coverage medieval vs reference dataset	Supplementary Figure 62	Diploid genotype	POPRES	-
Model-based clustering analysis	Figures 3A-B, Supplementary Figures 29-43,45-46, 69, 72	Pseudo-haploid (best likelihood)	1000 Genomes Project, SGDP, ancient	Supplementary Note 8
Y-chromosome haplogroup inference	Supplementary Figures 47-49, Supplementary Table 2	Haploid (best likelihood)	1000 Genomes Project, Sardinians	Supplementary Note 9
Population assignment analysis	Supplementary Data 4	Pseudo-haploid (best likelihood)	POPRES, HellBus	Supplementary Note 10
D-statistics - Medieval vs. modern samples	Supplementary Figures 51-61, Supplementary Table 3	Pseudo-haploid (random read)	SGDP, HellBus	Supplementary Note 11
D-statistics - Medieval vs. Bronze Age samples	Supplementary Figures 83-84	Pseudo-haploid (best likelihood)	Ancient Bronze Age	Supplementary Note 11
Rare variant analysis	Supplementary Figures 63-64, Supplementary Table 4	Pseudo-haploid (best likelihood) and diploid genotype	1000 Genomes Project, Nederthands and Danish WGS variants	Supplementary Note 12
Kinship inference	Figures 4A-C	Genotype likelihoods	1000 Genomes Project	Supplementary Note 13
SPA analysis	Supplementary Figures 88-96	Pseudo-haploid (best likelihood)	POPRES	Supplementary Note 14

SUPPLEMENTARY REFERENCES

1. Jarnut, J. *Geschichte Der Langobarden*. (Kohlhammer, 1982).
2. Pohl, W. *Kingdoms of the Empire: The Integration of Barbarians in Late Antiquity*. (BRILL, 1997).
3. Bergamo, N. *I Longobardi: dalle origini mitiche alla caduta del regno in Italia*. (Libreria Editrice Goriziana, 2012).
4. Ausenda, G., Delogu, P. & Wickham, P. *The Langobards before the Frankish Conquest: An Ethnographic Perspective*. (Boydell Press, 2009).
5. Gasparri, S. *Italia longobarda: Il regno, i Franchi, il papato*. (Gius.Laterza & Figli Spa, 2016).
6. Hen, Y. & Innes, M. *The Uses of the Past in the Early Middle Ages*. (Cambridge University Press, 2000).
7. Diaconus, P. Historia Langobardorum,. in *Monumenta Germaniae Historica, Scriptores Rerum Langobardorum* (ed. Waitz, G.) 12–187 (1878).
8. Goffart, W. A. *The Narrators of Barbarian History (A.D. 550-800): Jordanes, Gregory of Tours, Bede, and Paul the Deacon*. (Publications in Medieval Studies, 1988).
9. Skinner, P. Gender, memory and Jewish identity: reading a family history from medieval southern Italy. *Early Medieval Europe* **13,** 277–296 (2005).
10. Pohl, W. *Werkstätte der Erinnerung: Montecassino und die Gestaltung der langobardischen Vergangenheit*. (Oldenbourg Verlag, 2001).
11. Ausenda, G. & Barnish, S. A Comparative Discussion Of Langobardic Feud And Blood-Money Compensation With Parallels From Contemporary Anthropology And From Medieval History. in *The Langobards before the Frankish Conquest: An Ethnographic Perspective* (eds. Ausenda, G., Delogu, P. & Wickham, C.) 309–339 (Boydell Press, 2009).
12. Murray, A. C. *Germanic Kinship Structure: Studies in Law and Society in Antiquity and in the Early Middle Ages*. (PIMS, 1983).
13. Wickham, C. *Early Medieval Italy: Central Power and Local Society, 400-1000*. (1981).
14. Christie, N. Pannonia: Foundations of Langobardic Power and Identity. in *The Langobards before the Frankish Conquest: An Ethnographic Perspective* (eds. Ausenda, G., Delogu, P. & Wickham, C.) 6–29 (Boydell Press,

2009).

15. Costambeys, M. Kinship, Gender and Property in Lombard Italy. in *The Langobards before the Frankish Conquest: An Ethnographic Perspective* (eds. Ausenda, G., Delogu, P. & Wickham, C.) 69–94 (Boydell Press, 2009).

16. Green, D. Linguistic and Literary Traces of the Langobards. in *The Langobards before the Frankish Conquest: An Ethnographic Perspective* (eds. Ausenda, G., Delogu, P. & Wickham, C.) 174–194 (Boydell Press, 2009).

17. von Freeden, U., Vida, T. & Skriba, P. Ausgrabung des langobardenzeitlichen Gräberfeldes von Szólád, Komitat Somogy, Ungarn: Vorbericht und Überblick über langobardenzeitliche Besiedlung am Plattensee. *Germania: Anzeiger der Römisch-Germanischen Komission des Deutschen Archäologischen Institute* **85,** 359–384 (2007).

18. Honti, S. *et al.* A tervezett M7-es autópálya Somogy megyei szakaszának megelőző régészeti feltárása (2002-2003) Előzetes jelentés III.--Preliminary Report III. The preceding archaeological excavations (2002-2003) of the M7 highway in Somogy County. *Somogyi Múzeumok Közleményei* **16,** (2004).

19. von Freeden, U. Ausgewählte Befunde aus dem langobardenzeitlichen Gräberfeld von Szólád, Komitat Somogy, Ungarn. in *Kulturwandel in Mitteleuropa Langobarden – Awaren – Slawen* (eds. Bemmann, J. & Schmauder, J.) 399–413 (2008).

20. Sudhoff, I. *Kreisgräben, Grabhügel und verwandte Sonderformen von Grabanlagen im Merowingerreich*. (Rheinische Friedrich-Wilhelms-Universität, Bonn, 1999).

21. Passard, F. & Urlacher, J. P. Architectures funéraires des la nécropole des Saint-Vi. in *Burgondes, Alamans, Francs, Romains dans l'Est de la France, le Sud-Ouest de l'Allemagne et la Suisse, 5e-7e siècle après J.-C* (2003).

22. Neugebauer, J. W. Rettungsgrabungen im Unteren Traisental in den Jahren 2000 und 2001. *Fundberichte aus Österreich* **40,** 191–300 (2001).

23. Webster, C. J. & Brunning, R. A. A Seventh-Century ad Cemetery at Stoneage Barton Farm, Bishop's Lydeard, Somerset and Square-Ditched Burials in Post-Roman Britain. *Archaeological Journal* **161,** 54–81 (2004).

24. Winger, D. & Bartelt, D. Auf der Suche nach dem Haus des „Fürsten" – Siedlung und Prunkgrab der Merowingerzeit von Hemmingen-Hiddestorf, Region Hannover. in *Niedersächsisches Institut für historische*

Küstenforschung (Hrsg.), Aktuelle Forschungen an Gräberfeldern des 1. Jahrtausends n.Chr. Siedlungs- und Küstenforschung im südlichen Nordseegebiet 39 (Rahden/Westfalen 2016) 122-127. (ed. Niedersächsisches Institut Für) 122–127 (2016).

25. Mengarelli, R. La necropoli barbarica di Castel Trosino. in *Monumenti Antichi* (ed. Accademia dei Lincei, R.) **12,** (1902).
26. Horváth, E. A langobard ékkő-és üvegberakás technológiai sajátosságainak vizsgálata a várpalotai és jutasi fibulákon. *Veszprém Megyei Múzeumok Közleményei* **24,** 49–66 (2006).
27. Pánczél-Bajnok, K., Pánczél, P., Szakmány, G. & Vida, T. 5-6. századi, Pannónia területéről származó kerámiák archaeometriai elemzése. *Archeometriai Műhely* **XI,** 1–12 (2014).
28. Alt, K. W. *et al.* Lombards on the Move – An Integrative Study of the Migration Period Cemetery at Szólád, Hungary. *PLoS One* **9,** e110793 (2014).
29. Geary, P. J. Rethinking Barbarian Invasions through Genomic History. *Hungarian Archaeology* 1–8 (2014).
30. Stuiver, M. & Polach, H. A. Discussion Reporting of 14C Data. *Radiocarbon* **19,** 355–363 (1977).
31. Reimer, P. J. *et al.* IntCal13 and Marine13 Radiocarbon Age Calibration Curves 0–50,000 Years cal BP. *Radiocarbon* **55,** 1869–1887 (2013).
32. Peirani Baricco, L. L'insediamento e le necropoli dal VI all'VIII secolo. in *Presenze longobarde. Collegno nell'alto medioevo* (ed. Pejrani Baricco, L.) 17–52 (2004).
33. Pejrani Baricco, L. Longobardi da guerrieri a contadini. Le ultime ricerche in Piemonte. in *Archeologia e società nell'Italia altomedievale (V-IX secolo)* (eds. Brogiolo, G. P. & Chavarria Arnau, A.) 363–383 (2007).
34. Pejrani Baricco, L. Il Piemonte tra Ostrogoti e Longobardi. in *I Longobardi. Dalla caduta dell'impero all'alba dell'Italia* (eds. Brogiolo, G. P. & Chavarria Arnau, A.) 255–267 (2007).
35. Ferembach, D., Schwidetzky, I. & Stloukal, M. Recommandations pour déterminer l'âge et le sexe sur le squelette. *Bull. Mem. Soc. Anthropol. Paris* **6,** 7–45 (1979).
36. Bedini, E. & Bertoldi, F. Aspetto fisico, stile di vita e stato di salute del gruppo umano. in *Presenze longobarde. Collegno nell'alto medioevo* (ed. Pejrani Baricco, L.) 217–235 (2004).
37. Giostra, C. Gli oggetti di corredo; Catalogo. in *Presenze longobarde. Collegno nell'alto medioevo* (ed. Pejrani Baricco, L.) 53–152 (2004).

38. Giostra, C. La necropoli di Collegno (scheda 4.27). Tomba femminile 47. Tomba femminile 48. Tomba maschile 49. Tomba maschile 53. Tomba maschile 17. in *I Longobardi. Dalla caduta dell'Impero all'alba dell'Italia, catalogo della mostra* (eds. Brogiolo, G. P. & Chavarria Arnau, A.) 268–273 (2007).

39. Giostra, C. Goths and Lombards in Italy: the potential of archaeology with respect to ethnocultural identification. *Post-Classical Archaeologies* **1,** 7–36 (2011).

40. Martin, R. & Saller, K. *Lehrbuch der Anthropologie in systematischer Darstellung*. (Fischer, 1956-1959).

41. Bartoli, F. & Bedini, E. Le abitudini alimentari. in *Presenze longobarde. Collegno nell'alto medioevo* (ed. Pejrani Baricco, L.) 241–247 (2004).

42. Castiglioni, E., Cottini, M., Rettore, E. & Rottoli, M. Il legno, i tessuti, i cuoi e gli altri materiali organici dalla necropoli longobarda e dall'abitato. in *Presenze longobarde. Collegno nell'alto medioevo* (ed. Pejrani Baricco, L.) 177–206 (2004).

43. Pinhasi, R. *et al.* Optimal Ancient DNA Yields from the Inner Ear Part of the Human Petrous Bone. *PLoS One* **10,** e0129102 (2015).

44. Dabney, J. *et al.* Complete mitochondrial genome sequence of a Middle Pleistocene cave bear reconstructed from ultrashort DNA fragments. *Proc. Natl. Acad. Sci. U. S. A.* **110,** 15758–15763 (2013).

45. Meyer, M. & Kircher, M. Illumina Sequencing Library Preparation for Highly Multiplexed Target Capture and Sequencing. *Cold Spring Harb. Protoc.* **2010,** db.prot5448–pdb.prot5448 (2010).

46. Kircher, M., Sawyer, S. & Meyer, M. Double indexing overcomes inaccuracies in multiplex sequencing on the Illumina platform. *Nucleic Acids Res.* **40,** e3 (2012).

47. Peltzer, A. *et al.* EAGER: efficient ancient genome reconstruction. *Genome Biol.* **17,** 60 (2016).

48. Li, H. & Durbin, R. Fast and accurate short read alignment with Burrows-Wheeler transform. *Bioinformatics* **25,** 1754–1760 (2009).

49. Kircher, M. Analysis of high-throughput ancient DNA sequencing data. *Methods Mol. Biol.* **840,** 197–228 (2012).

50. Lassmann, T., Hayashizaki, Y. & Daub, C. O. TagDust--a program to eliminate artifacts from next generation sequencing data. *Bioinformatics* **25,** 2839–2840 (2009).

51. Jónsson, H., Ginolhac, A., Schubert, M., Johnson, P. L. F. & Orlando, L. mapDamage2.0: fast approximate

Bayesian estimates of ancient DNA damage parameters. *Bioinformatics* **29,** 1682–1684 (2013).

52. Rohland, N., Harney, E., Mallick, S., Nordenfelt, S. & Reich, D. Partial uracil-DNA-glycosylase treatment for screening of ancient DNA. *Philos. Trans. R. Soc. Lond. B Biol. Sci.* **370,** 20130624–20130624 (2014).
53. Mittnik, A., Wang, C.-C., Svoboda, J. & Krause, J. A Molecular Approach to the Sexing of the Triple Burial at the Upper Paleolithic Site of Dolní Věstonice. *PLoS One* **11,** e0163019 (2016).
54. Fu, Q. *et al.* DNA analysis of an early modern human from Tianyuan Cave, China. *Proc. Natl. Acad. Sci. U. S. A.* **110,** 2223–2227 (2013).
55. Haak, W. *et al.* Massive migration from the steppe was a source for Indo-European languages in Europe. *Nature* **522,** 207–211 (2015).
56. DePristo, M. A. *et al.* A framework for variation discovery and genotyping using next-generation DNA sequencing data. *Nat. Genet.* **43,** 491–498 (2011).
57. Fu, Q. *et al.* The genetic history of Ice Age Europe. *Nature* **534,** 200–205 (2016).
58. Korneliussen, T. S., Albrechtsen, A. & Nielsen, R. ANGSD: Analysis of Next Generation Sequencing Data. *BMC Bioinformatics* **15,** 356 (2014).
59. Renaud, G., Slon, V., Duggan, A. T. & Kelso, J. Schmutzi: estimation of contamination and endogenous mitochondrial consensus calling for ancient DNA. *Genome Biol.* **16,** 224 (2015).
60. Vianello, D. *et al.* HAPLOFIND: a new method for high-throughput mtDNA haplogroup assignment. *Hum. Mutat.* **34,** 1189–1194 (2013).
61. Schiffels, S. *et al.* Iron Age and Anglo-Saxon genomes from East England reveal British migration history. *Nat. Commun.* **7,** 10408 (2016).
62. Veeramah, K. R. *et al.* Population genomic analysis of elongated skulls reveals extensive female-biased immigration in Early Medieval Bavaria. *Proc. Natl. Acad. Sci. U. S. A.* (in press).
63. Damgaard, P. de B. *et al.* 137 ancient human genomes from across the Eurasian steppes. *Nature* **557,** 369–374 (2018).
64. Hofmanová, Z. *et al.* Early farmers from across Europe directly descended from Neolithic Aegeans. *Proc. Natl. Acad. Sci. U. S. A.* **113,** 6886–6891 (2016).
65. Botigué, L. R. *et al.* Ancient European dog genomes reveal continuity since the Early Neolithic. *Nat. Commun.*

8, 16082 (2017).

66. Purcell, S. *et al.* PLINK: A Tool Set for Whole-Genome Association and Population-Based Linkage Analyses. *Am. J. Hum. Genet.* **81,** 559–575 (2007).
67. Nelson, M. R. *et al.* The Population Reference Sample, POPRES: a resource for population, disease, and pharmacological genetics research. *Am. J. Hum. Genet.* **83,** 347–358 (2008).
68. Novembre, J. *et al.* Genes mirror geography within Europe. *Nature* **456,** 98–101 (2008).
69. Das, S. *et al.* Next-generation genotype imputation service and methods. *Nat. Genet.* **48,** 1284–1287 (2016).
70. Loh, P.-R. *et al.* Reference-based phasing using the Haplotype Reference Consortium panel. *Nat. Genet.* **48,** 1443–1448 (2016).
71. 1000 Genomes Project Consortium *et al.* A global reference for human genetic variation. *Nature* **526,** 68–74 (2015).
72. Hellenthal, G. *et al.* A genetic atlas of human admixture history. *Science* **343,** 747–751 (2014).
73. Mallick, S. *et al.* The Simons Genome Diversity Project: 300 genomes from 142 diverse populations. *Nature* **538,** 201–206 (2016).
74. Danecek, P. *et al.* The variant call format and VCFtools. *Bioinformatics* **27,** 2156–2158 (2011).
75. Mathieson, I. *et al.* Genome-wide patterns of selection in 230 ancient Eurasians. *Nature* **528,** 499–503 (2015).
76. Mathieson, I. *et al.* The genomic history of southeastern Europe. *Nature* **555,** 197–203 (2018).
77. Olalde, I. *et al.* The Beaker phenomenon and the genomic transformation of northwest Europe. *Nature* **555,** 190–196 (2018).
78. Patterson, N., Price, A. L. & Reich, D. Population structure and eigenanalysis. *PLoS Genet.* **2,** e190 (2006).
79. Veeramah, K. R. & Hammer, M. F. The impact of whole-genome sequencing on the reconstruction of human population history. *Nat. Rev. Genet.* **15,** 149–162 (2014).
80. Lazaridis, I. *et al.* Ancient human genomes suggest three ancestral populations for present-day Europeans. *Nature* **513,** 409–413 (2014).
81. Allentoft, M. E. *et al.* Population genomics of Bronze Age Eurasia. *Nature* **522,** 167–172 (2015).
82. Petkova, D., Novembre, J. & Stephens, M. Visualizing spatial population structure with estimated effective migration surfaces. *Nat. Genet.* **48,** 94–100 (2016).

83. Alexander, D. H., Novembre, J. & Lange, K. Fast model-based estimation of ancestry in unrelated individuals. *Genome Res.* **19,** 1655–1664 (2009).

84. Francalacci, P. *et al.* Low-pass DNA sequencing of 1200 Sardinians reconstructs European Y-chromosome phylogeny. *Science* **341,** 565–569 (2013).

85. Hallast, P. *et al.* The Y-chromosome tree bursts into leaf: 13,000 high-confidence SNPs covering the majority of known clades. *Mol. Biol. Evol.* **32,** 661–673 (2015).

86. Karmin, M. *et al.* A recent bottleneck of Y chromosome diversity coincides with a global change in culture. *Genome Res.* **25,** 459–466 (2015).

87. Francalacci, P. *et al.* Detection of phylogenetically informative polymorphisms in the entire euchromatic portion of human Y chromosome from a Sardinian sample. *BMC Res. Notes* **8,** 174 (2015).

88. International Society of Genetic Genealogy (ISOGG). Available at: https://isogg.org/tree/index.html.

89. Y Chromosome Consortium. A nomenclature system for the tree of human Y-chromosomal binary haplogroups. *Genome Res.* **12,** 339–348 (2002).

90. Felsenstein, J. *PHYLIP (Phylogeny Inference Package)*. (2005).

91. Rambaut, A. *Fig.Tree. Tree Figure Drawing Tool*. (2012).

92. Francalacci, P. & Sanna, D. History and geography of human Y-chromosome in Europe: a SNP perspective. *J. Anthropol. Sci.* **86,** 59–89 (2008).

93. Semino, O. *et al.* The genetic legacy of Paleolithic Homo sapiens sapiens in extant Europeans: a Y chromosome perspective. *Science* **290,** 1155–1159 (2000).

94. Rootsi, S. *et al.* Phylogeography of Y-chromosome haplogroup I reveals distinct domains of prehistoric gene flow in europe. *Am. J. Hum. Genet.* **75,** 128–137 (2004).

95. Nasidze, I. *et al.* Mitochondrial DNA and Y-chromosome variation in the caucasus. *Ann. Hum. Genet.* **68,** 205–221 (2004).

96. Keller, A. *et al.* New insights into the Tyrolean Iceman's origin and phenotype as inferred by whole-genome sequencing. *Nat. Commun.* **3,** 698 (2012).

97. Mendez, F. L. *et al.* Increased resolution of Y chromosome haplogroup T defines relationships among populations of the Near East, Europe, and Africa. *Hum. Biol.* **83,** 39–53 (2011).

98. Patterson, N. *et al.* Ancient admixture in human history. *Genetics* **192,** 1065–1093 (2012).

99. Genome of the Netherlands Consortium. Whole-genome sequence variation, population structure and demographic history of the Dutch population. *Nat. Genet.* **46,** 818–825 (2014).

100. Besenbacher, S. *et al.* Novel variation and de novo mutation rates in population-wide de novo assembled Danish trios. *Nat. Commun.* **6,** 5969 (2015).

101. Lipatov, M., Sanjeev, K., Patro, R. & Veeramah, K. Maximum Likelihood Estimation of Biological Relatedness from Low Coverage Sequencing Data. (2015). doi:10.1101/023374

102. Weir, B. S., Anderson, A. D. & Hepler, A. B. Genetic relatedness analysis: modern data and new challenges. *Nat. Rev. Genet.* **7,** 771–780 (2006).

103. Anderson, A. D. & Weir, B. S. A maximum-likelihood method for the estimation of pairwise relatedness in structured populations. *Genetics* **176,** 421–440 (2007).

104. Staples, J. *et al.* PRIMUS: rapid reconstruction of pedigrees from genome-wide estimates of identity by descent. *Am. J. Hum. Genet.* **95,** 553–564 (2014).

105. Jacquard, A. *The Genetic Structure of Populations*. (Springer-Verlag, 1974).

106. Moltke, I. & Albrechtsen, A. RelateAdmix: a software tool for estimating relatedness between admixed individuals. *Bioinformatics* **30,** 1027–1028 (2014).

107. Yang, W.-Y., Novembre, J., Eskin, E. & Halperin, E. A model-based approach for analysis of spatial structure in genetic data. *Nat. Genet.* **44,** 725–731 (2012).

108. van Gennep, A. *Les rites de passage: étude systématique des rites de la porte et du seuil, de l'hospitalité, de l'adoption, de la grossesse et de l'accouchement, de la naissance, de l'enfance, de la puberté, de l'initiation, de l'ordination, du couronnement des fiançailles et du mariage, des funérailles, des saisons, etc*. (1981).

109. Insoll, T. *The Archaeology of Identities: A Reader*. (Routledge, 2007).

110. Rummel, P. *Habitus barbarus*. (2007).

111. Brather, S. *Ethnische Interpretationen in der frühgeschichtlichen Archäologie: Geschichte, Grundlagen und Alternativen*. (Walter de Gruyter, 2004).

112. Halsall, G. *Cemeteries and Society in Merovingian Gaul: Selected Studies in History and Archaeology, 1992-2009*. (BRILL, 2010).

113. Hakenbeck, S., McManus, E., Geisler, H., Grupe, G. & O'Connell, T. Diet and mobility in Early Medieval Bavaria: a study of carbon and nitrogen stable isotopes. *Am. J. Phys. Anthropol.* **143,** 235–249 (2010).

114. Alexander Bentley, R. Strontium Isotopes from the Earth to the Archaeological Skeleton: A Review. *J Archaeol Method Theory* **13,** 135–187 (2006).

115. IAEA/WMO. Global Network of Isotopes in Precipitation. The GNIP Database. (2006).

116. Daux, V. *et al.* Oxygen isotope fractionation between human phosphate and water revisited. *J. Hum. Evol.* **55,** 1138–1147 (2008).

117. Pollard, A. M., Pellegrini, M. & Lee-Thorp, J. A. Technical note: some observations on the conversion of dental enamel δ18O(p) values to δ18O(w) to determine human mobility. *Am. J. Phys. Anthropol.* **145,** 499–504 (2011).

118. Lightfoot, E. & O'Connell, T. C. On the Use of Biomineral Oxygen Isotope Data to Identify Human Migrants in the Archaeological Record: Intra-Sample Variation, Statistical Methods and Geographical Considerations. *PLoS One* **11,** e0153850 (2016).

119. Brettell, R., Montgomery, J. & Evans, J. Brewing and stewing: the effect of culturally mediated behaviour on the oxygen isotope composition of ingested fluids and the implications for human provenance studies. *J. Anal. At. Spectrom.* **27,** 778–785 (2012).

120. O'Leary, M. H. Carbon Isotopes in Photosynthesis. *Bioscience* **38,** 328–336 (1988).

121. Edgar Hare, P., Fogel, M. L., Stafford, T. W., Mitchell, A. D. & Hoering, T. C. The isotopic composition of carbon and nitrogen in individual amino acids isolated from modern and fossil proteins. *J. Archaeol. Sci.* **18,** 277–292 (1991).

122. Ambrose, S. H. Stable carbon and nitrogen isotope analysis of human and animal diet in Africa. *J. Hum. Evol.* **15,** 707–731 (1986).

123. Castiglioni, E. & Rottoli, M. Broomcorn millet, foxtail millet and sorghum in north Italian Early Medieval sites. *Eur. J. Postclassical Archaeol* **3,** 131–144 (2013).

124. Bocherens, H. & Drucker, D. Trophic level isotopic enrichment of carbon and nitrogen in bone collagen: case studies from recent and ancient terrestrial ecosystems. *Int. J. Osteoarchaeol.* **13,** 46–53 (2003).

125. Hedges, R. E. M. & Reynard, L. M. Nitrogen isotopes and the trophic level of humans in archaeology. *J.*

Archaeol. Sci. **34,** 1240–1251 (2007).

126. O'Connell, T. C., Kneale, C. J., Tasevska, N. & Kuhnle, G. G. C. The diet-body offset in human nitrogen isotopic values: a controlled dietary study. *Am. J. Phys. Anthropol.* **149,** 426–434 (2012).
127. Dufour, E., Bocherens, H. & Mariotti, A. Palaeodietary Implications of Isotopic Variability in Eurasian Lacustrine Fish. *J. Archaeol. Sci.* **26,** 617–627 (1999).
128. Maurer, A.-F. *et al.* Bioavailable 87Sr/86Sr in different environmental samples--effects of anthropogenic contamination and implications for isoscapes in past migration studies. *Sci. Total Environ.* **433,** 216–229 (2012).
129. Woelfel, J. B. & Scheid, R. C. *Dental anatomy: its relevance to dentistry*. (Lippincott Williams & Wilkins, 2012).
130. Bickle, M. J. *et al.* Fluxes of Sr into the headwaters of the Ganges. *Geochim. Cosmochim. Acta* **67,** 2567–2584 (2003).
131. Balasse, M., Ambrose, S. H., Smith, A. B. & Price, T. D. The Seasonal Mobility Model for Prehistoric Herders in the South-western Cape of South Africa Assessed by Isotopic Analysis of Sheep Tooth Enamel. *J. Archaeol. Sci.* **29,** 917–932 (2002).
132. Coplen, T. B. New IUPAC guidelines for the reporting of stable hydrogen, carbon, and oxygen isotope-ratio data. *J. Res. Natl. Inst. Stand. Technol.* **100,** 285 (1995).
133. Hoefs, J. *Stable Isotope Geochemistry*. (2009).
134. Privat, K. L., O'Connell, T. C. & Richards, M. P. Stable isotope analysis of human and faunal remains from the Anglo-Saxon cemetery at Berinsfield, Oxfordshire: dietary and social implications. *J. Archaeol. Sci.* **29,** 779–790 (2002).
135. DeNiro, M. J. Postmortem preservation and alteration of in vivo bone collagen isotope ratios in relation to palaeodietary reconstruction. *Nature* **317,** 806 (1985).
136. Ambrose, S. H. Preparation and characterization of bone and tooth collagen for isotopic analysis. *J. Archaeol. Sci.* **17,** 431–451 (1990).
137. Schoeninger, M. J. & DeNiro, M. J. Nitrogen and carbon isotopic composition of bone collagen from marine and terrestrial animals. *Geochim. Cosmochim. Acta* **48,** 625–639 (1984).

138. van der Merwe, N. J. & Vogel, J. C. 13C content of human collagen as a measure of prehistoric diet in woodland North America. *Nature* **276,** 815–816 (1978).

139. Lee-Thorp, J. A. On isotopes and old bones. *Archaeometry* **50,** 925–950 (2008).

140. Hedges, R. E. M., Clement, J. G., Thomas, C. D. L. & O'Connell, T. C. Collagen turnover in the adult femoral mid-shaft: modeled from anthropogenic radiocarbon tracer measurements. *Am. J. Phys. Anthropol.* **133,** 808–816 (2007).

141. de Jong, M. An Unresolved Riddle: Early Medieval Incest Legislation. in *Franks and Alamanni in the Merovingian Period: An Ethnographic Perspective* (ed. Wood, I.) (Boydell & Brewer Ltd, 2003).

142. Hakenbeck, S. E. Roman or barbarian? Shifting identities in early medieval cemeteries in Bavaria. *Post-Classical Archaeologies* **1,** 37–66 (2011).

143. Hakenbeck, S. 'Hunnic' modified skulls: physical appearance, identity and the transformative nature of migrations. in *Mortuary practices and social identities in the Middle Ages* (eds. Williams, H. & Sayer, D.) 64–80 (Exeter University Press, 2009).

144. Czermak, A., Ledderose, A., Strott, N., Meier, T. & Grupe, G. Social Structures and Social Relations—An Archaeological and Anthropological Examination of three Early Medieval Separate Burial Sites in Bavaria. *Anthropol. Anz.* 297–310 (2006).

145. Strott, N., Czermak, A. & Grupe, G. Are biological correlates to social stratification depicted in skeletal finds? Investigation of early medieval separate burial grounds in Bavaria. in *Skeletal Series and their Socio-Economic Context* (eds. Grupe, G. & Peters, J.) **5,** 68–86 (Marie Leidorf, 2008).

146. Knipper, C. *et al.* Superior in Life—Superior in Death: Dietary Distinction of Central European Prehistoric and Medieval Elites. *Curr. Anthropol.* **56,** 579–589 (2015).

147. Iacumin, P., Galli, E., Cavalli, F. & Cecere, L. C4-consumers in southern Europe: The case of Friuli VG (NE-Italy) during early and central middle ages. *Am. J. Phys. Anthropol.* **154,** 561–574 (2014).

148. Kiss, A. Das germanische Gräberfeld von Hács-Béndekpuzsta (Westungarn) aus dem 5.-6. Jahrhundert. *Acta Antiquae Academiae Scientiarum Hungaricae* **36,** 275–342 (1995).

149. Straub, P. Angaben zum hunnenzeitlichen ostgermanischen Fundhorizont in Südtransdanubien—Ausgehend von der Nekropole in Keszthely-Fenékpuszta. *Keszthely-Fenékpuszta in Kontext spätantiker*

Kontinuitätsforschung zwischen Noricum und Moesia. Castellum Pannonicum Pelsonense. Rahden/Westf. : Marie Leidorf 325–345 (2011).

150. Hakenbeck, S. E., Evans, J., Chapman, H. & Fóthi, E. Practising pastoralism in an agricultural environment: An isotopic analysis of the impact of the Hunnic incursions on Pannonian populations. *PLoS One* **12,** e0173079 (2017).
151. Sage, W. Das Reihengräberfeld von Altenerding in Oberbayern I.--Germanische Denkmäler der Völkerwanderungszeit, Serie A, 14, Gebr. (1984).
152. Geisler, H., Ganslmeier, R. & Christlein, R. *Das frühbairische Gräberfeld Straubing-Bajuwarenstrasse I: Katalog der archäologischen Befunde und Funde*. (Leidorf, 1998).
153. Schutkowski, H., Herrmann, B., Wiedemann, F., Bocherens, H. & Grupe, G. Diet, Status and Decomposition at Weingarten: Trace Element and Isotope Analyses on Early Mediaeval Skeletal Material. *J. Archaeol. Sci.* **26,** 675–685 (1999).
154. Grossi, G. L. A. *Guida alle cascine e vigne del territorio di Torino e suoi contorni*. (Torino : [s.n.], 1790).
155. Giostra, C. La struttura sociale nelle necropoli longobarde italiane: una lettura archeologica. in *Archeologia dei Longobardi: dati e metodi per nuovi percorsi di analisi* (ed. Giostra, C.) 83–112 (2017).
